# CF_3_‐Containing *para*‐Quinone Methides for Organic Synthesis

**DOI:** 10.1002/ejoc.202000295

**Published:** 2020-03-30

**Authors:** Michael Winter, Roman Schütz, Andreas Eitzinger, Armin R. Ofial, Mario Waser

**Affiliations:** ^1^ Institute of Organic Chemistry Johannes Kepler University Linz Altenbergerstraße 69 4040 Linz Austria; ^2^ Department Chemie Ludwig‐Maximilians‐Universität München Butenandtstraße 5‐13 81377 München Germany

**Keywords:** Quinone methides, Trifluoromethylation, Electrophilicity, Kinetics, Phase‐transfer catalysis

## Abstract

A new family of CF_3_‐containing *para*‐quinone methides (CF_3_‐QMs) was systematically investigated for its suitability in organic synthesis. Addition of different nucleophiles gives access to target molecules with a benzylic CF_3_‐containing stereogenic center straightforwardly. The electrophilicity parameter *E* of the prototypical CF_3_‐QM 2,6‐di‐*tert*‐butyl‐4‐(2,2,2‐trifluoroethylidene)cyclohexa‐2,5‐dien‐1‐one was determined to be –11.68 according to the Mayr scale, making it one of the most reactive quinone methides known so far.

## Graphical abstract

Novel CF_3_‐containing para‐quinone methides were systematically investigated for their use in (asymmetric) transformations.

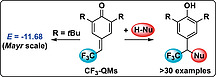

## Introduction

**Scheme 1:**
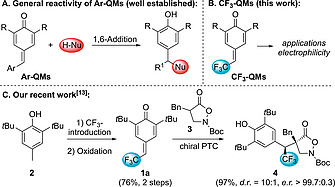
(A) General reactivity mode of p‐QMs. (B) The herein targeted investigations concerning novel CF_3_‐QMs. (C) Our previous report describing the synthesis of CF_3_‐QM **1a** and its use in a single asymmetric transformation.^13^

*para*‐Quinone methides (p‐QMs) have emerged as versatile reagents for a variety of different (asymmetric) transformations over the last years.^1^ Most commonly, arylidene‐based p‐QMs (Ar‐QMs) have been utilized as highly electrophilic acceptor molecules,^2^ which easily undergo 1,6‐addition reactions with a multitude of C‐ or hetero‐atom nucleophiles under a variety of (catalytic) conditions (Scheme 1A).^1^, ^3^, ^4^, ^5^, ^6^ Surprisingly, however, alternative ylidene groups have been much less explored so far.^1^

Given the broad interest in CF_3_‐containing (chiral) organic molecules,^7^, ^8^ we reasoned that the introduction and systematic investigation of CF_3_‐based *para*‐quinone methides (CF_3_‐QMs) may be a worthwhile task to establish a new platform of versatile prochiral starting materials (Scheme 1B).^8^ While racemic trifluoromethylation reactions of arylidene‐based Ar‐QMs have been reported before,^9^ reactions of the CF_3_‐containing acceptor compounds CF_3_‐QMs with different nucleophiles would result in an unprecedented and complementary synthesis strategy to access a broad variety of (chiral) target molecules straightforwardly. Furthermore, to get a more comprehensive understanding about the reactivity of such novel electrophiles and to predict new reactions thereof as well as to obtain a direct comparison with the well‐established Ar‐QMs,^2^ it would be beneficial to determine the electrophilicity parameter *E* (according to Mayr's free energy relationship^10^) of at least one new CF_3_‐QM derivative.

Interestingly, in 1985 already Umemoto and co‐workers described the formation of a CF_3_‐containing p‐QM^11^ and the intermediate formation of CF_3_‐containing p‐QMs was also observed in reactions of trifluoroethanol‐containing phenols some years ago,^12^ but apart from these two reports, such potentially useful building blocks have not been reported and utilized for further transformations until very recently, when we described the first synthesis of the CF_3_‐QM **1a**.^13^, ^14^ Starting from the bulk chemical **2**, p‐QM **1a** could be obtained in two steps via a benzylic trifluoromethylation first,^15^ followed by oxidation to the QM (Scheme 1C).^13^, ^16^ This compound was then successfully used in the asymmetric phase‐transfer catalyst (PTC) controlled reaction with pronucleophile **3** to access the masked β^2,2^‐amino acid derivative **4** with very high levels of stereoselectivity.^13^

Considering this very promising initial application of the novel building block **1a**, we now became interested in investigating this novel quinone methide‐platform more systematically (Scheme 1B). Besides utilizing **1a** for a variety of different transformations, we also wanted to install alternative groups R at the phenol part, and we became interested in determining the electrophilicity parameter *E* for at least one (stable) derivative.

## Results and Discussion

### *E*‐Parameter

Arylidene‐based p‐quinone methides Ar‐QMs have been studied by Mayr's group several years ago. They analyzed the second‐order rate constants of the reactions of Ar‐QMs with several nucleophiles^2^ according to the following linear free energy relationship [Equation (1)].^10^

(1) $$\mathrm{lg}k(\mathrm{20}\;\mathrm{{}^{\circ}C})=s_{N}(N+E)$$

The availability of electrophilicity parameters *E* for certain acceptor molecules, such as Ar‐QMs or CF_3_‐QMs, not only allows for a reactivity comparison between different electrophilic compounds, but also provides a tool for the reliable prediction of novel reactions upon considering the *E*‐parameter of the electrophile and the *N*‐parameter of the nucleophile.^10^ In general, if (*E* + *N*) > –5 is fulfilled, a certain electrophile/nucleophile‐combination can be expected to react at room temperature.

**Scheme 2:**
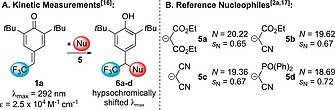
Kinetic measurements of the reactions of **1a** with the reference nucleophiles **5a** (Solvent: DMSO; counteranion for **5a**‐**d**; K^+^).

Thus, we started our investigations by determining the *E*‐parameter of the stable CF_3_‐QM **1a** by studying the kinetics of its reactions with the carbanions **5a**–**d**[2a], 17 as reference nucleophiles in DMSO at 20 °C (Scheme 2).

**Figure 1:**
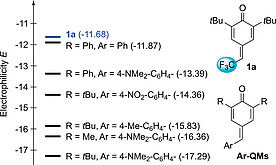
Electrophilicity *E* of **1a** in comparison with established arylidene‐based p‐QMs.^2^

The CF_3_‐QM **1a** has a *λ*_max_ of 292 nm (in DMSO) and a molar absorption coefficient of 2.5 × 10^4^ m ^–^^1^ cm^–1^, which enabled us to follow its reactions with the colorless nucleophiles **5a**–**d** to the hypsochromically shifted phenol derivatives **6a**‐**d** photometrically (**5a‐d** were generated by deprotonation of the pronucleophiles **5‐H** with 0.5 equiv. of KO*t*Bu).^16^ As well established for other nucleophile/electrophile combinations before,^2^, ^10^, ^17^ the reaction kinetics were determined by employing stopped‐flow UV/Vis photometry to follow the fading of the colored **1a** upon reaction with a large excess of the carbanions **5** (resulting in absorbance decays that follow first‐order kinetics). Based on these measurements, it was then possible to calculate the corresponding experimental second‐order rate constants for these four reactions.^2^, ^10^, ^16^ By using these rate constants together with the known parameters (*N, s*_N_) of our reference nucleophiles,^2^, ^17^ it was finally possible to determine the electrophilicity parameter *E* for CF_3_‐QM **1a** being –11.68.^16^ By comparing this value with *E*‐parameters for well‐known *tert*‐butyl‐substituted Ar‐QMs^2^ (Figure 1), it becomes obvious that the presence of the CF_3_‐group significantly boosts the electrophilicity of **1a**, which makes it a very promising building block for further transformations: The CF_3_‐QM **1a** should be capable of reacting with various types of nucleophiles provided that their nucleophilic reactivity parameter *N* exceeds the value of +7.

### Syntheses of CF_3_‐QMs

**Scheme 3:**
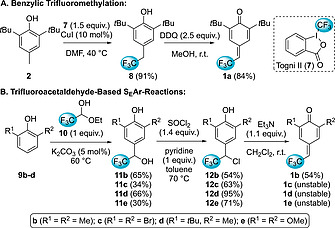
Synthesis strategies to access CF_3_‐QMs **1a–e**.

For the synthesis of the *t*Bu‐containing **1a** our recently developed strategy starting from phenol **2** turned out to be highly reproducible and was easily carried out on several gram scale (Scheme 3A).^13^ Hereby, the CF_3_‐group was first installed by means of a benzylic trifluoromethylation of **2** using Togni's second generation reagent **7** 18 in analogy to a recently published procedure.^16^ Conversion of **8** to the quinone methide **1a** was then achieved by employing 2,3‐dichloro‐5,6‐dicyano‐benzoquinone (DDQ) as an oxidant.

Unfortunately, this route was found to be not suited for other phenol derivatives (e.g. when using mesitylene derivatives to access QM **1b**, the radical trifluoromethylation was not site‐selective and the subsequent purification and oxidation turned out to be rather problematic). Therefore, we opted for a different strategy to access the alternatively substituted QMs **1b**‐**e** next. It was shown before,^12^, ^19^ that phenol **9b** can be converted into the CF_3_‐containing benzylic chloride **12b** in a two‐step procedure by first carrying out an S_E_Ar‐reaction with trifluoroacetaldehyde hemiacetal **10** (giving alcohol **11b**), followed by chlorination using SOCl_2_ (Scheme 3B). Interestingly, compound **12b** already contains traces of the corresponding QM **1b** and we found that treatment with Et_3_N allows for the direct formation of **1b** with a reasonable isolated yield of 54 %. It should however be noted that the dimethyl‐QM **1b** was found to be significantly less stable than the di‐*tert*‐butyl‐QM **1a**, making its purification and isolation a difficult task. The benzylic chlorides **12c‐e** could be accessed analogously from the corresponding phenols **9c–e**, but in neither of these cases was it possible to isolate the final QMs **1c–e** upon treatment with base. Interestingly however, as compounds **12c‐e** degraded quickly under basic conditions, and given the fact that we were able to detect traces of QMs **1** in crude samples of precursors **12** already, it seems very likely that the less‐stable QMs **1c–e** are actually formed from **12c‐e** under basic conditions and that it may be possible to generate and utilize them for further transformations in situ (vide infra).

We also tested if the S_E_Ar strategy outlined in Scheme 3B may be applicable to the corresponding di‐*t*Bu‐phenol **9a**, but surprisingly in this case the yields were rather low and unpractical (compared to the route outlined in Scheme 3A).

### Application Scope

**Scheme 4:**
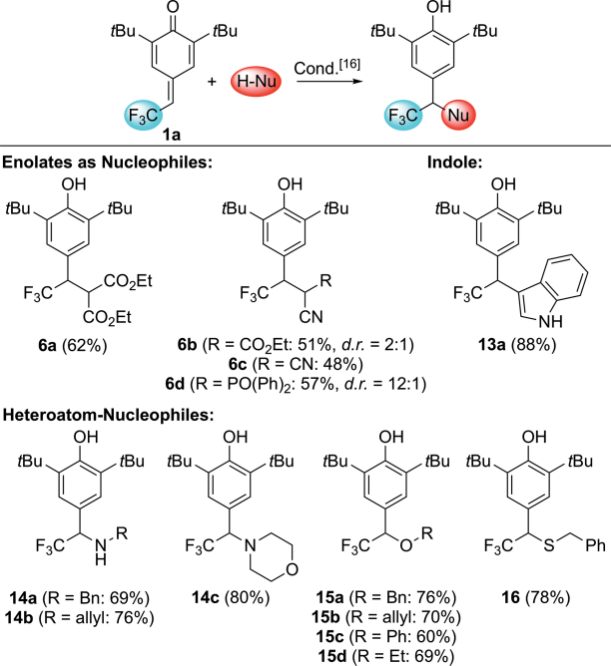
Racemic addition of different nucleophiles to QM **1a**.^16^

As the *t*Bu‐QM **1a** was found to be the most stable of the so far accessed CF_3_‐QM derivatives **1**, we started our investigations of the application scope by reacting **1a** with a variety of C‐ and heteroatom‐nucleophiles (Scheme 4).

All the racemic products shown in Scheme 4 were obtained straightforwardly under operationally simple conditions,^16^ giving access to a variety of trifluoromethylated functionalized phenol derivatives. The moderate yields obtained for products **6** can to some extent be rationalized by a certain sensitivity of these products under basic reaction conditions in the presence of an excess of nucleophile. The synthesis of indole derivative **13a** performed well under established Lewis acid (BF_3_ **·**OEt_2_)‐mediated conditions.^20^ In sharp contrast, when reacting **1a** with indole in the absence of any Lewis acid, no reaction occurs. This result is in perfect accordance with the hypothesis that a given electrophile/nucleophile‐combination can be expected to react at room temperature if (*E* + *N*) > –5,^10^ as the experimentally determined nucleophilicity of indole (*N* < 5.6)^21^ is too low to fulfil this requirement in combination with **1a** (*E* = –11.68) in the absence of any Lewis acid. It should be noted that we also tested the addition of NaBH_4_ and NaBD_4_ to QM **1a**, which resulted in quantitative formation of precursor **8** and the monodeuterated **8‐D** respectively.^16^

**Scheme 5:**
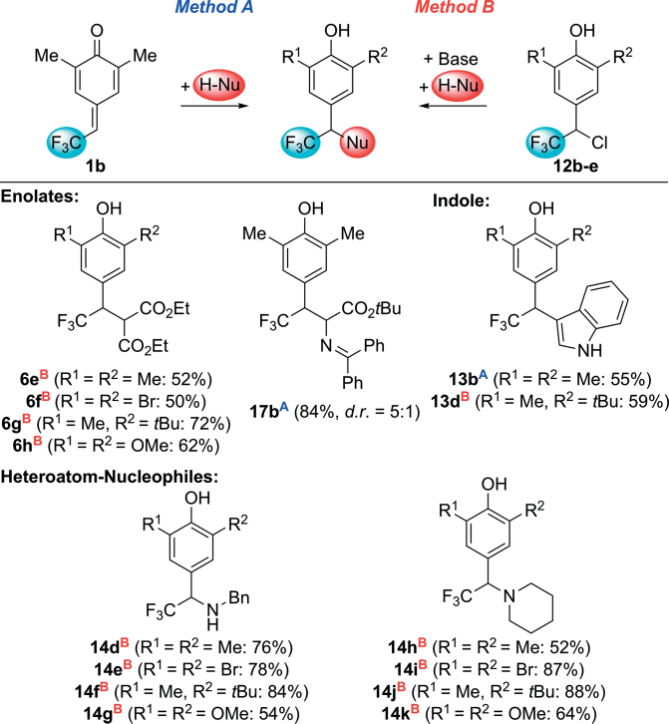
Reactions of QM **1b** and QM‐precursors **12b**–**e**.^16^

Having established that the most stable QM **1a** reacts well with a variety of different nucleophiles, we next focused on the use of QM **1b** (preformed and/or in situ formed from **12b**) and on QM‐precursors **12c‐e** (Scheme 5).^16^ First experiments carried out with **1b** allowed for the straightforward synthesis of the glycine Schiff base‐containing **17b** and the indole‐based **13b**. The later reaction again required the use of BF_3_ **·**OEt_2_ as a Lewis acid, indicating that the dimethyl QM **1b** is not significantly more electrophilic than the di‐*tert*‐butyl QM **1a** (vide supra). We next tested if it may be possible to use QM precursors like **12d** directly for the reaction with indole. When **12d** was treated with indole and BF_3_ **·**OEt_2_ in the absence of any base, no product **13d** was formed. However, when **12d** was first stirred with one equivalent of Et_3_N, before adding indole and BF_3_ **·**OEt_2_, the target **13d** was obtained straightforwardly. This strongly supports our hypothesis that the in situ formation and utilization of QMs **1** from precursors **12** is indeed possible. Thus, we next reacted **12b–e** with diethyl malonate and two different amines in the presence of Cs_2_CO_3_, which allowed for the direct formation of the products **6e–h** and **14d–k** in reasonable yields as well.

**Scheme 6:**
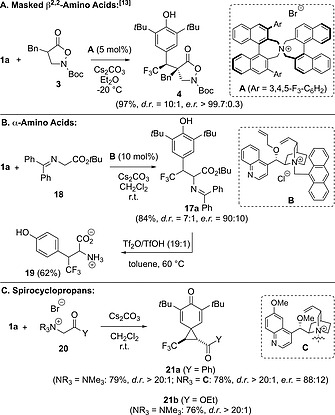
Asymmetric transformations of **1a** and debutylation/deprotection strategy for the synthesis of the free amino acid **19**.^16^

Finally, as QM **1a** could be successfully employed for the highly asymmetric phase transfer‐catalyzed synthesis of the masked β^2,2^‐amino acid derivative **4** (Scheme 6A),^13^ we also became interested in testing the two other asymmetric transformations depicted in Scheme 6. First, **1a** was treated with the glycine Schiff base **18** 22 under phase‐transfer conditions to access the protected α‐amino acid derivative **17a**. The asymmetric addition of nucleophiles **18** to Ar‐QMs was recently reported by the groups of Fan and Deng,^23^ and in analogy to Fan's work, the use of Cinchona alkaloid‐based chiral PTCs, i.e. ammonium salt **B**, allowed for reasonable selectivities for the addition of **18** to **1a** (Scheme 6B). Unfortunately, it was not possible to assign the relative and absolute configuration of enantioenriched **17a** as we were not able to obtain single crystals of sufficient quality for a detailed X‐ray analysis. However, it was easily possible to fully deprotect and debutylate **17a** under acidic conditions, which gave a direct entry to β‐CF_3_‐α‐tyrosine **19** (Scheme 6B).

We have recently shown that Ar‐QMs can also undergo highly enantioselective spirocyclopropanation reactions with chiral ammonium ylides (formed in situ upon deprotonation of ammonium salts **20**).[6e] While the addition of achiral ammonium salts **20** (with R_3_N = Me_3_N) to CF_3_‐QM **1a** yielded the racemic *trans*‐spirocyclopropanes **21a** and **21b** straightforwardly (Scheme 6C), the asymmetric synthesis turned out to be more difficult (i.e. compared to the highly asymmetric spirocyclopropanation of Ar‐QMs[6e]). When testing different chiral ammonium ylide‐precursors **20**, we found that Cinchona alkaloids allow for some moderate enantioselectivity in the synthesis of **21a** only, but unfortunately the synthesis of **21b** turned out to be rather sluggish and unselective. While this example demonstrates the sometimes significant reactivity difference of achiral and chiral ammonium ylides in asymmetric cyclization reactions,^24^, ^25^ it still serves as another illustrative example about the general use of CF_3_‐QMs **1** for asymmetric transformations.

### CF_2_H‐Quinone Methide

Difluoromethyl (CF_2_H)‐containing (chiral) molecules have attracted more and more attention recently,^26^ especially because CF_2_H‐containing compounds show unique properties like e.g. H‐bonding capability, in comparison to analogous CF_3_‐derivatives.^27^ This results in a powerful tool to alter the properties of a given structural motif by subtle structural changes only, and we were therefore interested to obtain a first proof‐of‐concept if our herein developed strategy to access and utilize the CF_3_‐QMs **1b‐e** in situ, starting from the benzylic chlorides **12b–e**, may also be useful to access analogous and so far unprecedented CF_2_H‐QMs.

**Scheme 7:**
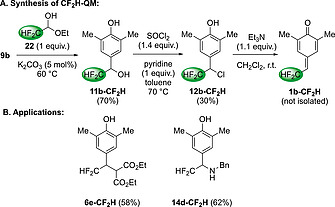
(A) Synthesis route to access the QM‐precursor **12b‐CF_2_H** and (B) applications thereof.^16^

The difluoromethyl‐based hemiacetal **22** is commercially available and we were glad to see that the synthesis of the QM‐precursor **12b‐CF_2_H** could be carried in the same manner (Scheme 7A) as for the CF_3_‐analogs (compare with Scheme 3B). NMR of purified **12b‐CF_2_H** showed around 10–15 % of the target quinone methide **1b‐CF_2_H**, but unfortunately we were not able to isolate this QM after treatment with base (decomposition). Nevertheless, it was again possible to use the precursor **12b‐CF_2_H** directly for further manipulations, as demonstrated for the synthesis of the two CF_2_H‐phenol derivatives **6e‐CF_2_H** and **14db‐CF_2_H** (Scheme 7B).

## Conclusions

A new family of either preformed, or in situ generated, CF_3_‐containing *para*‐quinone methides was developed and systematically investigated for their use as starting materials to access phenol derivatives with a CF_3_‐substituted benzylic stereogenic center. In addition, we determined the electrophilicity parameter *E* (according to Mayr's linear free energy relationship) for one derivative being –11.68. This is one of the highest *E* parameters for a quinone methide so far, making these quinone methides very useful and highly reactive building blocks that undergo 1,6‐addition reactions with a variety of C‐ and heteroatom nucleophiles straightforwardly. In addition, it was also shown that an analogous novel CF_2_H‐QM precursor can be accessed in a similar manner and utilized directly for further manipulations.

## Experimental Section

General details can be found in the online supporting information. This document also contains detailed synthesis procedures and analytical data of all novel compounds and reaction products, details about the kinetic investigations to determine the *E* parameter, as well as copies of NMR spectra and HPLC traces.

Synthesis of QM **1a**:

Step 1: CuI (190 mg; 1 mmol) and Togni's reagent **7** (4.74 g; 15 mmol) were dissolved in 50 mL of DMF. Then the phenol **2** (2.2 g; 10 mmol) was added and the mixture was stirred at 40 °C. After 1 h the reaction mixture was diluted with EtOAc and washed with NaHCO_3_. The organic layer was dried with Na_2_SO_4_, filtered, and concentrated in vacuo. The crude product was purified by column chromatography on silica gel (Heptanes/EtOAc = 20:1) to yield the product **8** in 91 % (2.62 g; 9.1 mmol).

Step 2: The trifluoroethylated phenol **8** (2.88 g; 10 mmol) was dissolved in 200 mL of MeOH and DDQ (5.67 g; 25 mmol) was added. The reaction mixture was stirred at room temperature for 1 h. After completion of the reaction, the solvent was evaporated, and the crude reaction mixture was purified by column chromatography (heptanes/CH_2_Cl_2_ = 10:1) to afford product **1a** in 84 % yield (2.4 g; 8.4 mmol).

Analytic details of** 1a**: m.p. 34.5–35.0 °C; ^1^H NMR (700 MHz, CDCl_3_, 298 K): *δ* = 7.33 (s, 1H), 6.78 (s, 1H), 6.03 (q, *J* = 8.9 Hz, 1H), 1.29 (s, 9H), 1.28 (s, 9H) ppm; ^19^F‐NMR (282 MHz, CDCl_3_, 298 K): *δ* = –55.40 (d, *J* = 8.9 Hz, 3F) ppm; ^13^C‐NMR (125 MHz, CDCl_3_, 298 K): *δ* = 186.2, 151.7, 151.4, 138.4 (q, *J* = 5.5 Hz), 132.4, 125.3, 124.1 (q, *J* = 34.7 Hz), 123.0 (q, *J* = 271.0 Hz), 35.8, 35.4, 29.5 ppm. HRMS (ESI): *m/z* calculated for C_16_H_21_F_3_O: 317.1734 [M – H + MeOH]^–^, found 317.1730.

General Synthesis of QM‐precursors **12** and Synthesis of QM **1b**:

The corresponding phenol **9** (30 mmol) and trifluoroacetaldehyde hemiacetal **10** (30 mmol) were mixed and K_2_CO_3_ (1.5 mmol) was added. The mixture was heated to 60 °C and stirred for 16 h. After cooling to room temperature, the reaction mixture was dissolved with ethyl acetate and washed with ammonium chloride, H_2_O and brine. The organic layer was dried with Na_2_SO_4_ and the solvents evaporated to dryness. The crude products **11** were directly used for the next step.

Step 2: A mixture of compound **11** (10 mmol) and SOCl_2_ (14 mmol) in 15 mL dry toluene was cooled to 0–5 °C and pyridine (10 mmol) was added. After 1 h the mixture was heated to 70 °C and stirred for another 2 h. After cooling to room temperature, the mixture was poured on 20 g ice and stirred for another 30 min. Then the organic layer was separated, and the aqueous layer was extracted with ethyl acetate twice. The combined organic layers were dried with Na_2_SO_4_ and the solvents evaporated to dryness. The crude reaction mixture was purified by column chromatography (CH_2_Cl_2_/heptanes = 2:1) giving the products **12** in the yields reported in Scheme 3.

Step 3 (Synthesis of **1b**): Precursor **12b** (1.19 g; 5 mmol) was dissolved in 20 mL CH_2_Cl_2_ and triethylamine (0.76 mL; 5.5 mmol) was added. The reaction mixture was stirred at room temperature overnight (completion shown by TLC) and H_2_O was added. The organic layer was washed with 1 N HCl followed by NaHCO_3_ (sat.) and brine. After drying the organic layer over Na_2_SO_4_ it was evaporated to dryness and the quinone methide **1b** was isolated by column chromatography (heptanes/EtOAc = 20:1–1:1) in 54 % yield (0.55 g; 2.7 mmol).

Analytic details of** 1b**: ^1^H NMR (300 MHz, CDCl_3_, 298 K): *δ* = 7.34 (s, 1H), 6.84 (s, 1H), 6.00 (q, *J* = 9.0 Hz, 1H), 2.05 (s, 3H), 2.02 (s, 3H) ppm; ^19^F‐NMR (282 MHz, CDCl_3_, 298 K): *δ* = –55.40 (d, *J* = 9 Hz, 3F) ppm; ^13^C‐NMR (125 MHz, CDCl_3_, 298 K): *δ* = 186.9, 140.0, 139.4, 137.8 (q, *J* = 5.5 Hz), 135.8, 128.7, 124.0 (q, *J* = 34.8 Hz), 122.7 (q, *J* = 271.7 Hz), 16.8, 16.2 ppm. HRMS (ESI): *m/z* calculated for C_10_H_9_F_3_O: 201.0533 [M – H]^–^, found 201.0535.

Analytic details of** 12c**: ^1^H NMR (300 MHz, CDCl_3_, 298 K): *δ* = 7.60 (s, 2H), 6.07 (s, 1H), 5.00 (q, *J* = 6.7 Hz, 1H) ppm; ^19^F‐NMR (282 MHz, CDCl_3_, 298 K): *δ* = –73.45 (d, *J* = 6.7 Hz, 3F) ppm; ^13^C‐NMR (125 MHz, CDCl_3_, 298 K): *δ* = 151.1, 132.5, 128.7, 123.2 (q, *J* = 278.8 Hz), 110.2, 50.0 (q, *J* = 34.4 Hz) ppm. HRMS (ESI): *m/z* calculated for C_8_H_4_Br_2_ClF_3_O: 366.8342 [M + H]^+^, found 366.8348.

Analytic details of** 12d**: ^1^H NMR (300 MHz, CDCl_3_, 298 K): *δ* = 7.22 (s, 1H), 7.17 (s, 1H), 5.03 (q, *J* = 7.0 Hz, 1H), 2.27 (s, 3H), 1.42 (s, 9H) ppm; ^19^F‐NMR (282 MHz, CDCl_3_, 298 K): *δ* = –73.21 (d, *J* = 7.0 Hz, 3F) ppm; ^13^C‐NMR (125 MHz, CDCl_3_, 298 K): *δ* = 154.2, 138.0, 136.1, 129.2, 128.8, 128.4, 126.1, 125.4, 123.7, 123.7 (q, *J* = 279.1 Hz), 123.5, 59.2 (q, *J* = 34.1 Hz), 34.7, 29.7, 16.1 ppm. HRMS (ESI): *m/z* calculated for C_13_H_16_ClF_3_O: 281.0915 [M + H]^+^, found 281.0915.

Analytic details of** 12e**: ^1^H NMR (300 MHz, CDCl_3_, 298 K): *δ* = 6.71 (s, 2H), 5.65 (s, 1H), 5.03 (q, *J* = 6.7 Hz, 1H), 3.92 (s, 6H) ppm; ^19^F‐NMR (282 MHz, CDCl_3_, 298 K): *δ* = –73.20 (d, *J* = 6.7 Hz, 3F) ppm; ^13^C‐NMR (125 MHz, CDCl_3_, 298 K): *δ* = 147.1, 136.4, 129.1, 128.4, 125.4, 123.8 (q, *J* = 282.8 Hz), 123.1, 105.8, 59.3 (q, *J* = 35.0 Hz), 56.6 ppm. HRMS (ESI): *m/z* calculated for C_10_H_10_ClF_3_O_3_: 271.0343 [M + H]^+^, found 271.0339.

## Supporting information

Supporting Information
