# Supplementary material for: CF3‐Containing para‐Quinone Methides for Organic Synthesis
Source: European J Org Chem. 2020 Mar 30;2020(25):3812–7. doi: 10.1002/ejoc.202000295 (PMC7335660; doi:10.1002/ejoc.202000295)
Supplement: Supplementary file 1 — Supporting Information [file EJOC-2020-3812-s001.pdf]

European Journal of Organic Chemistry

Supporting Information

CF_3-Containing *para*-Quinone Methides for Organic Synthesis

Michael Winter, Roman Schütz, Andreas Eitzinger, Armin R. Ofial, and Mario Waser*

1. General Information: 2
1.1. General Methods 2
1.2. Kinetic Investigations (General Details) 2
2. Syntheses, Kinetic Studies, and Application Scope 3
2.1 Syntheses of the Quinone-Methides and Quinone-Methide Precursors 3
2.2. Kinetics 9
2.3 Products 6a-d of the Kinetic Studies 13
2.4 Racemic Reactions with C- and Heteroatom Nucleophiles 15
2.5 Reactions with Indole 23
2.6 Spirocyclopropanation reaction 25
2.7 Reactions with Glycine Schiff Base 27
2.8 Deprotection and Debutylation of 17a 29
2.9 Hydrid- and deuterid-reduction of quinone methide 1a 30
3. Copies of NMR-Spectra of new Compounds 31

1. General Information:

1.1. General Methods

^{1}H-, ^{19}F- and ^{13}C-NMR spectra were recorded on a Bruker Avance III 300 MHz spectrometer with a broad band observe probe and a sample changer for 16 samples, on a Bruker Avance DRX 500 MHz spectrometer, and on a Bruker Avance III 700 MHz spectrometer with an Ascend magnet and TCI cryoprobe which are property to the Austro-Czech NMR-Research Center "RERI-uasb". All NMR spectra were referenced on the solvent peak. High resolution mass spectra were obtained using an Agilent 6520 Q-TOF mass spectrometer with an ESI source and an Agilent G1607A coaxial sprayer or a Thermo Fisher Scientific LTQ Orbitrap XL with an Ion Max API Source. Preparative column chromatography was carried out using Davisil LC 60A 70-200 MICRON silica gel. TLC probes were detected at 254 nm or stained with an appropriate staining solution (compare section 3.1.3). HPLC was performed using a Dionex Summit HPLC system with a Chiralcel YMC-SB (250 x 4.6 mm, 5 µm) and a Chiralpak AD-H (4.6 mm x 250 mm, 5 µm) chiral stationary phase. All chemicals were purchased from commercial suppliers and used without further purification unless otherwise stated. All reactions were carried out under Argon.

1.2. Kinetic Investigations (General Details)

UV-vis measurements of **1a** were done on a J&M TIDAS diode array spectrophotometer equipped with a Hellma 661.502-QX quartz Suprasil immersion probe (path d = 5 mm) and controlled by TIDASDAQ (v3) software. Kinetic studies were conducted photometrically at 20 °C (controlled by a circulating bath thermostat) in anhydrous DMSO (<50 ppm H_2O; commercial) using a stopped-flow spectrophotometer (Applied Photophysics SX.20MV-R) and stock solutions kept under nitrogen atmosphere by measuring the decay of the electrophile absorption (at λ_{max}). In order to achieve pseudo-first order kinetics, reference nucleophiles were used in large excess (up to 60 eq.). To show that the influence of the K^+-counterion or ion pairing can be neglected, 18-crown-6 ether was added for some kinetic measurements. CH-acids are added in excess (2 equivalents) with respect to the base to ensure a fast protonation of the intermediate reaction adduct.

The mono-exponential decay of the absorbance of **1a (CF$_3$QM)** upon reaction with reference nucleophiles **5** follows first-order kinetics $\left[A_t = A_0 e^{(-k_{obs} \cdot t)} + C\right]$ (eq. 1), giving observed rate constants k_{obs} (s^{-1}) by least-squares fitting. For each nucleophile concentration the average result of at least four runs is given. After plotting the nucleophile concentration against k_{obs} and applying a linear fit, second-order rate constants k_2 (M^{-1} s^{-1}) were obtained from the slope of the linear equation.

The electrophilicity parameter E is calculated using a nonlinear solver software for minimizing the squares of the deviations between calculated and experimental rate constants $\Delta^2 = \Sigma(\lg k_2 - s_N(N + E))^2$ (eq. 2).[1,2]

---

1 Ordering polar organic reactivity according to Equation 1 (main text): (a) H. Mayr, M. Patz, *Angew. Chem. Int. Ed. Engl.* **1994**, *33*, 938-957. (b) H. Mayr, T. Bug, M. F. Gotta, N. Hering, B. Irrgang, B. Janker, B. Kempf, R. Loos, A. R. Ofial, G. Remennikov, H. Schimmel, *J. Am. Chem. Soc.* **2001**, *123*, 9500-9512. (c) H. Mayr, A. R. Ofial, *Pure Appl. Chem.* **2005**, *77*, 1807-1821. (d) H. Mayr, A. R. Ofial, *SAR QSAR Environ. Res.* **2015**, *26*, 619-646. (e) H. Mayr, *Tetrahedron* **2015**, *71*, 5095-5111.

2 Reactivity parameters can be obtained from the online database at *www.cup.lmu.de/oc/mayr/DBintro.html*.

2. Syntheses, Kinetic Studies, and Application Scope

2.1 Syntheses of the Quinone-Methides and Quinone-Methide Precursors

General procedure 1 for the synthesis of 1a:

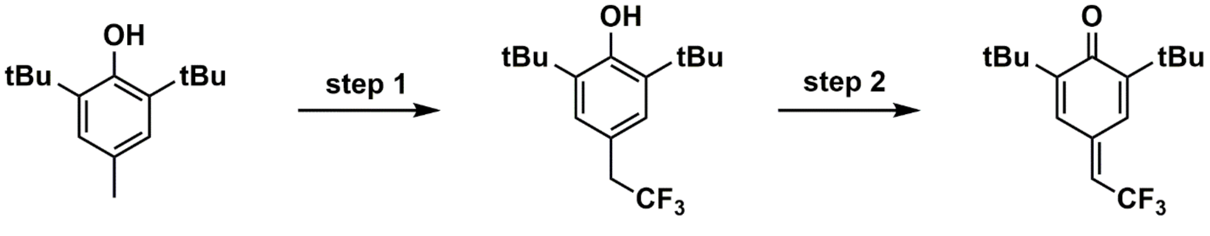

Step 1: The synthesis was performed according to a modified literature procedure.[3] CuI (1 mmol) and Togni reagent II (**7**, 15 mmol) were dissolved in 50 mL DMF. Then the phenol **2** (10 mmol) was added and the mixture was stirred at 40 °C. After 1 h the reaction mixture was diluted with EtOAc and washed with $NaHCO_3$. The organic layer was dried over Na_2SO_4, filtered and concentrated *in vacuo*. The crude product was purified by column chromatography on silica gel (heptanes/EtOAc = 20:1) to yield product **8** in 91 %.

Step 2: For the synthesis of compound **1a**, the trifluoroethylated phenol **8** (10 mmol) was dissolved in 200 mL MeOH and DDQ (25 mmol) was added. The reaction mixture was stirred at room temperature for 1 h. After completion of the reaction, the solvent was evaporated and the crude reaction mixture was purified by column chromatography (heptanes/dichloromethane = 10:1) to afford product **1a** in 84% yield.

[3] H. Egami, T. Ide, Y. Kawato, Y. Hamashima, *Chem. Commun.* **2015**, *51*, 16675-16678.

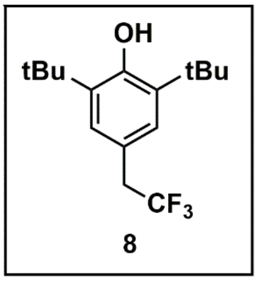

8

<u>8</u>: Compound was prepared according to the general procedure 1 step 1 in 91% yield. The product occurs as a colorless oil.

HRMS (ESI): m/z calculated for $C_{16}H_{21}F_3O$: 287.1628 $[M-H]^-$; found: 287.1628.

^{1}H-NMR (300 MHz, $CDCl_3$, 298 K): δ = 7.34 (s, 2H), 5.49 (s, 1H), 3.53 (q, J = 11.0 Hz, 2H), 1.71 (s, 18H) ppm; ^{19}F-NMR (282 MHz, $CDCl_3$, 298 K): δ = -66.05 (t, J = 11.0 Hz, 3F) ppm;

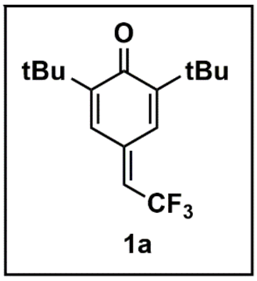

1a

<u>1a</u>: Prepared according to the general procedure 1, step 2 in 84% yield. The product occurs as yellow solid with a melting range of 34.5 – 35°C.

R_f (CH_2Cl_2:heptanes = 4:1) = 0.50.

HRMS (ESI): m/z calculated for $C_{16}H_{21}F_3O$: 317.1734 $[M-H+MeOH]^-$; found: 317.1730.

^{1}H-NMR (700 MHz, $CDCl_3$, 298 K): δ = 7.33 (s, 1H), 6.78 (s, 1H), 6.03 (q, J = 8.9 Hz, 1H), 1.29 (s, 9H), 1.28 (s, 9H) ppm; ^{19}F-NMR (282 MHz, $CDCl_3$, 298 K): δ = -55.40 (d, J = 8.9 Hz, 3F) ppm; ^{13}C-NMR (125 MHz, $CDCl_3$, 298 K): δ = 186.2, 151.7, 151.4, 138.4 (q, J = 5.5 Hz), 132.4, 125.3, 124.1 (q, J = 34.7 Hz), 123.0 (q, J = 271.0 Hz), 35.8, 35.4, 29.5 ppm.

General procedure 2:

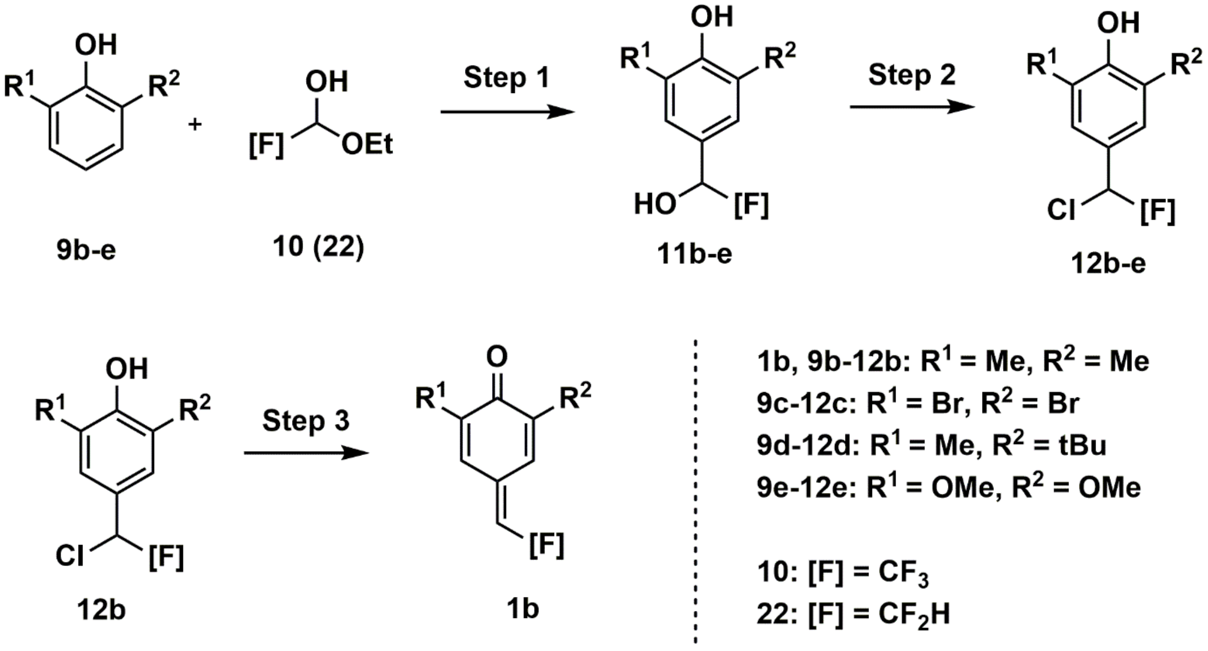

Step 1: The syntheses of compounds **11** were performed according to a modified literature procedure[4]. The corresponding phenol **9** (30 mmol) and trifluoroacetaldehyd ethyl hemiacetal **10** (30 mmol) or difluoroacetaldehyd ethyl hemiacetal **22** (30 mmol) were mixed and K_2CO_3 (1.5 mmol) was added. The mixture was heated to 60 °C and stirred for 16 h. After cooling the reaction mixture it was dissolved with ethyl acetate and washed with ammonium chloride, H_2O and brine. The organic layer was dried with Na_2SO_4 and evaporated to dryness. The crude products **11** were used directly in the next step.

Step 2: Compounds **12** were synthesized according to a modified literature procedure[5]. A mixture of phenol **11** (10 mmol) and $SOCl_2$ (14 mmol) in 15 mL abs. toluene was cooled to 0-5 °C and pyridine (10 mmol) was added. After 1 h the mixture was heated to 70 °C and stirred for another 2 h. After cooling of the reaction it was poured on 20 g of ice and stirred for another 30 min. Then the organic layer was separated and the aqueous layer was extracted twice with ethyl acetate. The combined organic layers were dried over Na_2SO_4 and evaporated to dryness. The crude reaction mixture was purified by column chromatography (CH_2Cl_2 / Heptane = 2:1) to give the products **12** in the reported yields.

Note: After column chromatography around 10 % of the QMs **1** can be detected in the product mixture.

Step 3: For the synthesis of quinone methide **1b** the product **12b** of step 2 (5 mmol) was dissolved in 20 mL CH_2Cl_2 and triethylamine (5.5 mmol) was added. The reaction mixture was stirred at room temperature and after completion of the reaction (followed by TLC / 2-24 h), H_2O was added. The organic layer was washed with 1 N HCl followed by sat. $NaHCO_3$ and brine. After drying the organic layer over Na_2SO_4 it was evaporated to dryness and the quinone methide **1b** was isolated by column chromatography with gradients of heptanes and ethyl acetate (20:1 – 10:1 – 5:1 – 1:1).

---

[4] Y. Gong, K. Kato, and H. Kimoto, *Bull. Chem. Soc. Jpn.* **2001**, 74, 377-383.
[5] Y. Gong, K. Kato, *Synlett* **2002**, 3, 431-434.

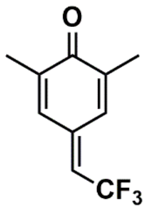

1b

<u>**1b**:</u> Prepared according to the general procedure 2 step 3 in 54%. The product occurs as a yellow oil (Precursors **11b** and **12b** are literature known compounds[4,5]).

R_f (CH_2Cl_2:heptanes = 2:1) = 0.23.

HRMS (ESI): m/z calculated for $C_{10}H_9F_3O$: 201.0533 $[M-H]^-$; found: 201.0535.

^{1}H-NMR (300 MHz, $CDCl_3$, 298 K): δ = 7.34 (s, 1H), 6.84 (s, 1H), 6.00 (q, J = 8.9 Hz, 1H), 2.05 (s, 3H), 2.02 (s, 3H) ppm; ^{19}F-NMR (282 MHz, $CDCl_3$, 298 K): δ = -55.40 (d, J = 8.9 Hz, 3F) ppm; ^{13}C-NMR (125 MHz, $CDCl_3$, 298 K): δ = 186.9, 140.0, 139.4, 137.8 (q, J = 5.5 Hz), 135.8, 128.7, 124.0 (q, J = 34.8 Hz), 122.7 (q, J = 271.7 Hz), 16.8, 16.2 ppm.

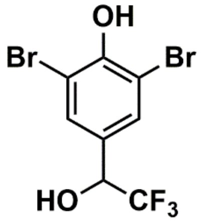

11c

<u>**11c**:</u> Compound was prepared according to the general procedure 2 step 1. The product occurs as yellow oil. Compound **11c** was used in the next step without further purification and the crude yield is 34%.

HRMS (ESI): m/z calculated for $C_8H_5Br_2F_3O_2$: 348.8681 $[M+H]^+$; found: 348.8678.

^{1}H-NMR (300 MHz, $CDCl_3$, 298 K): δ = 7.60 (s, 2H), 6.07 (s, 1H), 5.00 (q, J = 6.5 Hz, 1H) ppm; ^{19}F-NMR (282 MHz, $CDCl_3$, 298 K): δ = -73.36 (d, J = 6.5 Hz, 3F) ppm; ^{13}C-NMR (125 MHz, $CDCl_3$, 298 K): δ = 151.1, 132.5, 129.2, 128.4, 126.7, 125.4, 123.1 (q, J = 278.7 Hz), 110.2, 56.9 (q, J = 34.5 Hz) ppm.

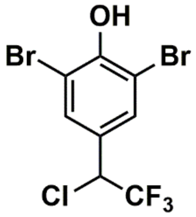

12c

<u>**12c**:</u> Compound was prepared according to the general procedure 2 step 2 in 63% yield. The product occurs as yellow oil.

R_f (CH_2Cl_2:heptanes = 2:1) = 0.56.

HRMS (ESI): m/z calculated for $C_8H_4Br_2ClF_3O$: 366.8342 $[M+H]^+$; found: 366.8348.

^{1}H-NMR (300 MHz, $CDCl_3$, 298 K): δ = 7.60 (s, 2H), 6.07 (s, 1H), 5.00 (q, J = 6.6 Hz, 1H) ppm; ^{19}F-NMR (282 MHz, $CDCl_3$, 298 K): δ = -73.45 (d, J = 6.6 Hz, 3F) ppm; ^{13}C-NMR (125 MHz, $CDCl_3$, 298 K): δ = 151.1, 132.5, 128.7, 123.2 (q, J = 278.8 Hz), 110.2, 50.0 (q, J = 34.4 Hz) ppm.

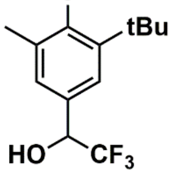

11d

<u>**11d**:</u> Compound was prepared according to the general procedure 2 step 1 in 66% crude yield. The product occurs as yellow oil. The crude product was directly used in the next step without further purification.

HRMS (ESI): m/z calculated for $C_{13}H_{17}F_3O_2$: 263.1253 $[M+H]^+$; found: 263.1252.

^{1}H-NMR (300 MHz, $CDCl_3$, 298 K): δ = 7.21 (s, 1H), 7.13 (s, 1H), 4.93-4.89 (m, 2H), 2.35 (d, J = 4.1 Hz, 1H), 2.27 (s, 3H), 1.42 (s, 9H) ppm; ^{19}F-NMR (282 MHz, $CDCl_3$, 298 K): δ = -78.30 (d, J = 6.9 Hz, 3F) ppm; ^{13}C-NMR (125 MHz, $CDCl_3$, 298 K): δ = 153.9, 136.1, 127.6, 125.4, 124.7, 124.6 (q, J = 283.5 Hz), 123.5, 73.1 (q, J = 31.8 Hz), 35.7, 29.8, 16.1 ppm.

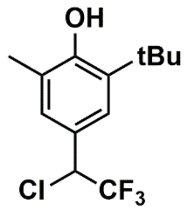

12d

12d: Compound was prepared according to the general procedure 2 step 2 in 95% (yellow oil).

R_f (CH_2Cl_2:heptanes = 2:1) = 0.42.

HRMS (ESI): m/z calculated for $C_{13}H_{16}ClF_3O$: 281.0915 $[M+H]^+$; found: 281.0915.

^{1}H-NMR (300 MHz, $CDCl_3$, 298 K): δ = 7.22 (s, 1H), 7.17 (s, 1H), 5.03 (q, J = 7.0 Hz, 1H), 2.27 (s, 3H), 1.42 (s, 9H) ppm; ^{19}F-NMR (282 MHz, $CDCl_3$, 298 K): δ = -73.21 (d, J = 7.0 Hz, 3F) ppm; ^{13}C-NMR (125 MHz, $CDCl_3$, 298 K): δ = 154.2, 138.0, 136.1, 129.2, 128.8, 128.4, 126.1, 125.4, 123.7, 123.7 (q, J = 279.1 Hz), 123.5, 59.2 (q, J = 34.1 Hz), 34.7, 29.7, 16.1 ppm.

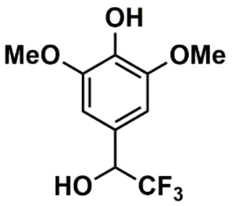

11e

11e: Prepared according to the general procedure 2 step 1 in 30% yield (colorless oil).

R_f (CH_2Cl_2:heptanes = 2:1) = 0.33.

HRMS (ESI): m/z calculated for $C_{11}H_{11}F_3O_4$: 253.0682 $[M+H]^+$; found: 253.0680.

^{1}H-NMR (300 MHz, $CDCl_3$, 298 K): δ = 6.70 (s, 2H), 5.60 (s, 1H), 4.98-4.90 (m, 1H), 3.91 (s, 6H), 2.54 (d, J = 4.2 Hz, 1H) ppm; ^{19}F-NMR (282 MHz, $CDCl_3$, 298 K): δ = -78.36 (d, J = 6.3 Hz, 3F) ppm; ^{13}C-NMR (125 MHz, $CDCl_3$, 298 K): δ = 147.2, 135.9, 127.9 (q, J = 280.2 Hz), 125.1, 125.5, 104.4, 73.0 (q, J = 32.8 Hz), 56.5 ppm.

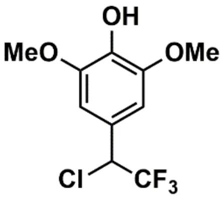

12e

12e: Prepared according to the general procedure 2 step 2 in 71% yield (pink oil).

R_f (CH_2Cl_2:heptanes = 2:1) = 0.33.

HRMS (ESI): m/z calculated for $C_{10}H_{10}ClF_3O_3$: 271.0343 $[M+H]^+$; found: 271.0339.

^{1}H-NMR (300 MHz, $CDCl_3$, 298 K): δ = 6.71 (s, 2H), 5.65 (s, 1H), 5.03 (q, J = 6.7 Hz, 1H), 3.92 (s, 6H) ppm; ^{19}F-NMR (282 MHz, $CDCl_3$, 298 K): δ = -73.20 (d, J = 6.7 Hz, 3F) ppm; ^{13}C-NMR (125 MHz, $CDCl_3$, 298 K): δ = 147.1, 136.4, 129.1, 128.4, 125.4, 123.8 (q, J = 282.8 Hz), 123.1, 105.8, 59.3 (q, J = 35.0 Hz), 56.6 ppm.

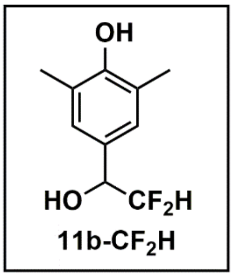

11b-CF_2H: Prepared according to the general procedure 2 step 1 in 70% yield (yellow oil). The product decomposes very quickly and have to be used directly for the next step.

R_f (heptanes:EtOAc = 1:1) = 0.62.

HRMS (ESI): m/z calculated for $C_{10}H_{12}F_2O_2$: 203.0878 $[M+H]^+$; found: 203.0877.

^{1}H-NMR (300 MHz, $CDCl_3$, 298 K): δ = 7.03 (s, 2H), 5.74 (td, J_1 = 56.2 Hz, J_2 = 4.8 Hz, 1H), 4.74-4.66 (m, 2H), 2.26 (s, 6H) ppm; ^{19}F-NMR (282 MHz, $CDCl_3$, 298 K): δ = -120.82 (dd, J_1 = 56.2 Hz, J_2 = 10.9 Hz, 1F), 120.91 (dd, J_1 = 56.2 Hz, J_2 = 10.9 Hz, 1F) ppm.

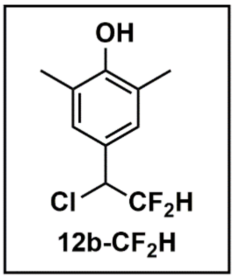

12b-CF_2H: Prepared according to the general procedure 2 step 2 in 30% yield (yellow oil).

R_f (CH_2Cl_2:heptanes = 2:1) = 0.42.

HRMS (ESI): m/z calculated for $C_{10}H_{11}ClF_2O$: 221.0539 $[M+H]^+$; found: 221.0539.

^{1}H-NMR (300 MHz, $CDCl_3$, 298 K): δ = 7.05 (s, 2H), 5.94 (td, J_1 = 56.1 Hz, J_2 = 4.9 Hz, 1H), 4.83 (td, J_1 = 10.9 Hz, J_2 = 4.9 Hz, 1H), 4.76 (s, 1H), 2.26 (s, 6H) ppm; ^{19}F-NMR (282 MHz, $CDCl_3$, 298 K): δ = -120.83 (dd, J_1 = 56.1 Hz, J_2 = 10.9 Hz, 1F), -120.88 (dd, J_1 = 56.1 Hz, J_2 = 10.9 Hz, 1F) ppm; ^{13}C-NMR (125 MHz, $CDCl_3$, 298 K): δ = 153.4, 128.9, 125.3, 123.6, 114.5 (t, J = 249.3 Hz), 60.3 (t, J = 25.5 Hz), 16.0 ppm.

2.2. Kinetics

UV-Spectrum for 1a and Its Molar Absorption Coefficient at λ_{max}

A solution of quinone methide **1a** in DMSO (3 mg in 1 mL solvent) was added stepwise to 24 mL of DMSO, by means of a microliter syringe. The molar absorption coefficient ε was determined by linear regression of a correlation of absorbance (at the absorption maximum λ_{max} = 292 nm) versus the concentration.

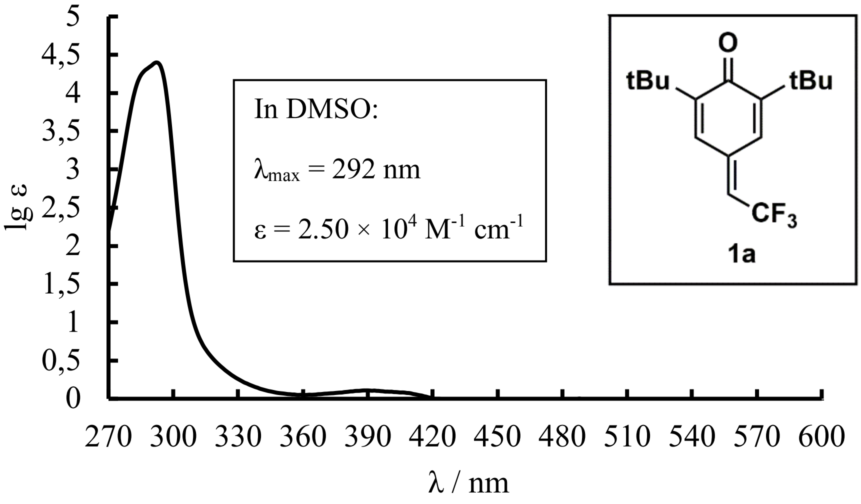

Kinetic Measurements

<u>Kinetics of the reaction of **1a** with **5a (generated from diethyl malonate 5a-H)**</u>

[**1a**]$_0$, mol L^{-1}	[**5a**-H]$_0$, mol L^{-1}	[KOtBu]$_0$, mol L^{-1}	[18-c-6]$_0$, mol L^{-1}	[**5a**]$_0$, mol L^{-1}	k_{obs}, s^{-1}
2.52×10^{-5}	4.00×10^{-4}	2.00×10^{-4}		2.00×10^{-4}	1.08×10^{2}
2.52×10^{-5}	5.00×10^{-4}	2.50×10^{-4}	3.00×10^{-4}	2.50×10^{-4}	1.38×10^{2}
2.52×10^{-5}	6.00×10^{-4}	3.00×10^{-4}		3.00×10^{-4}	1.65×10^{2}
2.52×10^{-5}	7.00×10^{-4}	3.50×10^{-4}	5.00×10^{-4}	3.50×10^{-4}	1.91×10^{2}
2.52×10^{-5}	8.00×10^{-4}	4.00×10^{-4}		4.00×10^{-4}	2.16×10^{2}

$k_2 = 5.38 \times 10^5$ M^{-1} s^{-1}

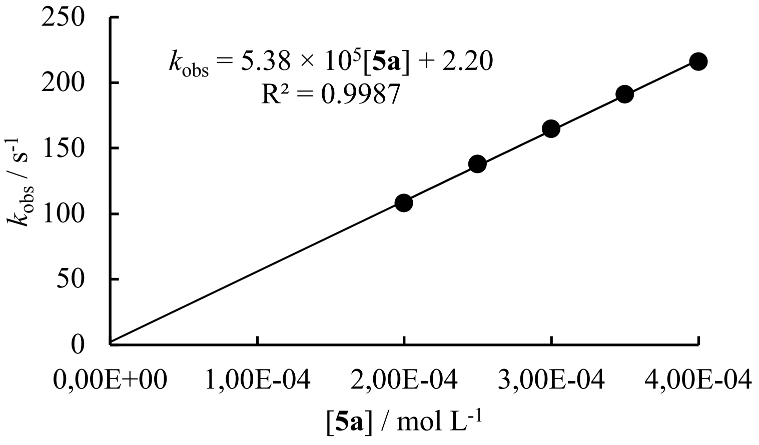

Kinetics of the reaction of **1a** with **5b (generated from ethyl cyanoacetate 5b-H)**

[**1a**]$_0$, mol L^{-1}	[**5b**-H]$_0$, mol L^{-1}	[KOtBu]$_0$, mol L^{-1}	[18-c-6]$_0$, mol L^{-1}	[**5b**]$_0$, mol L^{-1}	k_{obs}, s^{-1}
2.52×10^{-5}	4.00×10^{-4}	2.00×10^{-4}		2.00×10^{-4}	5.58×10^{1}
2.52×10^{-5}	5.00×10^{-4}	2.50×10^{-4}	3.00×10^{-4}	2.50×10^{-4}	7.04×10^{1}
2.52×10^{-5}	6.00×10^{-4}	3.00×10^{-4}		3.00×10^{-4}	8.22×10^{1}
2.52×10^{-5}	7.00×10^{-4}	3.50×10^{-4}	5.00×10^{-4}	3.50×10^{-4}	9.23×10^{1}
2.52×10^{-5}	8.00×10^{-4}	4.00×10^{-4}		4.00×10^{-4}	1.13×10^{2}

$k_2 = 2.73 \times 10^5$ M^{-1} s^{-1}

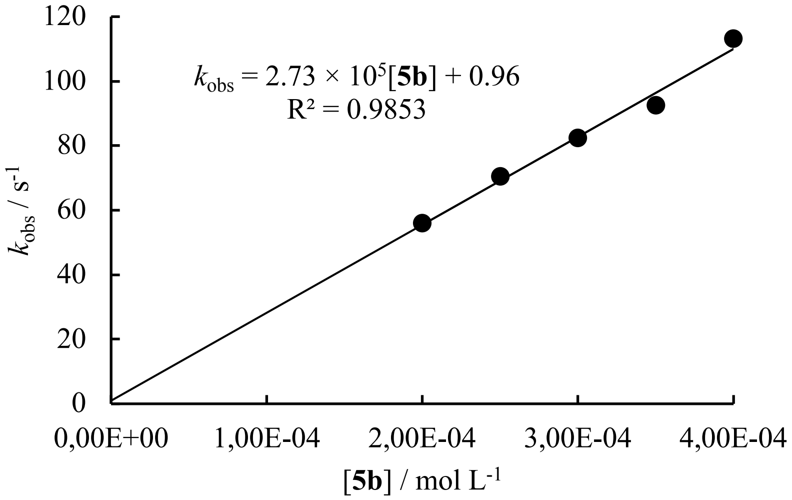

Kinetics of the reaction of **1a** with **5c (generated from malononitrile 5c-H)**

[**1a**]$_0$, mol L^{-1}	[**5c**-H]$_0$, mol L^{-1}	[KOtBu]$_0$, mol L^{-1}	[18-c-6]$_0$, mol L^{-1}	[**5c**]$_0$, mol L^{-1}	k_{obs}, s^{-1}
2.52×10^{-5}	4.00×10^{-4}	2.00×10^{-4}		2.00×10^{-4}	2.02×10^{1}
2.52×10^{-5}	6.00×10^{-4}	3.00×10^{-4}	3.00×10^{-4}	3.00×10^{-4}	3.14×10^{1}
2.52×10^{-5}	8.00×10^{-4}	4.00×10^{-4}		4.00×10^{-4}	4.21×10^{1}
2.52×10^{-5}	1.00×10^{-3}	5.00×10^{-4}	5.00×10^{-4}	5.00×10^{-4}	5.10×10^{1}

$k_2 = 1.03 \times 10^5$ M^{-1} s^{-1}

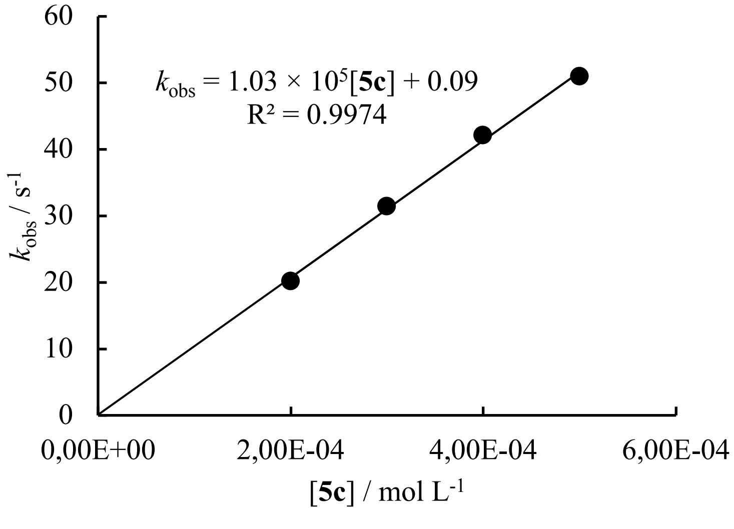

Kinetics of the reaction of **1a** with **5d (generated from the phosphine oxide 5d-H)**

[**1a**]$_0$, mol L^{-1}	[**5d**-H]$_0$, mol L^{-1}	[KOtBu]$_0$, mol L^{-1}	[18-c-6]$_0$, mol L^{-1}	[**5d**]$_0$, mol L^{-1}	k_{obs}, s^{-1}
2.52×10^{-5}	4.00×10^{-4}	2.00×10^{-4}		2.00×10^{-4}	1.59×10^{1}
2.52×10^{-5}	5.00×10^{-4}	2.50×10^{-4}	3.00×10^{-4}	2.50×10^{-4}	1.99×10^{1}
2.52×10^{-5}	6.00×10^{-4}	3.00×10^{-4}		3.00×10^{-4}	2.35×10^{1}
2.52×10^{-5}	7.00×10^{-4}	3.50×10^{-4}	5.00×10^{-4}	3.50×10^{-4}	2.79×10^{1}

$k_2 = 7.92 \times 10^{4}$ M^{-1} s^{-1}

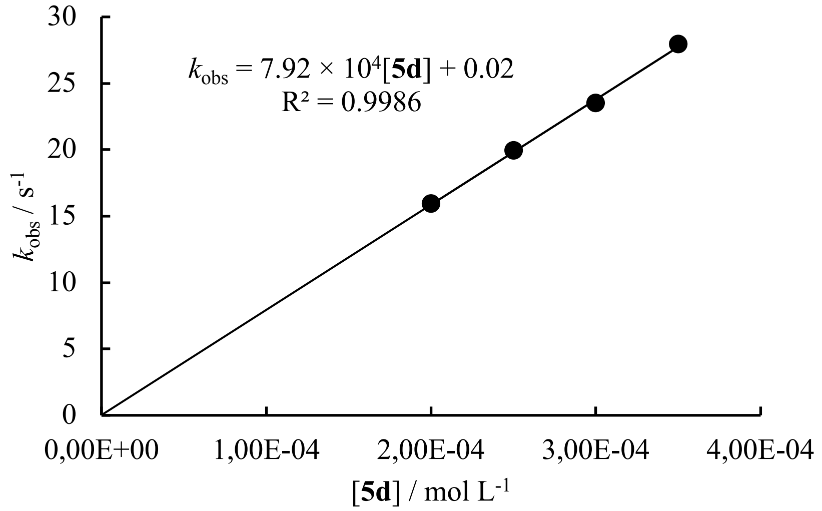

Determination of the Electrophilicity Parameter *E* for the Quinone Methide 1a

Nucleophile	*N*	s_N	k_2^{exp} ($M^{-1}s^{-1}$)	k_2^{Eq1} [a] ($M^{-1}s^{-1}$)	k_2^{exp}/k_2^{Eq1}
**5a**	20.22	0.65	5.38×10^5	3.56×10^5	1.5
**5b**	19.62	0.67	2.73×10^5	2.09×10^5	1.3
**5c**	19.36	0.67	1.03×10^5	1.40×10^5	0.74
**5d**	18.69	0.72	7.92×10^4	1.11×10^5	0.71

[a] Second-order rate constant k_2^{Eq1} calculated by applying Equation 1 (main text), the nucleophile-specific reactivity parameters *N* and s_N of **5a–d** (from ref [2]) and the *E* parameter of **1a**.

<u>*E* (**1a**) = –11.68</u>

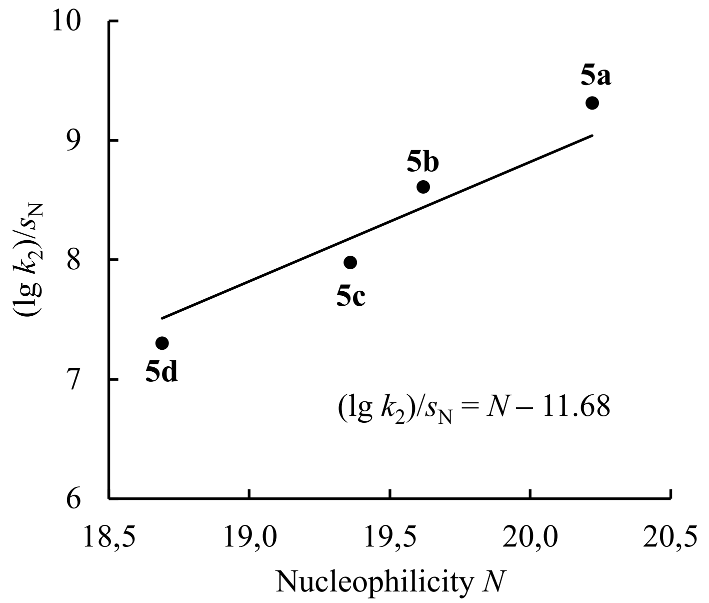

2.3 Products 6a-d of the Kinetic Studies

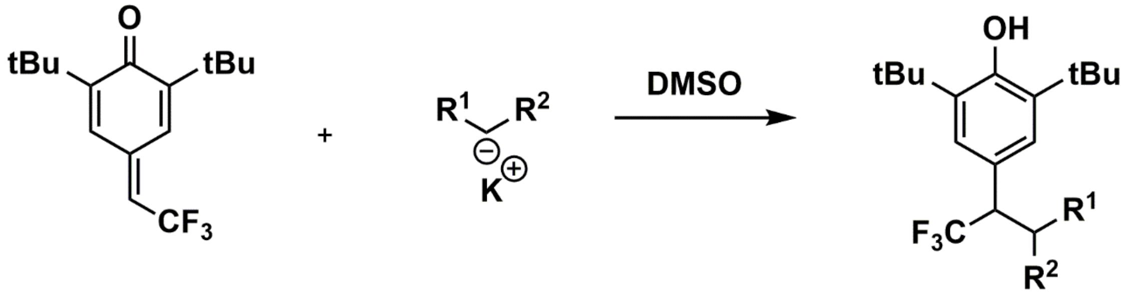

1a **5a-d** **6a-d**

5a/6a: R[1] = COOEt, R[2] = COOEt
5b/6b: R[1] = CN, R[2] = COOEt
5c/6c: R[1] = CN, R[2] = CN
5d/6d: R[1] = CN, R[2] = PO(Ph)2

General procedure 3 (Synthesis of compounds 6 in DMSO with KOtBu): 2,6-di-*tert*-Butyl-4-(2,2,2-trifluoroethylidene)cyclohexa-2,5-dien-1-on **1a** (0.1 mmol) and KOtBu (0.2 mmol) were dissolved in DMSO (2 ml) in a flame dried Schlenk flask. Then the corresponding reaction partner **5-H** (0.2 mmol) was added. The reaction mixture was stirred at room temperature. After 2 h the mixture was diluted with H_2O, extracted three times with EtOAc and with brine. The organic layer was dried with Na_2SO_4, filtered and concentrated in vacuo. The crude product was purified by column chromatography on silica gel (heptanes/EtOAc = 20:1).

General procedure 4 (Synthesis of compounds 6 under phase-transfer conditions): The quinone methide **1a** (0.2 mmol), the corresponding nucleophiles **5-H** (0.24 mmol) and triethylbenzylammonium chloride (TEBAC, 10 mol%) were dissolved in 4 mL CH_2Cl_2 and Cs_2CO_3 (0.24 mmol) was added. After stirring at room temperature for 24 h, the reaction mixture was filtered over a pad of Na_2SO_4 and evaporated to dryness. The products were purified by column chromatography (heptanes:EtOAc = 20:1).

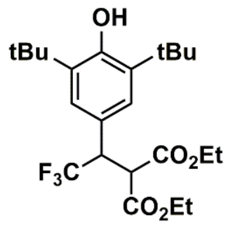

6a

<u>**6a**:</u> Compound was prepared according to the general procedure 3 in 49% isolated yield and according to procedure 4 in 62% (yellow oil).

R_f (CH_2Cl_2:heptanes = 2:1) = 0.73.

HRMS (ESI): m/z calculated for $C_{23}H_{33}F_3O_5$: 447.2350 $[M+H]^+$; found: 447.2353.

^{1}H-NMR (300 MHz, $CDCl_3$, 298 K): δ = 7.06 (s, 2H), 5.23 (s, 1H), 4.32-4.21 (m, 2H), 4.20-4.10 (m, 1H), 4.09-4.05 (m, 1H), 3.94-3.76 (m, 2H), 1.41 (s, 18H), 1.31 (t, J = 7.1 Hz, 3H), 0.84 (t, J = 7.1 Hz, 3H) ppm; ^{19}F-NMR (282 MHz, $CDCl_3$, 298 K): δ = -66.61 (d, J = 8.3 Hz, 3F)

ppm; ^{13}C-NMR (125 MHz, $CDCl_3$, 298 K): δ = 167.1, 165.9, 154.2, 136.0, 136.4, 136.3 (q, J = 285.0 Hz), 122.4, 62.4, 61.8, 53.2, 49.3 (q, J = 27.0 Hz), 34.4, 30.3, 14.1, 13.6 ppm.

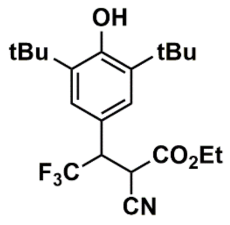

6b

<u>**6b**:</u> Prepared according to the general procedure 3 in 43% yield and with a d.r. of 2:1 and according to procedure 4 in 51% (yellow oil).

R_f (CH_2Cl_2:heptanes = 2:1) = 0.65.

HRMS (ESI): m/z calculated for $C_{21}H_{28}F_3NO_3$: 400.2094 $[M+H]^+$; found: 400.2095.

^{1}H-NMR (300 MHz, $CDCl_3$, 298 K): δ = 7.23 (s, 2H), 5.35 (s, 1H), 4.36-4.13 (m, 3H), 4.08-3.97 (m, 1H), 1.43 (s, 18H), 1.18-1.14 (m, 3H) ppm; ^{19}F-NMR (282 MHz, $CDCl_3$, 298 K): δ = -67.95 (d, J = 9.0 Hz, 3F) ppm; ^{13}C-NMR (125 MHz, $CDCl_3$, 298 K): δ = 163.8, 155.0, 154.9, 136.5, 126.3, 125.8, 120.0, 119.8 (q, J = 286.1 Hz), 114.1, 63.7, 49.3 (q, 28.6 Hz), 39.3, 30.3, 13.9 ppm.

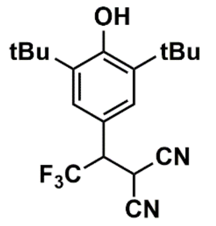

6c

<u>**6c**:</u> Prepared according to the general procedure 3 in 37% yield and according to procedure 4 in 48% (yellow oil).

R_f (CH_2Cl_2:heptanes = 2:1) = 0.69.

HRMS (ESI): m/z calculated for $C_{19}H_{23}F_3N_2O$: 353.1835 $[M+H]^+$; found: 353.1829.

^{1}H-NMR (300 MHz, $CDCl_3$, 298 K): δ = 7.23 (s, 2H), 5.46 (s, 1H), 4.33 (d, J = 5.7 Hz, 1H), 3.89-3.79 (m, 1H), 1.45 (s, 18H) ppm; ^{19}F-NMR (282 MHz, $CDCl_3$, 298 K): δ = -66.92 (d, J = 8.5 Hz, 3F) ppm; ^{13}C-NMR (125 MHz, $CDCl_3$, 298 K): δ = 155.7, 137.2, 125.9, 124.8 (q, J = 273.3Hz), 118.7, 110.5, 110.2, 50.5 (q, J = 29.1 Hz), 34.6, 30.2, 25.5 ppm.

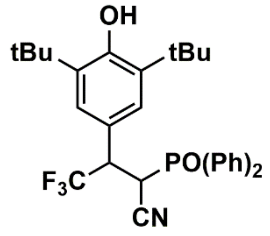

6d

<u>**6d**:</u> Prepared according to the general procedure 3 in 55% yield and with a d.r. of 12:1 and according to procedure 4 in 57% and with a d.r. of 12.5:1 (yellow oil).

R_f (CH_2Cl_2:heptanes = 2:1) = 0.72.

HRMS (ESI): m/z calculated for $C_{30}H_{33}F_3NO_2P$: 528.2274 $[M+H]^+$; found: 528.2268.

^{1}H-NMR (300 MHz, $CDCl_3$, 298 K): δ = 7.85-7.81 (m, 2H), 7.64-7.59 (m, 1H), 7.56-7.50 (m, 2H), 7.47-7.40 (m, 2H), 7.37-7.31 (m, 1H), 7.25 (s, 1H), 7.20-7.14 (m, 2H), 5.19, (s, 1H), 4.40-4.27 (m, 1H), 4.08-3.99 (m, 1H), 1.33 (s, 18H) ppm; ^{19}F-NMR (282 MHz, $CDCl_3$, 298 K): δ = -68.60 (d, J = 9.3 Hz, 3F) ppm; ^{13}C-NMR (125 MHz, $CDCl_3$, 298 K): δ = 154.8, 136.0, 133.3 (d, J = 2.7 Hz), 132.4 (d, J = 2.8 Hz), 131.9 (d, J = 9.47 Hz), 130.9 (d, J = 10.1 Hz), 129.2 (d, J = 12.9 Hz), 128.9 (q, J = 273.4 Hz), 127.9 (d, J = 12.6 Hz), 127.2 (2C), 119.9, 116.1 (d, J = 2.9 Hz), 45.9 (q, J = 28.7 Hz), 34.4, 33.7 (d, J = 62.2 Hz), 30.2 ppm.

2.4 Racemic Reactions with C- and Heteroatom Nucleophiles

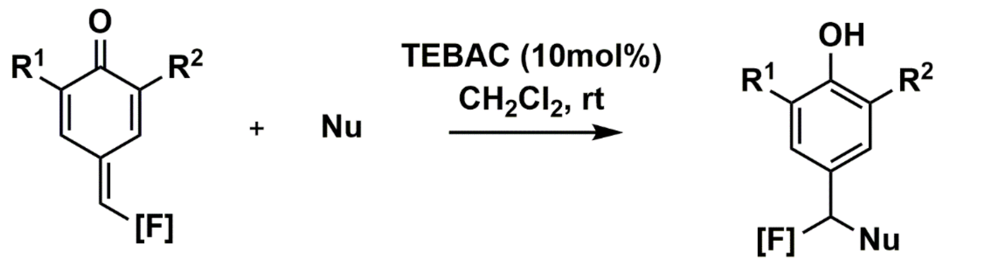

1a, 12b-f **6a-h, 14a-k, 15a-d, 16**

General procedure 5: The quinone methides **1a** or **1b** (0.2 mmol) or the QM-precursors **12b-e** (0.2 mmol), the corresponding nucleophiles (0.24 mmol) and triethylbenzylammonium chloride (TEBAC, 10 mol%) were dissolved in 4 mL CH_2Cl_2 and Cs_2CO_3 (0.24 mmol) was added. After stirring at room temperature for 24 h, the reaction mixture was filtered over a pad of Na_2SO_4 and evaporated to dryness. The products were purified by column chromatography (Heptanes:EtOAc = 20:1).

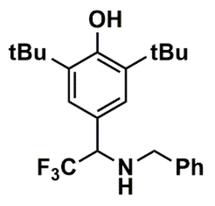

14a

14a: Prepared according to the general procedure 5 in an isolated yield of 69%. The product occurs as light brown solid with a melting range of 64.9 – 66.3°C.

R_f (CH_2Cl_2:heptanes = 2:1) = 0.71.

HRMS (ESI): m/z calculated for $C_{23}H_{30}F_3NO$: 394.2352 $[M+H]^+$; found: 394.2352.

^{1}H-NMR (300 MHz, $CDCl_3$, 298 K): δ = 7.33-7.26 (m, 5H), 7.15 (s, 2H), 5.29 (s, 1H), 4.02 (q, J = 7.5 Hz, 1H), 3.86-3.82 (m, 1H), 3.71-3.64 (m, 1H), 1.45 (s, 18H) ppm; ^{19}F-NMR (282 MHz, $CDCl_3$, 298 K): δ = -73.85 (d, J = 7.5 Hz, 3F) ppm; ^{13}C-NMR (125 MHz, $CDCl_3$, 298 K): δ = 154.4, 139.5, 136.1, 128.6, 128.4, 127.4, 126.0 (q, J = 283.2 Hz), 124.0, 63.5 (q, J = 28.6 Hz), 51.4, 34.5, 30.4 ppm.

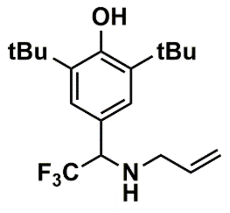

14b

14b: Prepared according to the general procedure 5 in 76% yield (yellow oil).

R_f (CH_2Cl_2:heptanes = 3:1) = 0.54.

HRMS (ESI): m/z calculated for $C_{19}H_{28}F_3NO$: 344.2196 $[M+H]^+$; found: 344.2199.

^{1}H-NMR (300 MHz, $CDCl_3$, 298 K): δ = 7.14 (s, 2H), 5.93-5.78 (m, 1H), 5.27 (s, 1H), 5.19-5.122 (m, 2H), 4.06 (q, J = 7.1 Hz, 1H), 3.22-3.16 (m, 2H), 1.44 (s, 18H) ppm; ^{19}F-NMR (282 MHz, $CDCl_3$, 298 K): δ = -73.85 (d, J = 7.1 Hz, 3F) ppm; ^{13}C-NMR (125 MHz, $CDCl_3$, 298 K): δ = 154.4, 136.1, 136.0, 125.8 (q, J = 282.7 Hz), 125.2, 117.1, 63.5 (q, J = 29.3 Hz), 50.2, 34.5, 30.4 ppm.

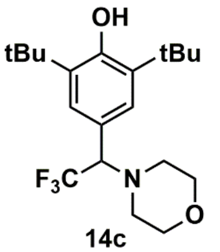

14c

14c: Prepared according to the general procedure 5 in 80% yield (yellow oil).

R_f (CH_2Cl_2:heptanes = 3:1) = 0.42.

HRMS (ESI): m/z calculated for $C_{20}H_{30}F_3NO_2$: 374.2301 $[M+H]^+$; found: 374.2302.

^{1}H-NMR (300 MHz, $CDCl_3$, 298 K): δ = 7.11 (s, 2H), 5.27 (s, 1H), 3.88 (q, J = 9.5 Hz, 1H), 3.71-3.68 (m, 4H), 2.66-2.58 (m, 2H), 2.54-2.48 (m, 2H), 1.44 (s, 18H) ppm; ^{19}F-NMR (282 MHz, $CDCl_3$, 298 K): δ = -66.91 (d, J = 9.5 Hz, 3F) ppm; ^{13}C-NMR (125 MHz, $CDCl_3$, 298 K): δ = 154.1, 135.8, 126.1, 126.0 (q, J = 286.1 Hz), 122.3, 71.1 (q, J = 26.9 Hz), 67.4, 34.4, 30.4 ppm.

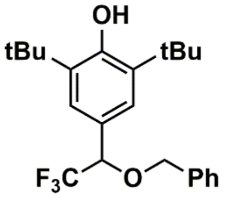

15a

15a: Prepared according to the general procedure 5 in 76% yield (yellow oil).

R_f (CH_2Cl_2:heptanes = 2:1) = 0.56.

HRMS (ESI): m/z calculated for $C_{23}H_{29}F_3O_2$: 395.2192 $[M+H]^+$; found: 395.2190.

^{1}H-NMR (300 MHz, $CDCl_3$, 298 K): δ = 7.36-7.27 (m, 5H), 7.16 (s, 2H), 5.28 (s, 1H), 4.04 (q, J = 7.6 Hz, 1H), 3.85-3.80 (m, 1H), 3.70-3.66 (m, 1H), 1.44 (s, 18H) ppm; ^{19}F-NMR (282 MHz, $CDCl_3$, 298 K): δ = -73.87 (d, J = 7.6 Hz, 3F) ppm; ^{13}C-NMR (125 MHz, $CDCl_3$, 298 K): δ = 154.4, 139.4, 136.1, 128.6, 128.4, 127.4, 126.2 (q, J = 281.2 Hz), 124.0, 63.5 (q, J = 28.1 Hz), 51.4, 34.5, 30.4 ppm.

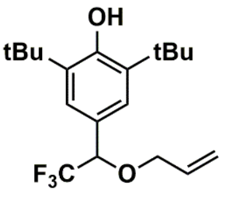

15b

15b: Prepared according to the general procedure 5 in 70% yield. The product occurs as yellow solid with a melting range of 66.8 – 67.3°C.

R_f (CH_2Cl_2:heptanes = 2:1) = 0.74.

HRMS (ESI): m/z calculated for $C_{19}H_{27}F_3O_2$: 343.1890 $[M-H]^-$; found: 343.1885.

^{1}H-NMR (300 MHz, $CDCl_3$, 298 K): δ = 7.19 (s, 2H), 5.96-5.83 (m, 1H), 5.32-5.22 (m, 3H), 4.58 (q, J = 6.8 Hz, 1H), 4.14-4.08 (m, 1H), 3.99-3.92 (m, 1H), 1.44 (s, 18H) ppm; ^{19}F-NMR (282 MHz, $CDCl_3$, 298 K): δ = -76.40 (d, J = 6.8 Hz, 3F) ppm; ^{13}C-NMR (125 MHz, $CDCl_3$, 298 K): δ = 154.8, 136.1, 133.8, 125.1, 124.1 (q, J = 283.1 Hz), 123.2, 118.2, 78.9 (q, J = 30.2 Hz), 70.5, 34.5, 30.4 ppm.

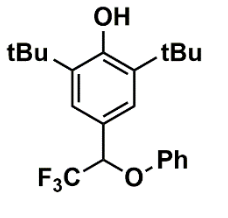

15c

15c: Prepared according to the general procedure 5 in 60% yield. The product occurs as white solid with a melting range of 80.4 – 81.6°C.

R_f (CH_2Cl_2:heptanes = 2:1) = 0.70.

HRMS (ESI): m/z calculated for $C_{22}H_{27}F_3O_2$: 379.1890 $[M-H]^-$; found: 379.1885.

^{1}H-NMR (300 MHz, $CDCl_3$, 298 K): δ = 7.26-7.20 (m, 4H), 7.00-6.90 (m, 3H), 5.32-5.26 (m 2H), 1.41 (s, 18H) ppm; ^{19}F-NMR (282 MHz, $CDCl_3$, 298 K): δ = -76.70 (d, J = 6.5 Hz, 3F) ppm; ^{13}C-NMR (125 MHz, $CDCl_3$, 298 K): δ = 157.5, 154.9, 136.2, 129.6, 124.7, 123.8 (q, J = 278.2 Hz), 122.8, 122.4, 122.0, 116.5, 79.0 (q, J = 32.8 Hz), 34.4, 30.4 ppm.

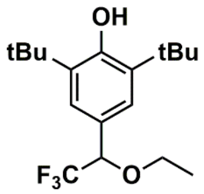

15d

15d: Prepared according to the general procedure 5 in 69% yield (yellow oil).

R_f (CH_2Cl_2:heptanes = 2:1) = 0.73.

HRMS (ESI): m/z calculated for $C_{18}H_{27}F_3O_2$: 331.1890 $[M-H]^-$; found: 331.1886.

^{1}H-NMR (300 MHz, $CDCl_3$, 298 K): δ = 7.20 (s, 2H), 5.31 (s, 1H), 4.52 (q, J = 7.0 Hz, 1H), 3.57 (q, J = 6.8 Hz, 2H), 1.44 (s, 18H), 1.24 (t, J = 6.8 Hz, 3H) ppm; ^{19}F-NMR (282 MHz, $CDCl_3$, 298 K): δ = -76.67 (d, J = 7.0 Hz, 3F) ppm; ^{13}C-NMR (125 MHz, $CDCl_3$, 298 K): δ = 154.7, 136.0, 124.9, 124.2 (q, J = 284.1 Hz), 123.7, 80.0 (q, J = 32.2 Hz), 66.0, 34.5, 30.4, 15.3 ppm.

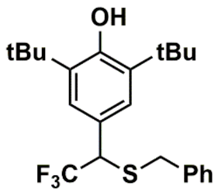

16

16: Prepared according to the general procedure 5 in 78% yield (yellow oil).

R_f (CH_2Cl_2:heptanes = 2:1) = 0.68.

HRMS (ESI): m/z calculated for $C_{13}H_{29}F_3OS$: 409.1818 $[M-H]^-$; found: 409.1812.

^{1}H-NMR (300 MHz, $CDCl_3$, 298 K): δ = 7.35-7.27 (m, 5H), 7.01 (s, 2H), 5.28 (s, 1H), 4.00 (q, J = 8.6 Hz, 1H), 3.89-3.85 (m, 1H), 3.73-3.69 (m, 1H), 1.40 (s, 18H) ppm; ^{19}F-NMR (282 MHz, $CDCl_3$, 298 K): δ = -67.73 (d, J = 8.6 Hz, 3F) ppm; ^{13}C-NMR (125 MHz, $CDCl_3$, 298 K): δ = 154.2, 136.8, 136.2, 129.3, 128.7, 126.8 (q, J = 278.8 Hz), 125.8, 124.0, 51.5 (q, J = 28.9 Hz), 37.0, 34.5, 30.4 ppm.

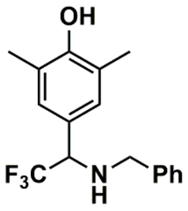

14d

<u>**14d**:</u> Prepared according to the general procedure 5 starting from **12b** in 76% yield (yellow oil).

R_f (CH_2Cl_2:heptanes = 2:1) = 0.43.

HRMS (ESI): m/z calculated for $C_{17}H_{18}F_3NO$: 310.1413 $[M+H]^+$; found: 310.1409.

^{1}H-NMR (300 MHz, $CDCl_3$, 298 K): δ = 7.35-7.24 (m, 5H), 7.00 (s, 2H), 4.67 (s, 1H), 3.99, (q, J = Hz, 1H), 3.84-3.79 (m, 1H), 3.68-3.63 (m, 1H), 2.26 (s, 6H) ppm; ^{19}F-NMR (282 MHz, $CDCl_3$, 298 K): δ = -74.09 (d, J = 7.5 Hz, 3F) ppm; ^{13}C-NMR (125 MHz, $CDCl_3$, 298 K): δ = 152.9, 139.3, 128.9, 128.7, 128.3, 127.4, 123.4, 122.7 (q, J = 273.3 Hz), 63.0 (q, J = 29.3 Hz), 51.2, 16.1 ppm

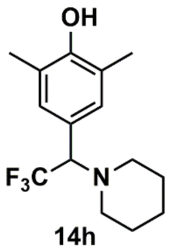

14h

<u>**14h**:</u> Prepared according to the general procedure 5 starting from **12b** in 52% yield (yellow oil).

R_f (CH_2Cl_2:heptanes = 2:1) = 0.55.

HRMS (ESI): m/z calculated for $C_{15}H_{20}F_3NO$: 288.1570 $[M+H]^+$; found: 288.1567.

^{1}H-NMR (300 MHz, $CDCl_3$, 298 K): δ = 6.96 (s, 2H), 4.65 (s, 1H), 3.88 (q, J = 9.1 Hz, 1H), 2.64-2.56 (m, 2H), 2.48-2.42 (m, 2H), 2.25 (s, 6H), 1.59-1.52 (m, 4H), 1.40-1.34 (m, 2H) ppm; ^{19}F-NMR (282 MHz, $CDCl_3$, 298 K): δ = -66.88 (d, J = 9.1 Hz, 3F) ppm; ^{13}C-NMR (125 MHz, $CDCl_3$, 298 K): δ = 152.3, 129.8, 126.2 (q, J = 286.0 Hz), 124.1, 122.8, 70.8 (q, J = 27.5 Hz), 52.1, 26.5, 24.3, 16.2 ppm.

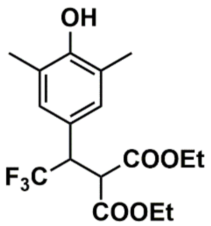

6e

<u>**6e**:</u> Prepared according to the general procedure 5 starting from **12b** in 52% yield (colourless oil).

R_f (CH_2Cl_2:heptanes = 2:1) = 0.43.

HRMS (ESI): m/z calculated for $C_{17}H_{21}F_3O_5$: 363.1414 $[M+H]^+$; found: 363.1414.

^{1}H-NMR (300 MHz, $CDCl_3$, 298 K): δ = 6.92 (s, 2H), 4.79 (s, 1H), 4.31-4.19 (m, 2H), 4.11-4.02 (m, 2H), 3.91 (q, J = 8.7 Hz, 2H), 2.21 (s, 6H), 1.30 (t, J = 7.7 Hz, 3H), 0.96 (t, J = 7.7 Hz, 3H) ppm; ^{19}F-NMR (282 MHz, $CDCl_3$, 298 K): δ = -68.42 (d, J = 8.7 Hz, 3F) ppm; ^{13}C-NMR (125 MHz, $CDCl_3$, 298 K): δ = 167.0, 165.9, 152.7, 129.8, 125.8 (q, J = 280.9 Hz) 123.3, 123.2, 62.5, 61.9, 53.0, 48.7 (q, J = 28.5 Hz), 18.0, 14.0, 13.7 ppm.

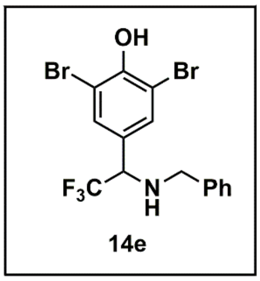

14e: Prepared according to the general procedure 5 starting from **12c** in 78% yield (yellow oil).

R_f (CH_2Cl_2:heptanes = 2:1) = 0.68.

HRMS (ESI): m/z calculated for $C_{15}H_{12}Br_2F_3NO$: 437.9311 $[M+H]^+$; found: 437.9310.

^{1}H-NMR (300 MHz, $CDCl_3$, 298 K): δ = 7.51 (s, 2H), 7.38-7.32 (m, 2H), 7.31-7.29 (m, 1H), 7.27-7.24 (m, 2H), 5.98 (s, 1H), 4.04 (q, J = 7.1 Hz, 1H), 3.84-3.80 (m, 1H), 3.65-5.60 (m, 1H) ppm; ^{19}F-NMR (282 MHz, $CDCl_3$, 298 K): δ = -74.16 (d, J = 7.1 Hz, 3F) ppm; ^{13}C-NMR (125 MHz, $CDCl_3$, 298 K): δ = 150.2, 138.6, 132.4, 128.8, 128.2, 127.7, 124.9 (q, J = 281.6 Hz), 110.24, 62.17 (q, J = 29.4 Hz), 61.0 ppm.

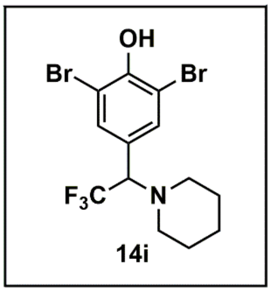

14i: Prepared according to the general procedure 5 starting from **12c** in 87% yield (yellow oil).

R_f (CH_2Cl_2:heptanes = 2:1) = 0.87.

HRMS (ESI): m/z calculated for $C_{13}H_{14}Br_2F_3NO$: 415.9467 $[M+H]^+$; found: 415.9467.

^{1}H-NMR (300 MHz, $CDCl_3$, 298 K): δ = 7.49 (s, 2H), 5.92 (s, 1H), 3.94 (q, J = 8.7 Hz, 1H), 2.62-2.48 (m, 4H), 1.60-1.52 (m, 4H), 1.44-1.40 (m, 2H) ppm; ^{19}F-NMR (282 MHz, $CDCl_3$, 298 K): δ = -66.20 (d, J = 8.7 Hz, 3F) ppm; ^{13}C-NMR (125 MHz, $CDCl_3$, 298 K): δ = 149.5, 132.7, 127.9, 126.0 (q, J = 287.1 Hz), 109.8, 69.6 (q, J = 27.7 Hz), 51.8, 26.5, 24.2 ppm.

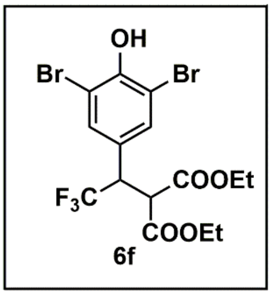

6f: Prepared according to the general procedure 5 starting from **12c** in 50% yield (yellow oil).

R_f (CH_2Cl_2:heptanes = 2:1) = 0.61.

HRMS (ESI): m/z calculated for $C_{15}H_{15}Br_2F_3O_5$: 490.9311 $[M+H]^+$; found: 490.9309.

^{1}H-NMR (700 MHz, $CDCl_3$, 298 K): δ = 7.43 (s, 2H), 5.95 (s, 1H), 4.32-4.21 (m, 2H), 4.19-4.10 (m, 1H), 4.02-3.93 (m, 3H), 1.30 (t, J = 7.1 Hz, 3H), 1.05 (t, J = 7.1 Hz, 3H) ppm; ^{19}F-NMR (282 MHz, $CDCl_3$, 298 K): δ = -68.26 (d, J = 8.3 Hz, 3F) ppm; ^{13}C-NMR (125 MHz, $CDCl_3$, 298 K): δ = 166.3, 165.5, 150.1, 134.5, 133.2, 126.6, 125.8 (q, J = 283.3 Hz), 110.0, 62.8, 62.4, 52.5, 48.1 (q, J = 28.2 Hz), 14.0, 13.9 ppm.

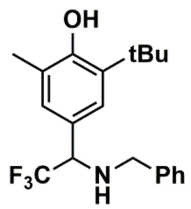

14f

14f: Prepared according to the general procedure 5 starting from **12d** in 84% yield (colorless oil).

R_f (CH_2Cl_2:heptanes = 2:1) = 0.68.

HRMS (ESI): m/z calculated for $C_{16}H_{21}F_3O$: 352.1883 $[M+H]^+$; found: 352.1882.

^{1}H-NMR (300 MHz, $CDCl_3$, 298 K): δ = 7.34-7.26 (m, 5H), 7.10 (s, 1H), 7.06 (s, 1H), 4.85 (s, 1H), 4.02 (q, J = 7.6 Hz, 1H), 3.85-3.80 (m, 1H), 3.69-3.65 (m, 1H), 2.27 (s, 3H), 1.42 (s, 9H) ppm; ^{19}F-NMR (282 MHz, $CDCl_3$, 298 K): δ = -73.08 (d, J = 7.6 Hz, 3F) ppm; ^{13}C-NMR (125 MHz, $CDCl_3$, 298 K): δ = 153.3, 139.4, 135.9, 128.6, 128.4, 128.4, 127.4, 125.9, 125.8 (q, J = 281.0 Hz), 125.4, 123.4, 63.3 (q, J = 27.8 Hz), 51.3, 34.7, 29.8, 29.2, 16.2 ppm.

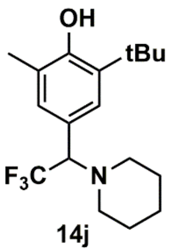

14j

14j: Prepared according to the general procedure 5 starting from **12d** in 88% yield (colorless oil).

R_f (CH_2Cl_2:heptanes = 2:1) = 0.73.

HRMS (ESI): m/z calculated for $C_{16}H_{21}F_3O$: 330.2039 $[M+H]^+$; found: 330.2041.

^{1}H-NMR (700 MHz, $CDCl_3$, 298 K): δ 7.11 (s, 1H), 7.00 (s, 1H), 4.78 (s, 1H), 3.93 (q, J = 9.1 Hz, 1H), 2.63-2.60 (m, 2H), 2.50-2.47 (m, 2H), 2.26 (s, 3H), 1.59-1.54 (m, 4H), 1.43 (s, 9H), 1.40-1.38 (m, 2H) ppm; ^{19}F-NMR (282 MHz, $CDCl_3$, 298 K): δ = -66.82 (d, J = 9.1 Hz, 3F) ppm; ^{13}C-NMR (125 MHz, $CDCl_3$, 298 K): δ = 152.7, 135.4, 129.2, 126.6, 126.5 (q, J = 286.6 Hz), 123.7, 122.8, 71.0 (q, J = 27.1 Hz), 52.0, 34.7, 29.9, 26.6, 24.3, 16.3 ppm.

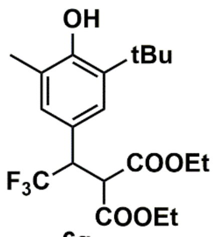

6g

6g: Prepared according to the general procedure 5 starting from **12d** in 72% yield (colorless oil).

R_f (CH_2Cl_2:heptanes = 2:1) = 0.42.

HRMS (ESI): m/z calculated for $C_{16}H_{21}F_3O$: 405.1883 $[M+H]^+$; found: 405.1881.

^{1}H-NMR (700 MHz, $CDCl_3$, 298 K): δ = 7.05 (s, 1H), 6.94 (s, 1H), 4.80 (s, 1H), 4.30-4.23 (m, 2H), 4.16-4.13 (m, 1H), 4.06-4.04 (m, 1H), 3.92-3.85 (m, 2H), 2.22 (s, 3H), 1.40 (s, 9H), 1.31 (t, J = 7.1 Hz, 3H), 0.94 (t, J = 7.1 Hz, 3H) ppm; ^{19}F-NMR (282 MHz, $CDCl_3$, 298 K): δ = -68.51 (d, J = 8.4 Hz, 3F) ppm; ^{13}C-NMR (125 MHz, $CDCl_3$, 298 K): δ = 167.0, 165.9, 153.1, 143.2, 135.8, 129.7, 127.0 (q, J = 267.4 Hz), 126.5, 123.0, 122.9, 62.4, 61.9, 53.1, 49.0 (q, J = 27.5 Hz), 34.7, 29.8, 16.1, 14.0, 13.8 ppm.

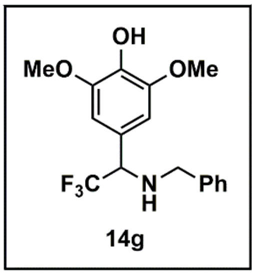

<u>14g</u>: Prepared according to the general procedure 5 starting from **12e** in 54% yield (yellow oil).

R_f (CH_2Cl_2:heptanes = 2:1) = 0.25.

HRMS (ESI): m/z calculated for $C_{17}H_{18}F_3NO_3$: 342.1312 $[M+H]^+$; found: 342.1315.

^{1}H-NMR (300 MHz, $CDCl_3$, 298 K): δ = 7.34-7.28 (m, 5H), 6.63 (s, 1H), 5.58 (br s, 1H), 4.03 (q, J = 7.6 Hz, 1H), 3.90 (s, 6H), 3.89-3.79 (m, 1H), 3.70 (s, 1H), 3.67-3.63 (m, 1H) ppm; ^{19}F-NMR (282 MHz, $CDCl_3$, 298 K): δ = -74.11 (d, J = 7.6 Hz, 3F) ppm; ^{13}C-NMR (125 MHz, $CDCl_3$, 298 K): δ = 153.1, 147.3, 139.1, 135.4, 128.7, 128.3, 127.5, 126.6 (q, J = 287.4 Hz), 125.1, 105.5, 63.6 (q, J = 28.1 Hz), 56.5, 51.1 ppm.

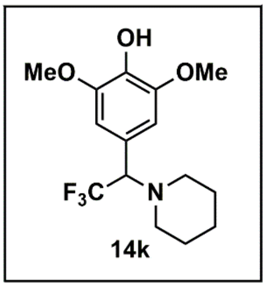

<u>14k</u>: Prepared according to the general procedure 5 starting from **12e** in 64% yield (yellow oil).

R_f (CH_2Cl_2:heptanes = 2:1) = 0.25.

HRMS (ESI): m/z calculated for $C_{15}H_{20}F_3NO_3$: 320.1468 $[M+H]^+$; found: 320.1474.

^{1}H-NMR (300 MHz, $CDCl_3$, 298 K): δ = 6.61 (s, 2H), 5.53 (s, 1H), 3.95-3.87 (m, 7H), 2.64-2.56 (m, 2H), 2.54-2.47 (m, 2H), 1.60-1.53 (m, 4H), 1.43-1.37 (m, 2H) ppm; ^{19}F-NMR (282 MHz, $CDCl_3$, 298 K): δ = -66.41 (d, J = 8.7 Hz, 3F) ppm; ^{13}C-NMR (125 MHz, $CDCl_3$, 298 K): δ = 146.9, 134.9, 128.1, 124.1, 122.9 (q, J = 288.9 Hz), 106.2, 71.2 (q, J = 26.7 Hz), 56.5, 52.0, 26.6, 24.3 ppm.

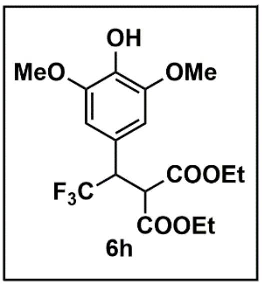

<u>6h</u>: Prepared according to the general procedure 5 starting from **12e** in 62% yield (yellow oil).

R_f (CH_2Cl_2:heptanes = 2:1) = 0.25.

HRMS (ESI): m/z calculated for $C_{17}H_{21}F_3O_7$: 395.1312 $[M+H]^+$; found: 395.1321.

^{1}H-NMR (300 MHz, $CDCl_3$, 298 K): δ = 6.53 (s, 1H), 5.53 (s, 1H), 4.31-4.25 (m, 2H), 4.20-4.04 (m, 2H), 3.94-3.92 (m, 2H), 3.88 (s, 6H), 1.31 (t, J = 7.2 Hz, 3H), 0.99 (t, J = 7.2 Hz, 3H)ppm; ^{19}F-NMR (282 MHz, $CDCl_3$, 298 K): δ = -68.40 (d, J = 8.1 Hz, 3F) ppm; ^{13}C-NMR (125 MHz, $CDCl_3$, 298 K): δ = 166.8, 165.8, 147.0, 135.3, 124.7 (q, J = 276.1 Hz), 106.6, 62.6, 62.1, 56.5, 53.1, 49.5 (q, J = 27.5 Hz), 14.1, 13.9 ppm.

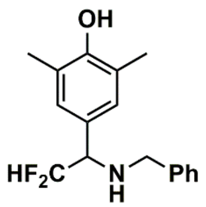

14d-CF2H

<u>14d-CF2H</u>: Prepared according to the general procedure 5 starting from **12b-CF₂H** in 62% yield (yellow oil).

R_f (CH_2Cl_2:heptanes = 2:1) = 0.33.

HRMS (ESI): m/z calculated for $C_{17}H_{19}F_2NO$: 292.1507 $[M+H]^+$; found: 292.1511.

^{1}H-NMR (500 MHz, $CDCl_3$, 298 K): δ = 7.35-7.32 (m, 2H), 7.28-7.25 (m, 3H), 7.03 (s, 2H), 5.77 (td, J_1 = 55.8 Hz, J_2 = 5.3 Hz, 1H), 5.28 (s, 1H), 3.83 (td, J_1 = 6.7 Hz, J_2 = 5.3 Hz, 1H), 3.75 (d, J = 13.5 Hz, 1H), 3.58 (d, J = 13.5 Hz, 1H), 2.26 (s, 1H) ppm; ^{19}F-NMR (470 MHz, $CDCl_3$, 298 K): δ = -123.96 (dd, J_1 = 55.8 Hz, J_2 = 6.7 Hz, 1F), -124.50 (dd, J_1 = 55.8 Hz, J_2 = 6.7 Hz, 1F) ppm; ^{13}C-NMR (125 MHz, $CDCl_3$, 298 K): δ = 153.9, 139.7, 132.0, 129.0, 128.7, 128.3, 127.4, 117.2 (t, J = 245.5 Hz), 97.8, 63.6 (t, J = 21.7 Hz), 51.0, 16.9 ppm.

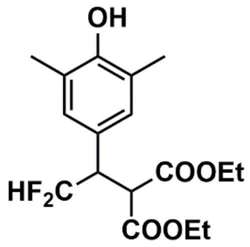

6e-CF2H

<u>6e-CF_2H</u>: Prepared according to the general procedure 5 starting from **12b-CF_2H** in 58% yield (yellow oil).

R_f (CH_2Cl_2:heptanes = 2:1) = 0.76.

HRMS (ESI): m/z calculated for $C_{17}H_{22}F_2O_5$: 345.1508 $[M+H]^+$; found: 345.1510.

^{1}H-NMR (500 MHz, $CDCl_3$, 298 K): δ = 6.89 (s, 2H), 6.09 (td, J_1 = 56.2 Hz, J_2 = 3.0 Hz, 1H), 4.58 (s, 1H), 4.25 (q, J = 7.1 Hz, 2H), 3.96 (q, J = 7.1 Hz, 2H), 3.80-3.74 (m, 2H), 2.21 (s, 6H), 1.29 (t, J = 7.1 Hz, 3H), 1.00 (t, J = 7.1 Hz, 3H) ppm; ^{19}F-NMR (470 MHz, $CDCl_3$, 298 K): δ = -120.3 - -122.2 (m, 2F) ppm; ^{13}C-NMR (125 MHz, $CDCl_3$, 298 K): δ = 167.7, 166.9, 125.3, 129.9, 124.6, 123.2, 116.2 (t, J = 243.6 Hz), 62.2, 61.7 52.8, 48.1 (t, J = 20.1 Hz), 41.8, 16.1, 14.1, 13.8 ppm.

2.5 Reactions with Indole

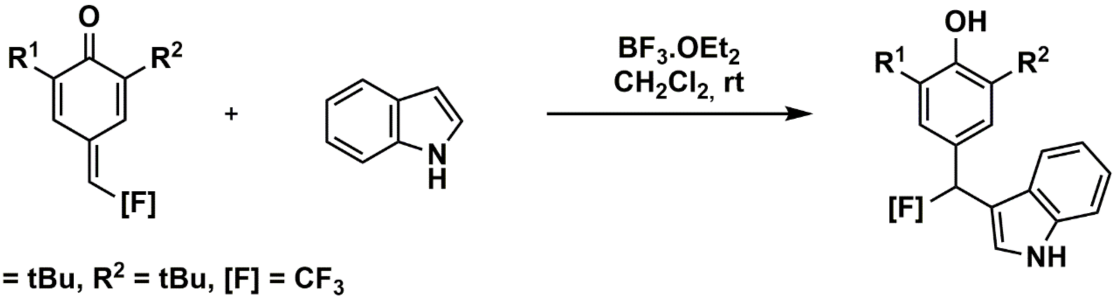

1a: R^1 = tBu, R^2 = tBu, [F] = CF_3
1b: R^1 = Me, R^2 = Me, [F] = CF_3
1d: R^1 = tBu, R^2 = Me, [F] = CF_3

13a-b, 13d

General procedure 6: To 0.1 mmol of the corresponding quinone methide **1a** or **1b** and 0.12 mmol indole, in 2 mL CH_2Cl_2, 20 mol% of BF_3.OEt$_2$ were added. After stirring the reaction for 16 h at room temperature, the solvent was evaporated and the crude product was purified by column chromatography (CH_2Cl_2:heptanes = 2:1) to yield the products in the yields stated below.

General procedure 7 (in situ formation of the quinone methide): The chlorinated QM precursor **12d** (0.1 mmol) was dissolved in 2 mL CH_2Cl_2 and NEt_3 (0.12 mmol) was added. This reaction mixture was stirred for 1 h at room temperature and then indole (0.12 mmol) and BF_3.OEt$_2$ (0.2 mmol) were added. After stirring the reaction for 16 h at room temperature, the solvent was evaporated and the crude product was purified by column chromatography (CH_2Cl_2:heptanes = 2:1) to yield product **13d**.

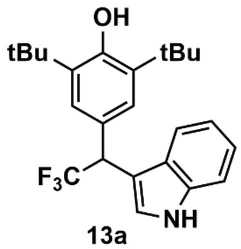

13a

<u>13a:</u> Prepared according to the general procedure 6 in 88% isolated yield (brown oil).

R_f (CH_2Cl_2:heptanes = 2:1) = 0.68.

HRMS (ESI): m/z calculated for $C_{24}H_{28}F_3NO$: 404.2196 $[M+H]^+$; found: 404.2196.

^{1}H-NMR (300 MHz, $CDCl_3$, 298 K): δ = 8.15 (s, 1H), 7.54-7.52 (m, 1H), 7.40-7.37 (m, 1H), 7.30-7.28 (m, 1H), 7.25-7.19 (m, 2H), 7.15-7.10 (m, 1H), 5.19 (s, 1H), 4.89 (q, J = 9.8 Hz, 1H), 1.42 (s, 18H) ppm; ^{19}F-NMR (282 MHz, $CDCl_3$, 298 K): δ = -67.38 (d, J = 9.8 Hz, 3F) ppm; ^{13}C-NMR (125 MHz, $CDCl_3$, 298 K): δ = 153.5, 135.9, 127.0, 126.8 (q, J = 278.2 Hz), 126.1, 125.9, 122.9, 122.5, 120.0, 119.2, 111.3, 47.6 (q, J = 29.1 Hz), 34.5, 30.3, 29.2 ppm.

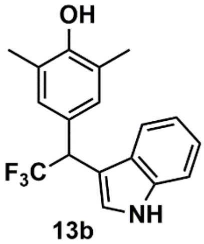

13b

13b: Prepared according to the general procedure 6 in 55% yield (brown oil).

HRMS (ESI): m/z calculated for $C_{18}H_{16}F_3NO$: 320.1257 $[M+H]^+$; found: 320.1255.

R_f (CH_2Cl_2:heptanes = 2:1) = 0.18.

^{1}H-NMR (300 MHz, $CDCl_3$, 298 K): δ = 8.16 (s, 1H), 7.41-7.36 (m, 2H), 7.31 (s, 1H), 7.22-7.17 (m, 1H), 7.10-7.05 (m, 1H) 7.03 (s, 2H), 4.84 (q, J = 9.5 Hz, 1H), 4.58 (s, 1H), 2.21 (s, 6H) ppm; ^{19}F-NMR (282 MHz, $CDCl_3$, 298 K): δ = -67.58 (d, J = 9.5 Hz, 3F) ppm; ^{13}C-NMR (125 MHz, $CDCl_3$, 298 K): δ = 152.1, 136.0, 129.6, 127.3 (q, J = 288.9 Hz), 127.0, 123.1, 122.7, 122.6, 122.0, 119.0, 111.3, 110.7, 47.1 (q, J = 28.8 Hz), 29.8, 16.1 ppm.

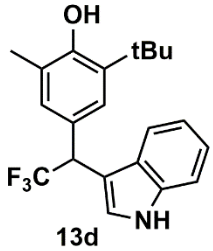

13d

13d: Prepared according to the general procedure 7 in 59% yield (yellow oil).

R_f (CH_2Cl_2:heptanes = 2:1) = 0.71.

HRMS (ESI): m/z calculated for $C_{21}H_{22}F_3NO$: 362.1726 $[M+H]^+$; found: 362.1722.

^{1}H-NMR (300 MHz, $CDCl_3$, 298 K): δ = 8.14 (s, 1H), 7.46-7.43 (m, 1H), 7.39-7.36 (m, 1H), 7.30 (s, 1H), 7.23-7.18 (m, 2H), 7.12-7.06 (m, 1H), 7.03 (s, 1H), 4.87 (q, J = 9.3 Hz, 1H), 4.73 (s, 1H), 2.19 (s, 3H), 1.40 (s, 9H) ppm; ^{19}F-NMR (282 MHz, $CDCl_3$, 298 K): δ = -67.50 (d, J = 9.3 Hz, 3F) ppm; ^{13}C-NMR (125 MHz, $CDCl_3$, 298 K): δ = 152.5, 136.0, 135.7, 129.2, 127.3 (q, J = 281.0 Hz), 127.0, 126.6, 126.5, 123.1, 122.8, 122.6, 120.0, 119.1, 111.3, 111.0, 47.3 (q, J = 29.1 Hz), 34.6, 29.8, 29.1, 16.2 ppm.

2.6 Spirocyclopropanation reaction

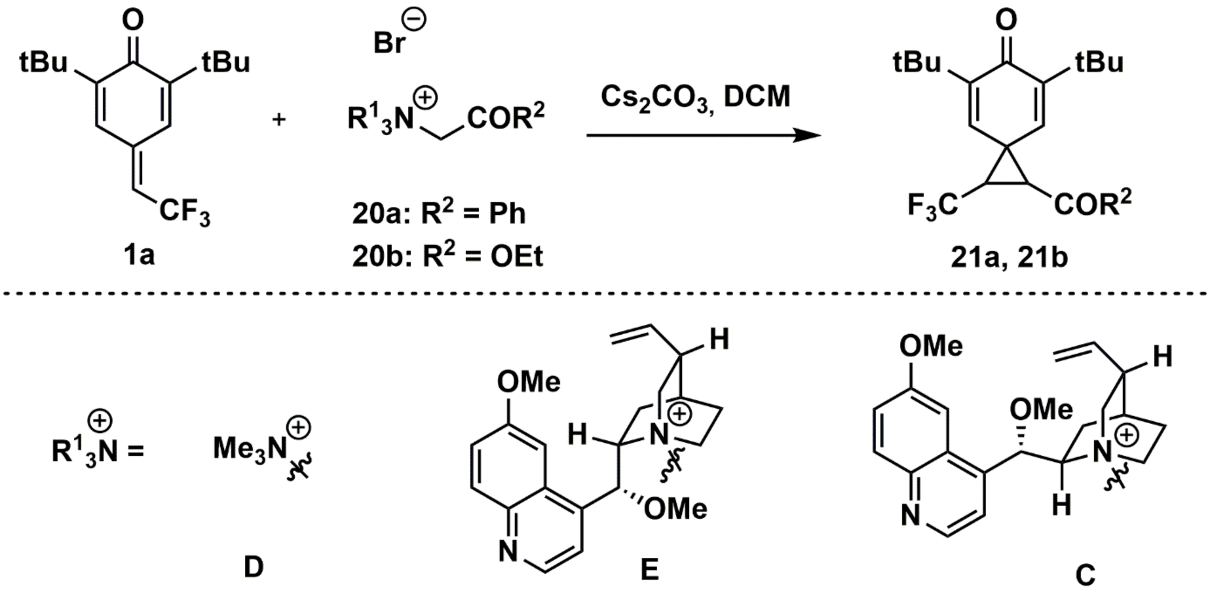

General procedure 8: QM **1a** (0.2 mmol, 57.2 mg) and Cs_2CO_3 (0.4 mmol, 130.4 mg) were suspended in 2 mL DCM. Then the corresponding ammonium salt **20** (0.3 mmol) was added and the mixture stirred at RT for 24 h. Afterwards the reaction mixture was dried with Na_2SO_4, filtered and then concentrated *in vacuo*. The crude product was purified by column chromatography on silica gel (Heptanes/EtOAc = 50:1).

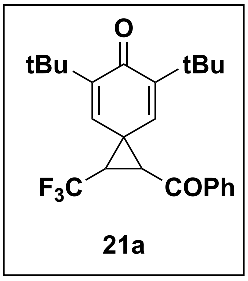

21a: The compound was prepared according to the general procedure 8 using achiral and chiral ammonium salts **20**. The product occurs as yellow solid with a melting range of 122.9 – 123.3°C. When using Me_3N-based salt **20** the product was obtained in 79% yield with a d.r. >20:1. With E the product was obtained in 76% with d.r. > 20:1 and an e.r. of 77:23. With C the product was obtained in 78% with d.r. > 20:1 and an e.r. of 88:12.

R_f (CH_2Cl_2:heptanes = 2:1) = 0.77.

C: $[\alpha]_d^{22}$ (c = 1.00, dichloromethane, 77:23 e.r.)= 31.3°.

E: $[\alpha]_d^{22}$ (c = 1.00, dichloromethane, 88:12 e.r.)= -41.3°.

HRMS (ESI): m/z calculated for $C_{24}H_{27}F_3O_2$: 405.2036 $[M+H]^+$; found: 405.2035.

^{1}H-NMR (300 MHz, $CDCl_3$, 298 K): δ = 7.83-7.80 (m, 2H), 7.64-7.59 (m, 1H), 7.51-7.46 (m, 2H), 6.55 (s, 1H), 6.23 (s, 1H), 3.77 (d, J = 7.1 Hz, 1H), 3.35 (qu, J = 7.1 Hz, 1H), 1.30 (s, 9H), 1.10 (s, 8H) ppm; ^{19}F-NMR (282 MHz, $CDCl_3$, 298 K): δ = -60.12 (d, J = 7.1 Hz, 3F) ppm; ^{13}C-NMR (125 MHz, $CDCl_3$, 298 K): δ = 191.9, 185.3, 152.2, 151.4, 136.6, 134.6, 134.2, 133.8, 133.5 (q, J = 282.6 Hz), 129.1, 128.4, 37.2, 36.1, 35.5 (q, J = 35.5 Hz), 29.5, 29.4 ppm.

Column: Chiralcel YMC-SB (250 x 4.6 mm, 5 µm) chiral stationary phase; flow: 0.5 mL/min; Temperature: 10 °C; Hexane/IPA: 200/1.

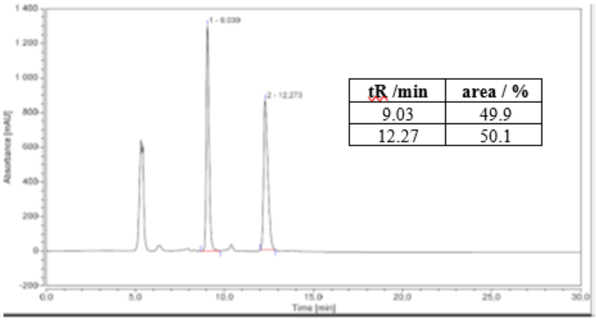

tR /min	area / %
9.03	49.9
12.27	50.1

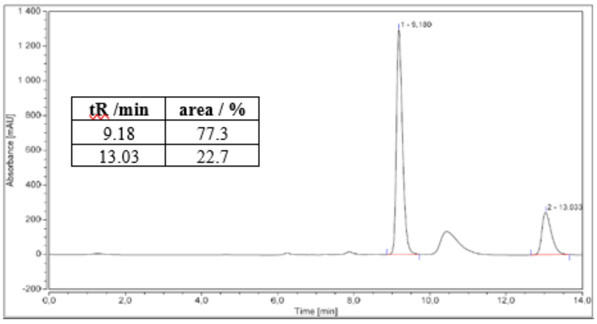

tR /min	area / %
9.18	77.3
13.03	22.7

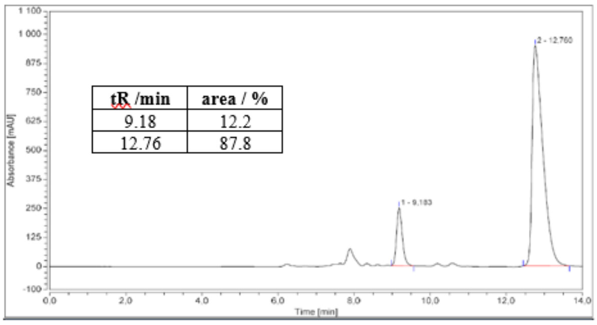

tR /min	area / %
9.18	12.2
12.76	87.8

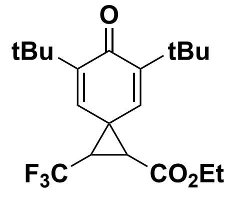

21b

21b: Compound was prepared according to the general procedure 8 in 76% yield (d.r. > 20:1). The product occurs as yellow solid with a melting range of 115.2 – 115.9°C.

R_f (CH_2Cl_2:heptanes = 2:1) = 0.74.

HRMS (ESI): m/z calculated for $C_{20}H_{27}F_3O_3$: 373.1985 $[M+H]^+$; found: 373.1988.

^{1}H-NMR (300 MHz, $CDCl_3$, 298 K): δ = 6.52 (s, 1H), 6.32 (s, 1H), 4.24 (q, J = 7.3 Hz, 2H), 3.00 (qu, J = 7.3 Hz, 1H), 2.89 (d, J = 7.3 Hz, 1H), 1.31 (t, J = 7.1 Hz, 3H), 1.24 (s, 9H), 1.22 (s, 9H) ppm; ^{19}F-NMR (282 MHz, $CDCl_3$, 298 K): δ = -60.39 (d, J = 7.3 Hz, 3F) ppm; ^{13}C-NMR (125 MHz, $CDCl_3$, 298 K): δ = 185.6, 167.6, 151.4, 151.1, 134.7, 134.1, 124.1 (q, J = 276.8 Hz), 62.4, 35.5, 35.1, 34.1, 33.2, 30.4, 29.5, 29.5 ppm.

2.7 Reactions with Glycine Schiff Base

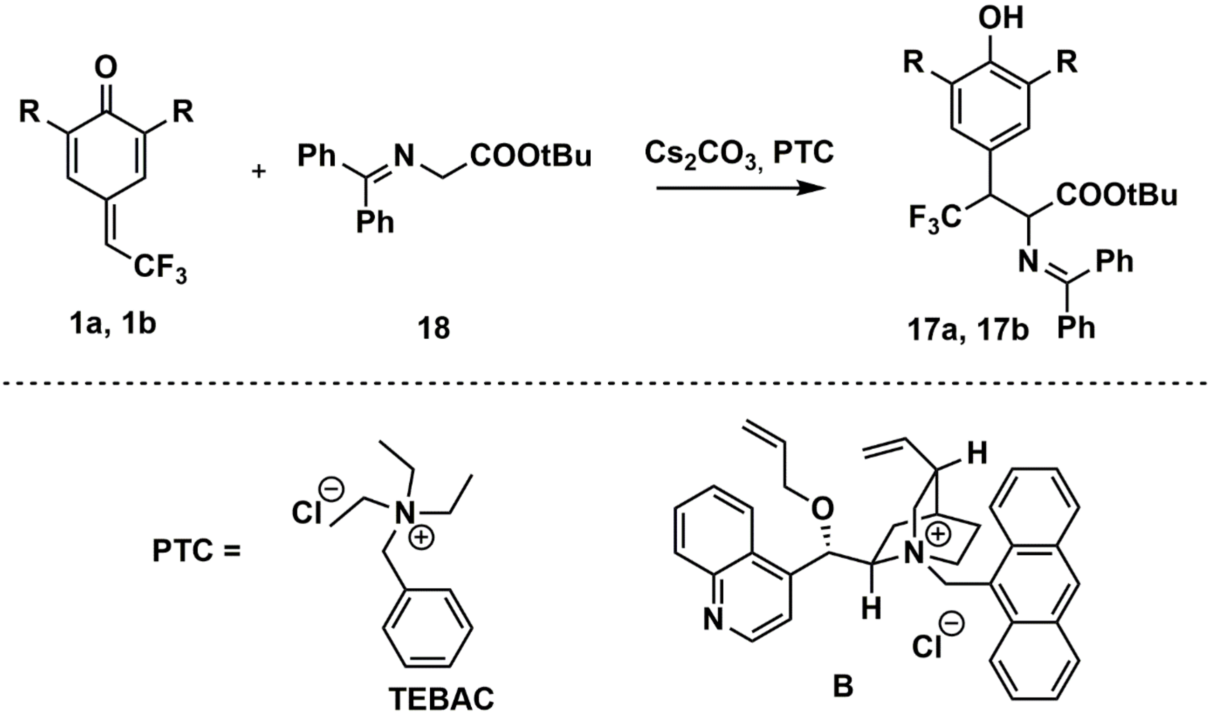

General Procedure 9: The quinone methides **1a** or **1b** (0.1 mmol), the glycine Schiff base **18** (0.1 mmol) and the chiral or achiral PTC (10 mol%) were dissolved in 2 mL CH_2Cl_2 and Cs_2CO_3 (0.11 mmol) was added. After the reaction was stirred for 24 h at room temperature, the mixture was filtered over Na_2SO_4, washed with Et_2O and evaporated to dryness. Products **17** were purified by column chromatography (DCM:Heptane = 2:1).

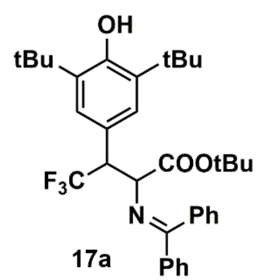

17a

<u>**17a**</u>: Prepared according to the general procedure 9, the product occurs as white oil, with an isolated yield of 96% (using TEBAC) or 84% (catalyst **B**). The asymmetric conditions resulted in a d.r. = 7:1 and an e.r. = 90:10.

R_f (CH_2Cl_2:heptanes = 2:1) = 0.70.

$[\alpha]_d^{22}$ (c = 1.00, dichloromethane, 90:10 e.r.)= 12.3°.

HRMS (ESI): m/z calculated for $C_{35}H_{42}F_3NO_3$: 582.3196 $[M+H]^+$; found: 582.3191.

^{1}H-NMR (300 MHz, $CDCl_3$, 298 K): δ = 7.62-7.59 (m, 2H), 7.44-7.42 (m, 5H), 7.38-7.35 (m, 1H), 7.31-7.29 (m, 2H), 7.08-7.05 (m, 2H), 5.18 (s, 1H), 4.50 (d, J = 5.7 Hz, 1H), 4.02-3.91 (m, 1H), 1.43 (1s, 18H), 1.25 (s, 9H) ppm; ^{19}F-NMR (282 MHz, $CDCl_3$, 298 K): δ = -65.73 (d, J = 9.9 Hz, 3F) ppm; ^{13}C-NMR (125 MHz, $CDCl_3$, 298 K): δ = 171.1, 168.7, 153.8, 139.7, 136.2, 135.4, 130.4, 129.1, 128.9, 128.5,128.0, 127.7, 126.6 (q, J = 279.7 Hz), 123.4, 81.8, 52.6 (q, J = 25.9 Hz), 34.5, 30.4, 27.8 ppm.

Column: Chiralcel AD-H (250 x 4.6 mm, 5 µm) chiral stationary phase; flow: 0.5 mL/min; Temperature: 10 °C; Hexane/IPA: 99/1.

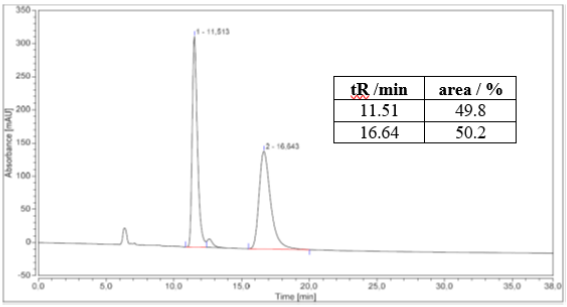

tR /min	area / %
11.51	49.8
16.64	50.2

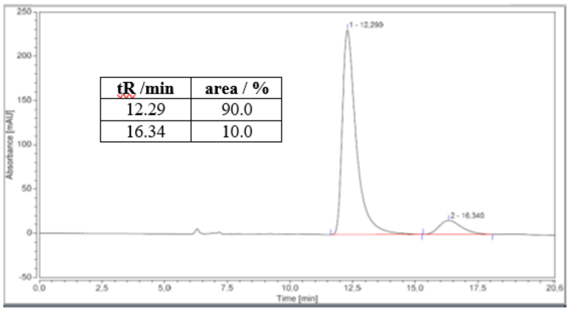

tR /min	area / %
12.29	90.0
16.34	10.0

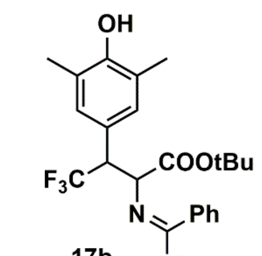

17b

<u>**17b**</u>: Prepared according to the general procedure 9 using TEBAC in 84% isolated yield and with a d.r. = 5:1 (colorless oil).

R_f (CH_2Cl_2:heptanes = 2:1) = 0.53.

HRMS (ESI): m/z calculated for $C_{29}H_{30}F_3NO_3$: 498.2251 $[M+H]^+$; found: 498.2255.

^{1}H-NMR (300 MHz, $CDCl_3$, 298 K): δ = 7.33-7.28 (m, 2H), 7.27-7.22 (m, 3H), 7.19-7.00 (m, 4H), 6.92-6.87 (m, 3H), 4.50 (s, 1H), 4.31 (d, J = 5.9 Hz, 1H), 3.90-3.76 (m, 1H), 2.03 (s, 6H), 1.16 (s, 8H) ppm; ^{19}F-NMR (282 MHz, $CDCl_3$, 298 K): δ = -66.05 (d, J = 9.7 Hz, 3F) ppm; ^{13}C-NMR (125 MHz, $CDCl_3$, 298 K): δ = 136.2, 131.1, 130.4, 129.1, 128.9, 128.3, 128.1, 128.0, 126.5 (q, J = 272.5 Hz), 123.7, 122.9, 122.6, 82.0, 65.6, 52.1 (q, 26.2 Hz), 27.9, 16.0 ppm.

2.8 Deprotection and Debutylation of 17a

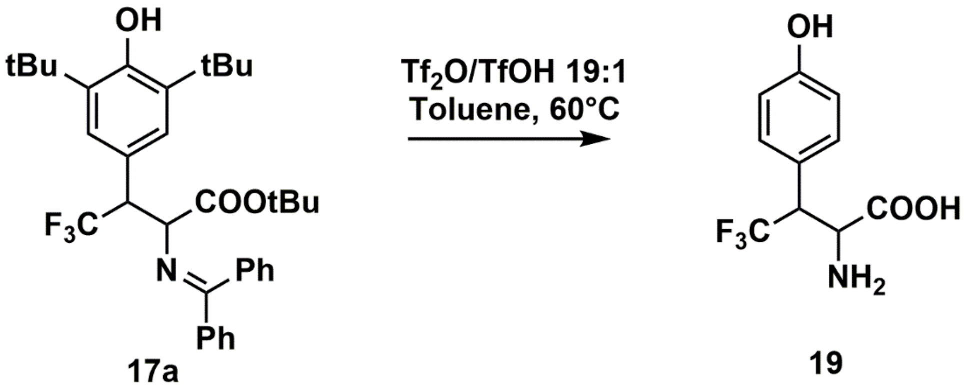

17a **19**

General procedure 10: Compound **17a** (0.1 mmol) was dissolved in 10 mL toluene and a mixture of Tf_2O and TfOH (19/1, 20 µL) was added. After stirring the reaction for 24 h at 60 °C, the reaction was quenched with water and extracted two times with EtOAc. The water phase was evaporated to dryness to yield the debutylated and deprotected amino acid **19**.

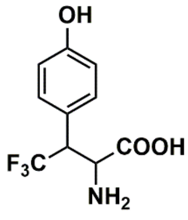

19

19: Prepared according to the general procedure 10 in 62% yield with d.r. = 8.2:1 (colorless oil).

HRMS (ESI): m/z calculated for $C_{10}H_{10}F_3NO_3$: 250.0686 $[M+H]^+$; found: 250.0688.

^{1}H-NMR (300 MHz, D_2O, 298 K): δ = 7.33-7.30 (m, 2H), 6.97-6.94 (m, 2H), 4.65 (d, J = 5.8 Hz, 1H), 4.48-4.36 (m, 1H) ppm; ^{19}F-NMR (282 MHz, $CDCl_3$, 298 K): δ = -66.51 (d, J = 9.7 Hz, 3F) ppm; ^{13}C-NMR (125 MHz, $CDCl_3$, 298 K): δ = 169.5, 156.9, 131.1, 130.0, 125.9, 125.1 (q, J = 285.2 Hz), 121.7, 119.3, 117.4, 116.4,116.0,113.3, 52.9, 48.7 (q, J = 30.2 Hz) ppm.

2.9 Hydrid- and deuterid-reduction of quinone methide 1a

General procedure 11: Quinone methide **1a** (0.1 mmol) was dissolved in 4 mL *t*-BuOH and $NaBH_4$ respectively $NaBD_4$ (0.12 mmol) was added portionwise. The reaction mixture was stirred at room temperature overnight and after completion of the reaction it was filtered over a pad of Na_2SO_4, washed with Et_2O and evaporated to dryness. The crude product was purified by column chromatography (CH_2Cl_2:heptanes = 2:1) to yield compound **8** and **8-D**.

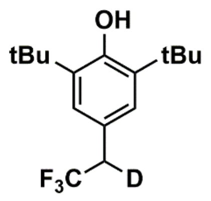

8-D

8-D: Prepared according to the general procedure 11 in 97% isolated yield (colorless oil).

R_f (CH_2Cl_2:heptanes = 2:1) = 0.91.

HRMS (ESI): m/z calculated for $C_{16}H_{22}DF_3O$: 290.1837 $[M+H]^+$; found: 290.1837.

^{1}H-NMR (300 MHz, $CDCl_3$, 298 K): δ = 7.06 (s, 2H), 5.22 (s, 1H), 3.25 (q, J = 10.7 Hz, 1H), 1.11 (s, 18H) ppm; ^{19}F-NMR (282 MHz, $CDCl_3$, 298 K): δ = -66.27 (d, J = 10.7 Hz, 3F) ppm; ^{13}C-NMR (125 MHz, $CDCl_3$, 298 K): δ = 153.7, 136.2, 126.9, 126.1, 122.8 (q, J = 262.2 Hz), 39.9 (m), 34.4, 30.4 ppm.

3. Copies of NMR-Spectra of new Compounds

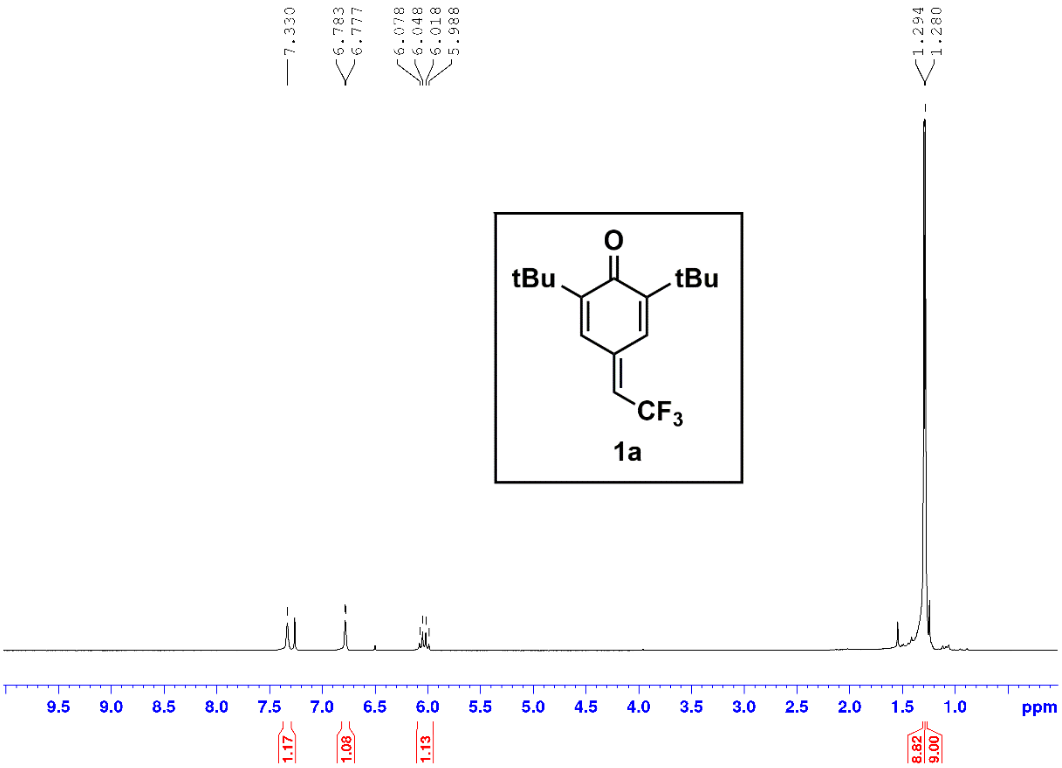

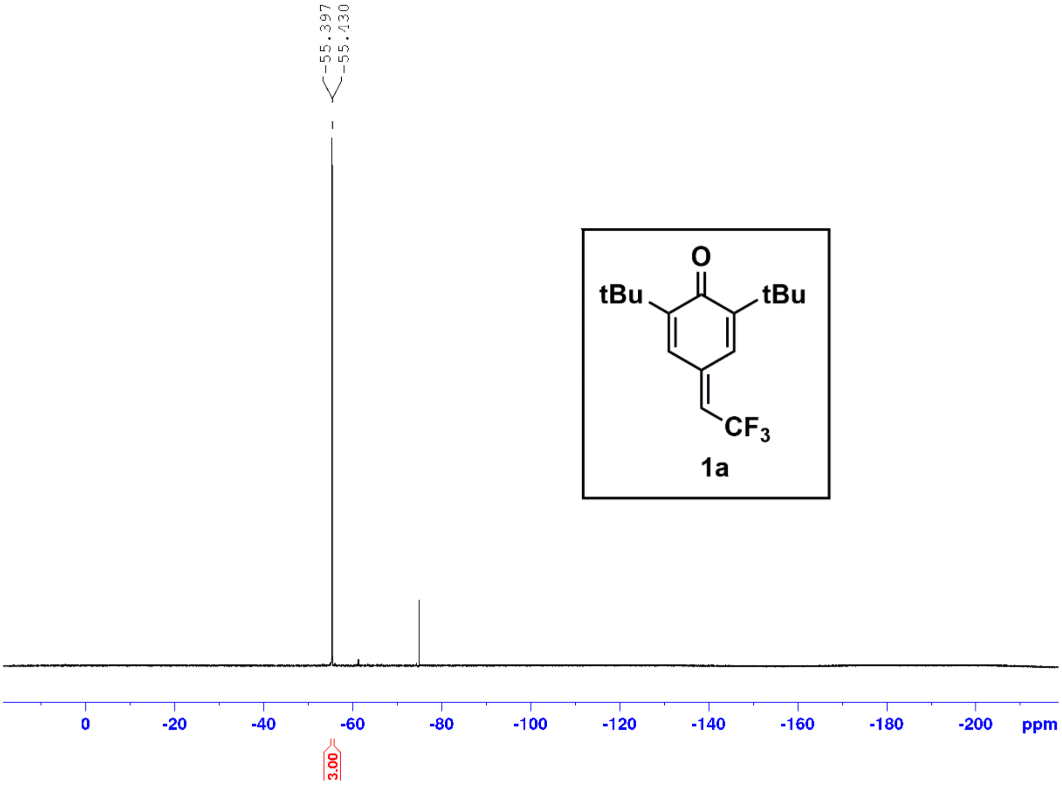

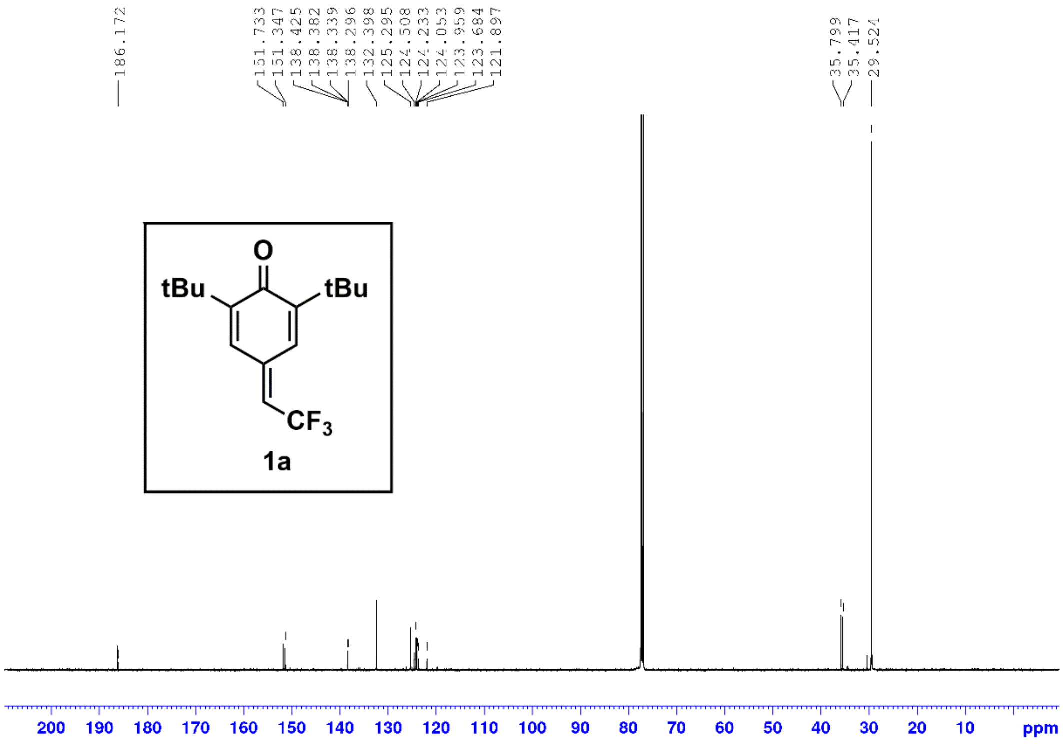
186.172
151.733
151.347
138.425
138.382
138.339
138.296
132.398
125.295
124.508
124.233
124.053
123.959
123.684
121.897
35.799
35.417
29.524
O
tBu
tBu
CF3
1a
ppm

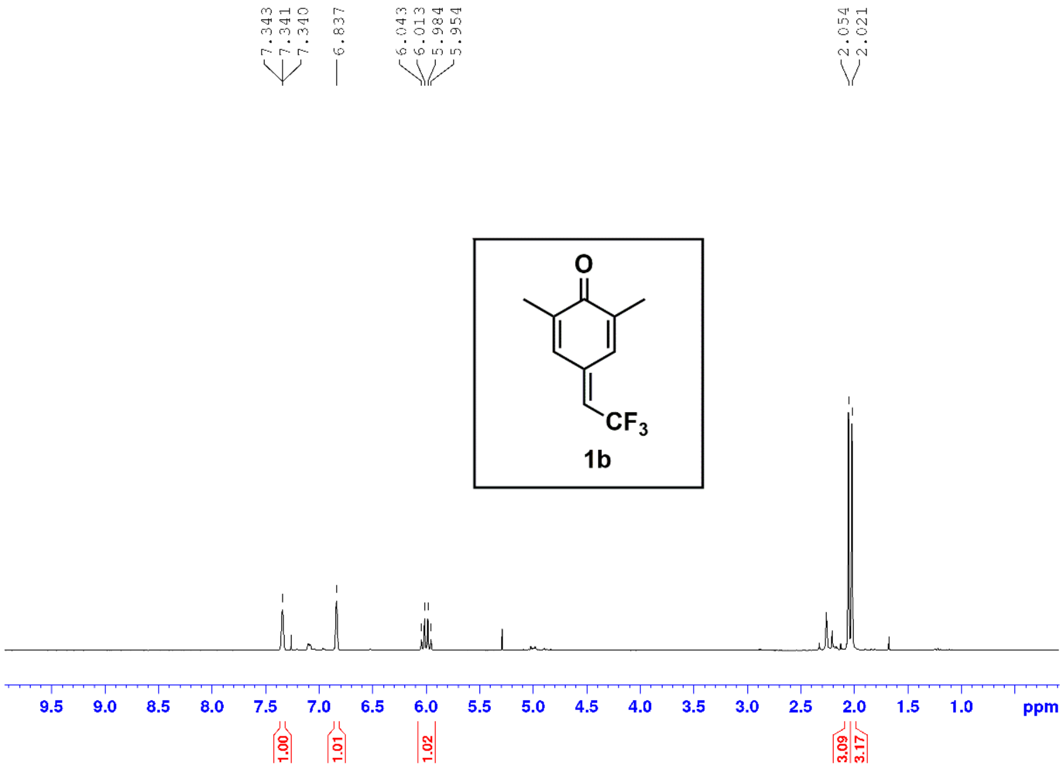
7.343
7.341
7.340
6.837
6.043
6.013
5.984
5.954
2.054
2.021
O
CF3
1b
ppm
1.00
1.01
1.02
3.09
3.17

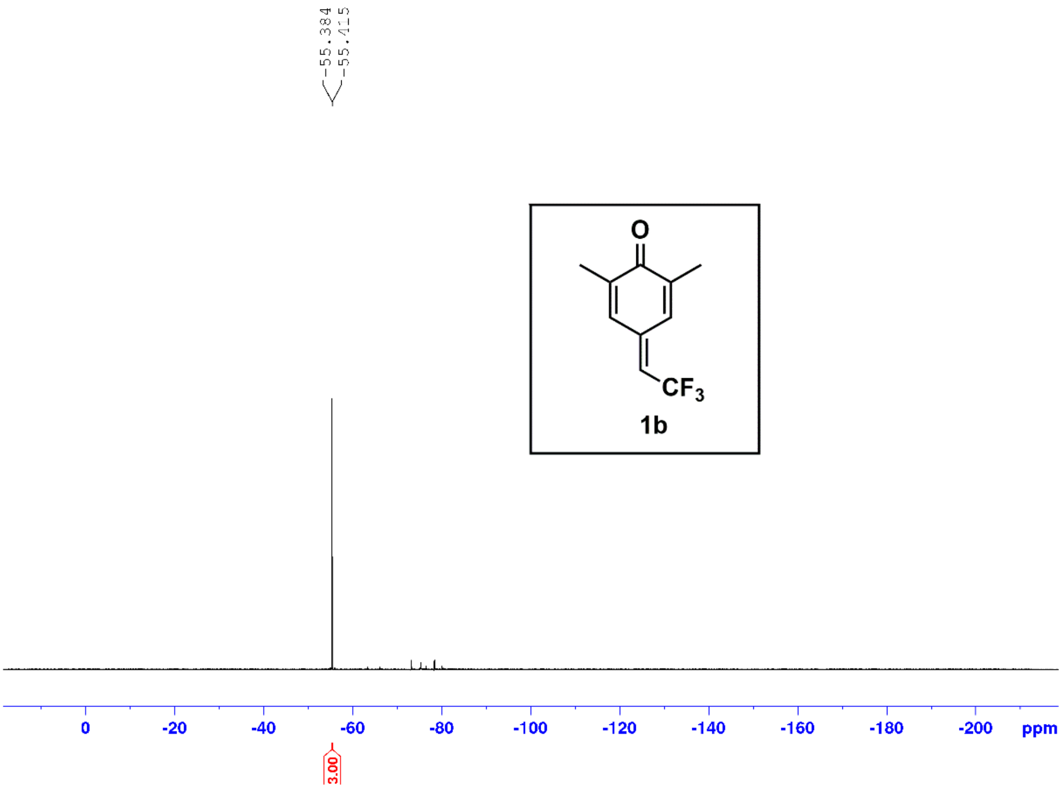
-55.384
-55.415
CF3
1b
0
-20
-40
-60
-80
-100
-120
-140
-160
-180
-200
ppm
3.00

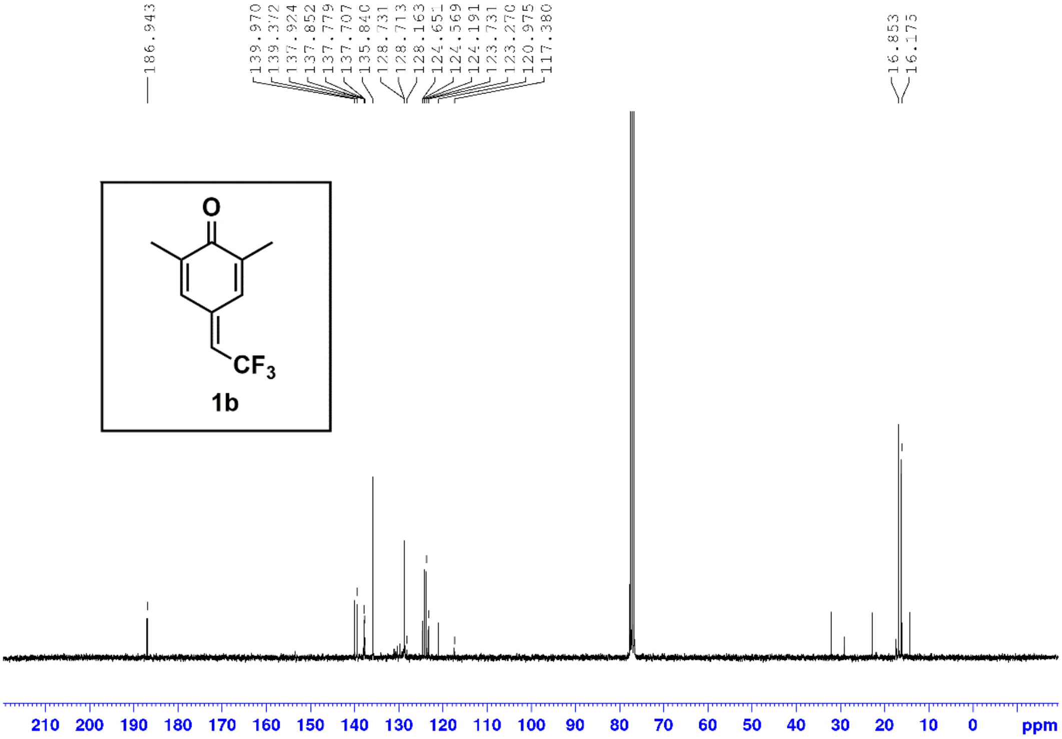
186.943
139.970
139.372
137.924
137.852
137.779
137.707
135.840
128.731
128.713
128.163
124.651
124.569
124.191
123.731
123.270
120.975
117.380
16.853
16.173
CF3
1b
210
200
190
180
170
160
150
140
130
120
110
100
90
80
70
60
50
40
30
20
10
0
ppm

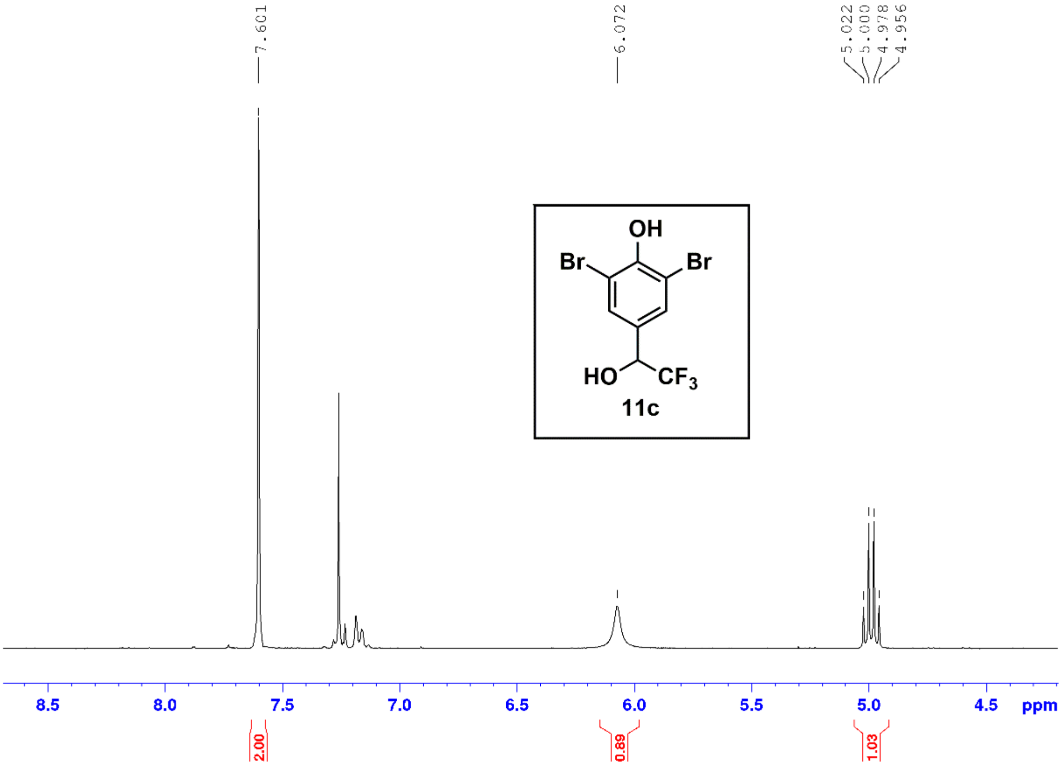
7.601
6.072
5.022
5.000
4.978
4.956
OH
Br
Br
HO
CF3
11c
8.5
8.0
7.5
7.0
6.5
6.0
5.5
5.0
4.5
ppm
2.00
0.89
1.03

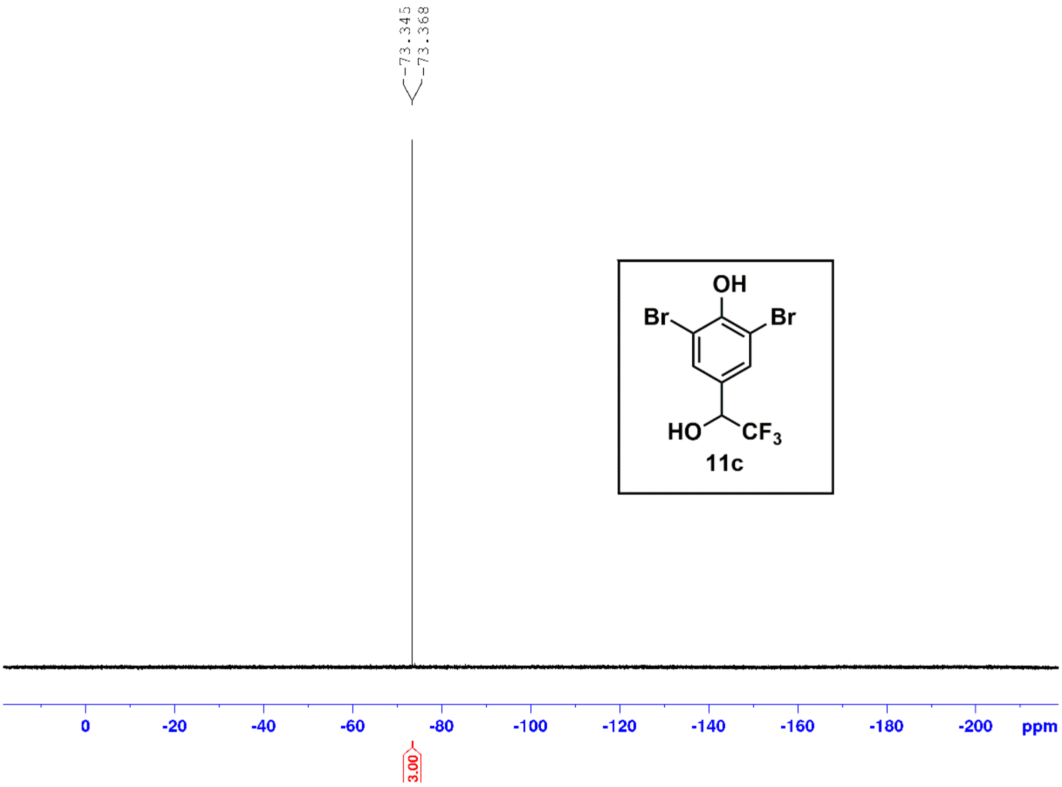
-73.345
-73.368
OH
Br
Br
HO
CF3
11c
0
-20
-40
-60
-80
-100
-120
-140
-160
-180
-200
ppm
3.00

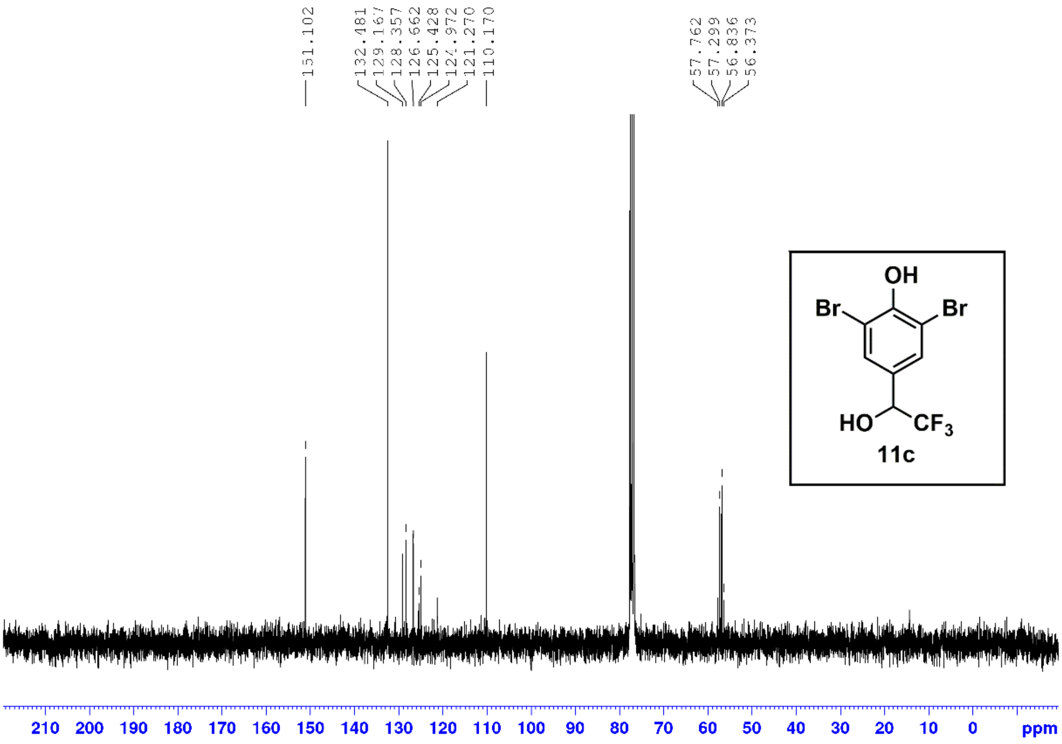
151.102
132.481
129.167
128.357
126.662
125.428
124.972
121.270
110.170
57.762
57.299
56.836
56.373
OH
Br
Br
HO
CF3
11c
210 200 190 180 170 160 150 140 130 120 110 100 90 80 70 60 50 40 30 20 10 0 ppm

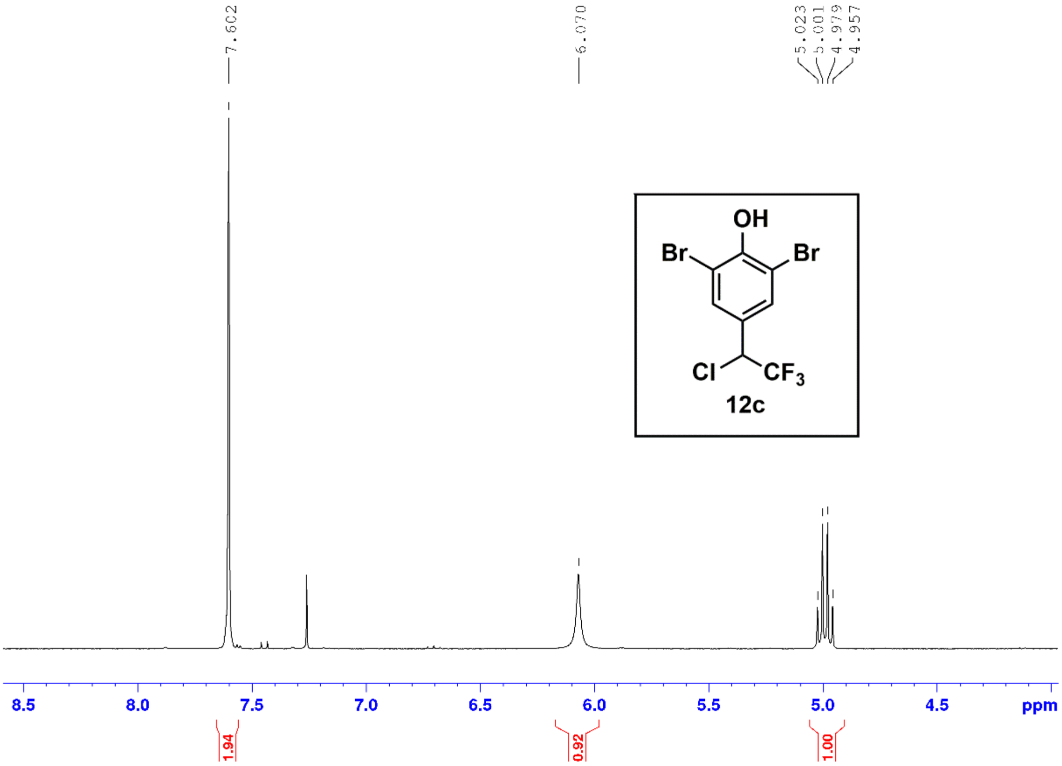
7.602
6.070
5.023
5.001
4.979
4.957
OH
Br
Br
Cl
CF3
12c
8.5 8.0 7.5 7.0 6.5 6.0 5.5 5.0 4.5 ppm
1.94
0.92
1.00

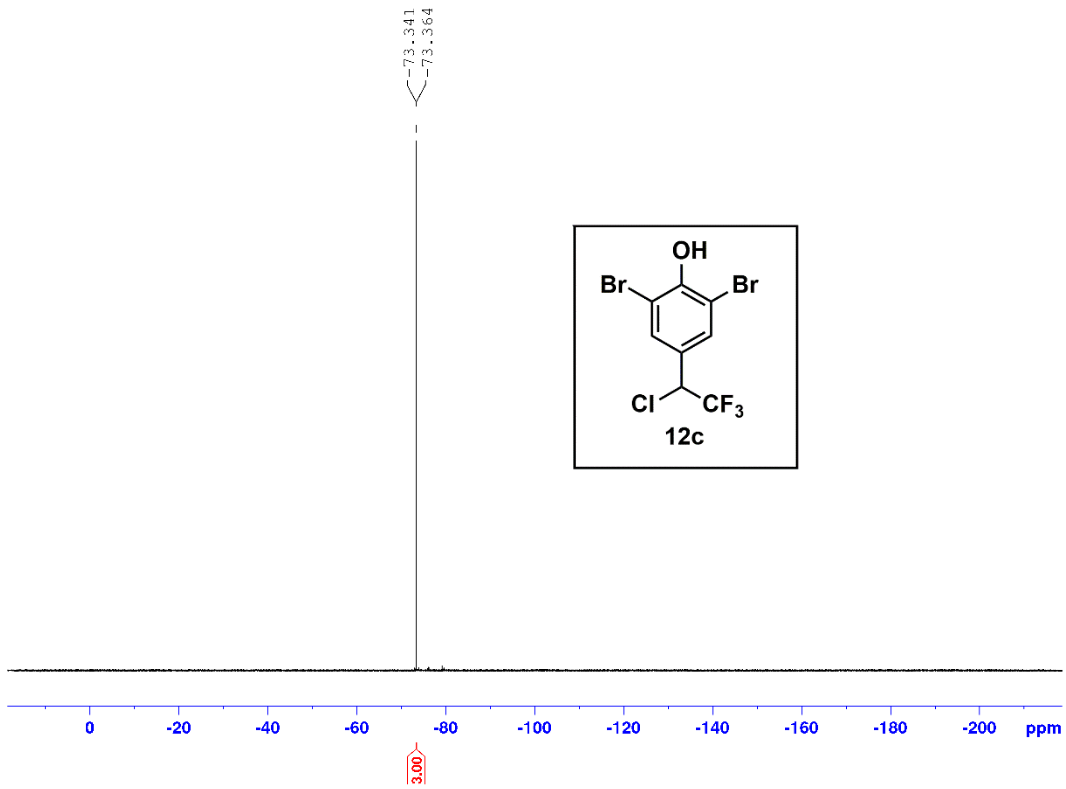
-73.341
-73.364
OH
Br
Br
Cl
CF3
12c
0 -20 -40 -60 -80 -100 -120 -140 -160 -180 -200 ppm
3.00

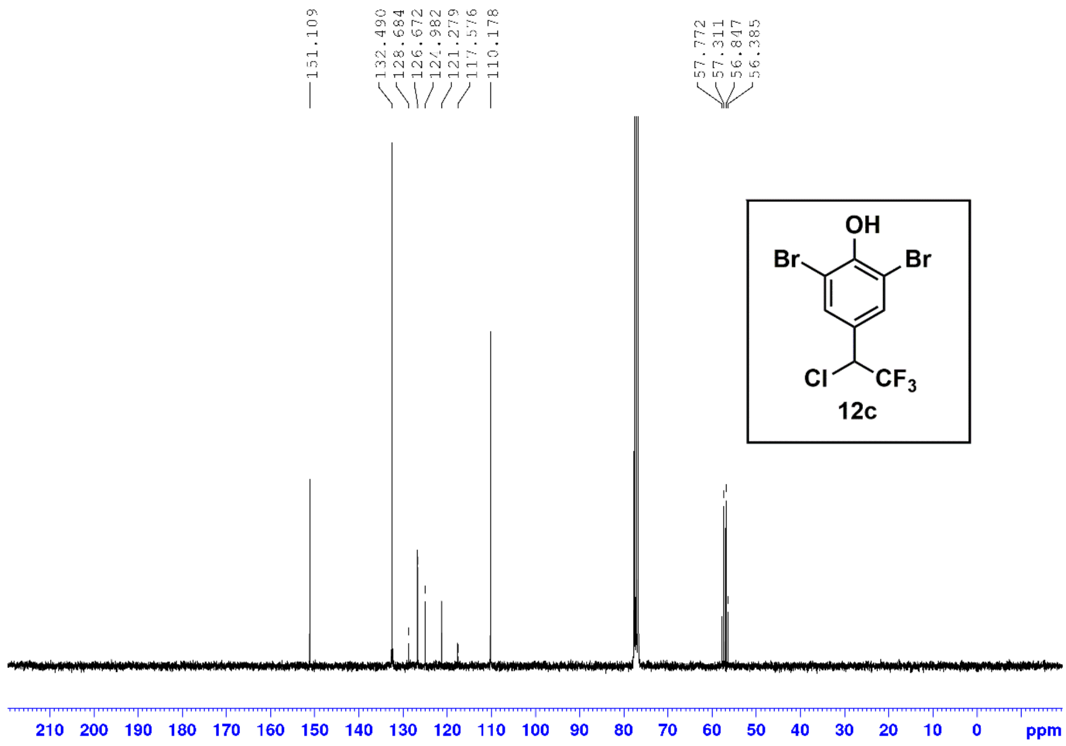
151.109
132.490
128.684
126.672
124.982
121.279
117.576
110.178
57.772
57.311
56.847
56.385
OH
Br
Br
Cl
CF3
12c
210 200 190 180 170 160 150 140 130 120 110 100 90 80 70 60 50 40 30 20 10 0 ppm

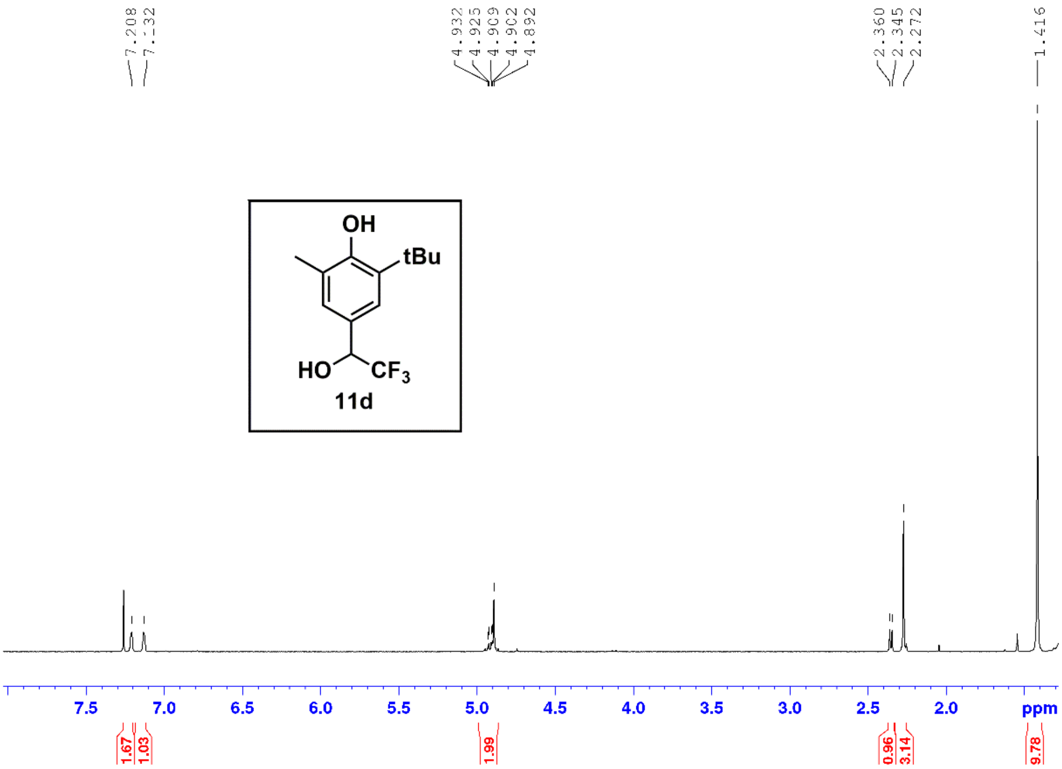
7.208
7.132
4.932
4.925
4.909
4.902
4.892
2.360
2.345
2.272
1.416
OH
tBu
HO
CF3
11d
7.5
7.0
6.5
6.0
5.5
5.0
4.5
4.0
3.5
3.0
2.5
2.0
ppm
1.67
1.03
1.99
0.96
3.14
9.78

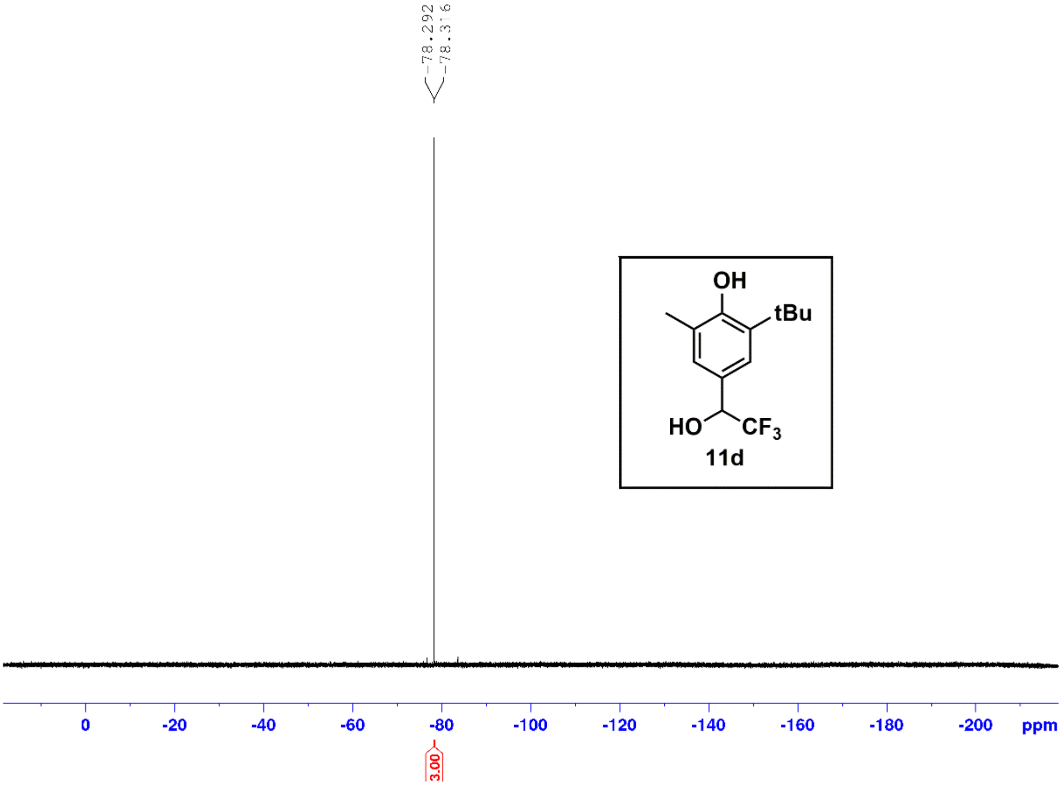
-78.292
-78.316
OH
tBu
HO
CF3
11d
0
-20
-40
-60
-80
-100
-120
-140
-160
-180
-200
ppm
3.00

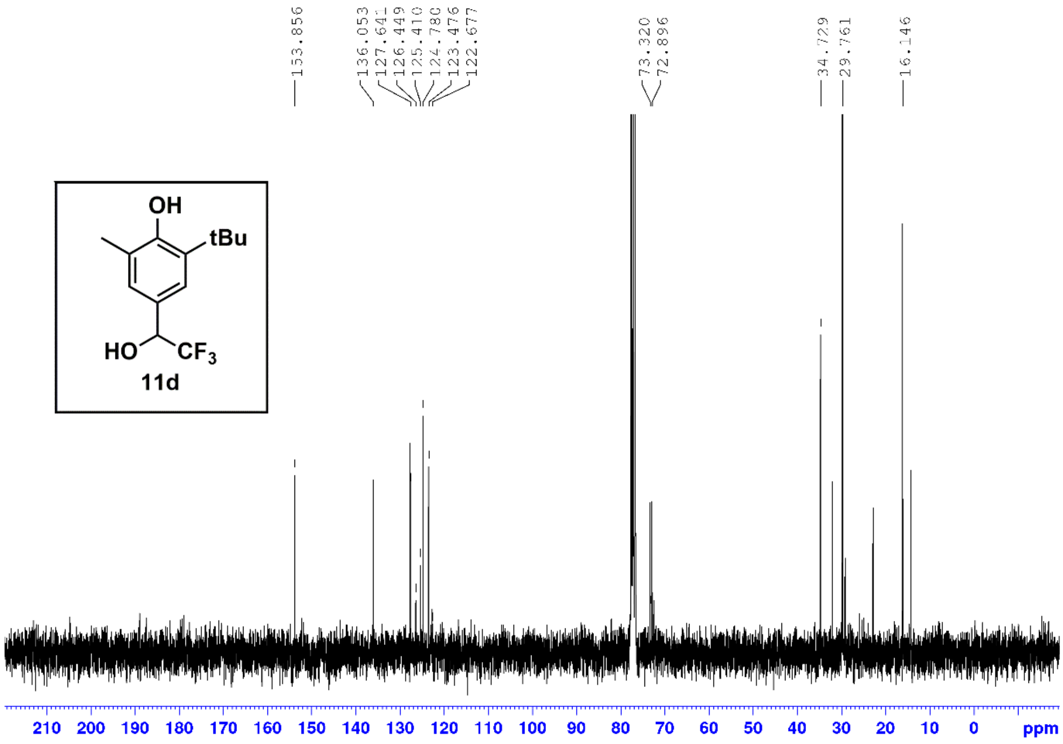

153.856
136.053
126.449
125.410
124.780
123.476
122.677
73.320
72.896
34.729
29.761
16.146
OH
tBu
HO
CF3
11d
210 200 190 180 170 160 150 140 130 120 110 100 90 80 70 60 50 40 30 20 10 0 ppm

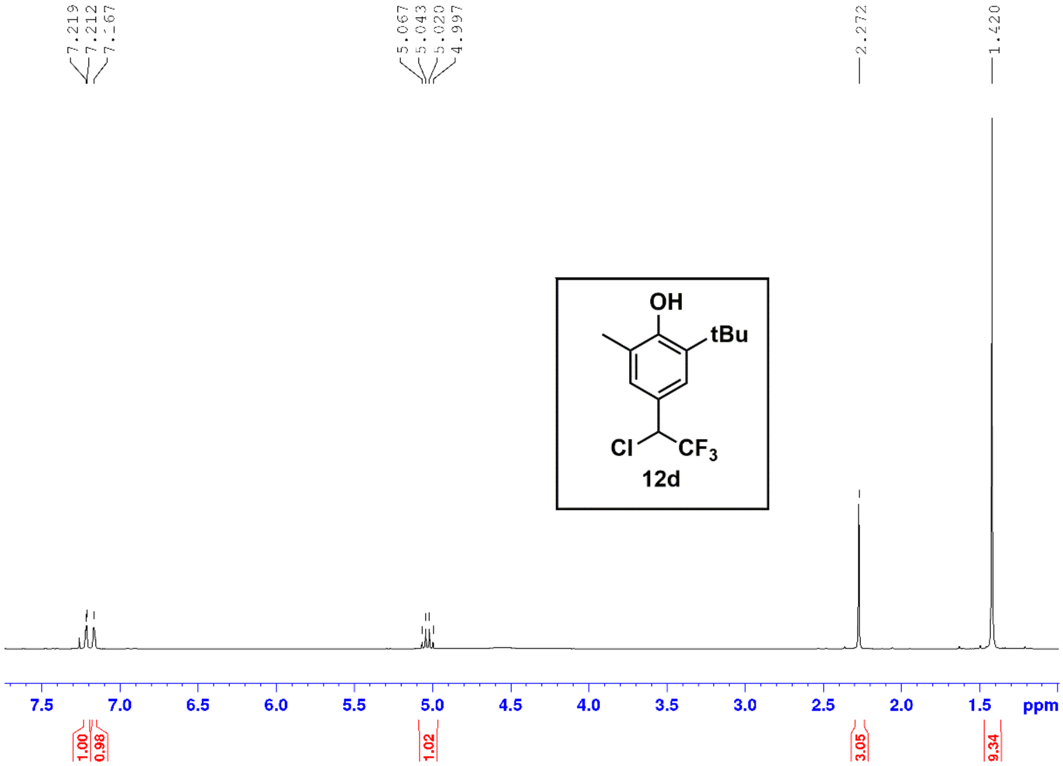

7.219
7.212
5.067
5.043
5.020
4.997
2.272
1.420
OH
tBu
Cl
CF3
12d
7.5 7.0 6.5 6.0 5.5 5.0 4.5 4.0 3.5 3.0 2.5 2.0 1.5 ppm
1.00
0.98
1.02
3.05
9.34

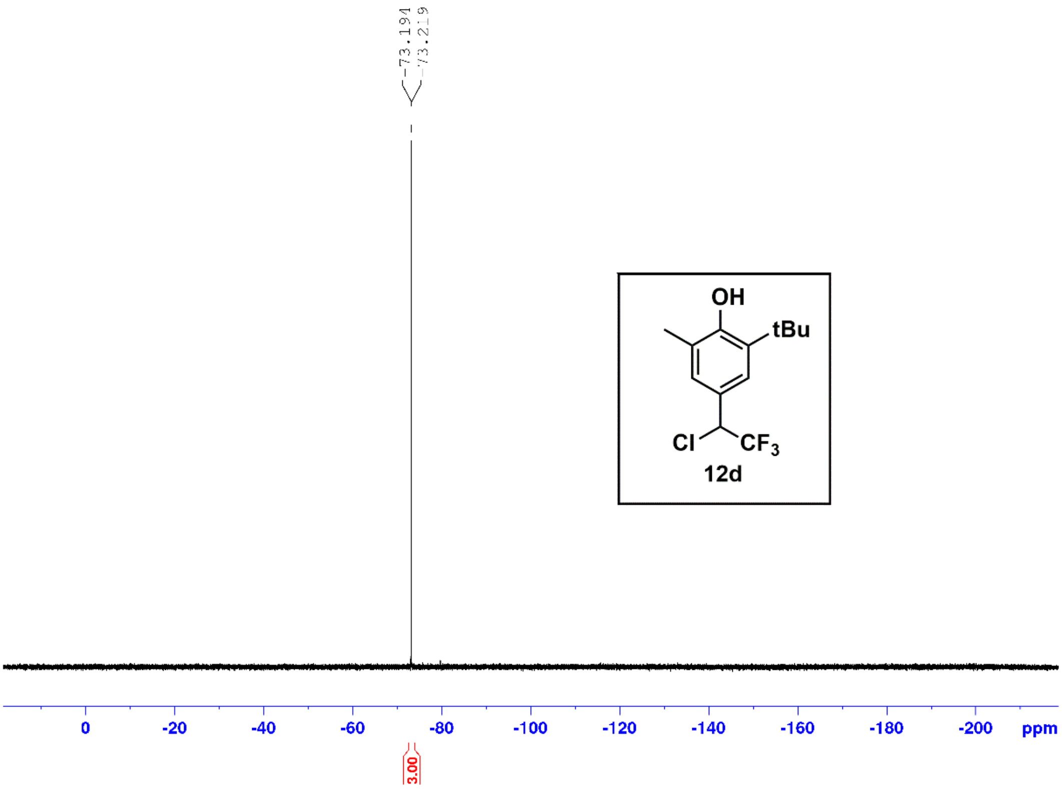
-73.194
-73.219
OH
tBu
Cl
CF3
12d
0
-20
-40
-60
-80
-100
-120
-140
-160
-180
-200
ppm
3.00

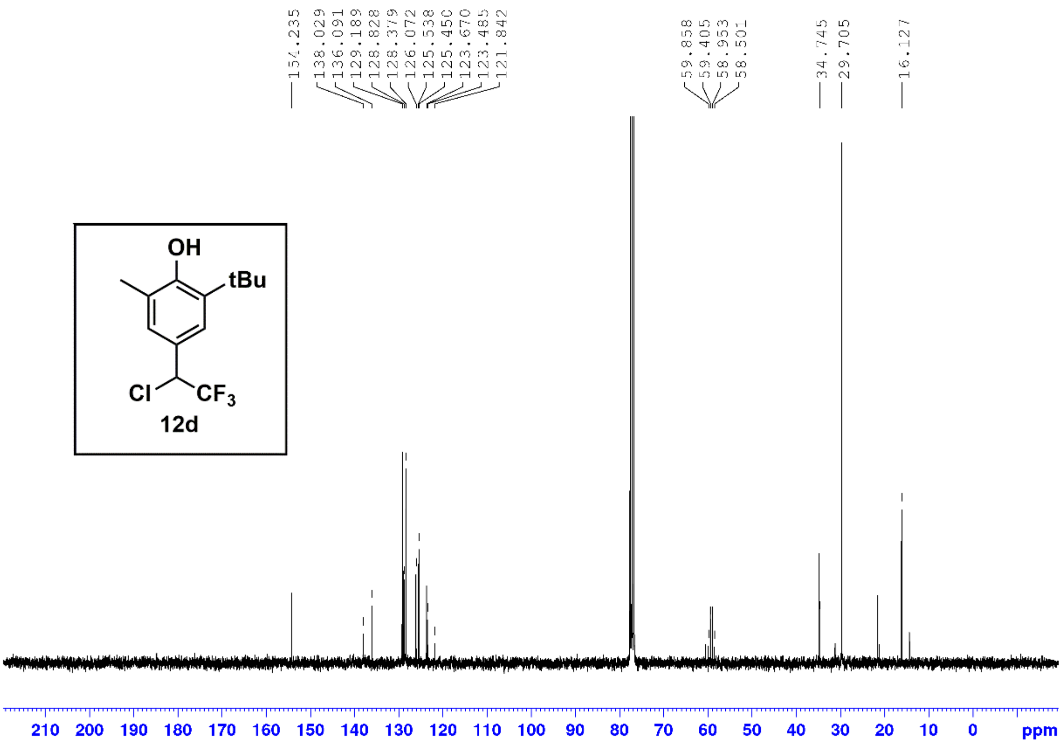
154.235
138.029
136.091
129.189
128.828
128.379
126.072
125.538
125.450
123.670
123.485
121.842
59.858
59.405
58.953
58.501
34.745
29.705
16.127
OH
tBu
Cl
CF3
12d
210
200
190
180
170
160
150
140
130
120
110
100
90
80
70
60
50
40
30
20
10
0
ppm

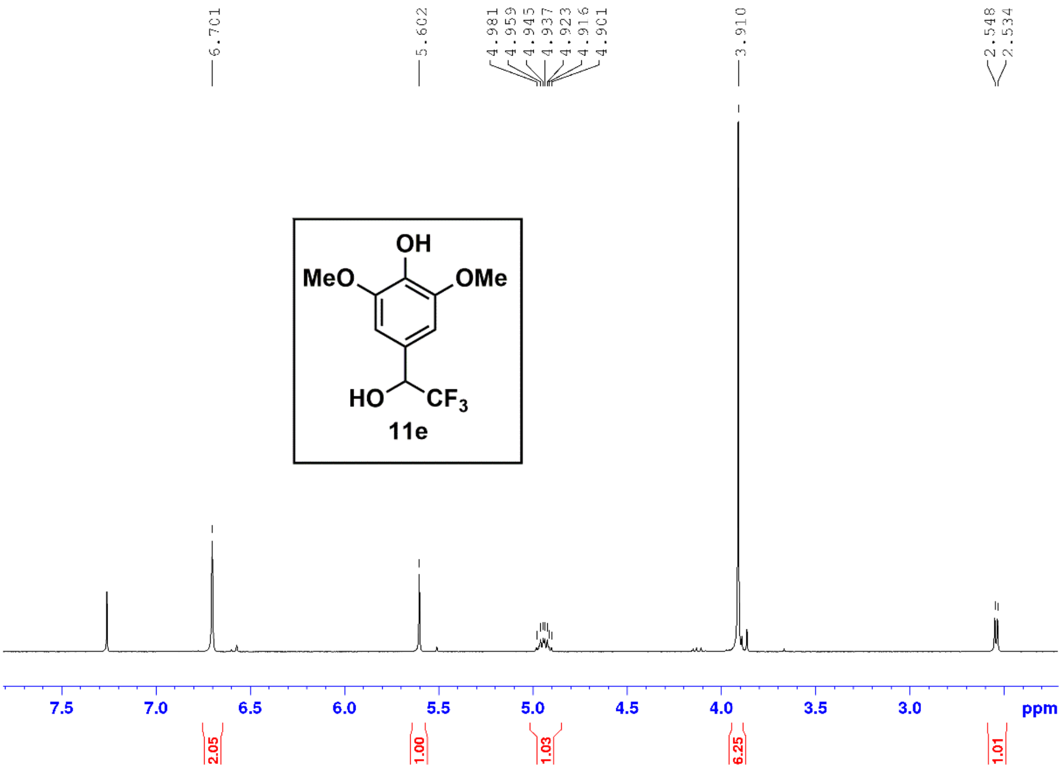
6.701
5.602
4.981
4.959
4.945
4.937
4.923
4.916
4.901
3.910
2.548
2.534
OH
MeO
OMe
HO
CF3
11e
7.5
7.0
6.5
6.0
5.5
5.0
4.5
4.0
3.5
3.0
ppm
2.05
1.00
1.03
6.25
1.01

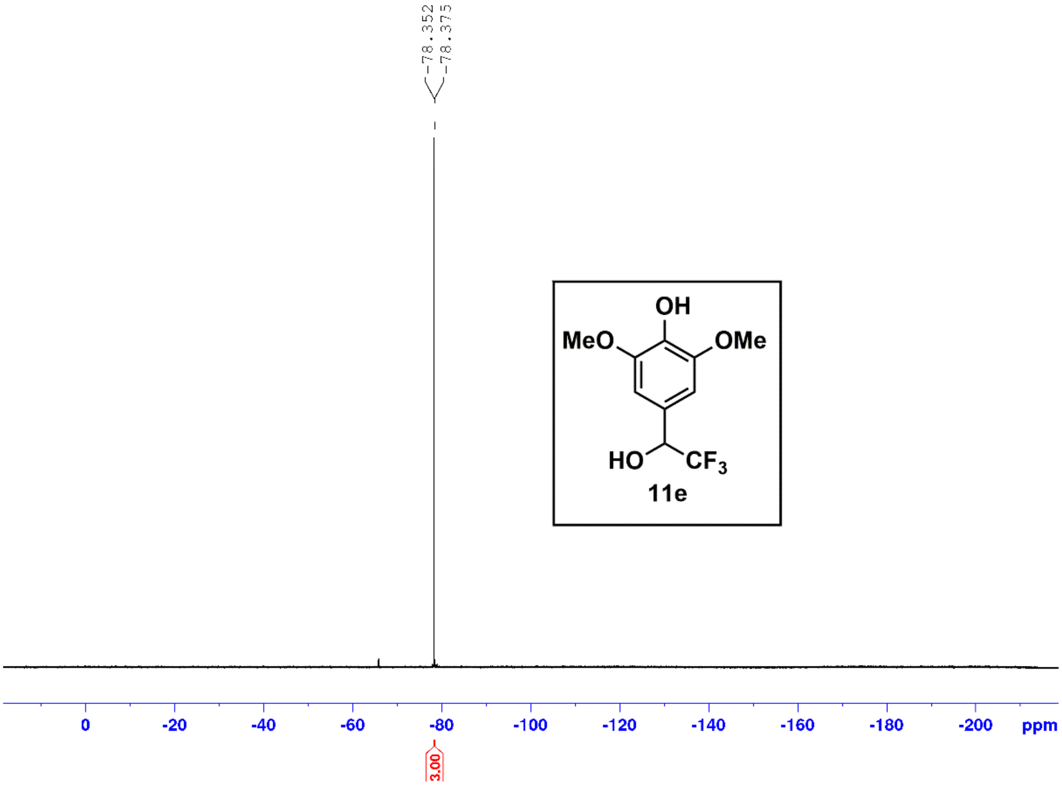
-78.352
-78.375
OH
MeO
OMe
HO
CF3
11e
0
-20
-40
-60
-80
-100
-120
-140
-160
-180
-200
ppm
3.00

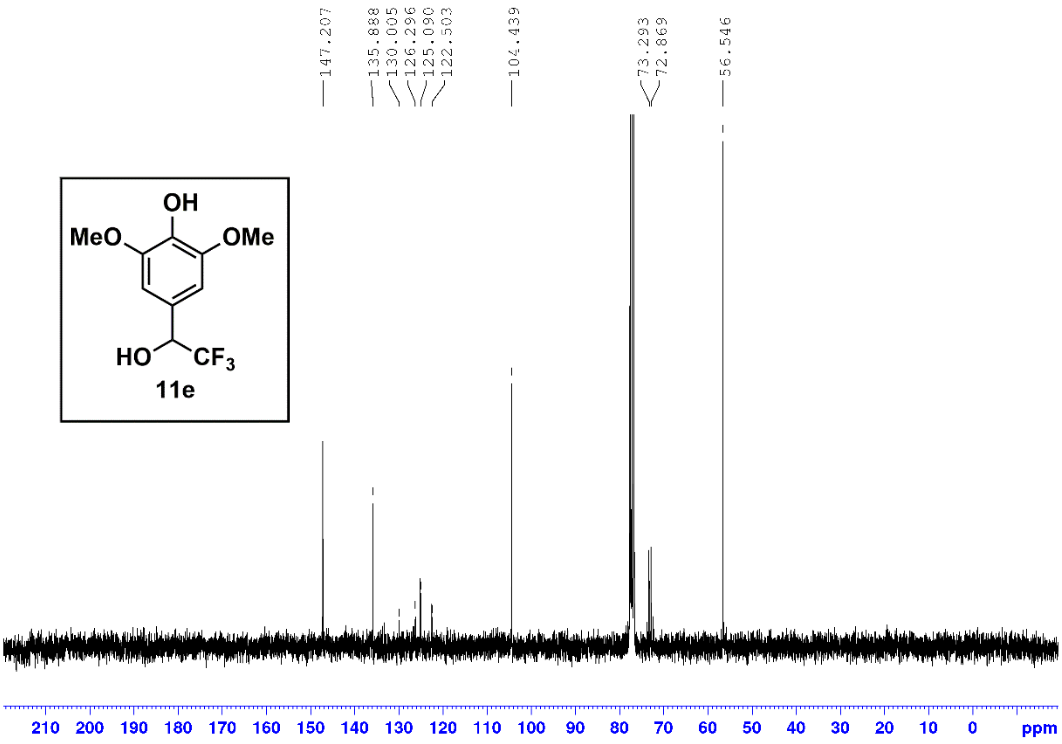
147.207
135.888
130.005
126.296
125.090
122.503
104.439
73.293
72.869
56.546
OH
MeO
OMe
HO
CF3
11e
210
200
190
180
170
160
150
140
130
120
110
100
90
80
70
60
50
40
30
20
10
0
ppm

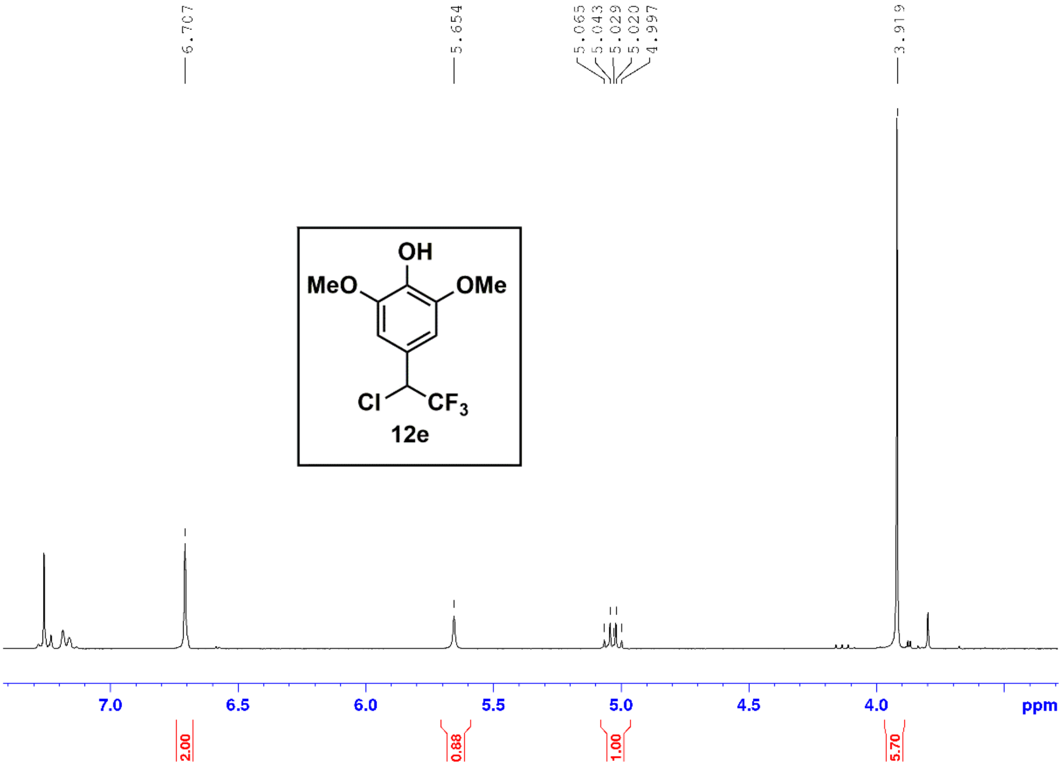
6.707
5.654
5.065
5.043
5.029
5.020
4.997
3.919
OH
MeO
OMe
Cl
CF3
12e
7.0
6.5
6.0
5.5
5.0
4.5
4.0
ppm
2.00
0.88
1.00
5.70

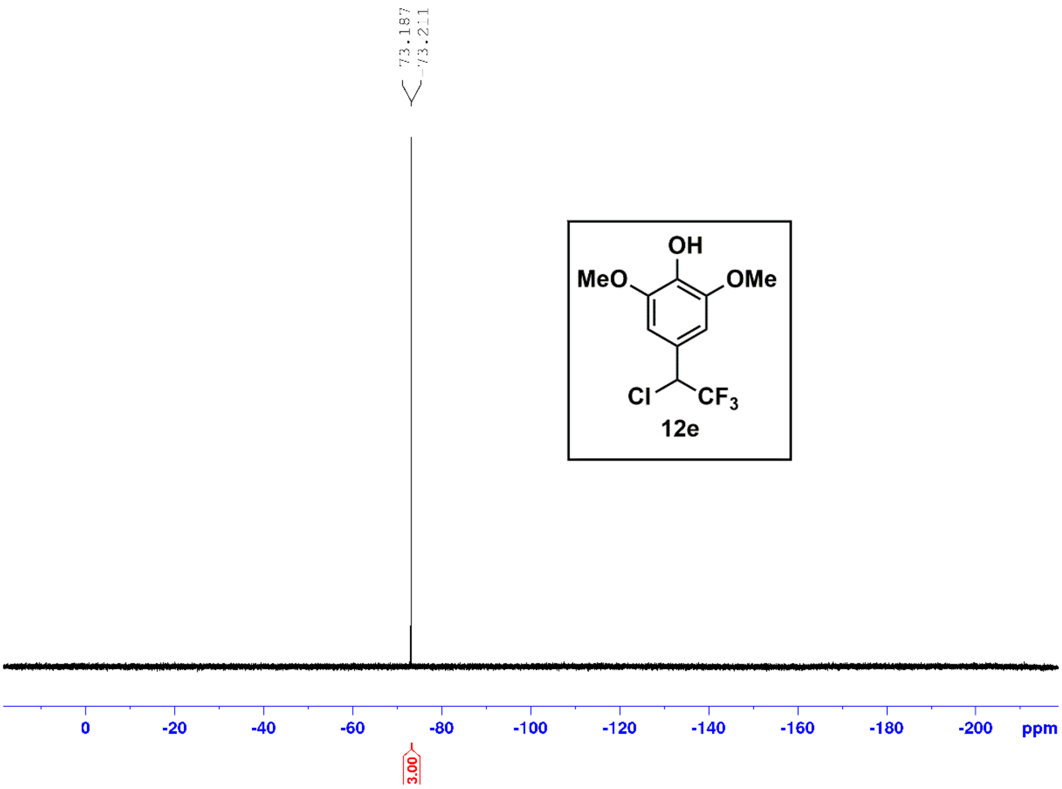
-73.187
-73.211
OH
MeO
OMe
Cl
CF3
12e
0 -20 -40 -60 -80 -100 -120 -140 -160 -180 -200 ppm
3.00

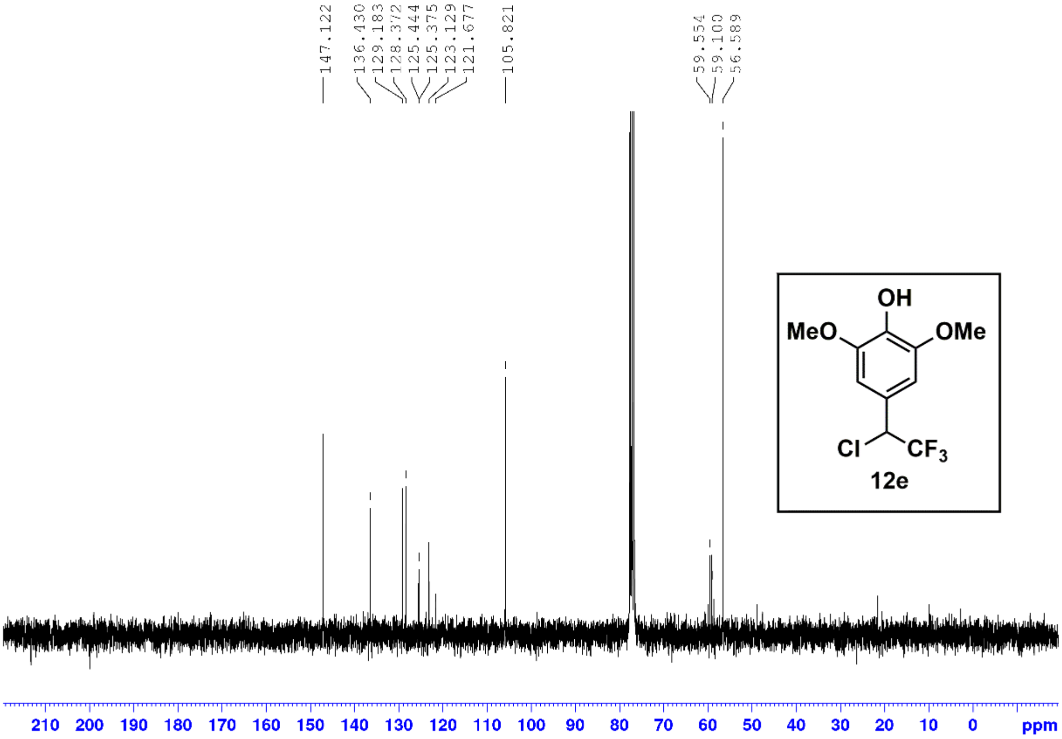
147.122
136.430
129.183
128.372
125.444
125.375
123.129
121.677
105.821
59.554
59.100
56.589
OH
MeO
OMe
Cl
CF3
12e
210 200 190 180 170 160 150 140 130 120 110 100 90 80 70 60 50 40 30 20 10 0 ppm

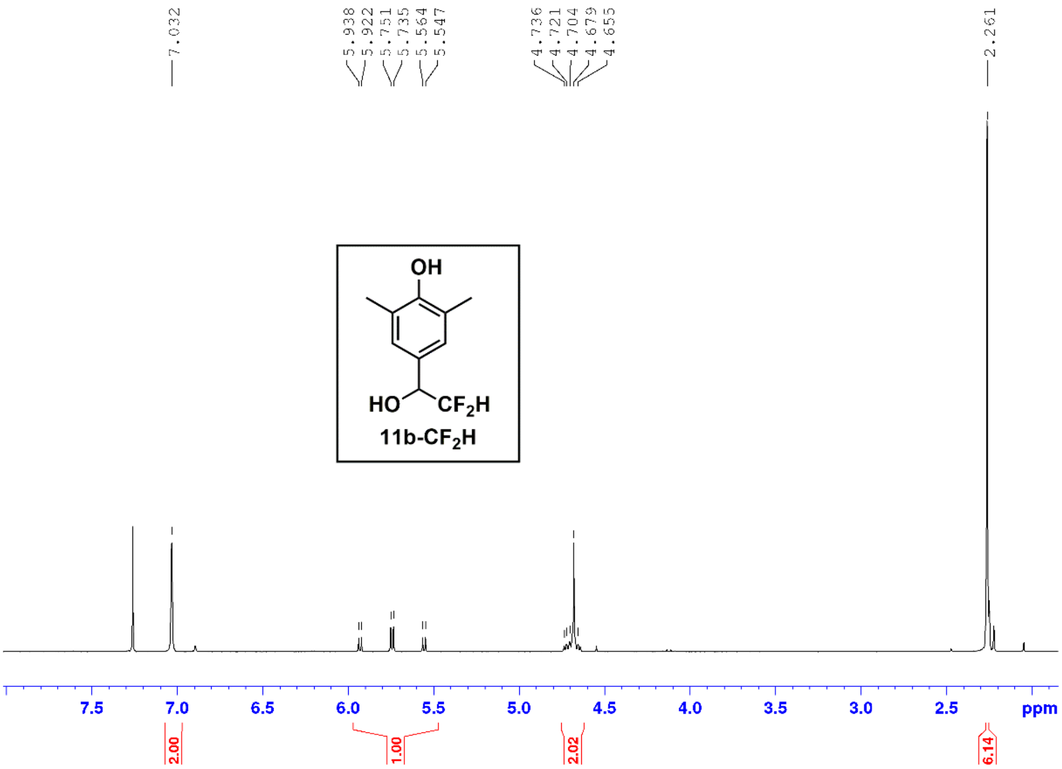

7.032
5.938
5.922
5.751
5.735
5.564
5.547
4.736
4.721
4.704
4.679
4.655
2.261
OH
HO
CF2H
11b-CF2H
7.5
7.0
6.5
6.0
5.5
5.0
4.5
4.0
3.5
3.0
2.5
ppm
2.00
1.00
2.02
6.14

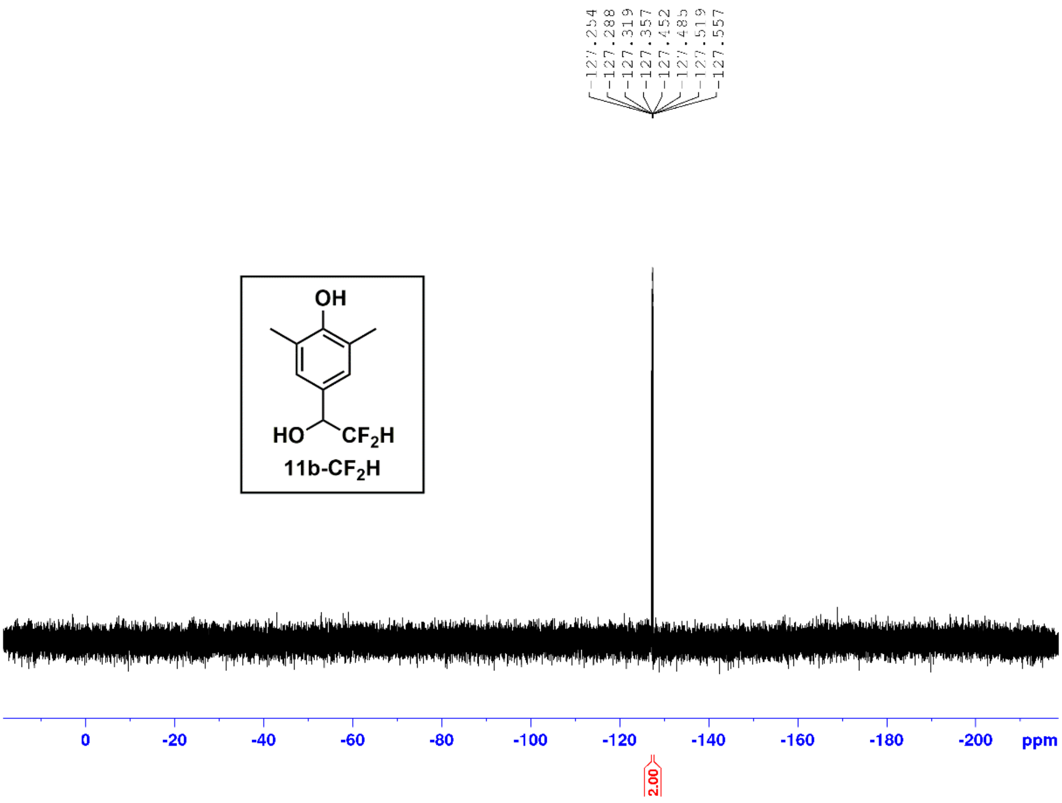

-127.254
-127.288
-127.319
-127.357
-127.452
-127.485
-127.519
-127.557
OH
HO
CF2H
11b-CF2H
0
-20
-40
-60
-80
-100
-120
-140
-160
-180
-200
ppm
2.00

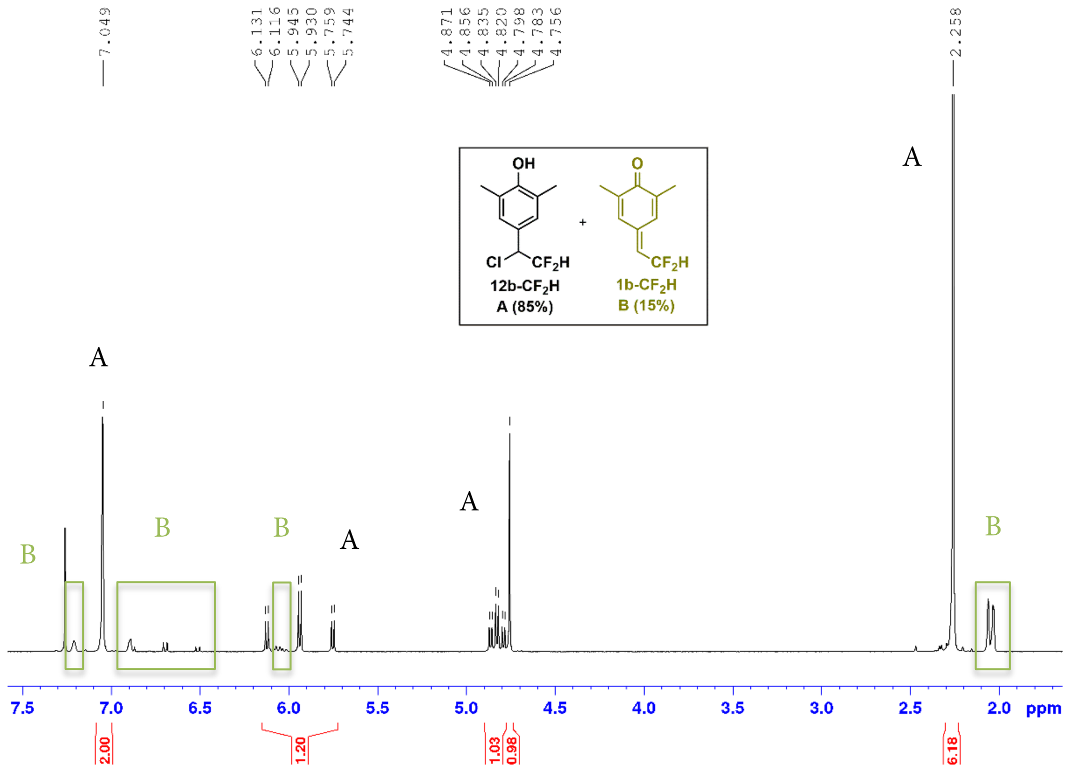
7.049
6.131
6.116
5.945
5.930
5.759
5.744
4.871
4.856
4.835
4.820
4.798
4.783
4.756
2.258
OH
Cl
CF2H
12b-CF2H
A (85%)
+
O
CF2H
1b-CF2H
B (15%)
A
B
7.5
7.0
6.5
6.0
5.5
5.0
4.5
4.0
3.5
3.0
2.5
2.0
ppm
2.00
1.20
1.03
0.98
6.18

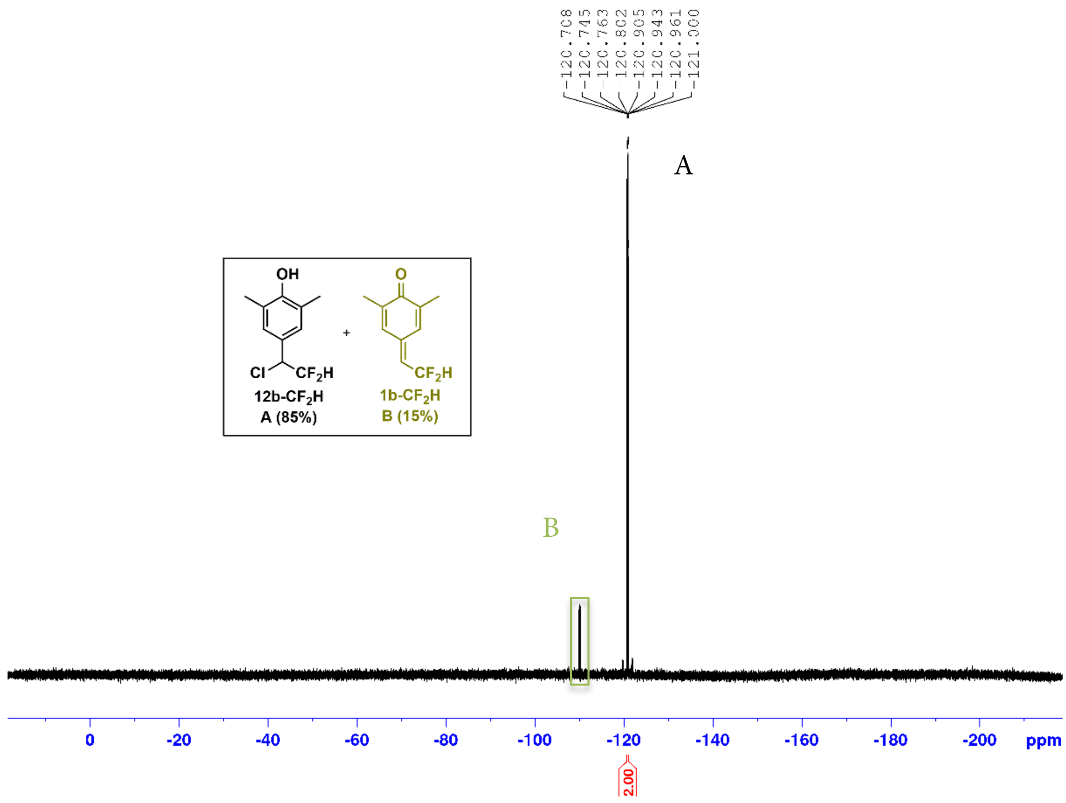
-120.708
-120.745
-120.763
-120.802
-120.905
-120.943
-120.961
-121.000
A
B
OH
Cl
CF2H
12b-CF2H
A (85%)
+
O
CF2H
1b-CF2H
B (15%)
0
-20
-40
-60
-80
-100
-120
-140
-160
-180
-200
ppm
2.00

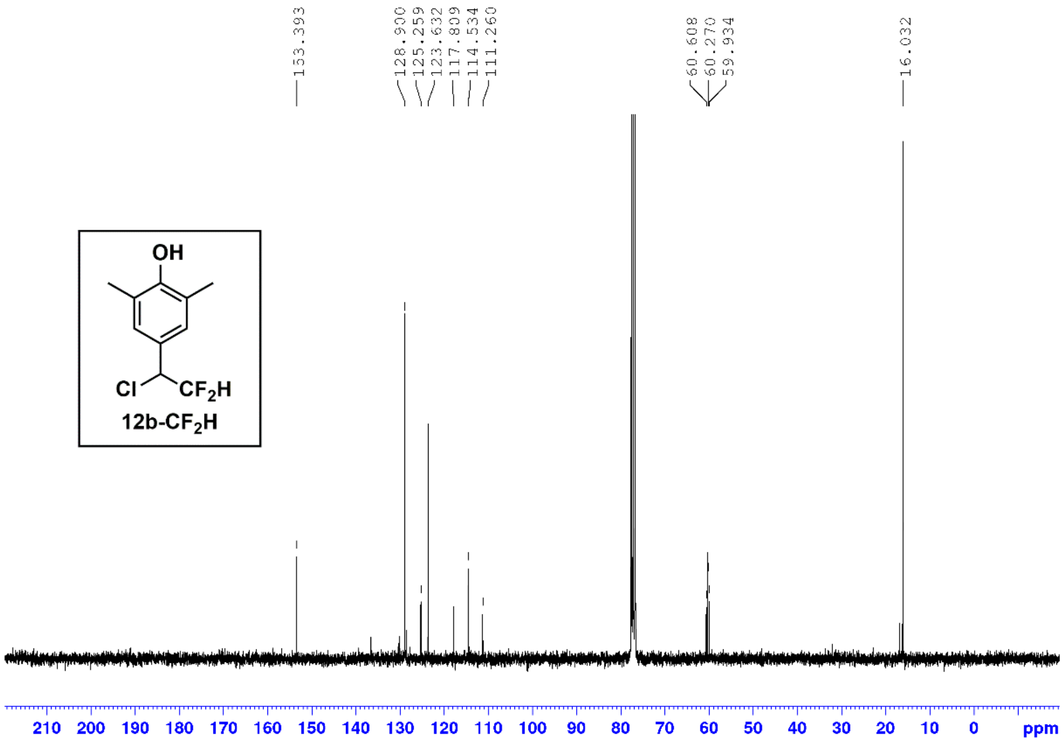

133.393
128.900
125.259
123.632
117.809
114.534
111.260
60.608
60.270
59.934
16.032
OH
Cl
CF2H
12b-CF2H
210 200 190 180 170 160 150 140 130 120 110 100 90 80 70 60 50 40 30 20 10 0 ppm

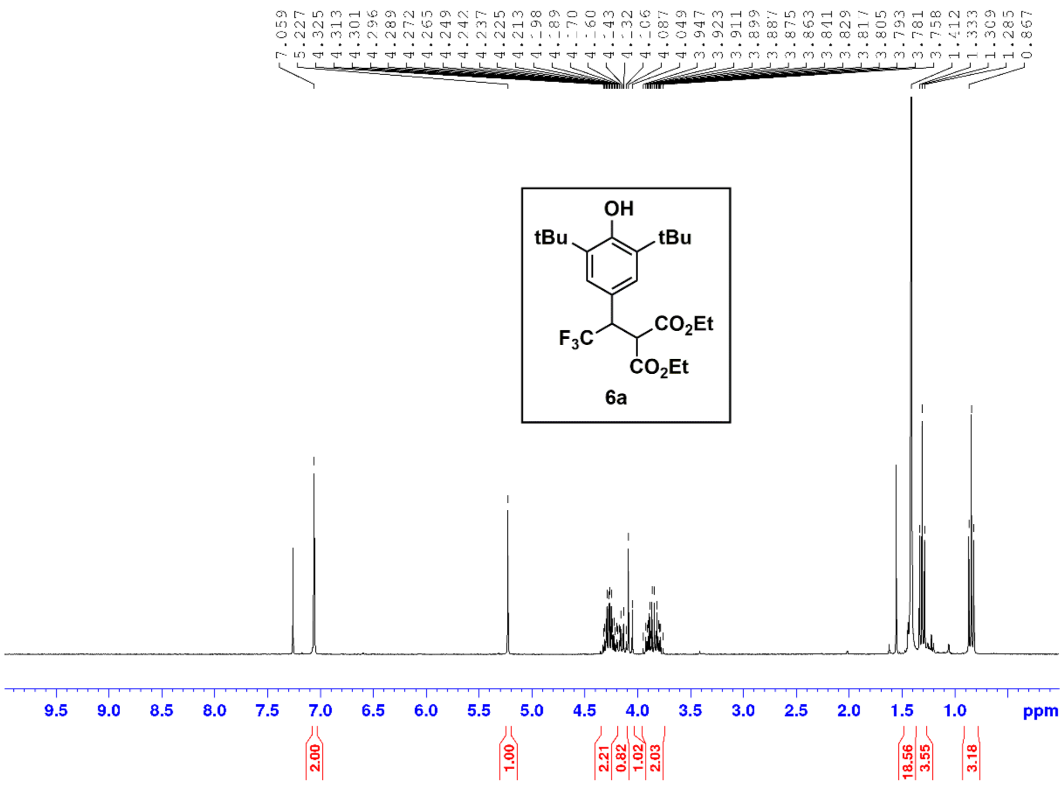

OH
tBu
tBu
CO2Et
F3C
CO2Et
6a
9.5 9.0 8.5 8.0 7.5 7.0 6.5 6.0 5.5 5.0 4.5 4.0 3.5 3.0 2.5 2.0 1.5 1.0 ppm
2.00
1.00
2.21
0.82
1.02
2.03
18.56
3.55
3.18

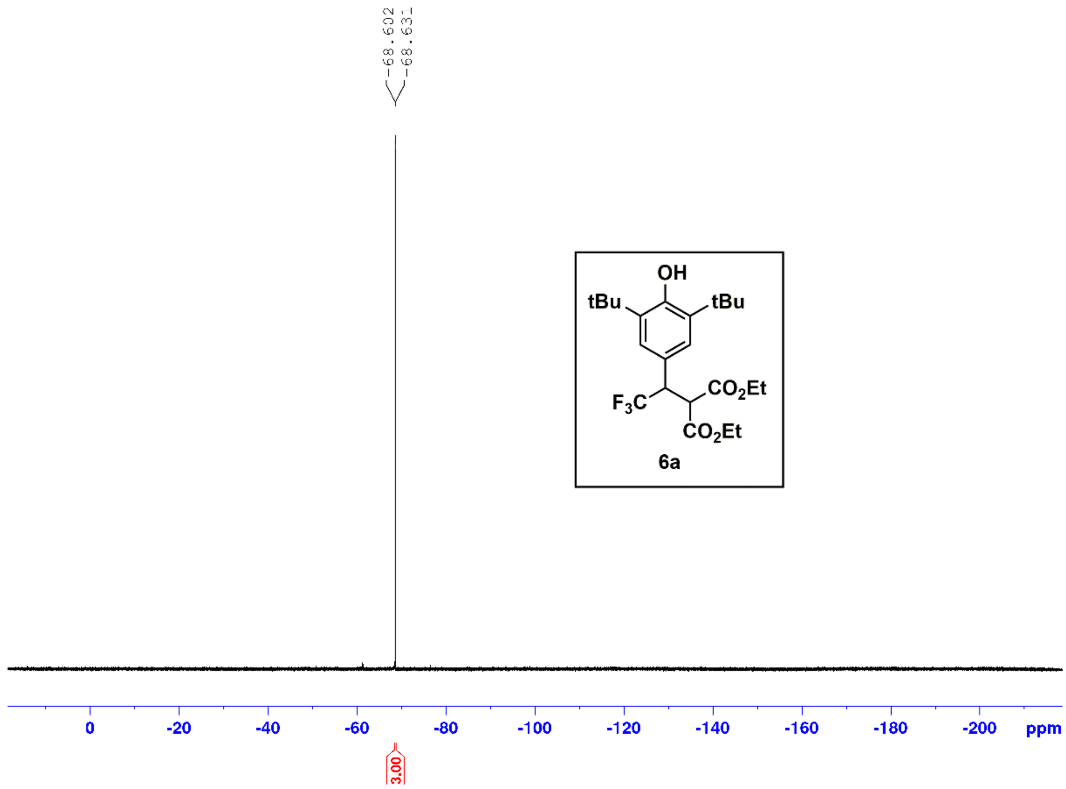
-68.602
-68.631
OH
tBu
tBu
F3C
CO2Et
CO2Et
6a
0 -20 -40 -60 -80 -100 -120 -140 -160 -180 -200 ppm
3.00

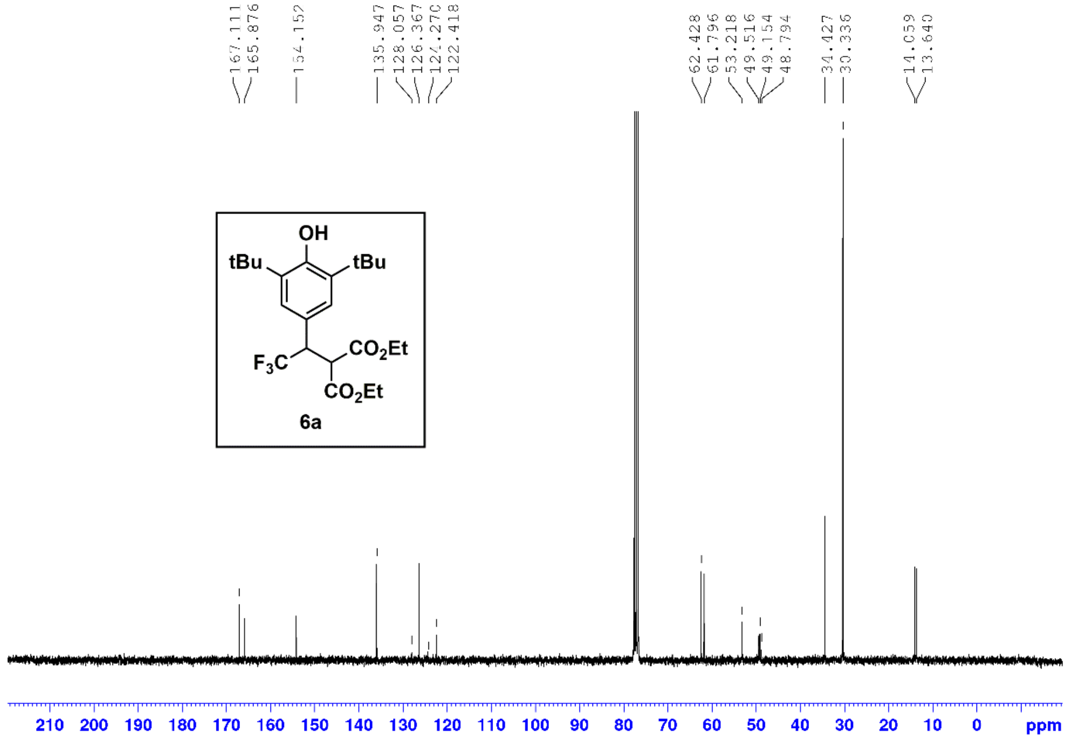
167.111
165.876
154.152
135.947
128.057
126.367
124.270
122.418
62.428
61.796
53.218
49.516
49.154
48.794
34.427
30.336
14.059
13.640
OH
tBu
tBu
F3C
CO2Et
CO2Et
6a
210 200 190 180 170 160 150 140 130 120 110 100 90 80 70 60 50 40 30 20 10 0 ppm

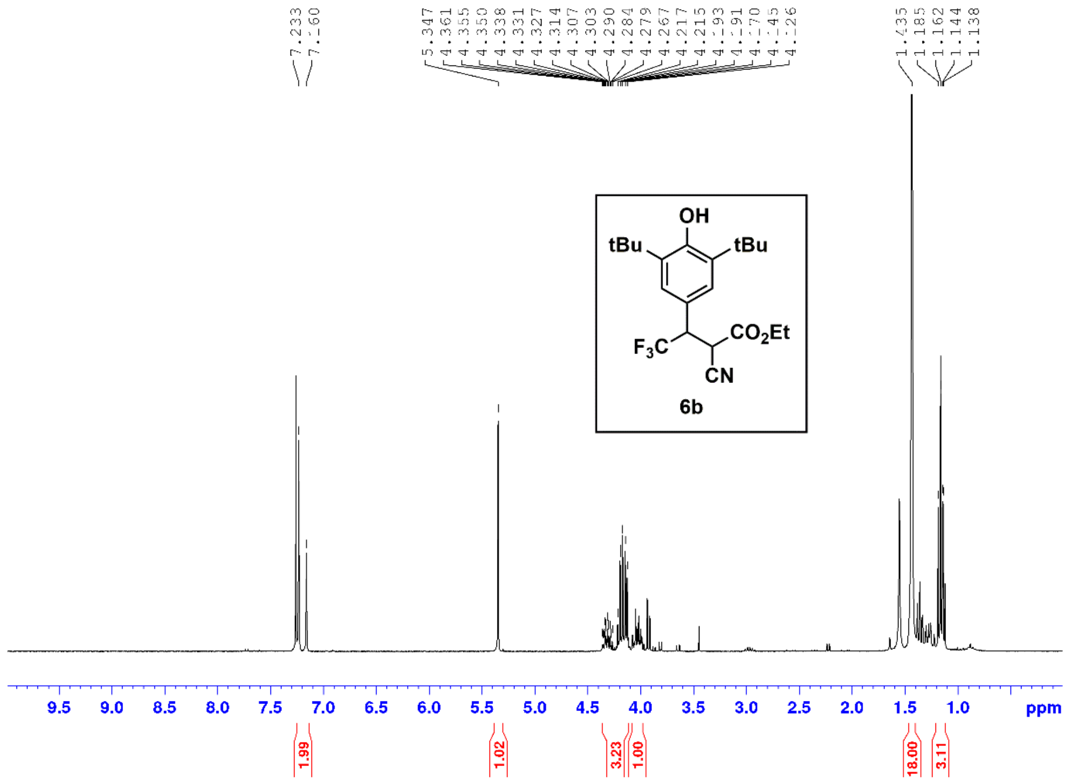

OH
tBu
tBu
F3C
CO2Et
CN
6b
ppm

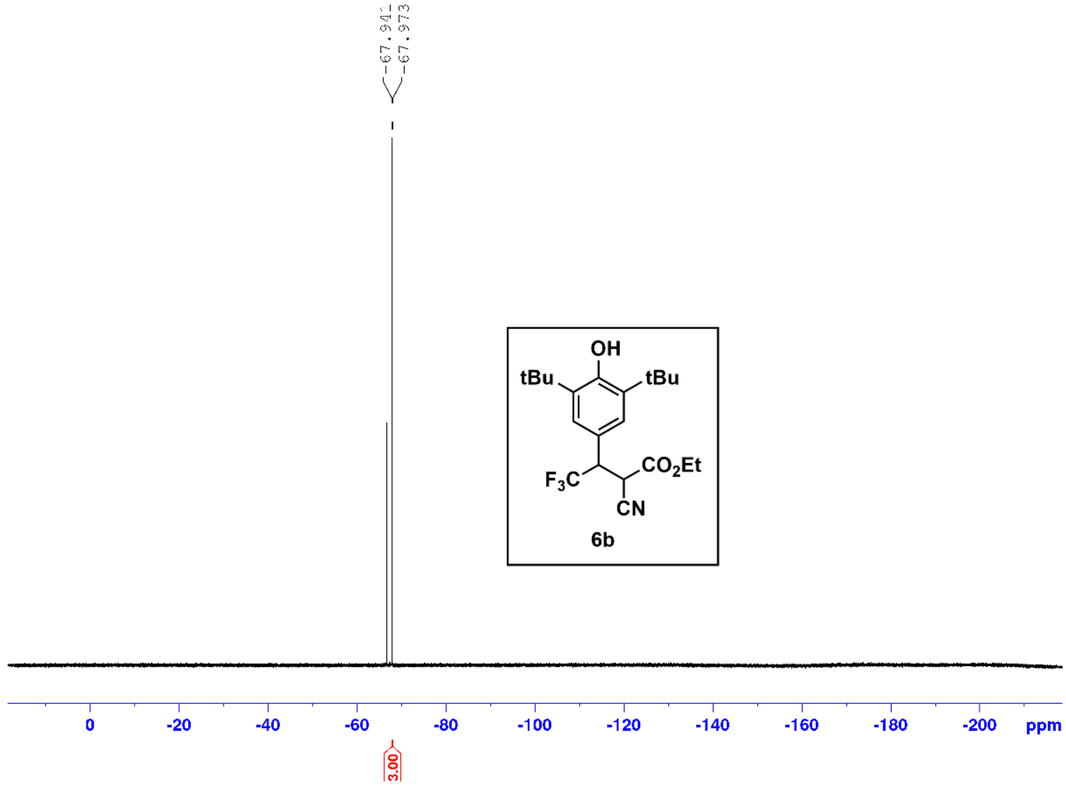

-67.941
-67.973
OH
tBu
tBu
F3C
CO2Et
CN
6b
ppm

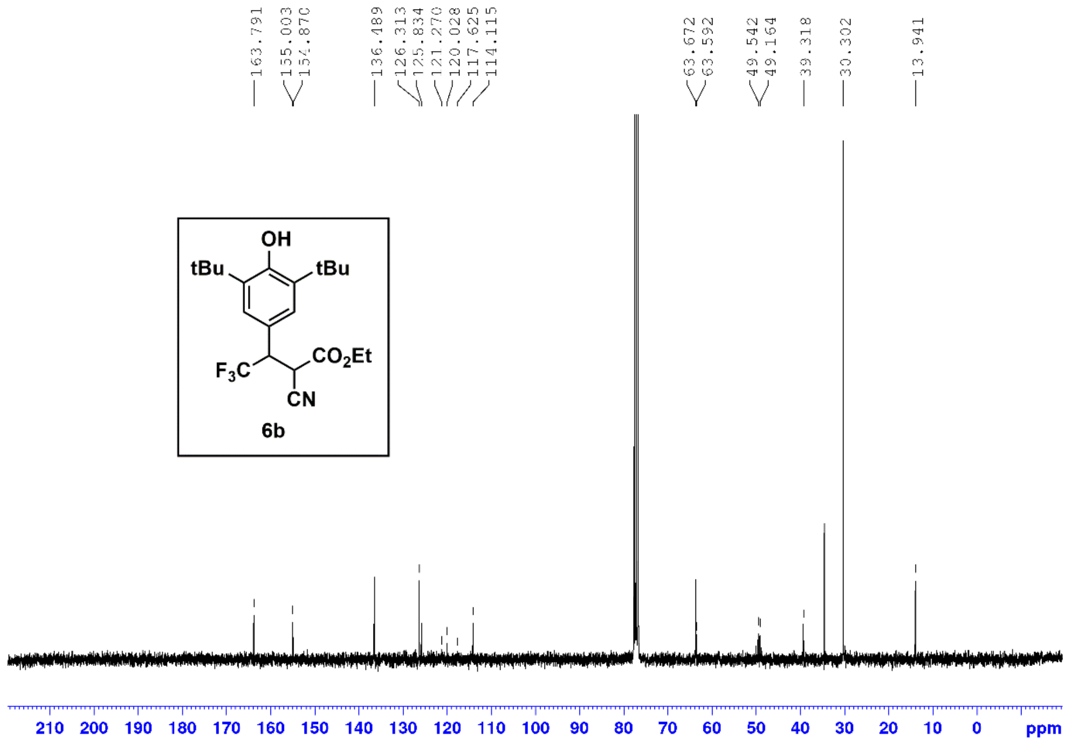
163.791
155.003
154.870
136.489
126.313
125.834
121.270
120.028
117.625
114.115
63.672
63.592
49.542
49.164
39.318
30.302
13.941
OH
tBu
tBu
F3C
CO2Et
CN
6b
210 200 190 180 170 160 150 140 130 120 110 100 90 80 70 60 50 40 30 20 10 0 ppm

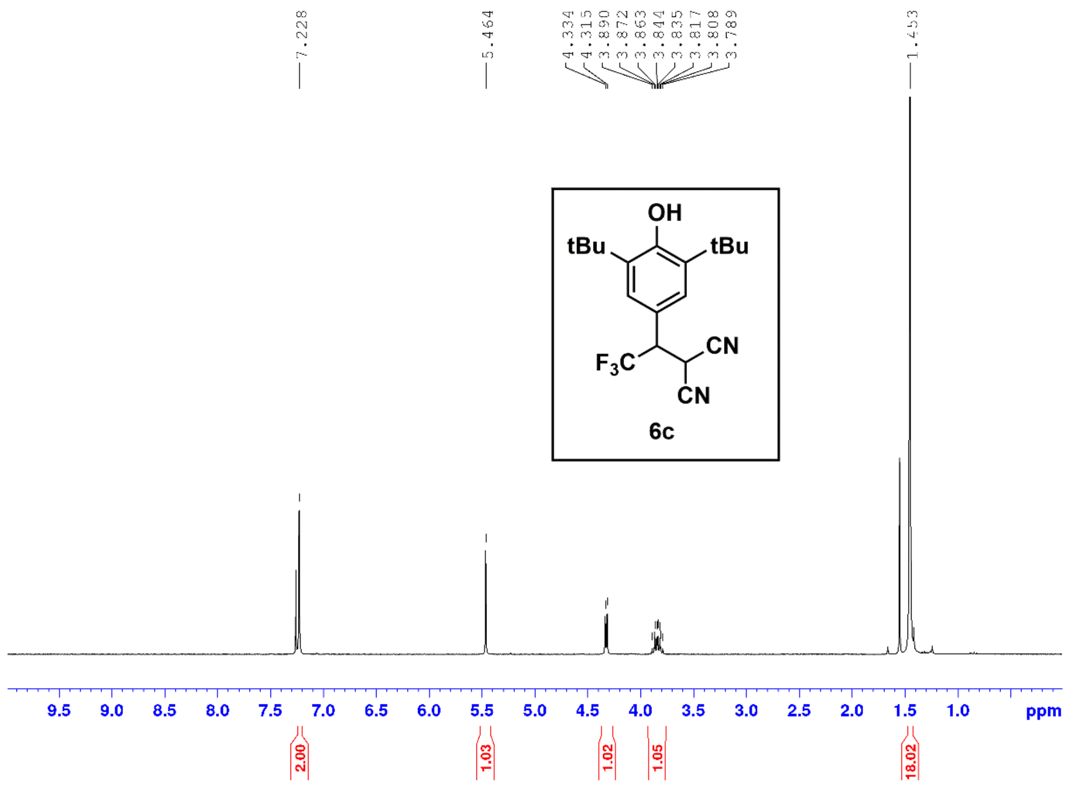
7.228
5.464
4.334
4.315
3.890
3.872
3.863
3.844
3.835
3.817
3.808
3.789
1.453
OH
tBu
tBu
F3C
CN
CN
6c
9.5 9.0 8.5 8.0 7.5 7.0 6.5 6.0 5.5 5.0 4.5 4.0 3.5 3.0 2.5 2.0 1.5 1.0 ppm
2.00
1.03
1.02
1.05
18.02

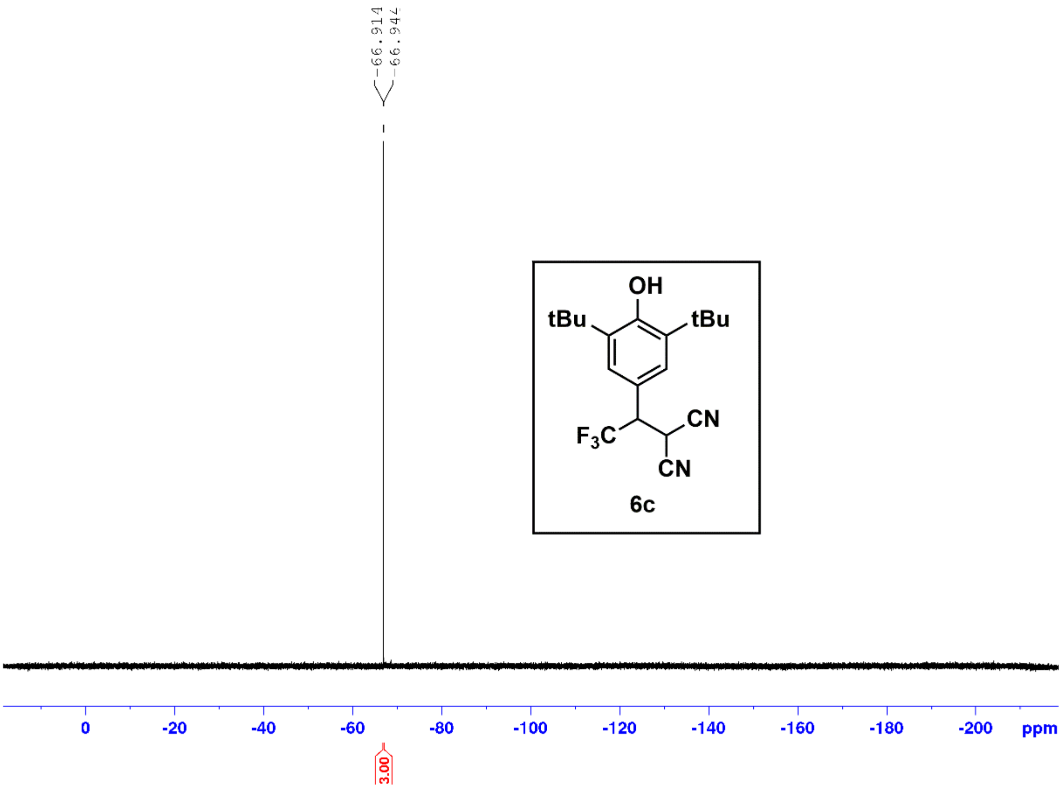

66.914
66.944
OH
tBu
tBu
F3C
CN
CN
6c
0
-20
-40
-60
-80
-100
-120
-140
-160
-180
-200
ppm
3.00

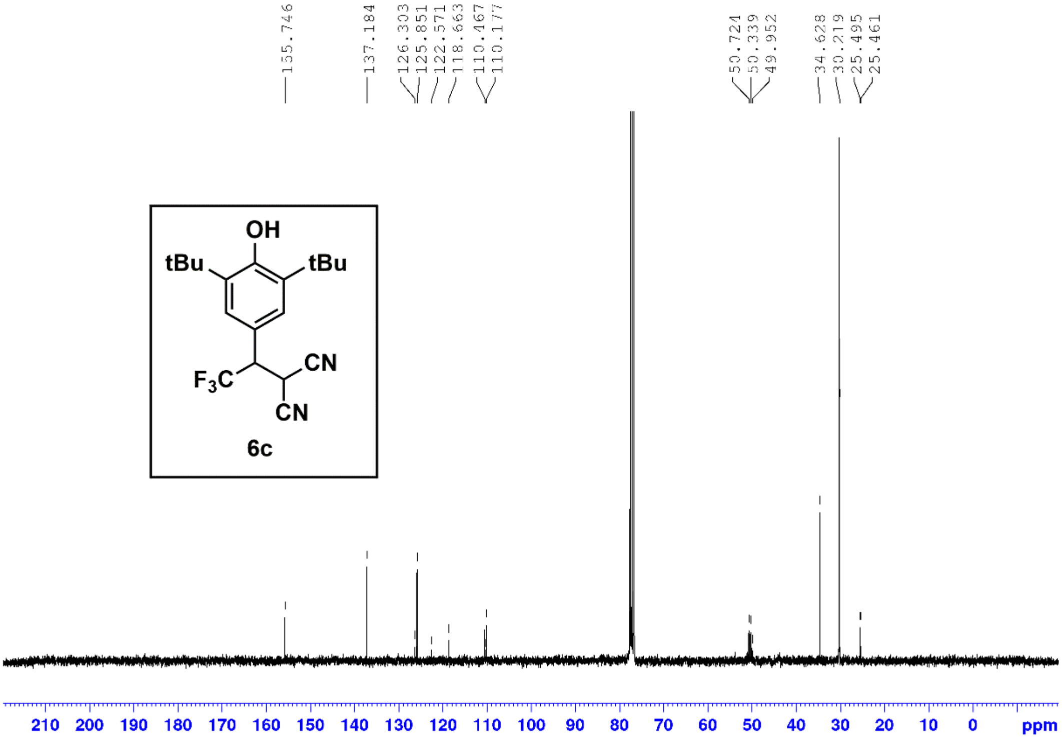

155.746
137.184
126.303
125.851
122.571
118.663
110.467
110.177
50.724
50.339
49.952
34.628
30.219
25.495
25.461
OH
tBu
tBu
F3C
CN
CN
6c
210
200
190
180
170
160
150
140
130
120
110
100
90
80
70
60
50
40
30
20
10
0
ppm

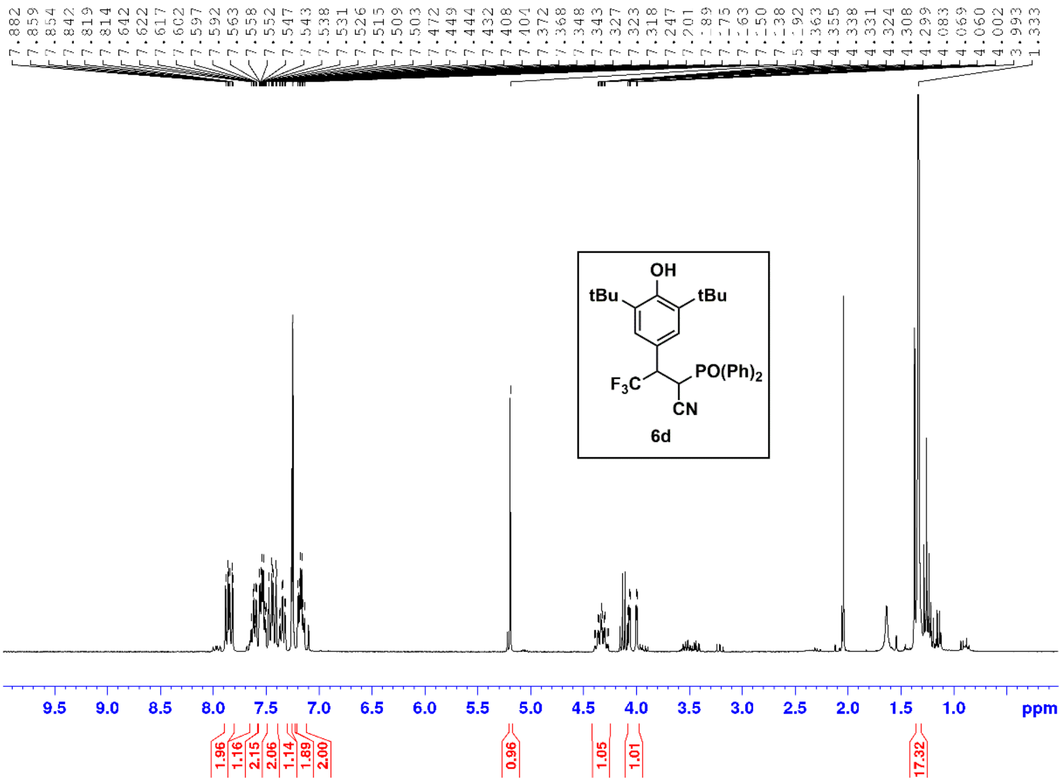

OH
tBu
tBu
F3C
PO(Ph)2
CN
6d
ppm

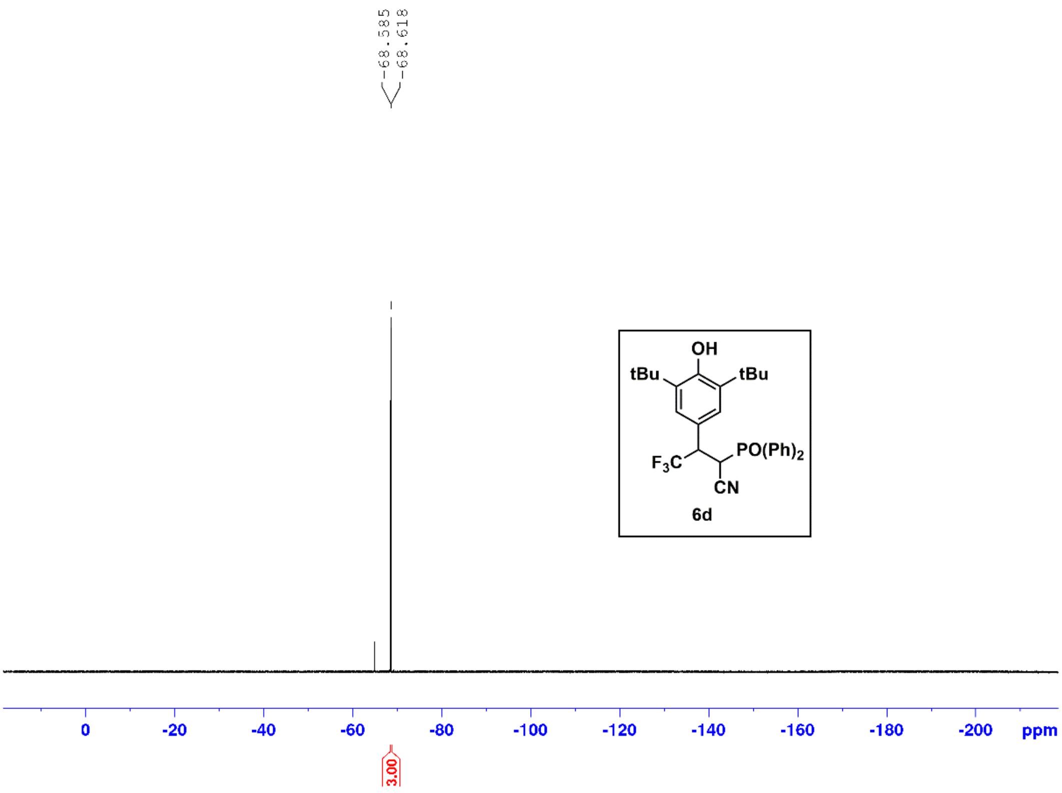

-68.585
-68.618
OH
tBu
tBu
F3C
PO(Ph)2
CN
6d
ppm

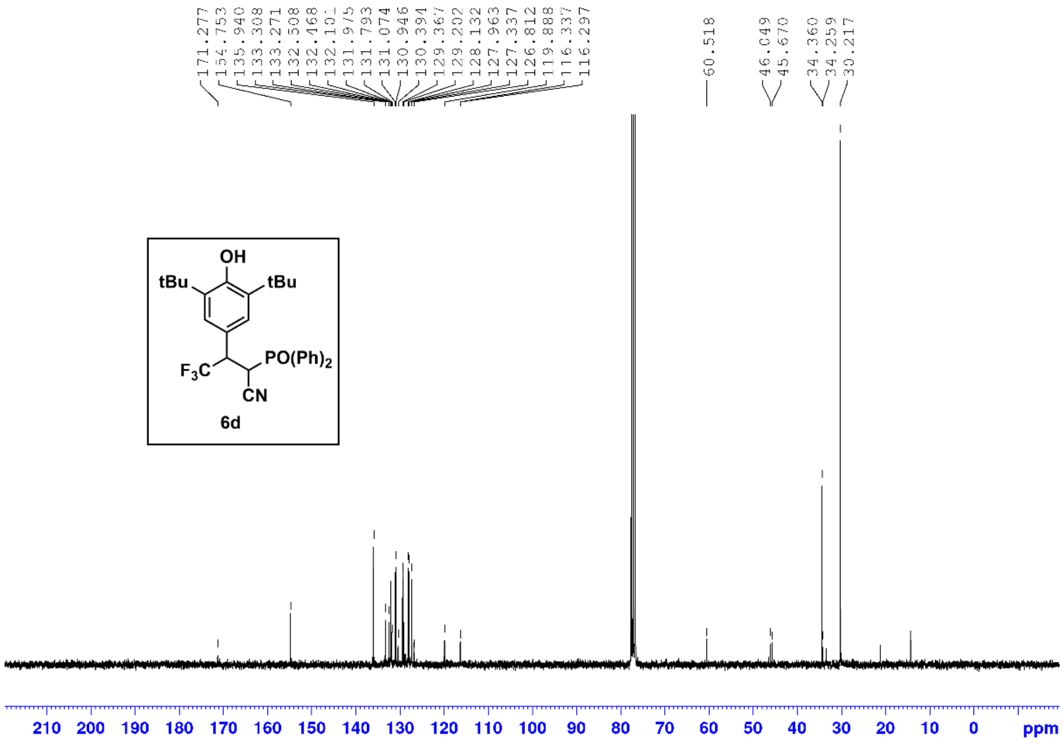
171.277
154.753
135.940
133.308
133.271
132.508
132.468
131.793
131.074
130.946
130.391
129.367
129.202
128.132
127.963
127.337
126.812
119.888
116.337
116.297
60.518
46.049
45.670
34.360
34.259
30.217
OH
tBu
tBu
F_3C
$PO(Ph)_2$
CN
6d
210 200 190 180 170 160 150 140 130 120 110 100 90 80 70 60 50 40 30 20 10 0 ppm

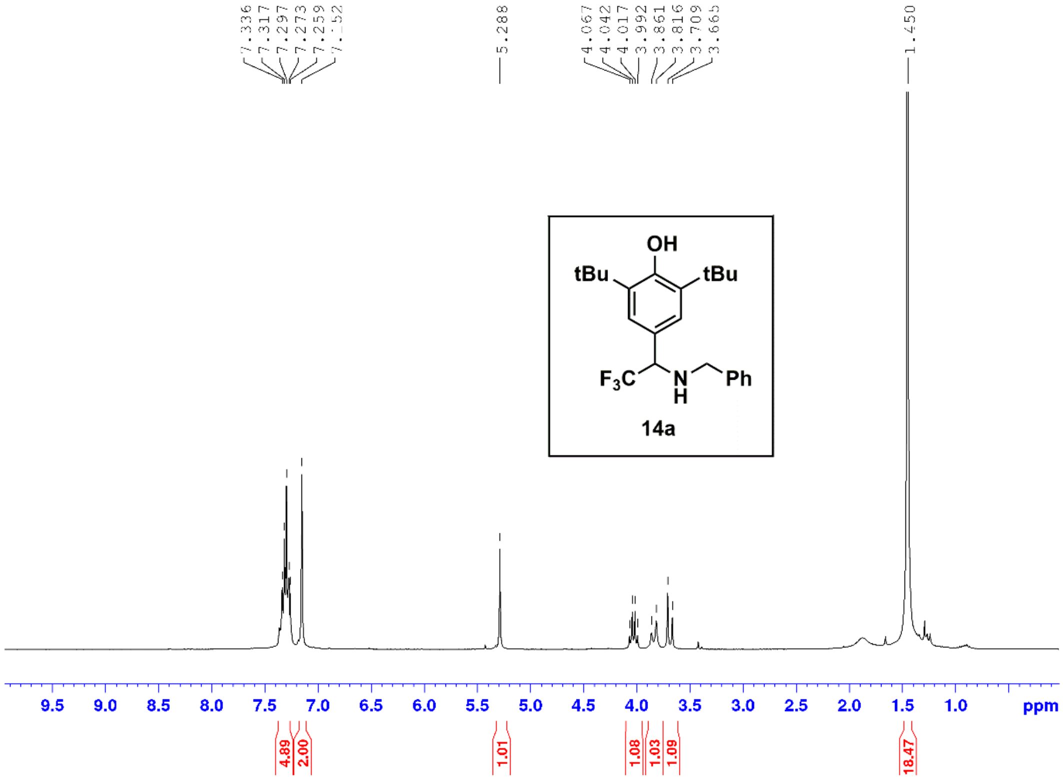
7.336
7.317
7.297
7.273
7.259
5.288
4.067
4.042
4.017
3.992
3.861
3.816
3.709
3.665
1.450
OH
tBu
tBu
F_3C
N
H
Ph
14a
9.5 9.0 8.5 8.0 7.5 7.0 6.5 6.0 5.5 5.0 4.5 4.0 3.5 3.0 2.5 2.0 1.5 1.0 ppm
4.89
2.00
1.01
1.08
1.03
1.09
18.47

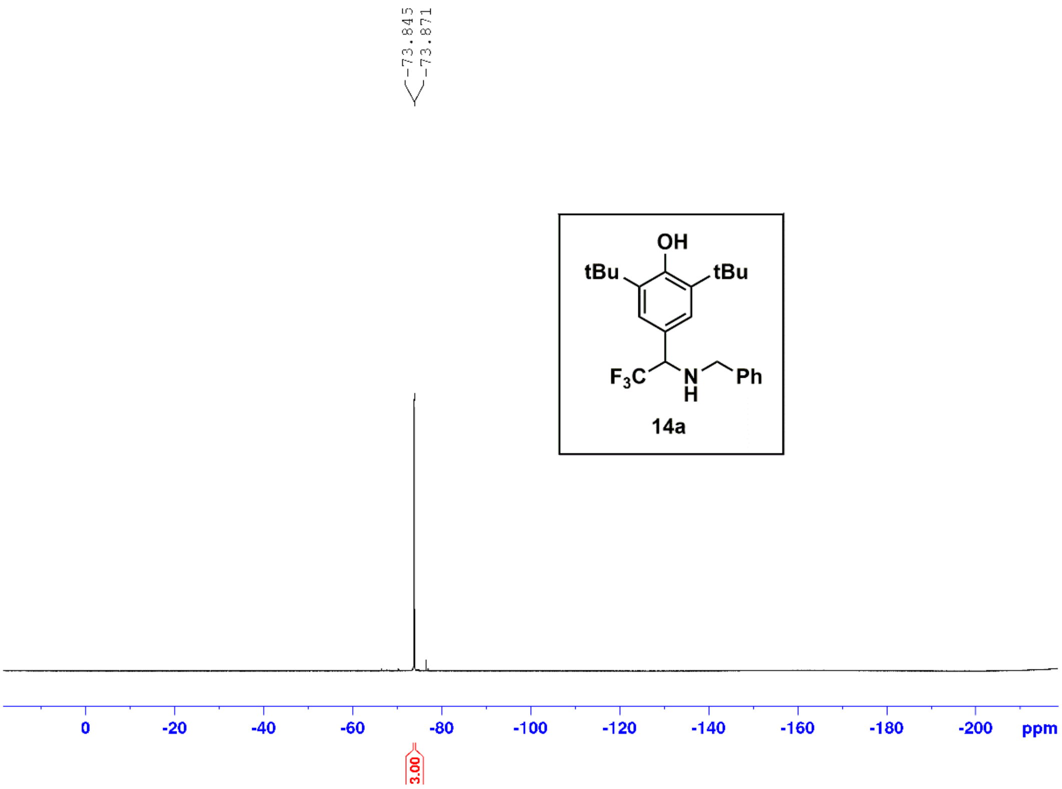
-73.845
-73.871
OH
tBu
tBu
F3C
N
H
Ph
14a
0
-20
-40
-60
-80
-100
-120
-140
-160
-180
-200
ppm
3.00

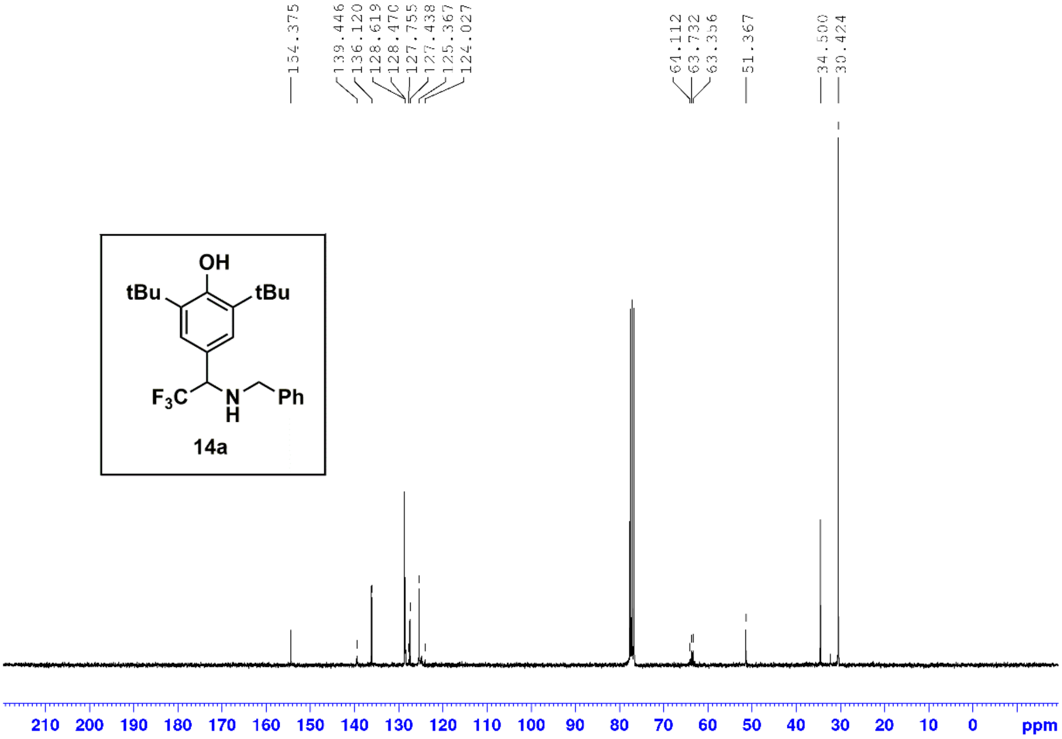
154.375
139.446
136.120
128.619
128.470
127.755
127.438
125.367
124.027
64.112
63.732
63.356
51.367
34.500
30.424
OH
tBu
tBu
F3C
N
H
Ph
14a
210
200
190
180
170
160
150
140
130
120
110
100
90
80
70
60
50
40
30
20
10
0
ppm

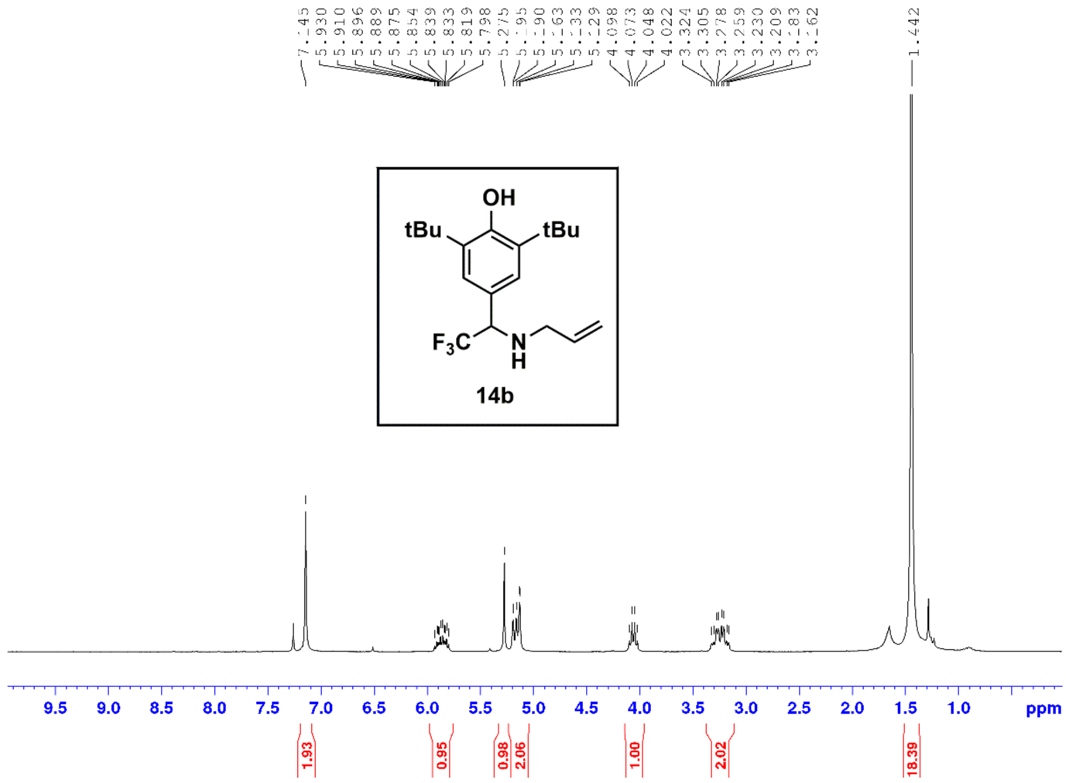
OH
tBu
tBu
F3C
N
H
14b
9.5
9.0
8.5
8.0
7.5
7.0
6.5
6.0
5.5
5.0
4.5
4.0
3.5
3.0
2.5
2.0
1.5
1.0
ppm
1.93
0.95
0.98
2.06
1.00
2.02
18.39
1.442

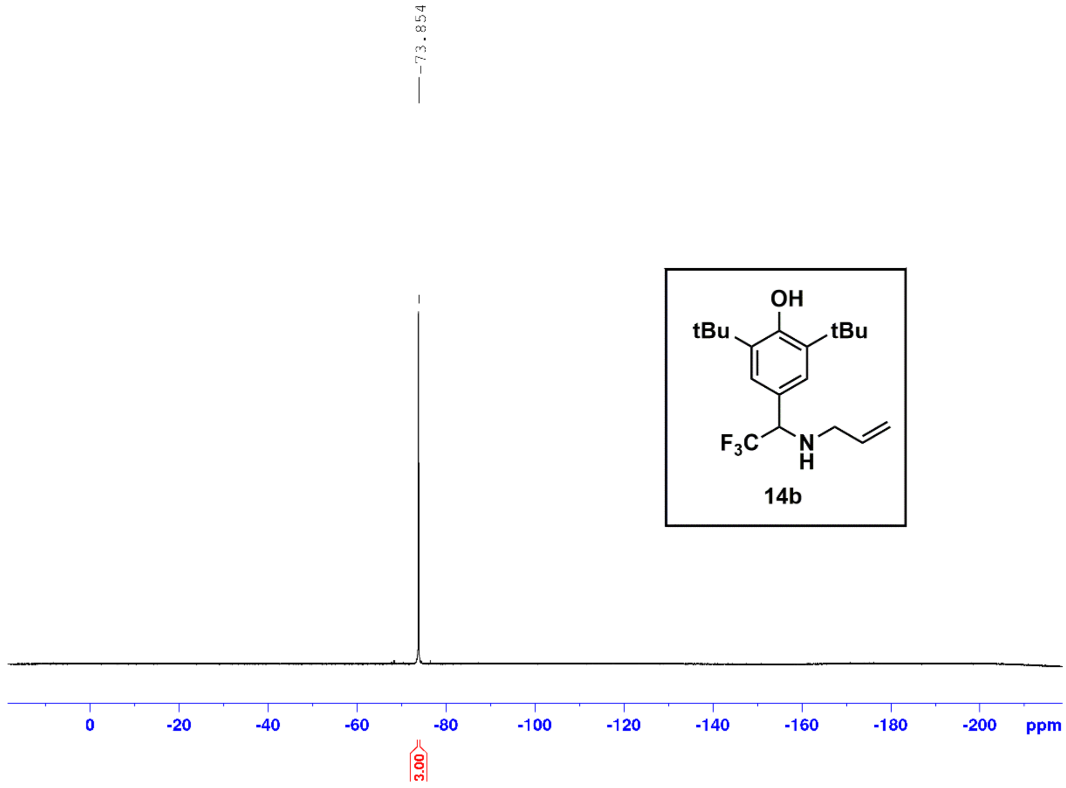
-73.854
OH
tBu
tBu
F3C
N
H
14b
0
-20
-40
-60
-80
-100
-120
-140
-160
-180
-200
ppm
3.00

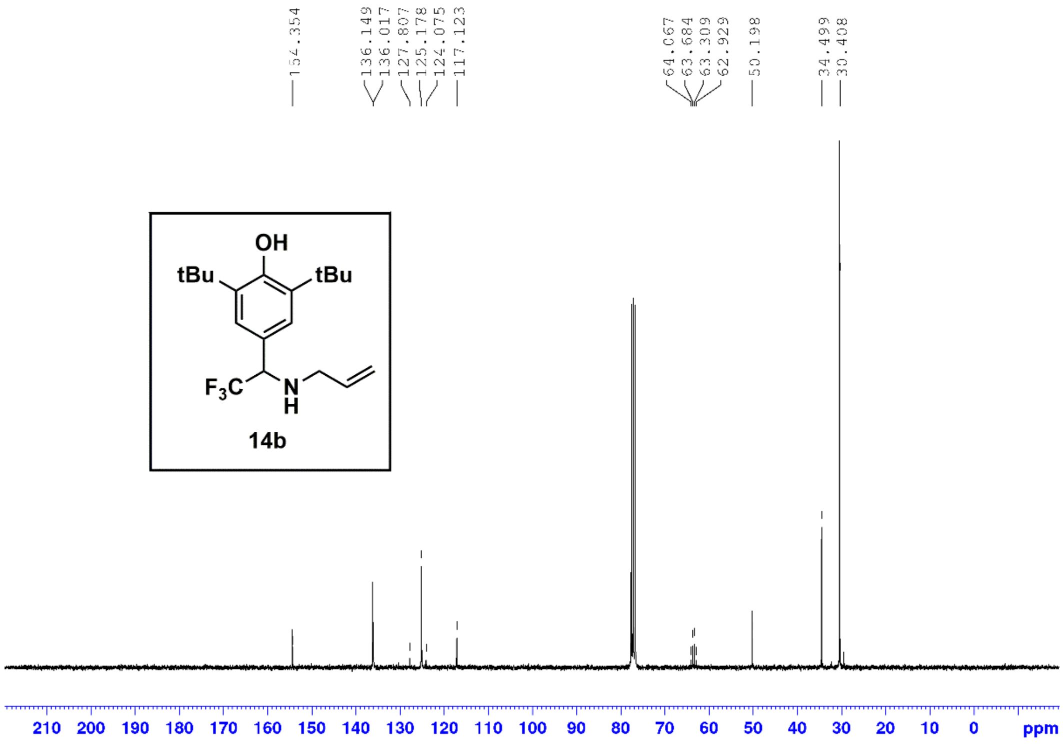
136.149
136.017
127.807
125.178
124.075
117.123
64.067
63.684
63.309
62.929
50.198
34.499
30.408
OH
tBu
tBu
F3C
N
H
14b
210 200 190 180 170 160 150 140 130 120 110 100 90 80 70 60 50 40 30 20 10 0 ppm

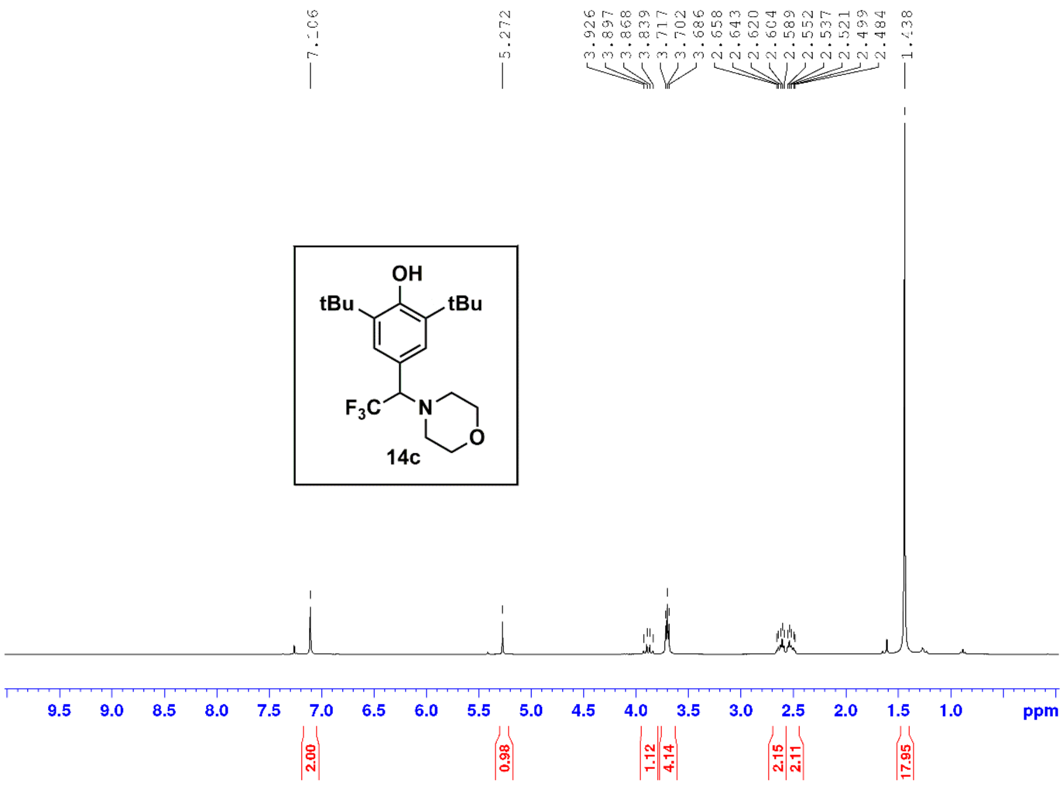
5.272
3.926
3.897
3.868
3.839
3.717
3.702
3.686
2.658
2.643
2.620
2.604
2.589
2.552
2.537
2.521
2.499
2.484
OH
tBu
tBu
F3C
N
O
14c
9.5 9.0 8.5 8.0 7.5 7.0 6.5 6.0 5.5 5.0 4.5 4.0 3.5 3.0 2.5 2.0 1.5 1.0 ppm
2.00
0.98
1.12
4.14
2.15
2.11
17.95

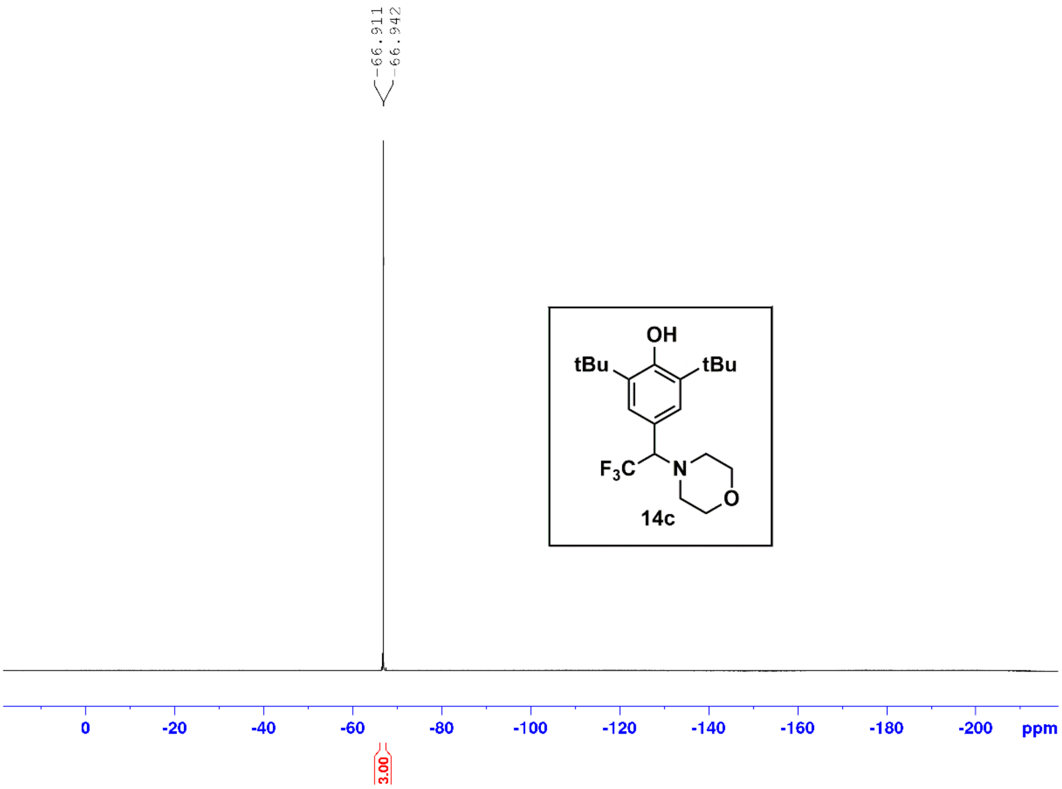
-66.911
-66.942
OH
tBu
tBu
F3C
N
O
14c
0
-20
-40
-60
-80
-100
-120
-140
-160
-180
-200
ppm
3.00

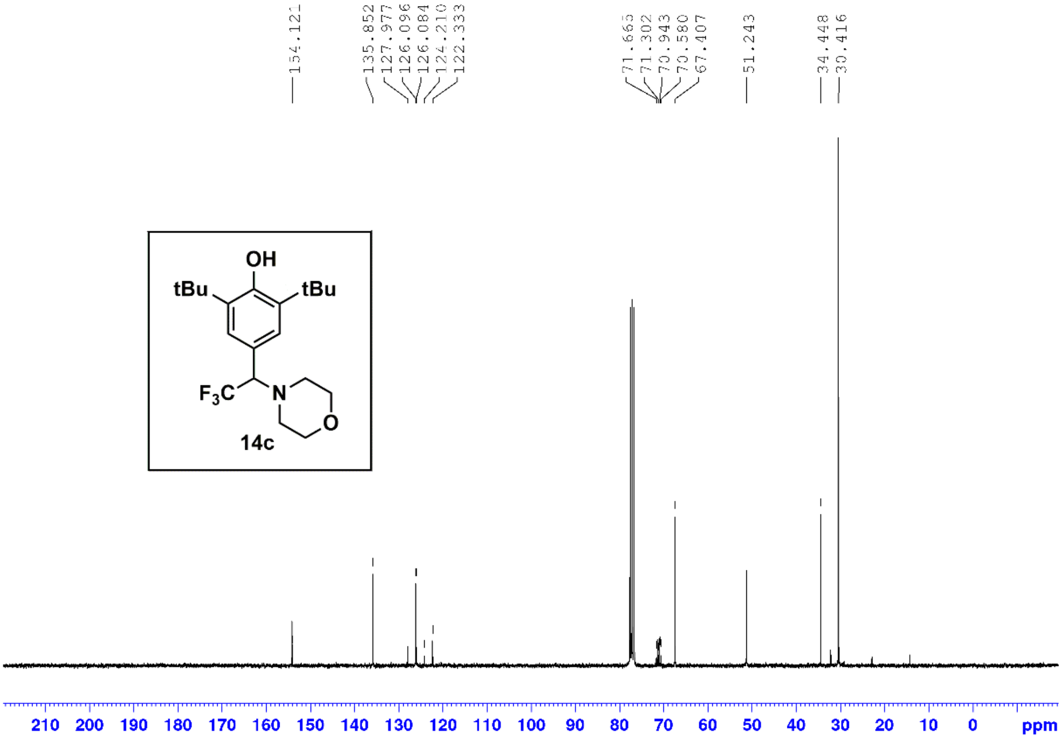
154.121
135.852
127.977
126.096
126.084
124.210
122.333
71.663
71.302
70.943
70.580
67.407
51.243
34.448
30.416
OH
tBu
tBu
F3C
N
O
14c
210
200
190
180
170
160
150
140
130
120
110
100
90
80
70
60
50
40
30
20
10
0
ppm

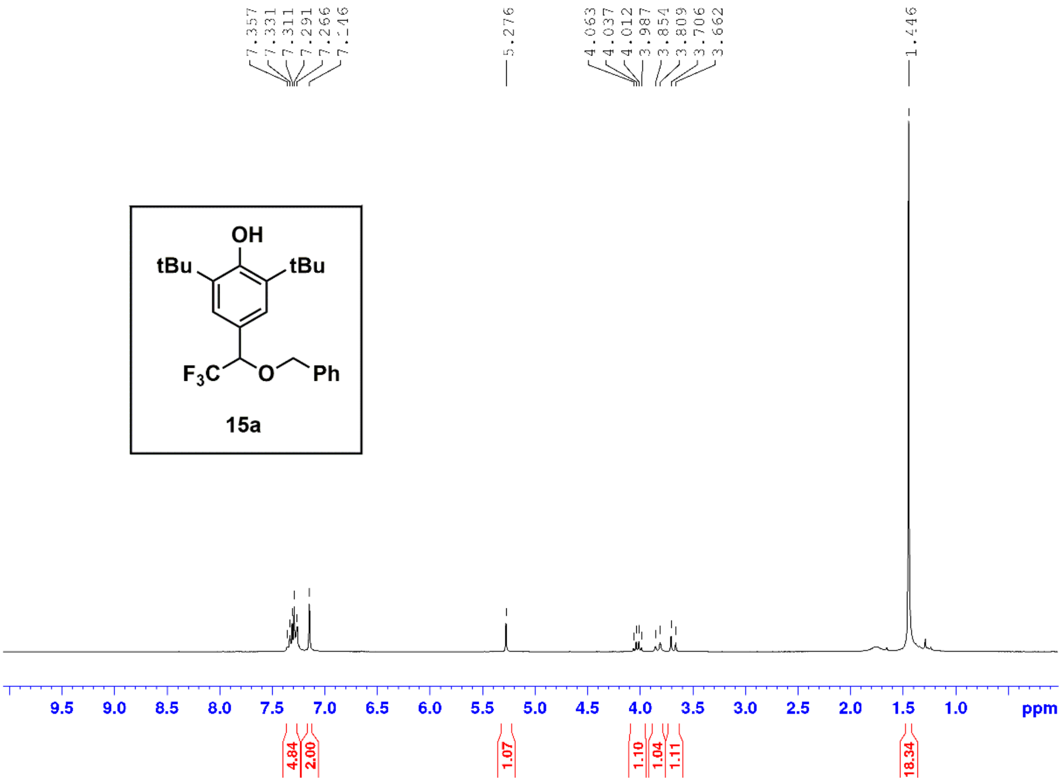
7.357
7.331
7.311
7.291
7.266
7.146
5.276
4.063
4.037
4.012
3.987
3.854
3.809
3.706
3.662
1.446
OH
tBu
tBu
F3C
O
Ph
15a
9.5
9.0
8.5
8.0
7.5
7.0
6.5
6.0
5.5
5.0
4.5
4.0
3.5
3.0
2.5
2.0
1.5
1.0
ppm
4.84
2.00
1.07
1.10
1.04
1.11
18.34

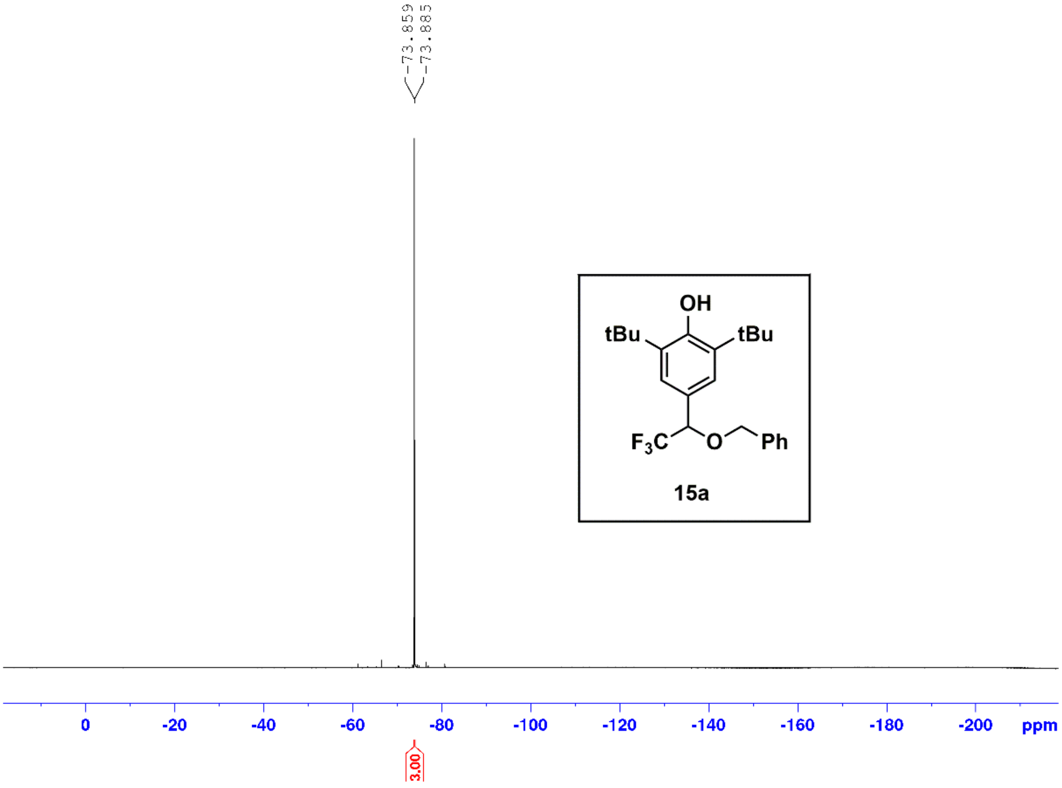
-73.859
-73.885
OH
tBu
tBu
F3C
O
Ph
15a
0
-20
-40
-60
-80
-100
-120
-140
-160
-180
-200
ppm
3.00

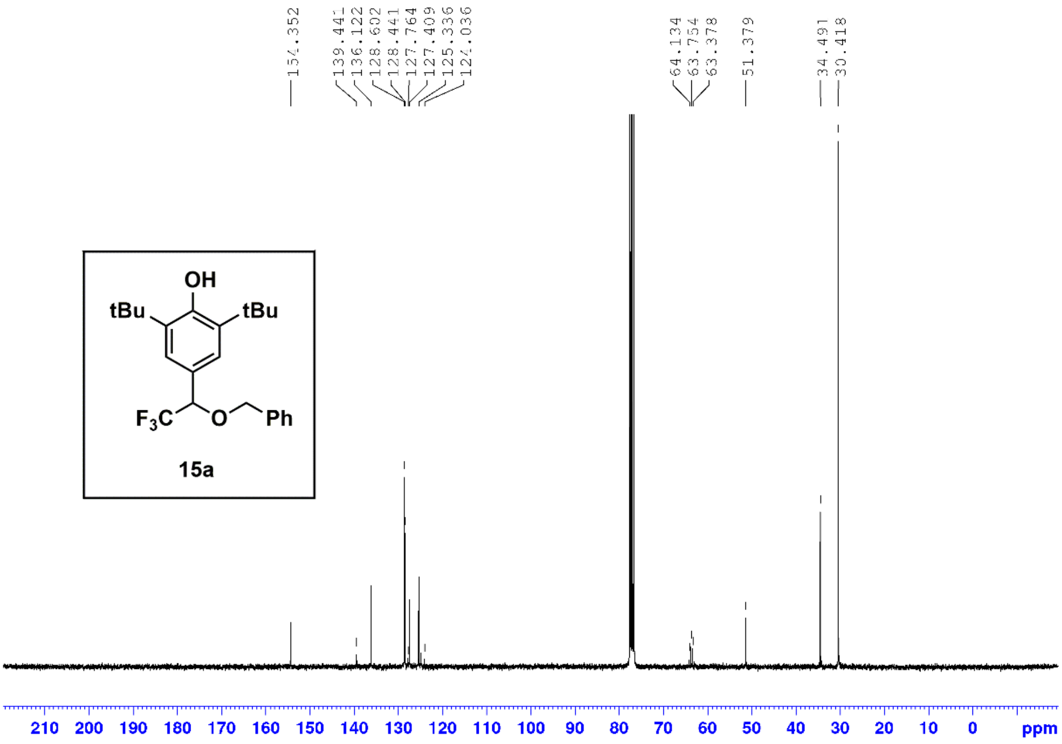
OH
tBu
tBu
F3C
O
Ph
15a
136.122
128.602
128.441
127.764
127.409
125.336
64.134
63.754
63.378
51.379
34.491
210 200 190 180 170 160 150 140 130 120 110 100 90 80 70 60 50 40 30 20 10 0 ppm

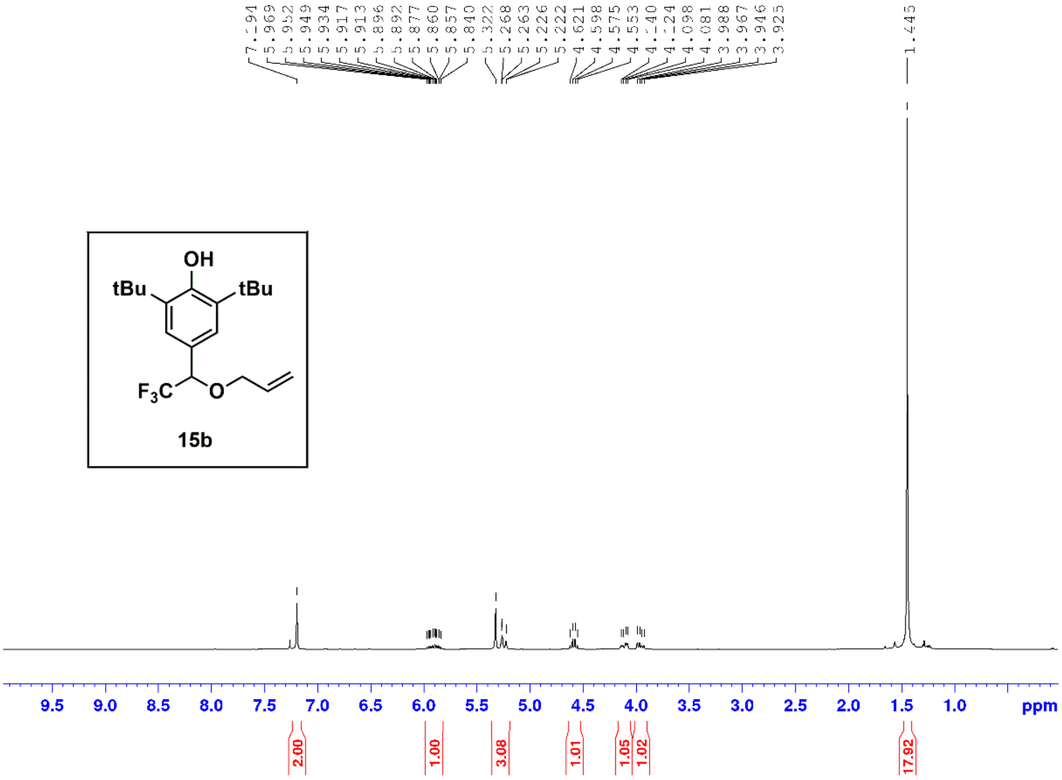
OH
tBu
tBu
F3C
O
15b
5.969
5.949
5.934
5.917
5.913
5.896
5.892
5.877
5.860
5.857
5.840
5.322
5.268
5.263
5.226
5.222
4.621
4.598
4.575
4.553
4.098
4.081
3.988
3.967
3.946
3.925
1.445
9.5 9.0 8.5 8.0 7.5 7.0 6.5 6.0 5.5 5.0 4.5 4.0 3.5 3.0 2.5 2.0 1.5 1.0 ppm
2.00
1.00
3.08
1.01
1.05
1.02
17.92

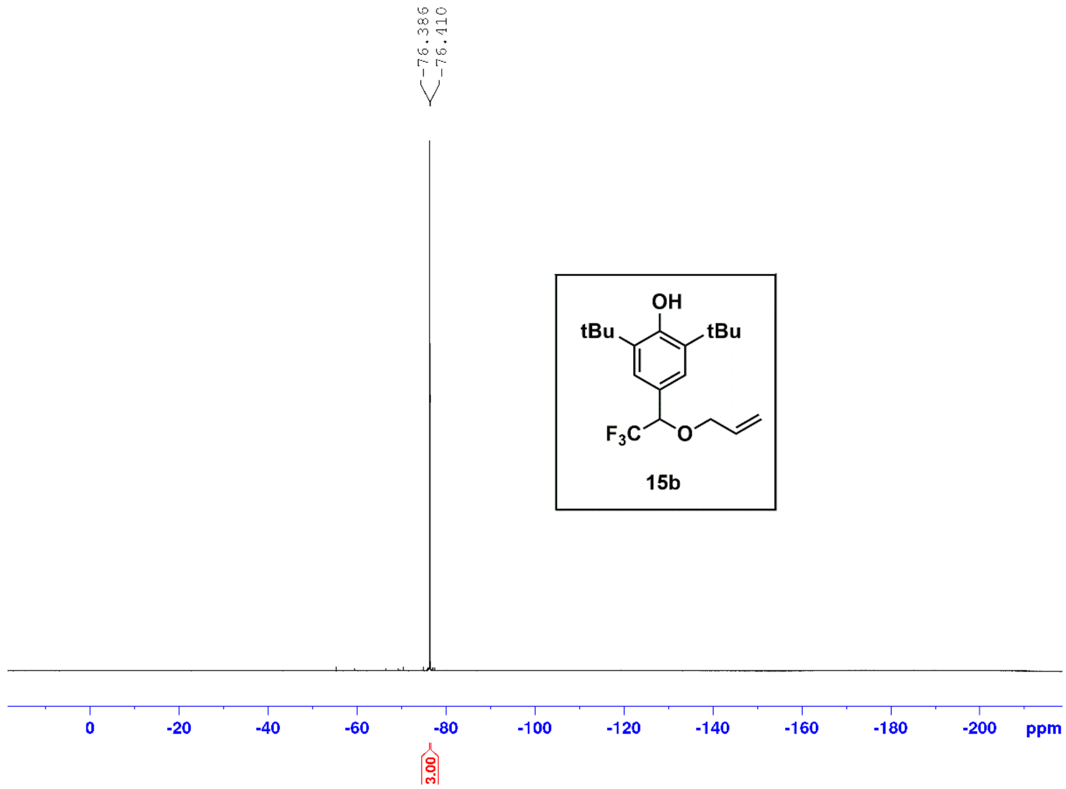
-76.386
-76.410
OH
tBu
tBu
F3C
O
15b
0
-20
-40
-60
-80
-100
-120
-140
-160
-180
-200
ppm
3.00

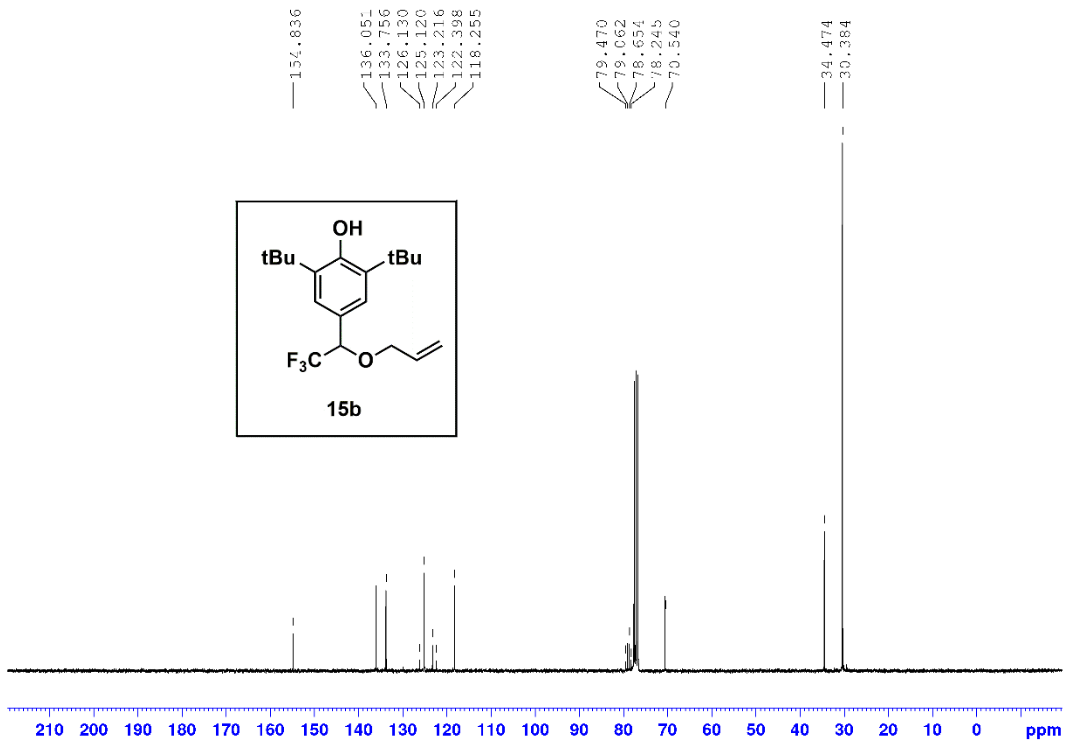
154.836
136.051
133.756
126.130
125.120
123.216
122.398
118.255
79.470
79.062
78.654
78.245
70.540
34.474
30.384
OH
tBu
tBu
F3C
O
15b
210
200
190
180
170
160
150
140
130
120
110
100
90
80
70
60
50
40
30
20
10
0
ppm

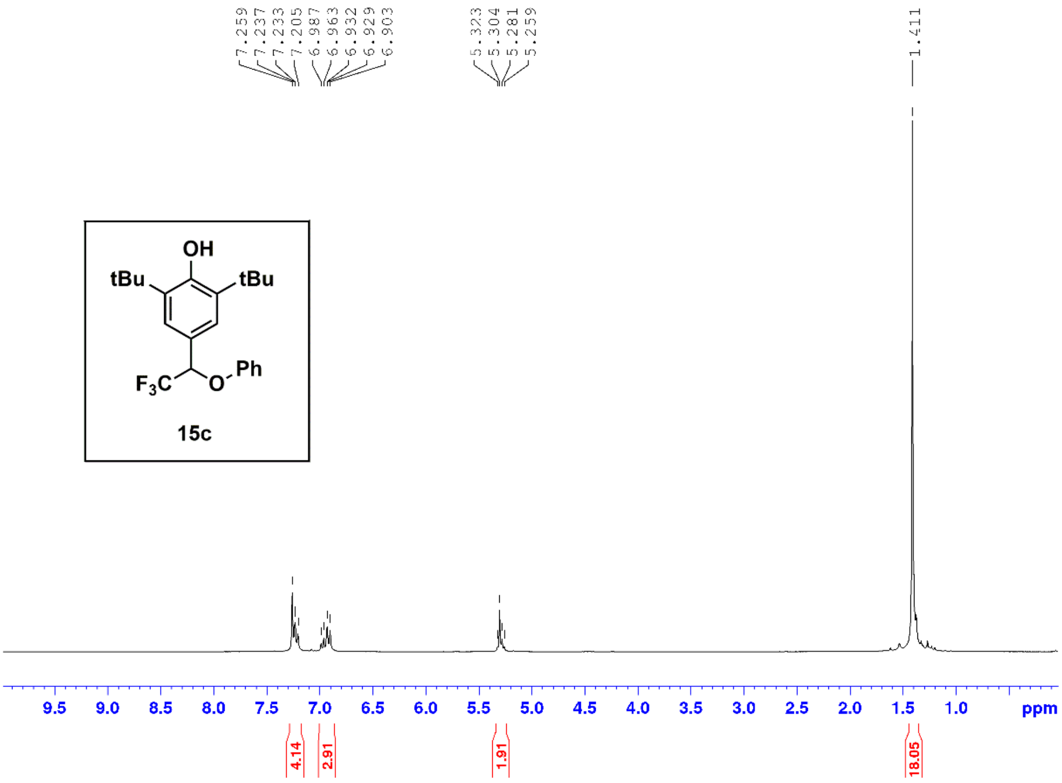
7.259
7.237
7.233
7.205
6.987
6.963
6.932
6.929
6.903
5.323
5.304
5.281
5.259
1.411
OH
tBu
tBu
F_3C
O
Ph
15c
9.5 9.0 8.5 8.0 7.5 7.0 6.5 6.0 5.5 5.0 4.5 4.0 3.5 3.0 2.5 2.0 1.5 1.0 ppm
4.14
2.91
1.91
18.05

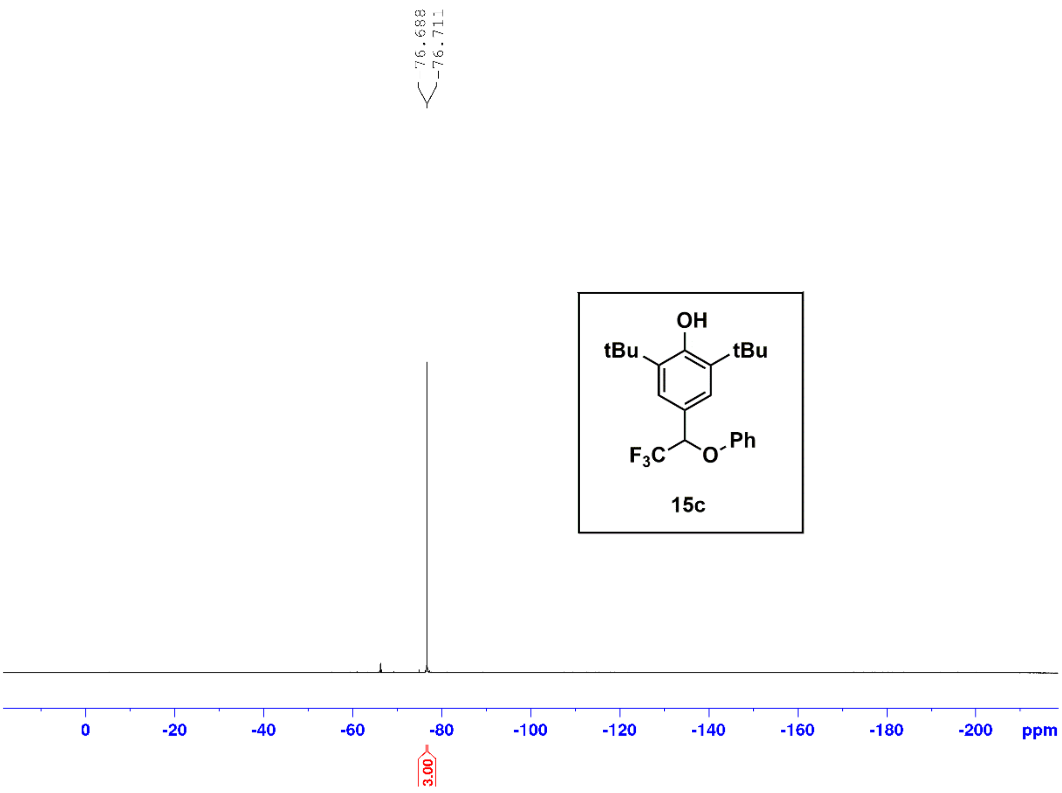
76.688
76.711
OH
tBu
tBu
F_3C
O
Ph
15c
0 -20 -40 -60 -80 -100 -120 -140 -160 -180 -200 ppm
3.00

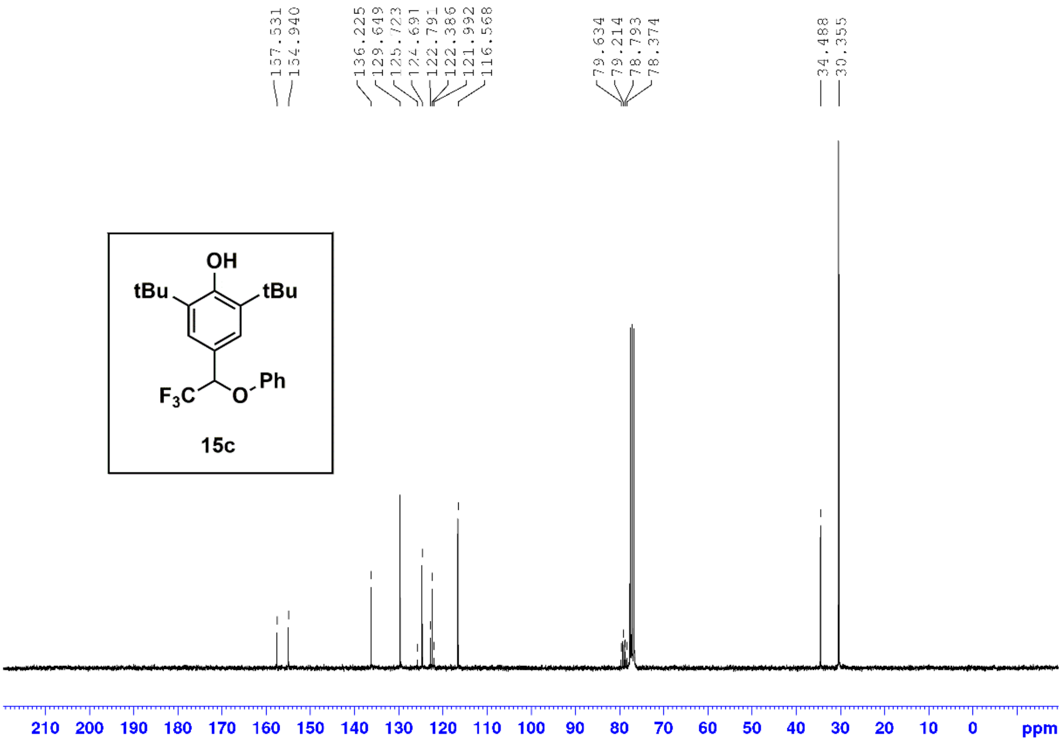
157.531
154.940
136.225
129.649
125.723
124.691
122.386
121.992
116.568
79.634
79.214
78.793
78.374
34.488
30.355
OH
tBu
tBu
F3C
O
Ph
15c
210 200 190 180 170 160 150 140 130 120 110 100 90 80 70 60 50 40 30 20 10 0 ppm

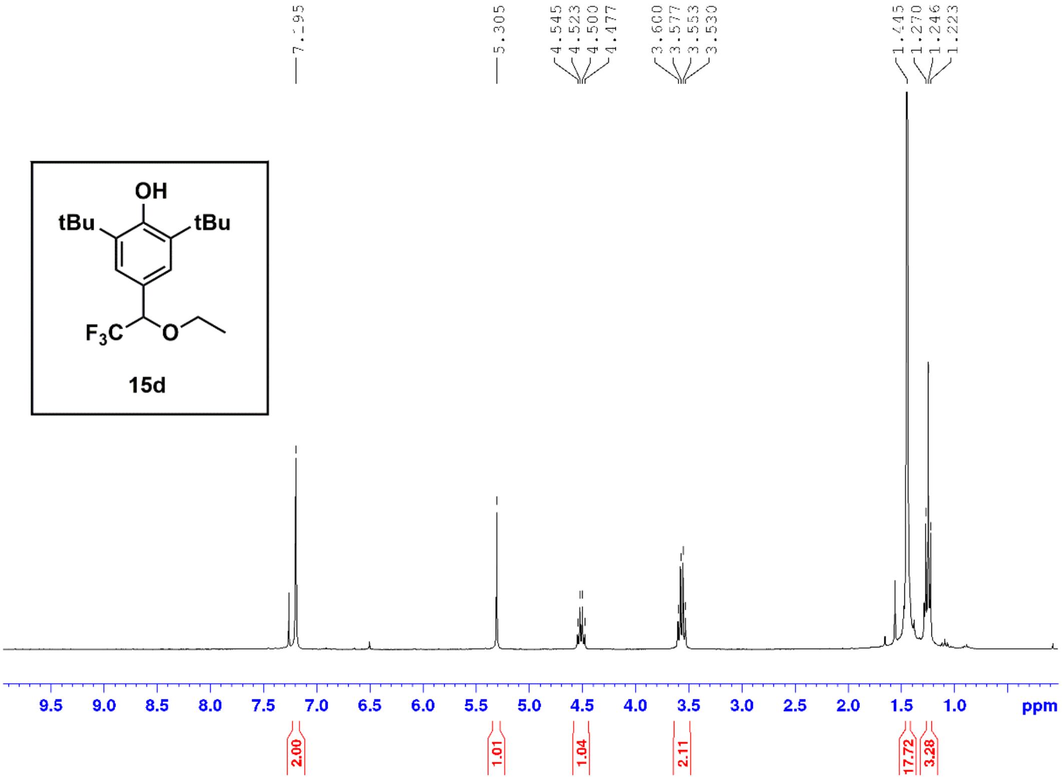
5.305
4.545
4.523
4.500
4.477
3.600
3.577
3.553
3.530
1.445
1.270
1.246
1.223
OH
tBu
tBu
F3C
O
15d
9.5 9.0 8.5 8.0 7.5 7.0 6.5 6.0 5.5 5.0 4.5 4.0 3.5 3.0 2.5 2.0 1.5 1.0 ppm
2.00
1.01
1.04
2.11
17.72
3.28

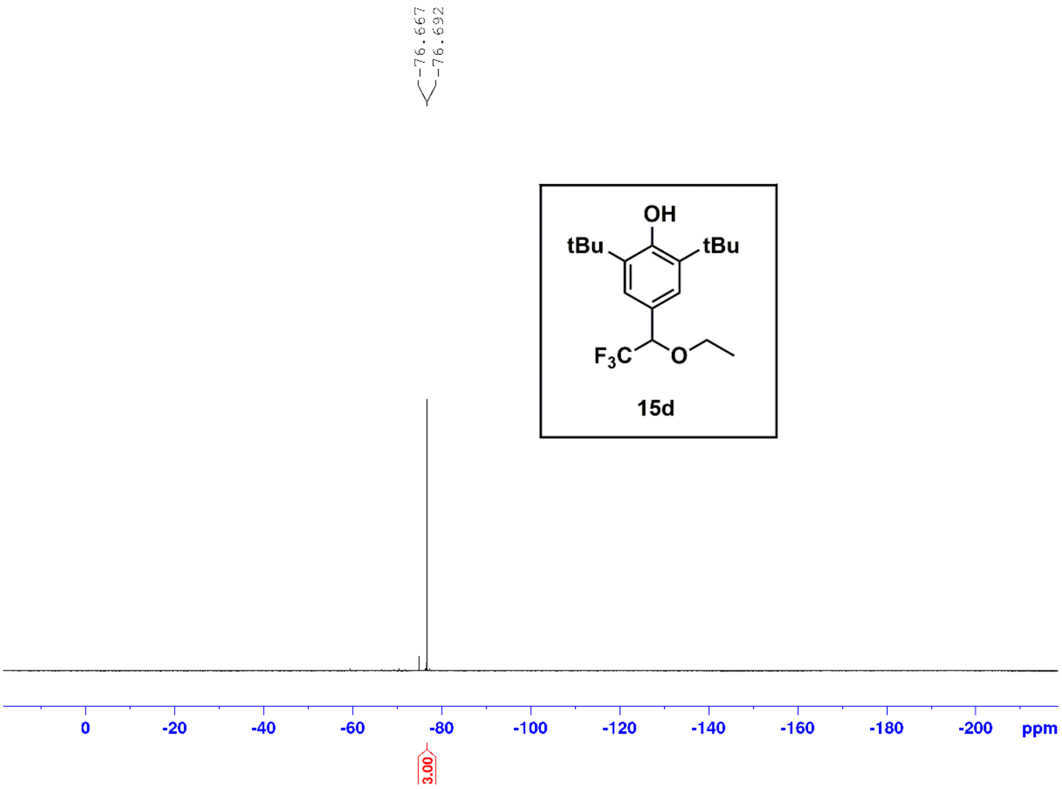
-76.667
-76.692
OH
tBu
tBu
F3C
O
15d
0
-20
-40
-60
-80
-100
-120
-140
-160
-180
-200
ppm
3.00

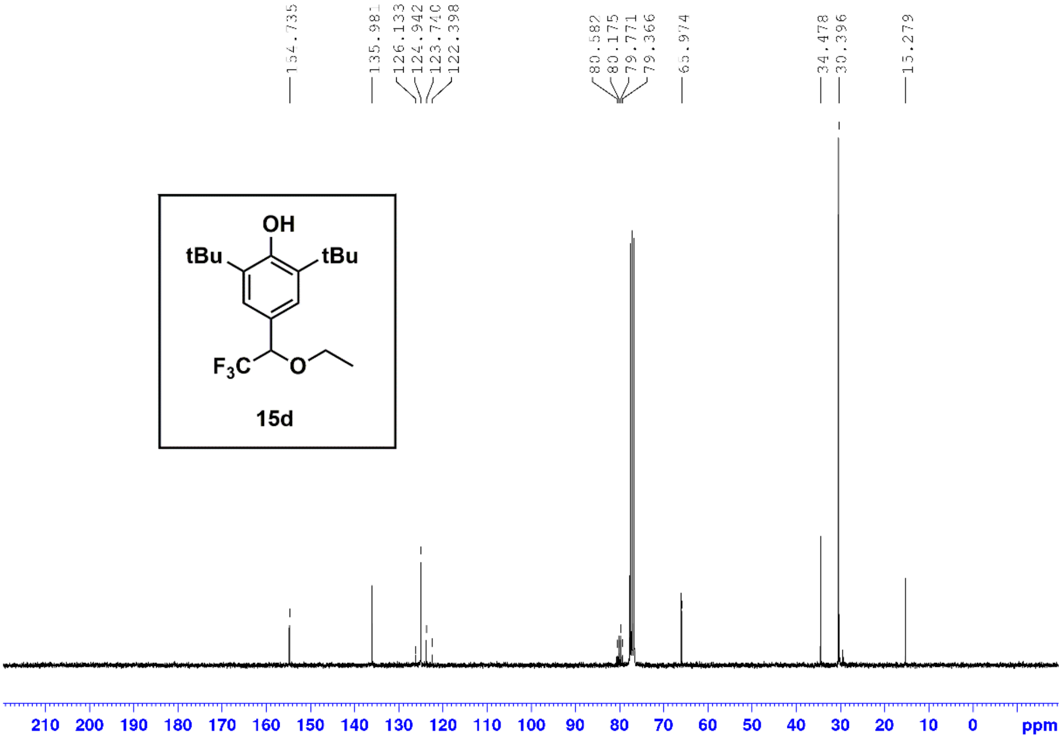
135.981
126.133
124.942
123.740
122.398
80.582
80.175
79.771
79.366
34.478
30.396
15.279
OH
tBu
tBu
F3C
O
15d
210
200
190
180
170
160
150
140
130
120
110
100
90
80
70
60
50
40
30
20
10
0
ppm

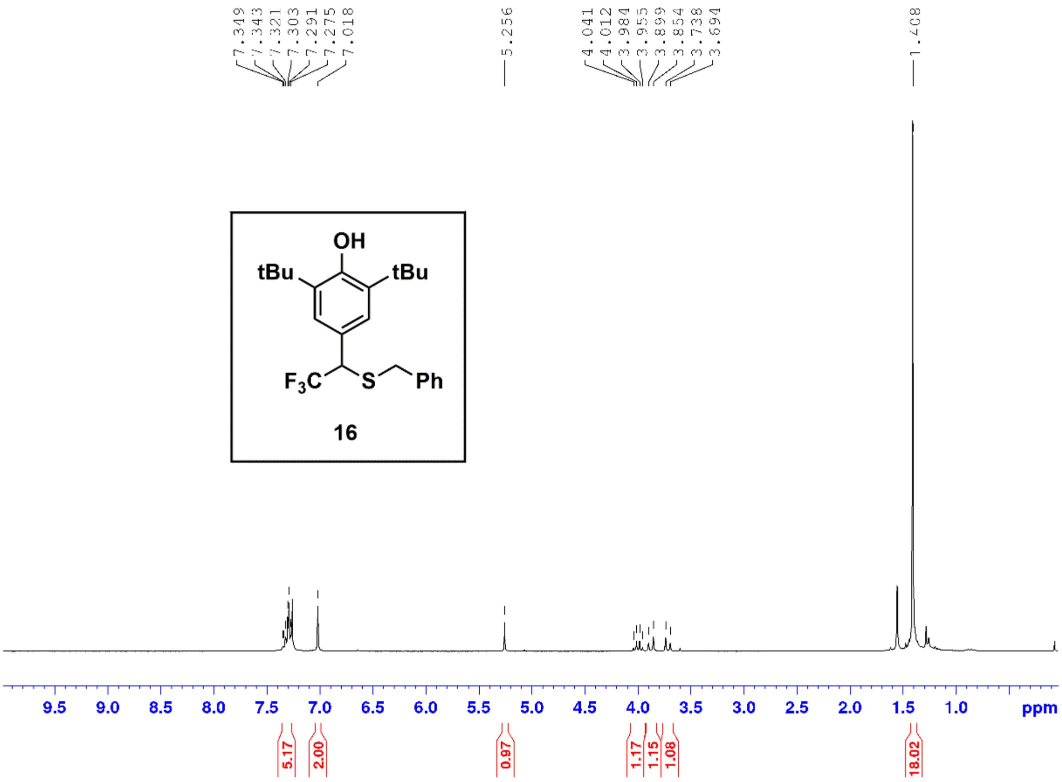
7.349
7.343
7.321
7.303
7.291
7.275
7.018
5.256
4.041
4.012
3.984
3.955
3.899
3.854
3.738
3.694
1.408
OH
tBu
tBu
F3C
S
Ph
16
9.5
9.0
8.5
8.0
7.5
7.0
6.5
6.0
5.5
5.0
4.5
4.0
3.5
3.0
2.5
2.0
1.5
1.0
ppm
5.17
2.00
0.97
1.17
1.15
1.08
18.02

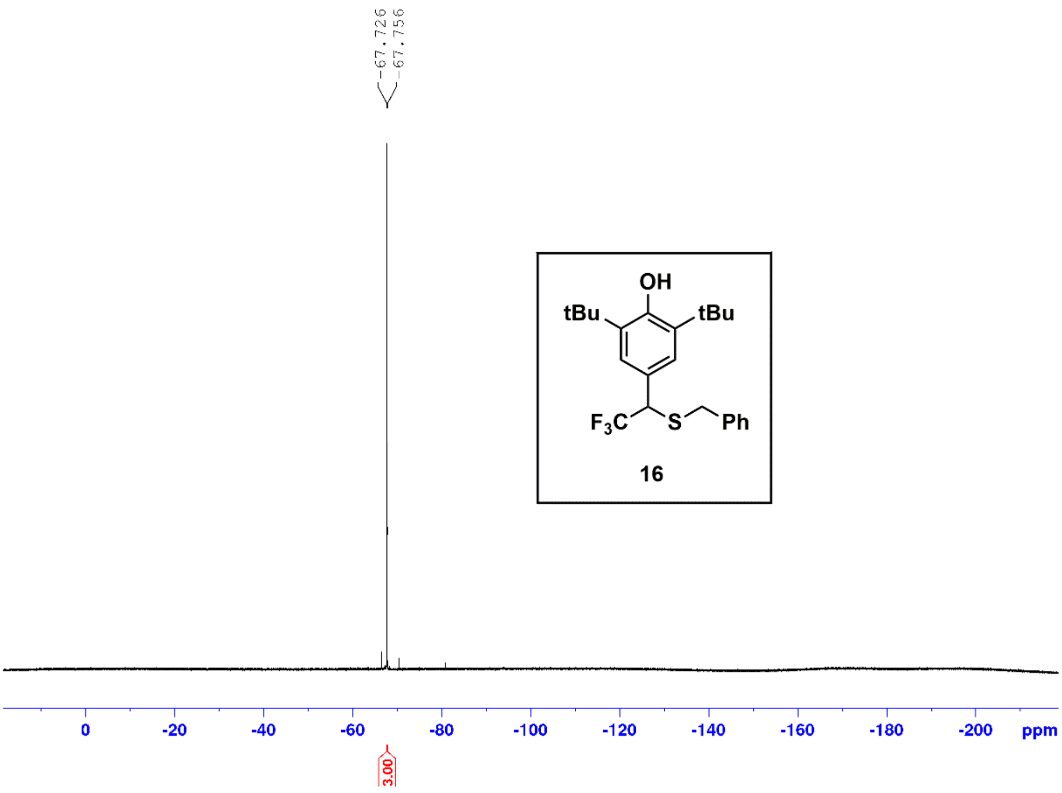
-67.726
-67.756
OH
tBu
tBu
F3C
S
Ph
16
0
-20
-40
-60
-80
-100
-120
-140
-160
-180
-200
ppm
3.00

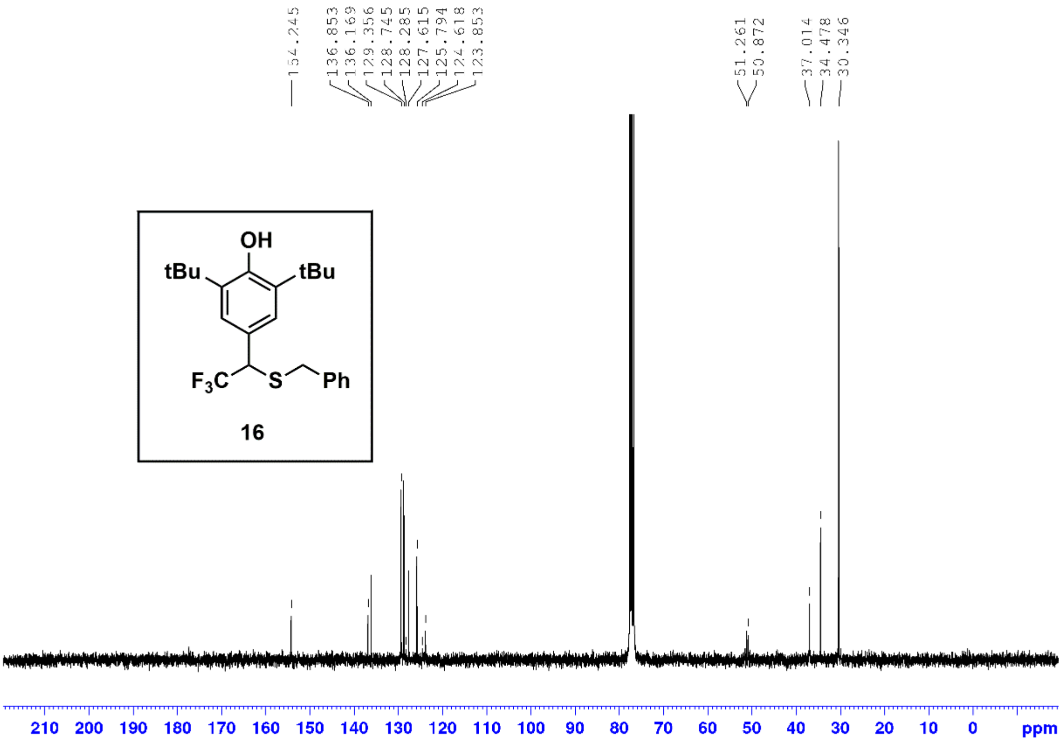
154.245
136.853
136.169
129.356
128.745
128.285
127.615
125.794
124.618
123.853
51.261
50.872
37.014
34.478
30.346
OH
tBu
tBu
F3C
S
Ph
16
210 200 190 180 170 160 150 140 130 120 110 100 90 80 70 60 50 40 30 20 10 0 ppm

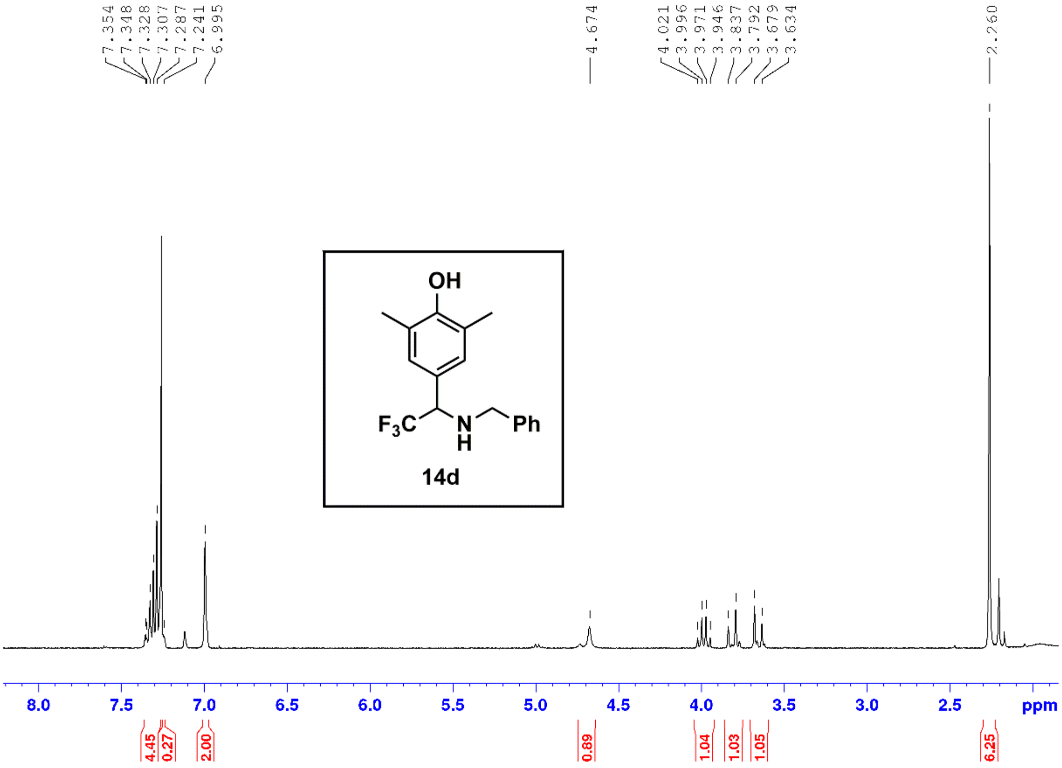
7.354
7.348
7.328
7.307
7.287
7.241
6.995
4.674
4.021
3.996
3.971
3.946
3.837
3.792
3.679
3.634
2.260
OH
F3C
N
H
Ph
14d
8.0 7.5 7.0 6.5 6.0 5.5 5.0 4.5 4.0 3.5 3.0 2.5 ppm
4.45
0.27
2.00
0.89
1.04
1.03
1.05
6.25

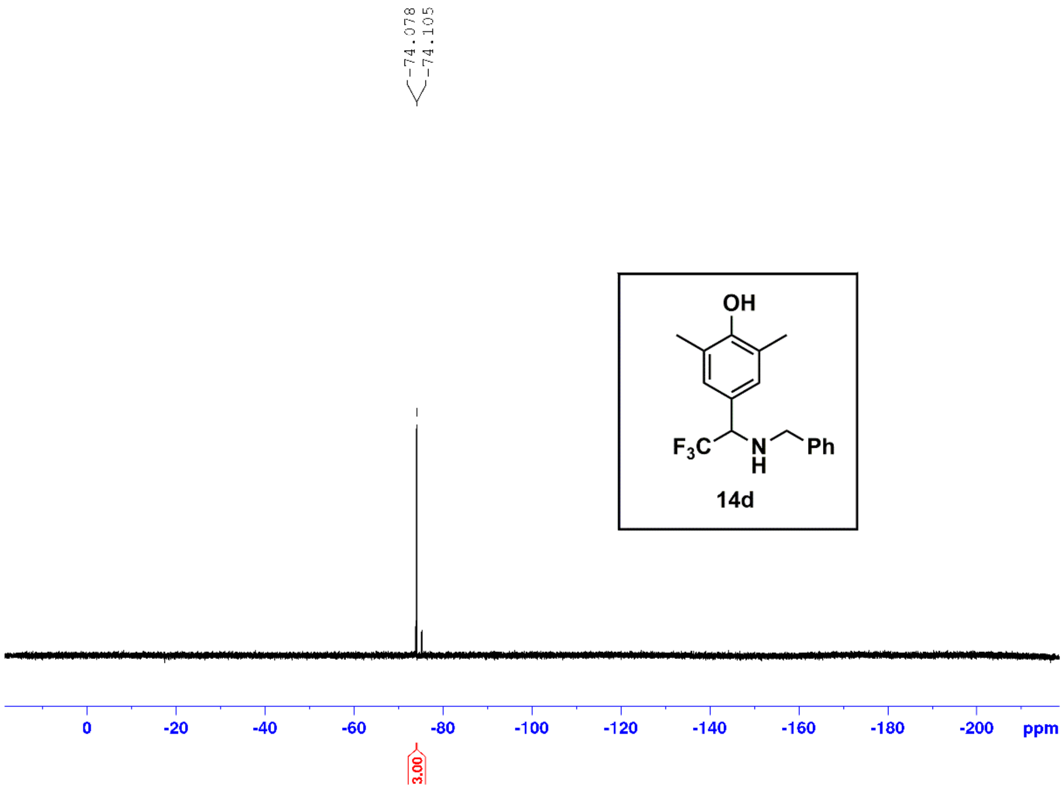
-74.078
-74.105
OH
F3C
N
H
Ph
14d
0
-20
-40
-60
-80
-100
-120
-140
-160
-180
-200
ppm
3.00

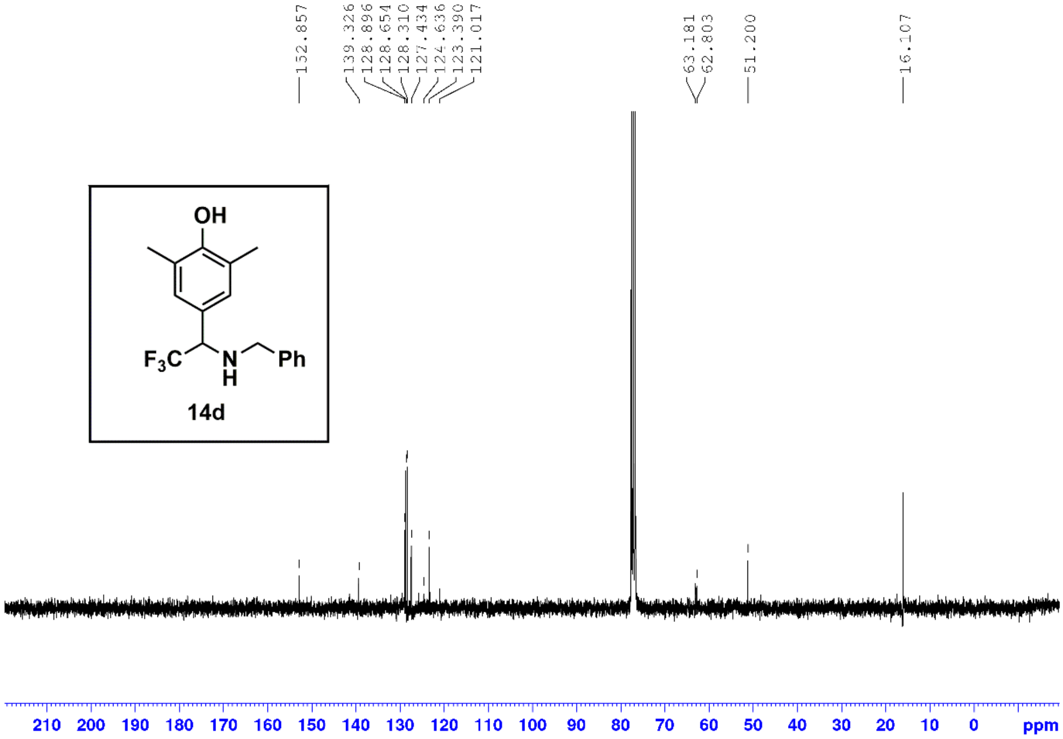
152.857
139.326
128.896
128.654
128.310
127.434
124.636
123.390
121.017
63.181
62.803
51.200
16.107
OH
F3C
N
H
Ph
14d
210
200
190
180
170
160
150
140
130
120
110
100
90
80
70
60
50
40
30
20
10
0
ppm

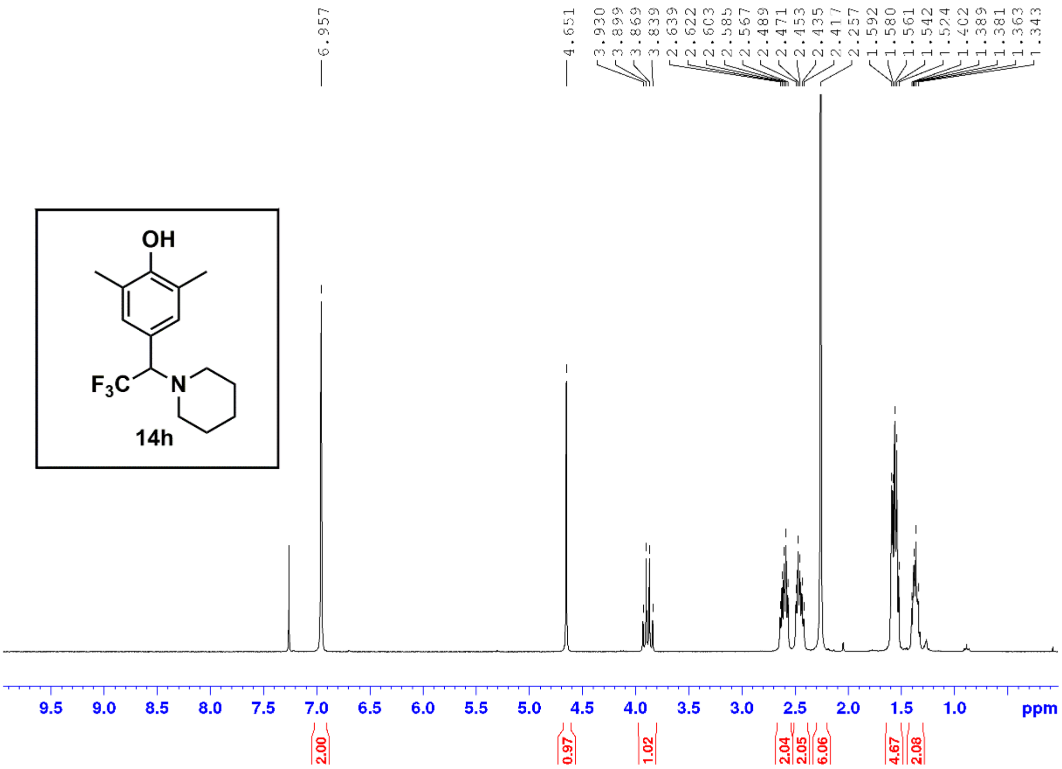
6.957
4.651
3.930
3.899
3.869
3.839
2.639
2.622
2.603
2.585
2.567
2.489
2.471
2.453
2.435
2.417
2.257
1.592
1.580
1.561
1.542
1.524
1.402
1.389
1.381
1.363
1.343
OH
F_3C
N
14h
9.5 9.0 8.5 8.0 7.5 7.0 6.5 6.0 5.5 5.0 4.5 4.0 3.5 3.0 2.5 2.0 1.5 1.0 ppm
2.00
0.97
1.02
2.04
2.05
6.06
4.67
2.08

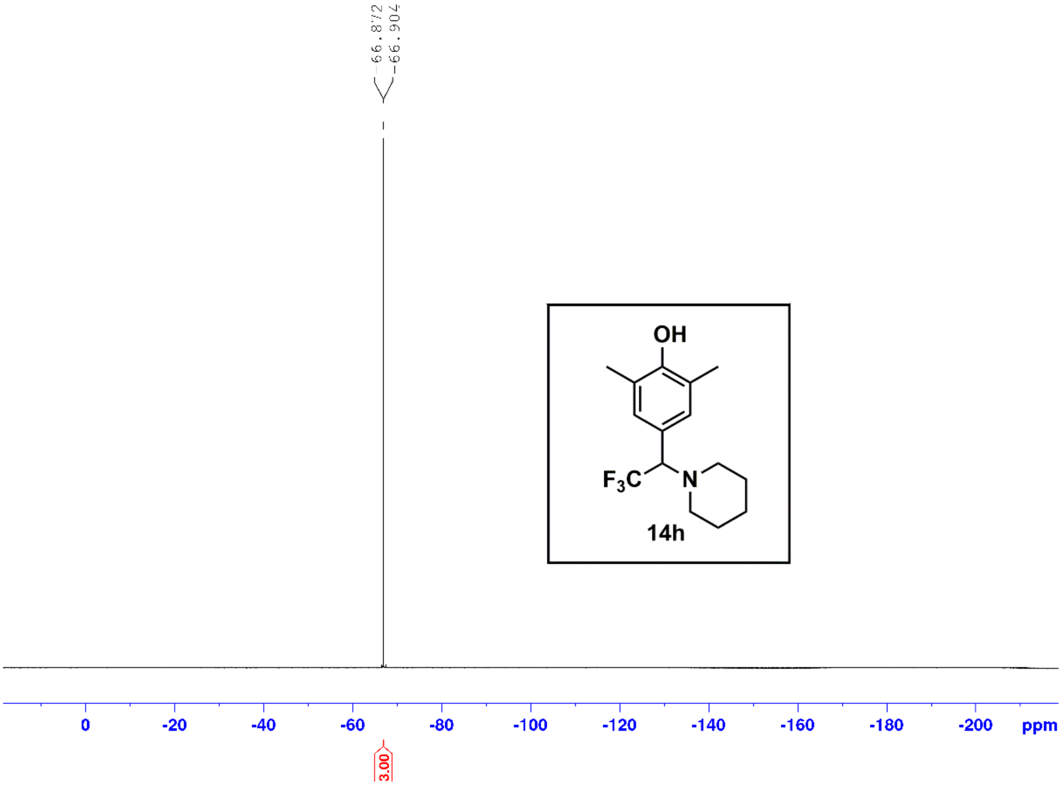
-66.872
-66.904
OH
F_3C
N
14h
0 -20 -40 -60 -80 -100 -120 -140 -160 -180 -200 ppm
3.00

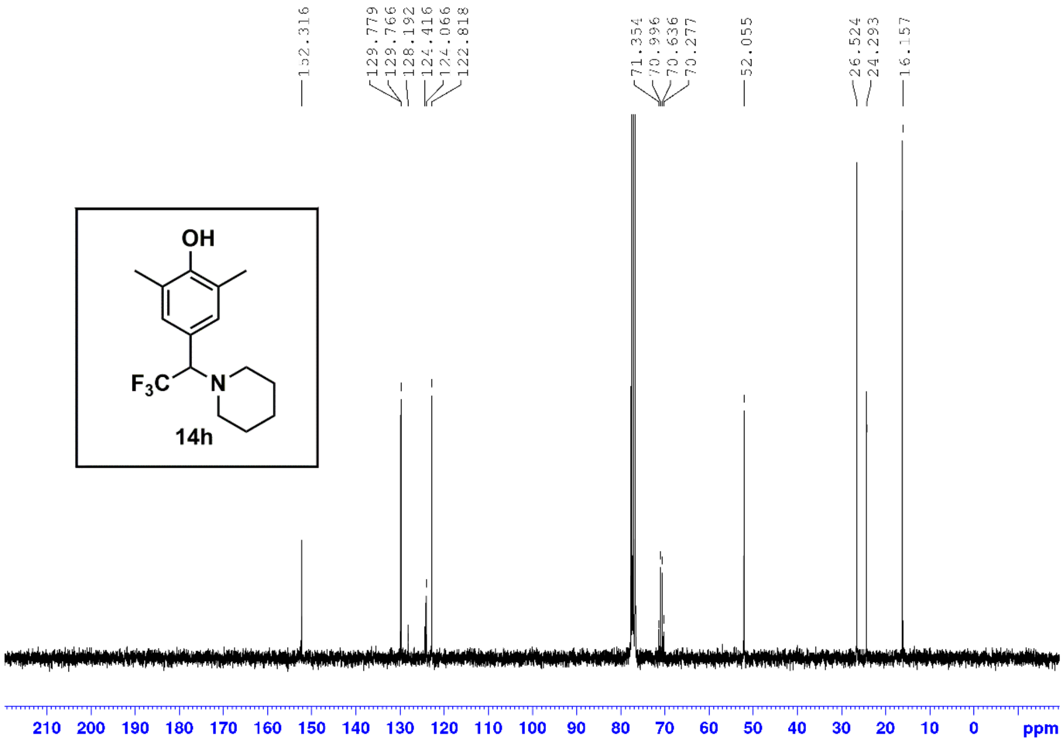
OH
F3C
N
14h
152.316
129.779
129.766
128.192
124.416
124.066
122.818
71.354
70.996
70.636
70.277
52.055
26.524
24.293
16.157
210 200 190 180 170 160 150 140 130 120 110 100 90 80 70 60 50 40 30 20 10 0 ppm

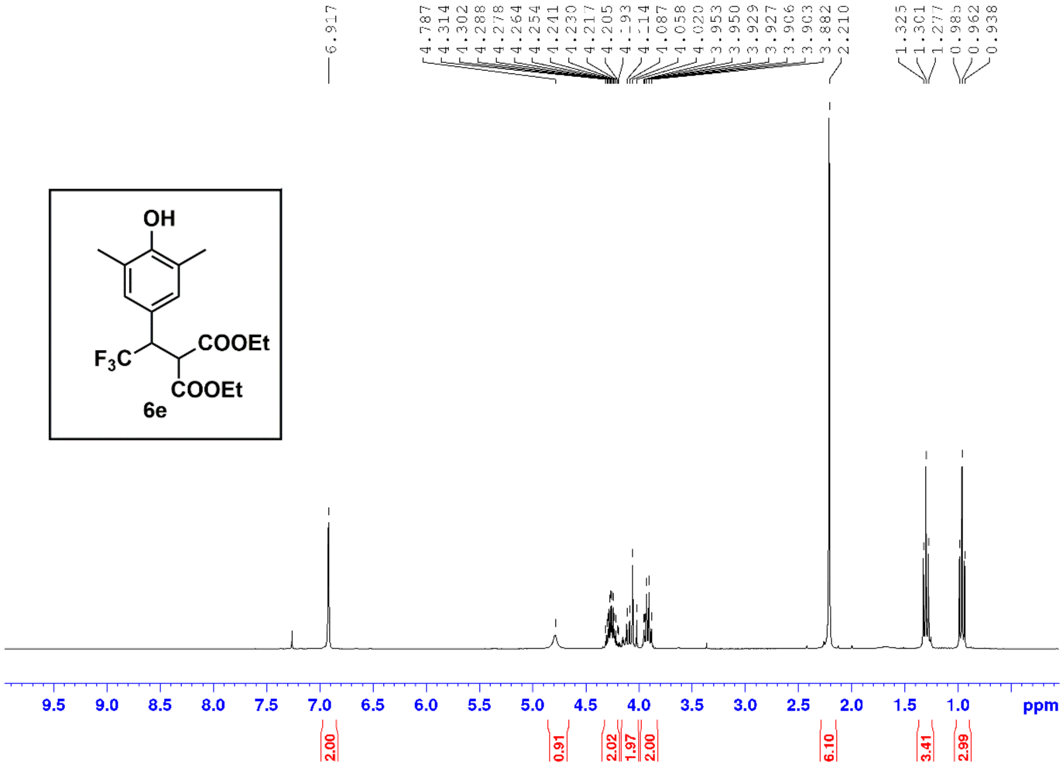
OH
F3C
COOEt
COOEt
6e
6.917
4.787
4.314
4.302
4.288
4.278
4.264
4.254
4.241
4.230
4.217
4.205
4.193
4.114
4.087
4.058
4.020
3.953
3.950
3.929
3.927
3.906
3.903
3.882
2.210
1.325
1.301
1.277
0.985
0.962
0.938
9.5 9.0 8.5 8.0 7.5 7.0 6.5 6.0 5.5 5.0 4.5 4.0 3.5 3.0 2.5 2.0 1.5 1.0 ppm
2.00
0.91
2.02
1.97
2.00
6.10
3.41
2.99

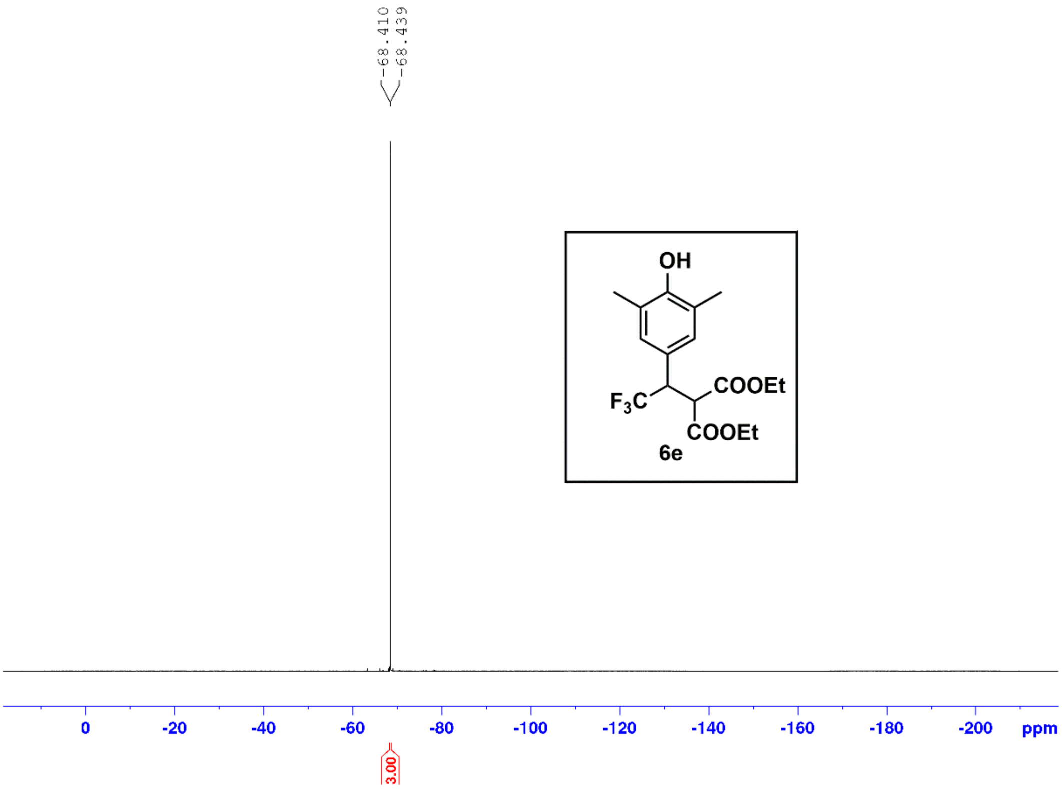
-68.410
-68.439
OH
COOEt
F3C
COOEt
6e
0 -20 -40 -60 -80 -100 -120 -140 -160 -180 -200 ppm
3.00

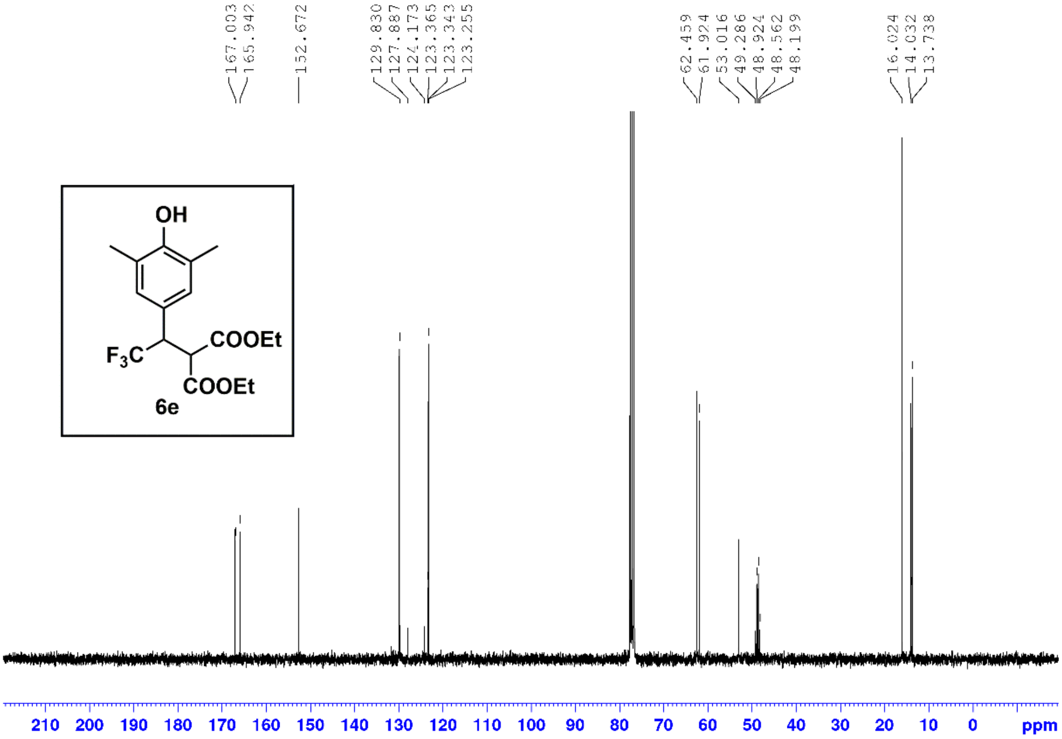
167.003
165.942
152.672
129.830
127.887
124.173
123.365
123.343
123.255
62.459
61.924
53.016
49.286
48.924
48.562
48.199
16.024
14.032
13.738
OH
COOEt
F3C
COOEt
6e
210 200 190 180 170 160 150 140 130 120 110 100 90 80 70 60 50 40 30 20 10 0 ppm

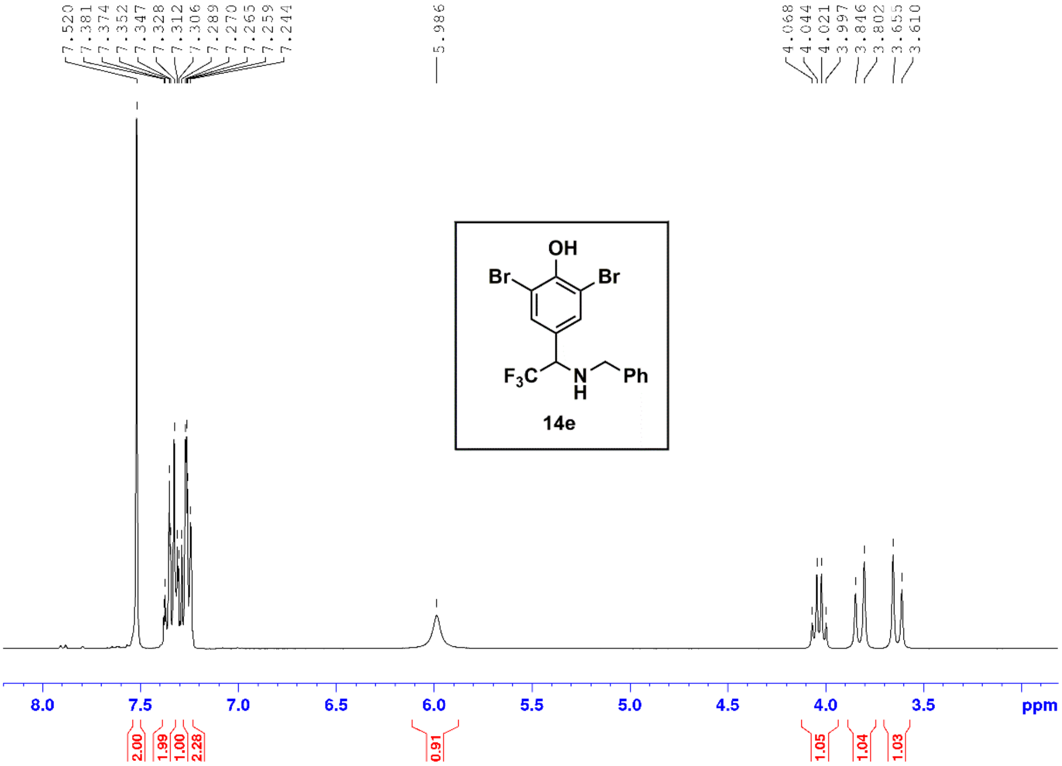

7.520
7.381
7.374
7.352
7.347
7.328
7.312
7.306
7.289
7.270
7.265
7.259
7.244
5.986
4.068
4.044
4.021
3.997
3.846
3.802
3.655
3.610
OH
Br
Br
F3C
N
H
Ph
14e
8.0
7.5
7.0
6.5
6.0
5.5
5.0
4.5
4.0
3.5
ppm
2.00
1.99
1.00
2.28
0.91
1.05
1.04
1.03

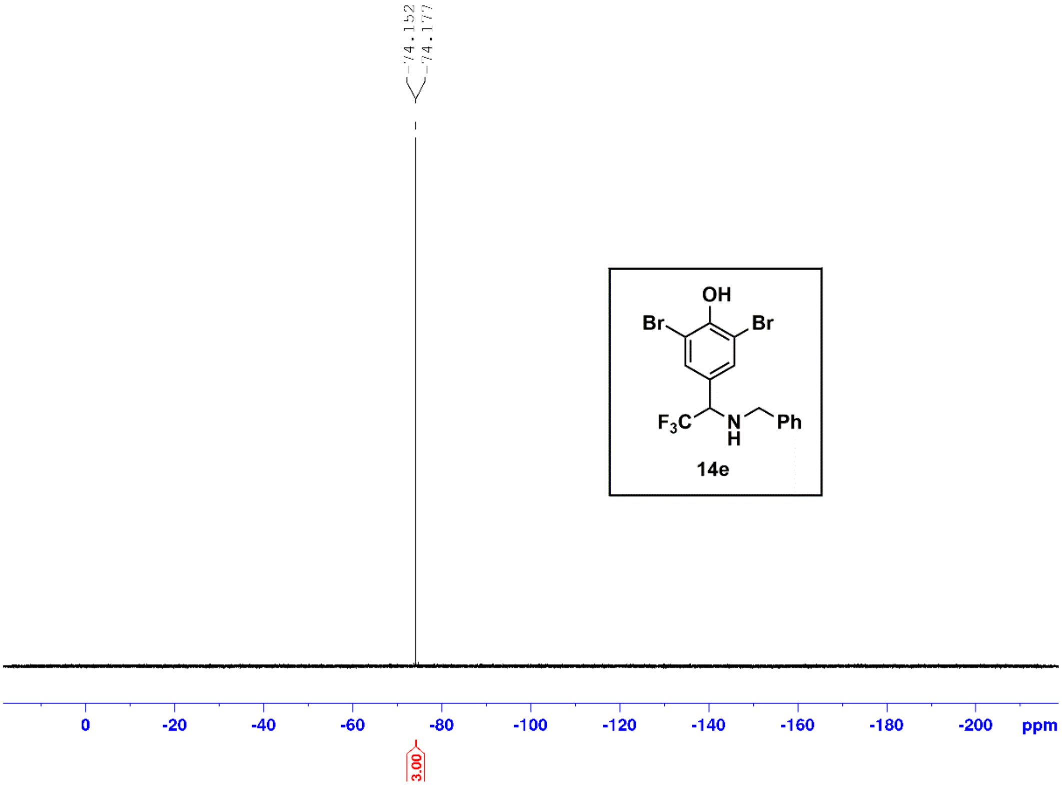

-74.152
-74.177
OH
Br
Br
F3C
N
H
Ph
14e
0
-20
-40
-60
-80
-100
-120
-140
-160
-180
-200
ppm
3.00

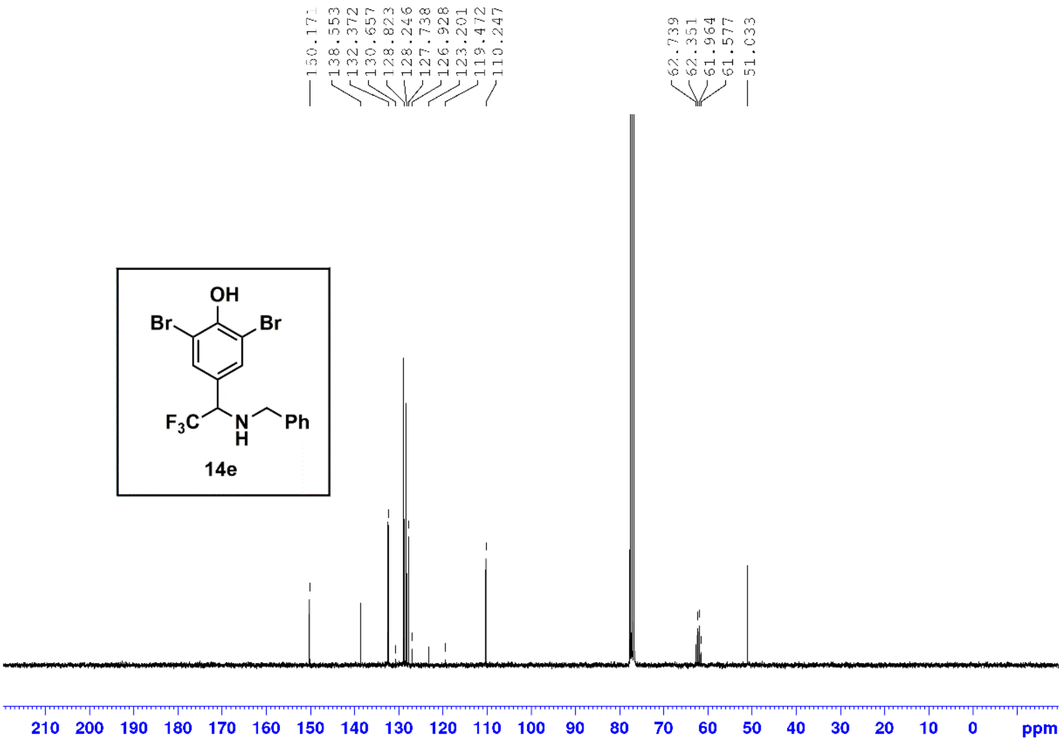
150.171
138.553
132.372
130.657
128.823
128.246
127.738
126.928
123.201
119.472
110.247
62.739
62.351
61.964
61.577
51.033
OH
Br
Br
F3C
N
H
Ph
14e
210 200 190 180 170 160 150 140 130 120 110 100 90 80 70 60 50 40 30 20 10 0 ppm

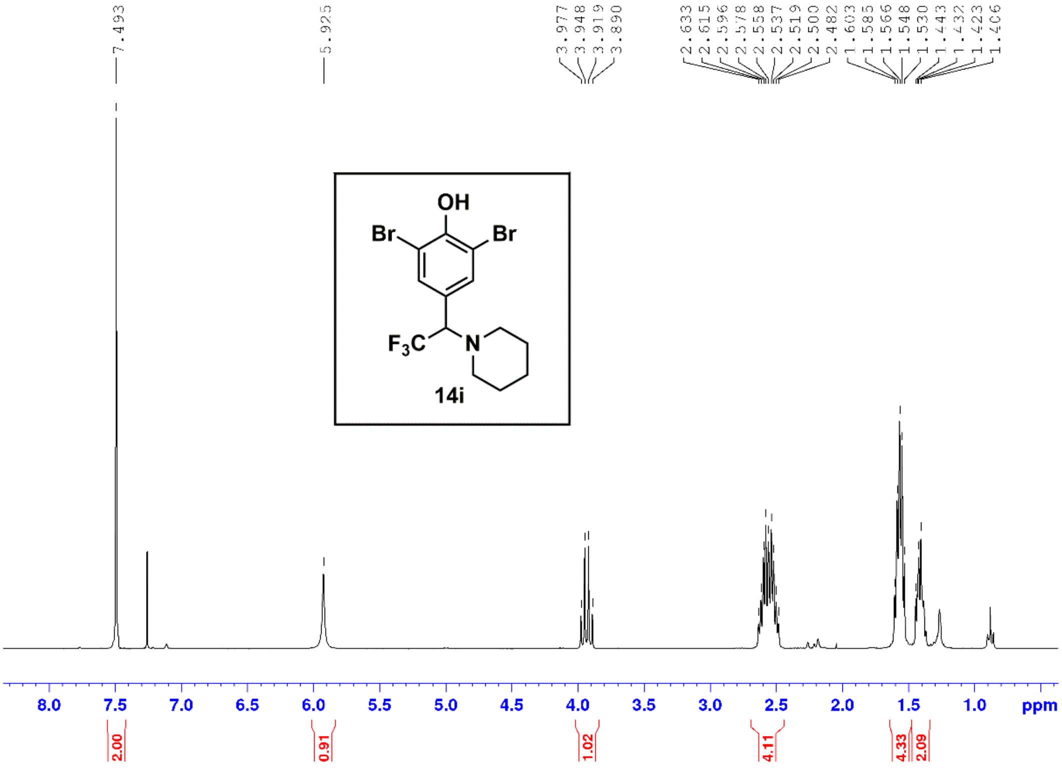
7.493
5.925
3.977
3.948
3.919
3.890
2.633
2.615
2.596
2.578
2.558
2.537
2.519
2.500
2.482
1.603
1.585
1.566
1.548
1.530
1.443
1.432
1.423
1.406
OH
Br
Br
F3C
N
14i
8.0 7.5 7.0 6.5 6.0 5.5 5.0 4.5 4.0 3.5 3.0 2.5 2.0 1.5 1.0 ppm
2.00
0.91
1.02
4.11
4.33
2.09

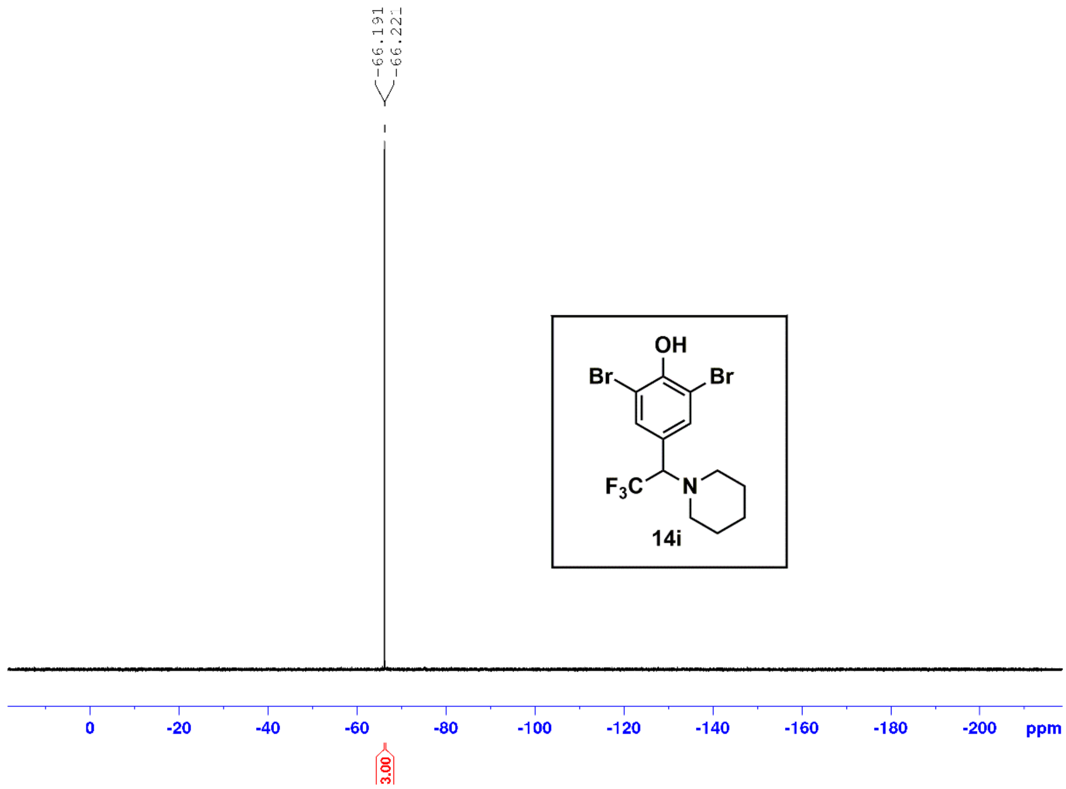
-66.191
-66.22
OH
Br
Br
F3C
N
14i
0
-20
-40
-60
-80
-100
-120
-140
-160
-180
-200
ppm
3.00

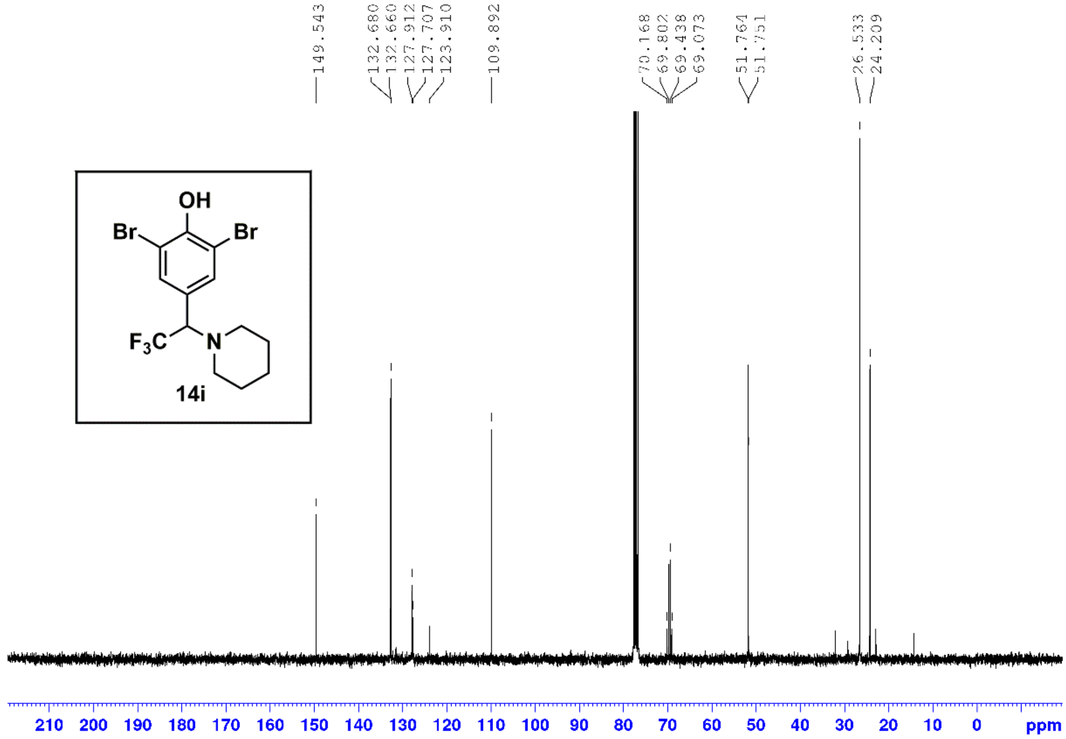
149.543
132.680
132.660
127.912
127.707
123.910
109.892
70.168
69.802
69.438
69.073
51.764
51.751
26.533
24.209
OH
Br
Br
F3C
N
14i
210
200
190
180
170
160
150
140
130
120
110
100
90
80
70
60
50
40
30
20
10
0
ppm

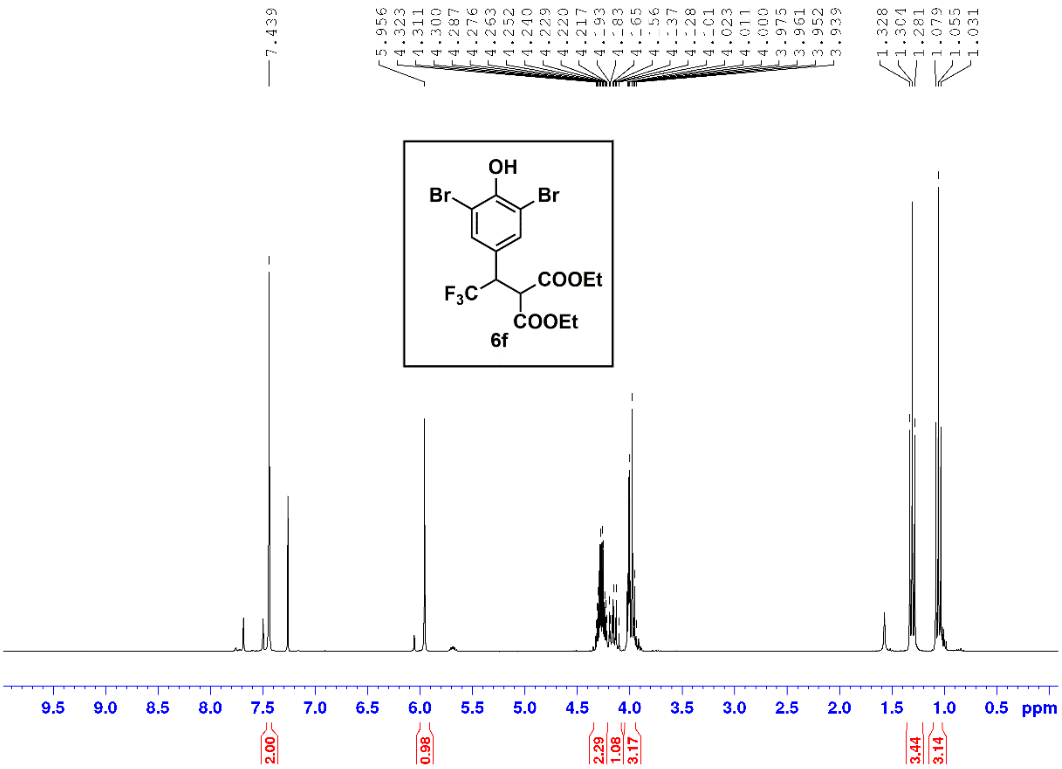
OH
Br
Br
F3C
COOEt
COOEt
6f
ppm

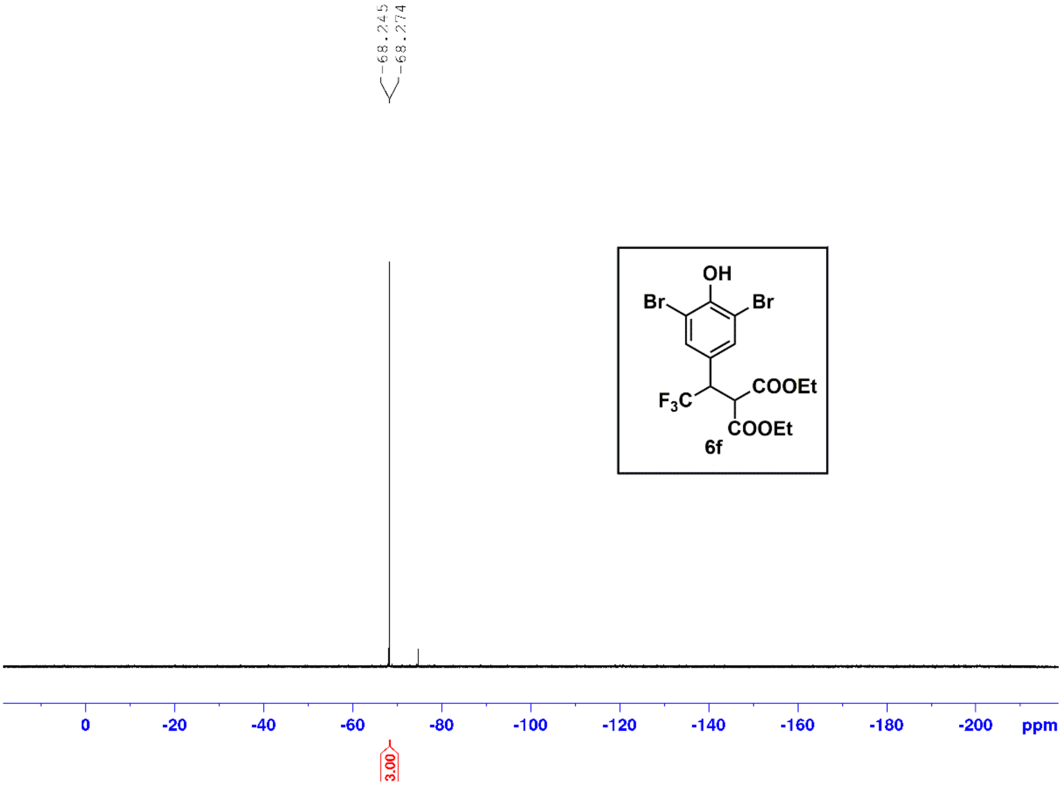
-68.245
-68.274
OH
Br
Br
F3C
COOEt
COOEt
6f
0
-20
-40
-60
-80
-100
-120
-140
-160
-180
-200
ppm
3.00

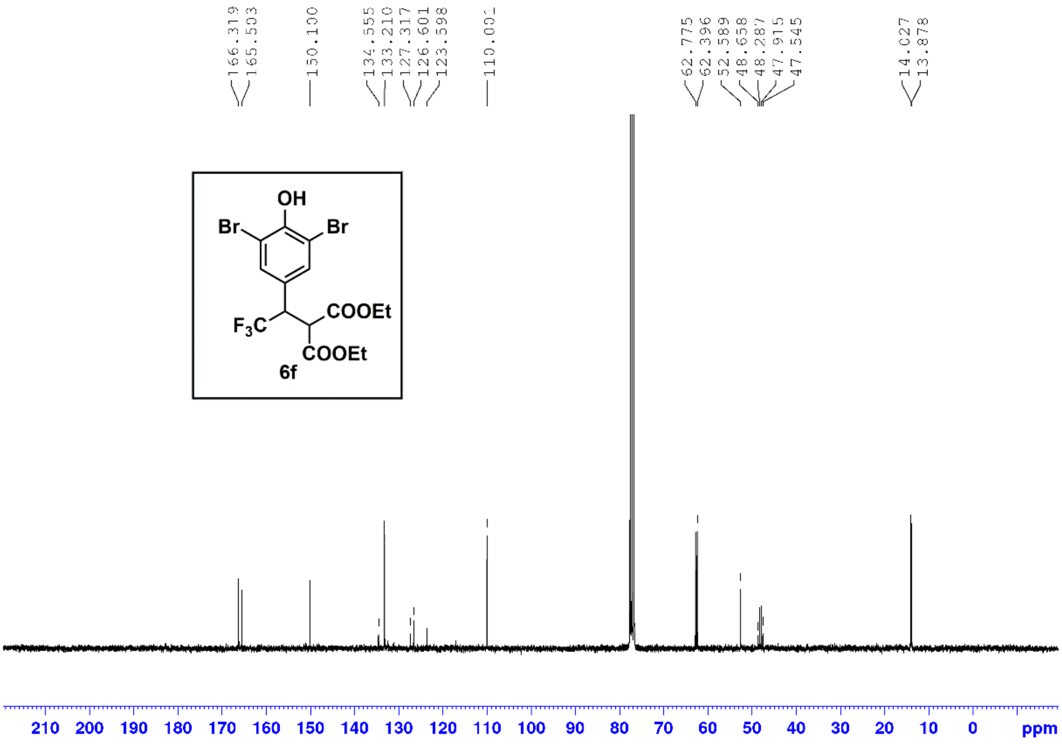
166.319
165.503
150.100
134.555
133.210
127.317
126.601
123.598
62.775
62.396
52.589
48.658
48.287
47.915
47.545
14.027
13.878
OH
Br
Br
COOEt
F3C
COOEt
6f
210 200 190 180 170 160 150 140 130 120 110 100 90 80 70 60 50 40 30 20 10 0 ppm

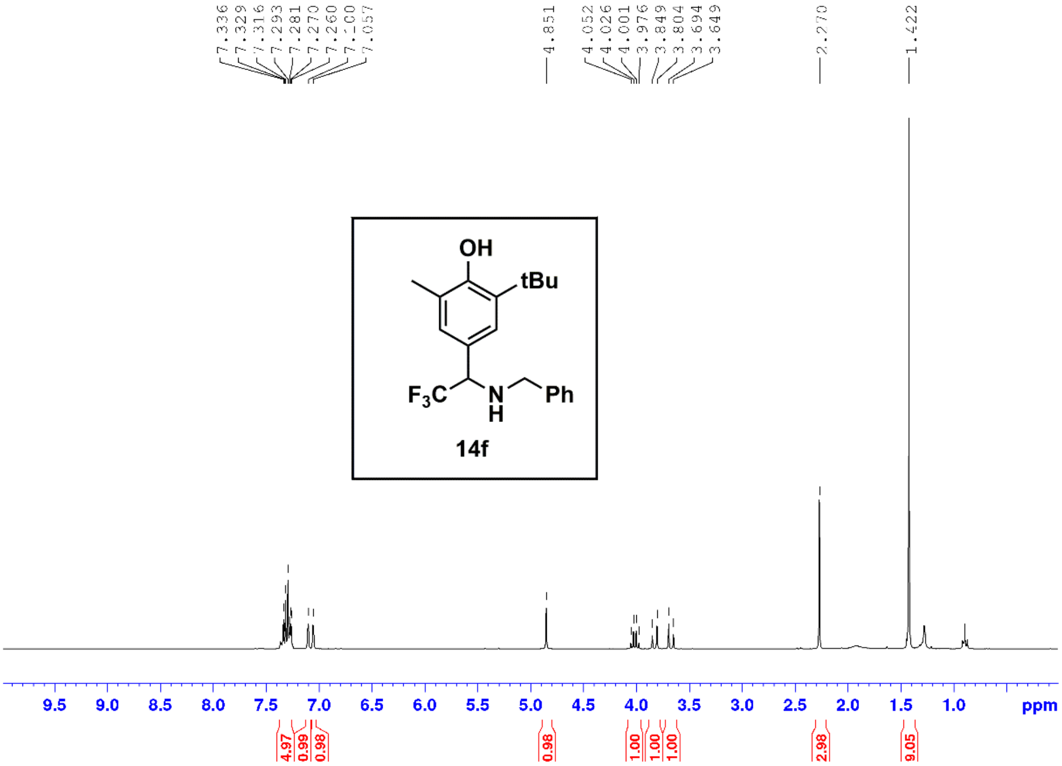
7.336
7.329
7.316
7.293
7.281
7.270
7.260
7.057
4.851
4.052
4.026
4.001
3.976
3.849
3.804
3.694
3.649
2.270
1.422
OH
tBu
F3C
N
H
Ph
14f
9.5 9.0 8.5 8.0 7.5 7.0 6.5 6.0 5.5 5.0 4.5 4.0 3.5 3.0 2.5 2.0 1.5 1.0 ppm
4.97
0.99
0.98
0.98
1.00
1.00
1.00
2.98
9.05

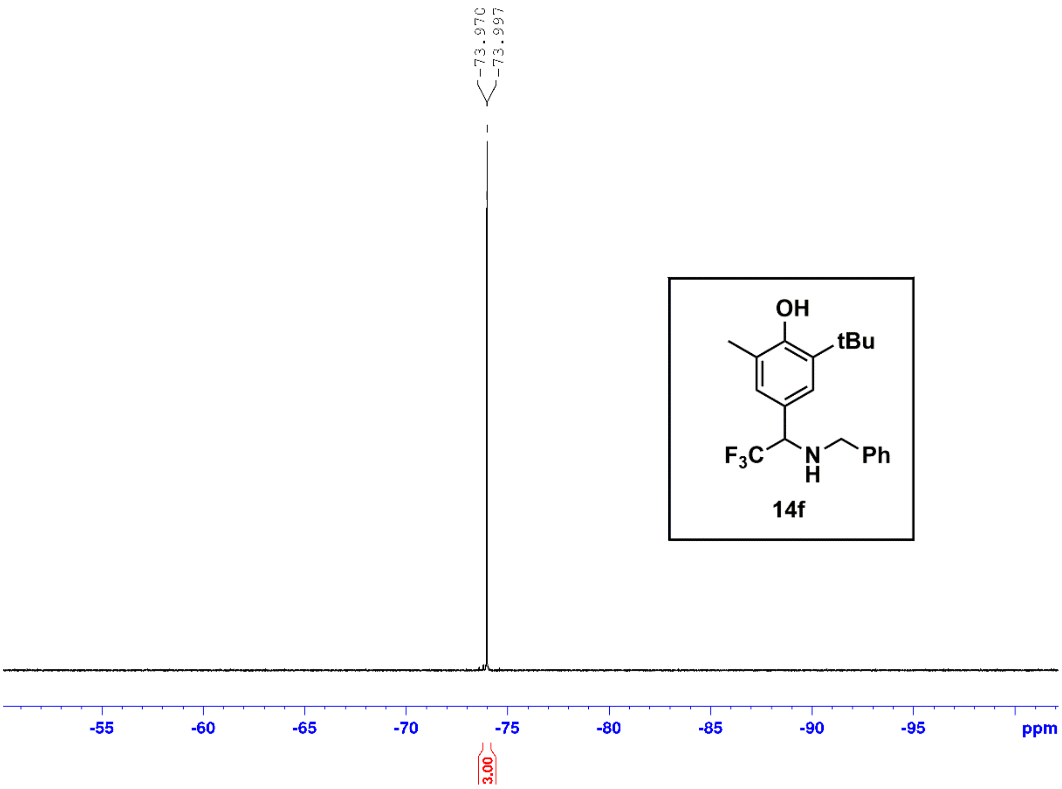
-73.970
-73.997
OH
tBu
F3C
N
H
Ph
14f
-55
-60
-65
-70
-75
-80
-85
-90
-95
ppm
3.00

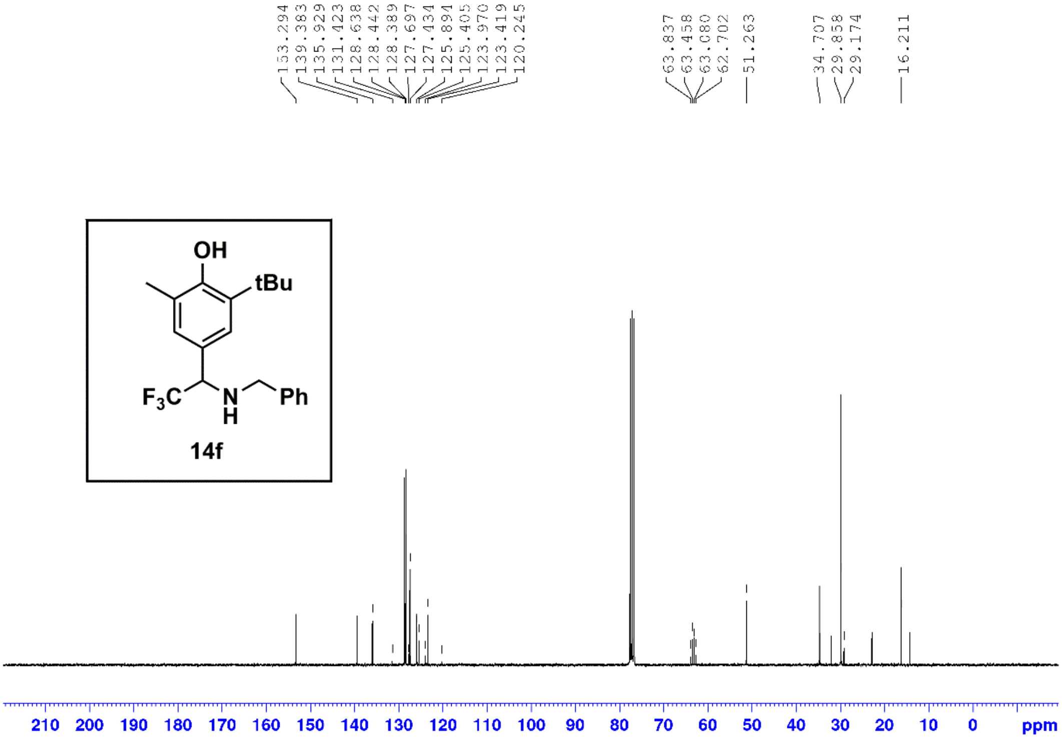
153.294
139.383
135.929
131.423
128.638
128.442
128.389
127.697
127.434
125.894
125.405
123.970
123.419
120.245
63.837
63.458
63.080
62.702
51.263
34.707
29.858
29.174
16.211
OH
tBu
F3C
N
H
Ph
14f
210
200
190
180
170
160
150
140
130
120
110
100
90
80
70
60
50
40
30
20
10
0
ppm

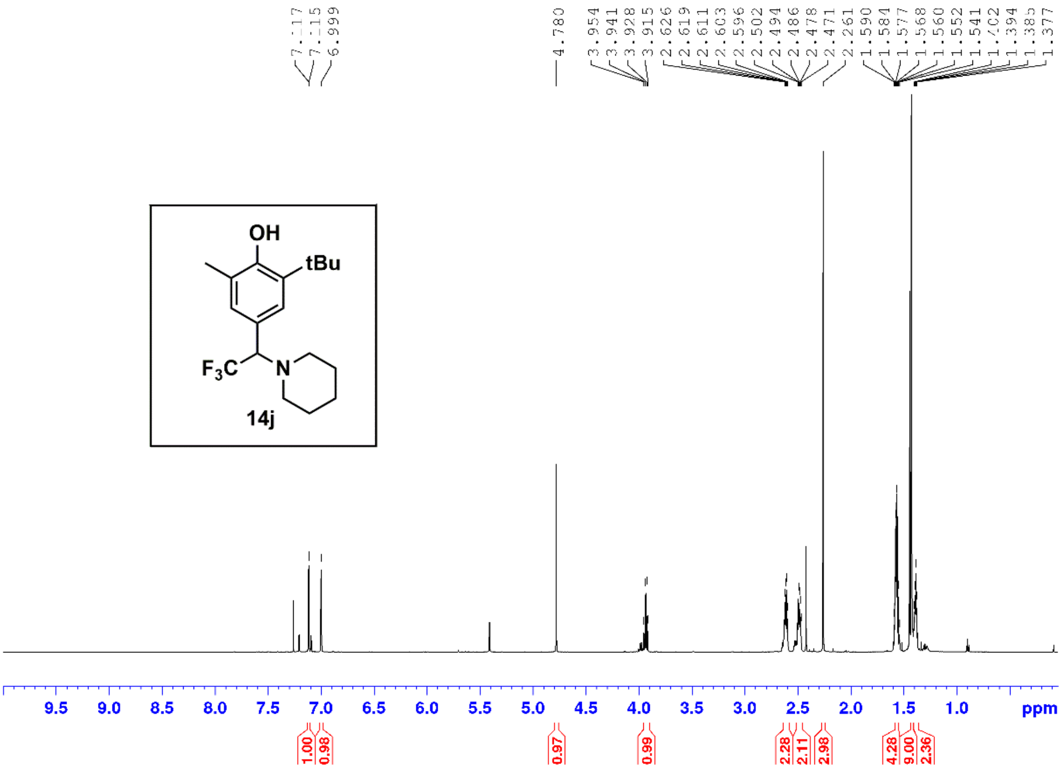

7.117
7.115
6.999
4.780
3.954
3.941
3.928
3.915
2.626
2.619
2.611
2.603
2.596
2.502
2.494
2.486
2.478
2.471
2.261
1.590
1.584
1.577
1.568
1.560
1.552
1.541
1.394
1.385
1.377
OH
tBu
F3C
N
14j
ppm
1.00
0.98
0.97
0.99
2.28
2.11
2.98
4.28
9.00
2.36

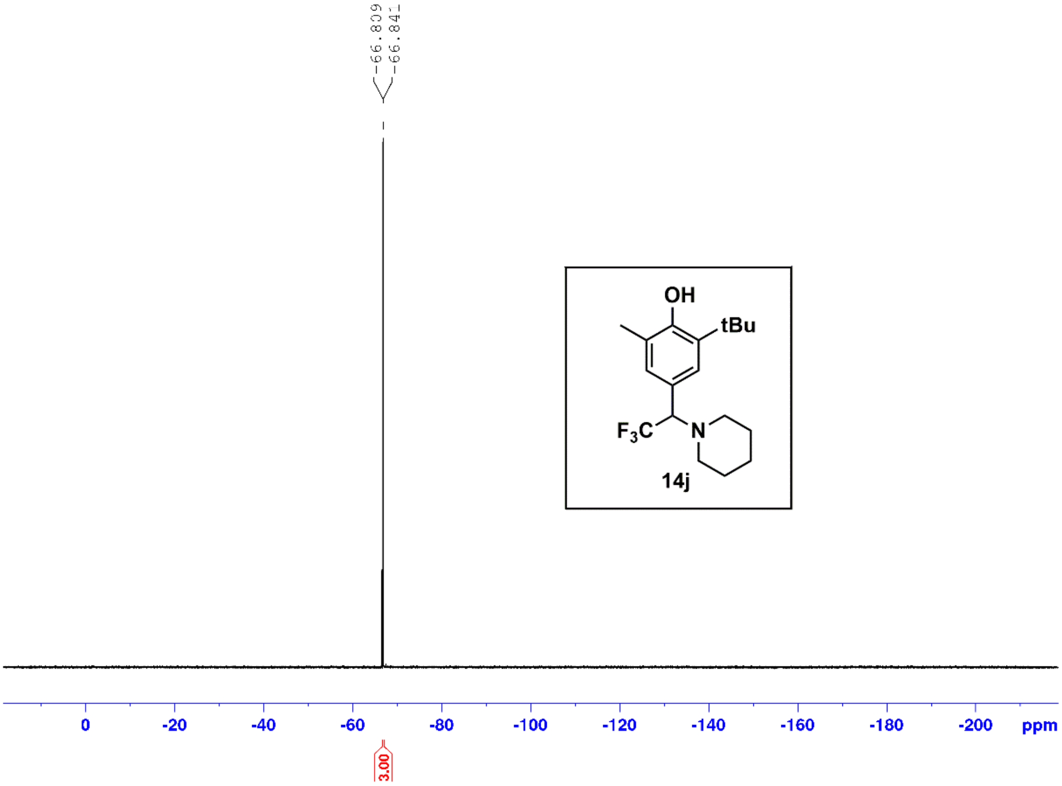

-66.809
-66.841
OH
tBu
F3C
N
14j
ppm
3.00

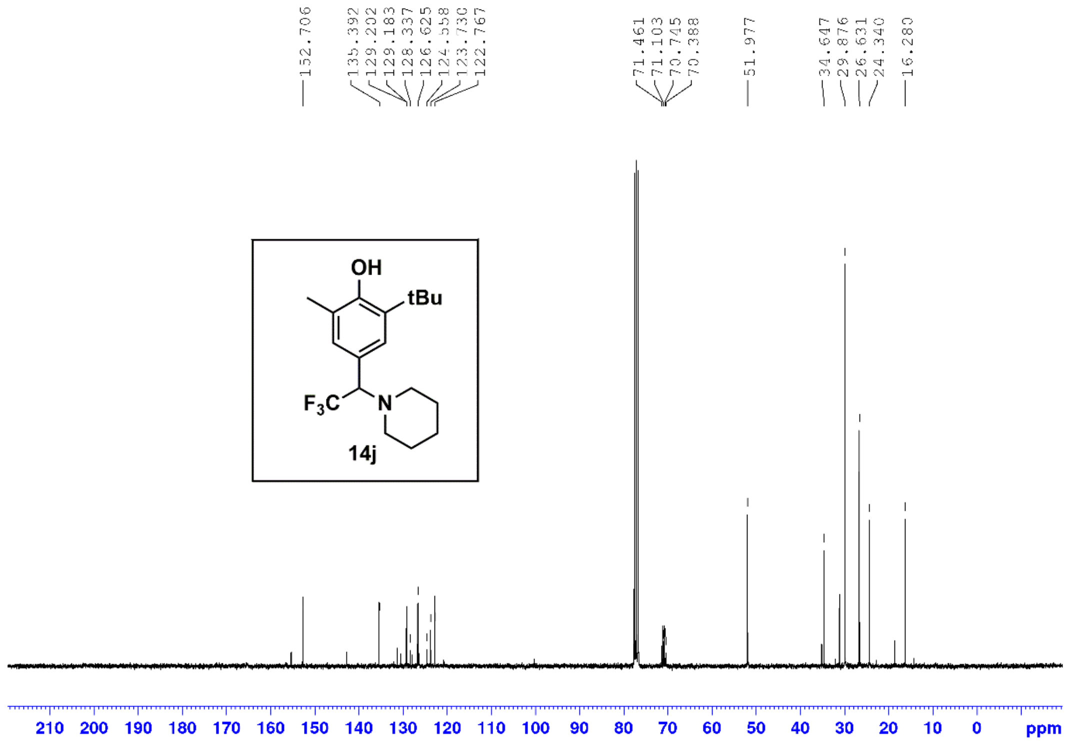
152.706
135.392
129.202
129.183
128.337
126.625
124.558
123.730
122.767
71.461
71.103
70.745
70.388
51.977
34.647
29.876
26.631
24.340
16.280
OH
tBu
F3C
N
14j
210 200 190 180 170 160 150 140 130 120 110 100 90 80 70 60 50 40 30 20 10 0 ppm

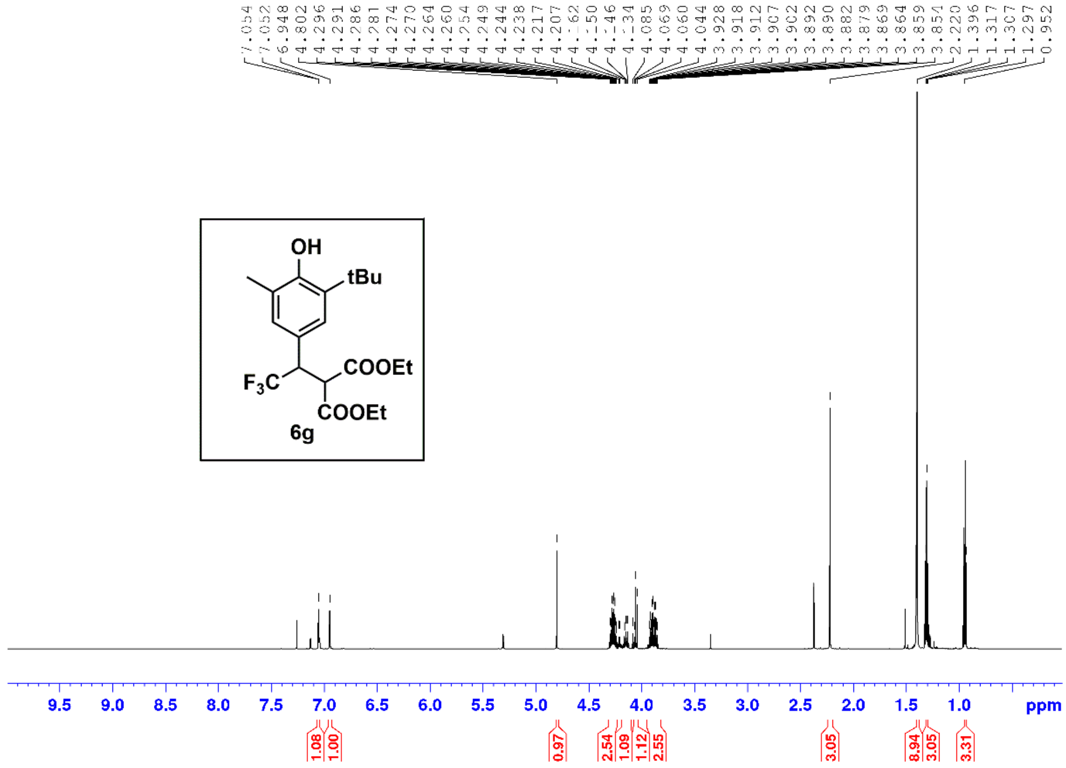
7.054
7.052
6.948
4.802
4.296
4.291
4.286
4.281
4.274
4.270
4.264
4.260
4.254
4.249
4.244
4.238
4.217
4.207
4.085
4.069
4.060
4.044
3.928
3.918
3.912
3.907
3.902
3.892
3.890
3.882
3.879
3.869
3.864
3.859
3.854
2.220
1.396
1.317
1.307
1.297
0.952
OH
tBu
F3C
COOEt
COOEt
6g
9.5 9.0 8.5 8.0 7.5 7.0 6.5 6.0 5.5 5.0 4.5 4.0 3.5 3.0 2.5 2.0 1.5 1.0 ppm
1.08
1.00
0.97
2.54
1.09
1.12
2.55
3.05
8.94
3.05
3.31

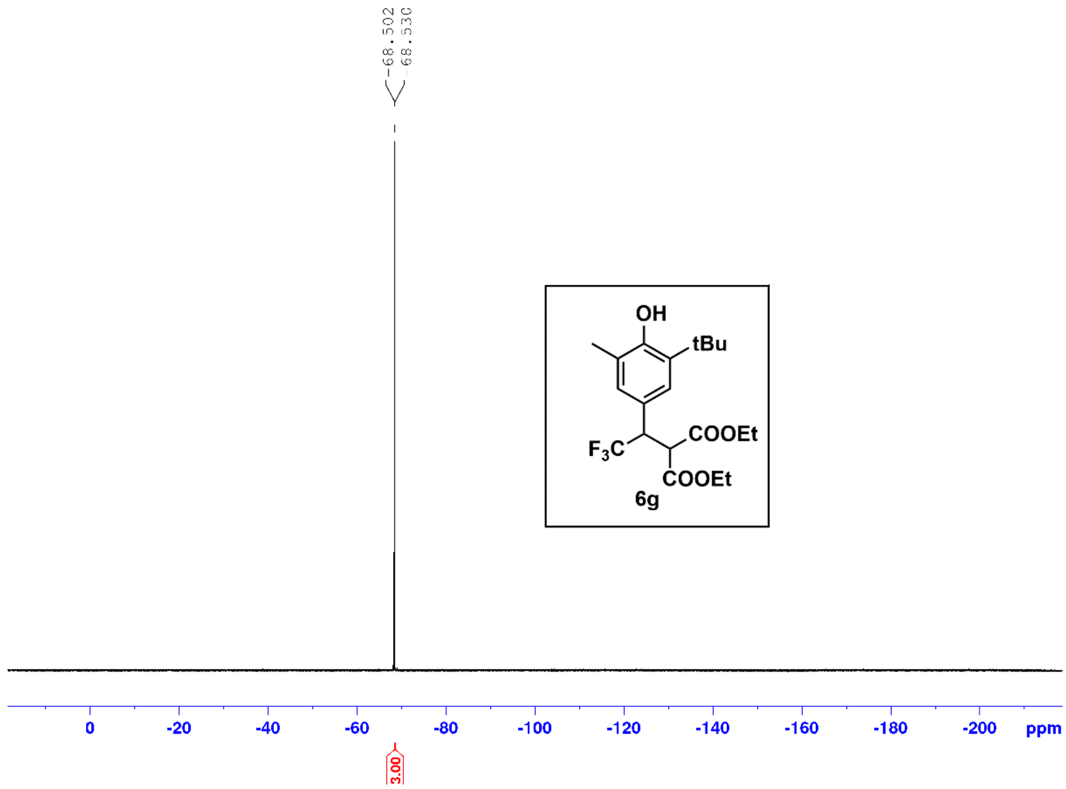
-68.502
68.530
OH
tBu
F3C
COOEt
COOEt
6g
0 -20 -40 -60 -80 -100 -120 -140 -160 -180 -200 ppm
3.00

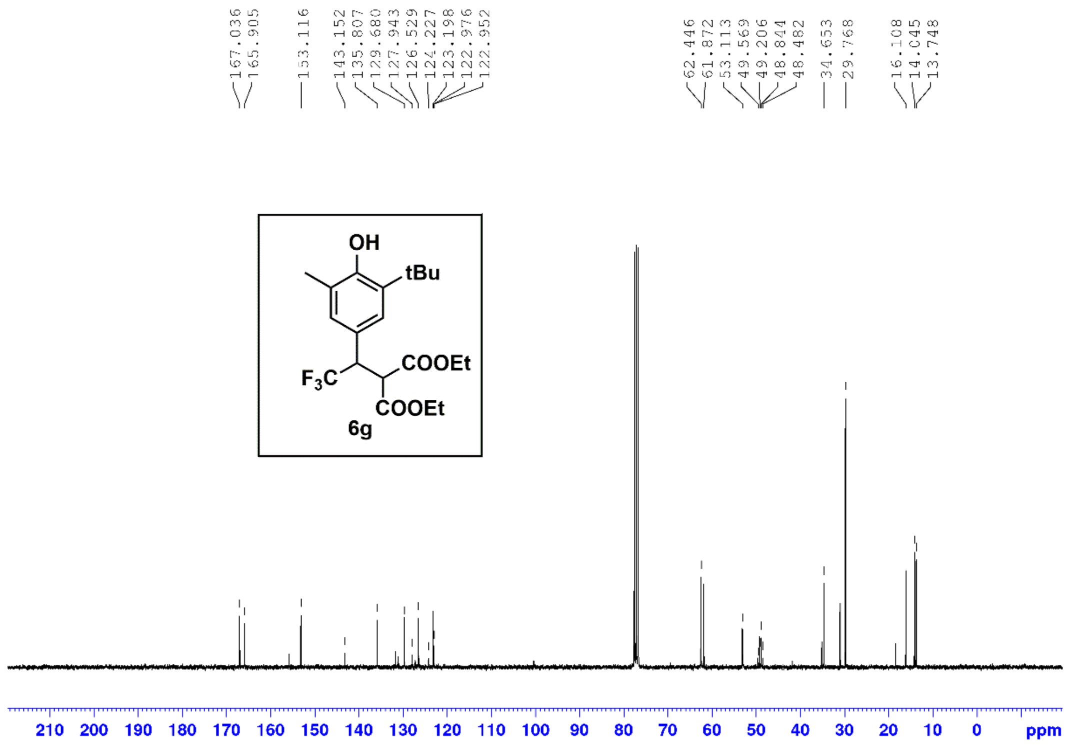
167.036
165.905
153.116
143.152
135.807
129.680
127.943
126.529
124.227
123.198
122.976
122.952
62.446
61.872
53.113
49.569
49.206
48.844
48.482
34.653
29.768
16.108
14.045
13.748
OH
tBu
F3C
COOEt
COOEt
6g
210 200 190 180 170 160 150 140 130 120 110 100 90 80 70 60 50 40 30 20 10 0 ppm

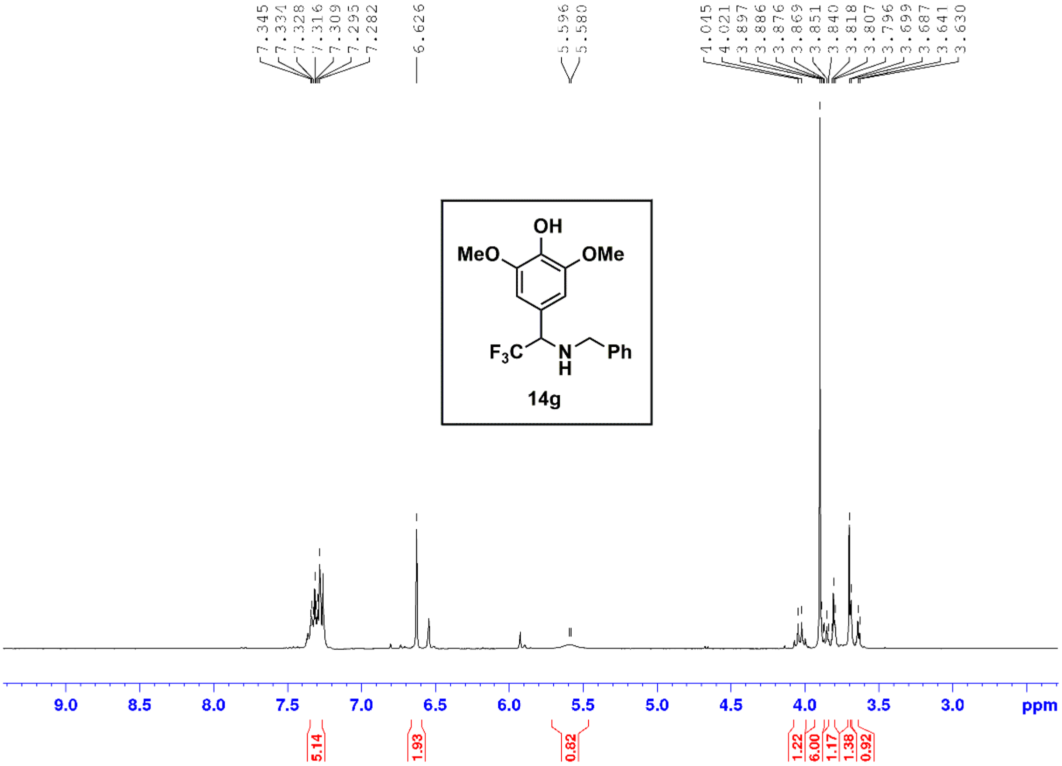
7.345
7.334
7.328
7.316
7.309
7.295
7.282
6.626
5.596
5.580
4.045
4.021
3.897
3.886
3.876
3.869
3.851
3.840
3.818
3.807
3.796
3.699
3.687
3.641
3.630
OH
MeO
OMe
F3C
N
H
Ph
14g
9.0
8.5
8.0
7.5
7.0
6.5
6.0
5.5
5.0
4.5
4.0
3.5
3.0
ppm
5.14
1.93
0.82
1.22
6.00
1.17
1.38
0.92

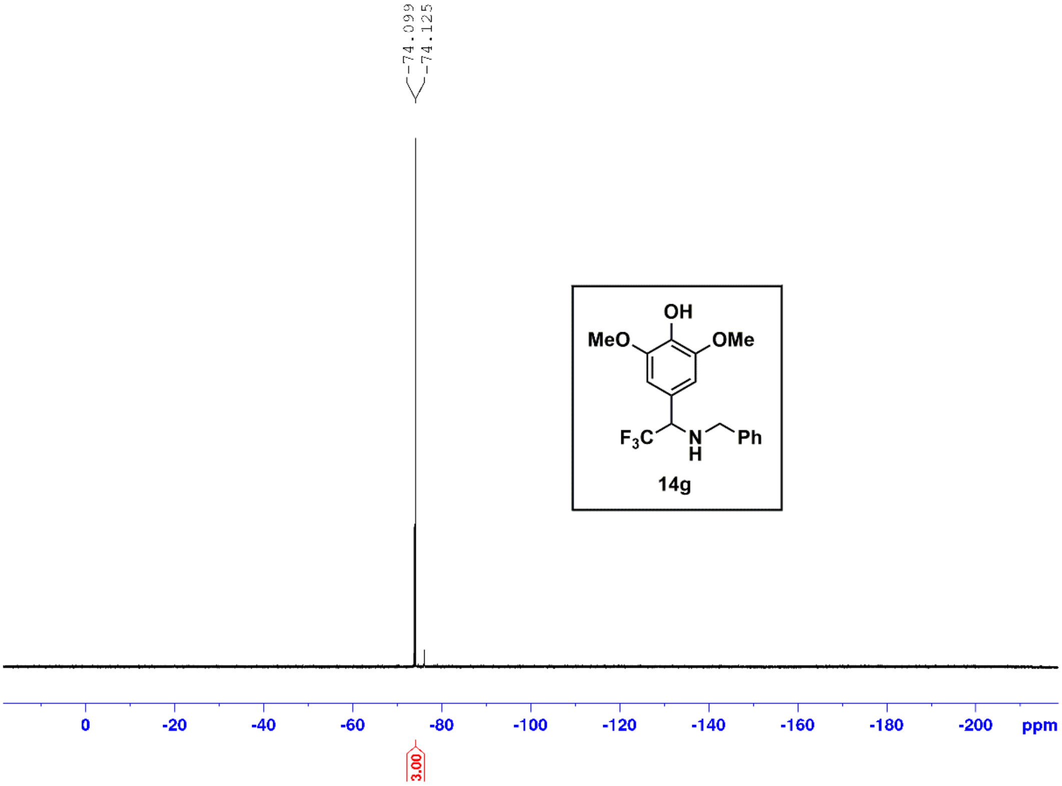
-74.099
-74.125
OH
MeO
OMe
F3C
N
H
Ph
14g
0
-20
-40
-60
-80
-100
-120
-140
-160
-180
-200
ppm
3.00

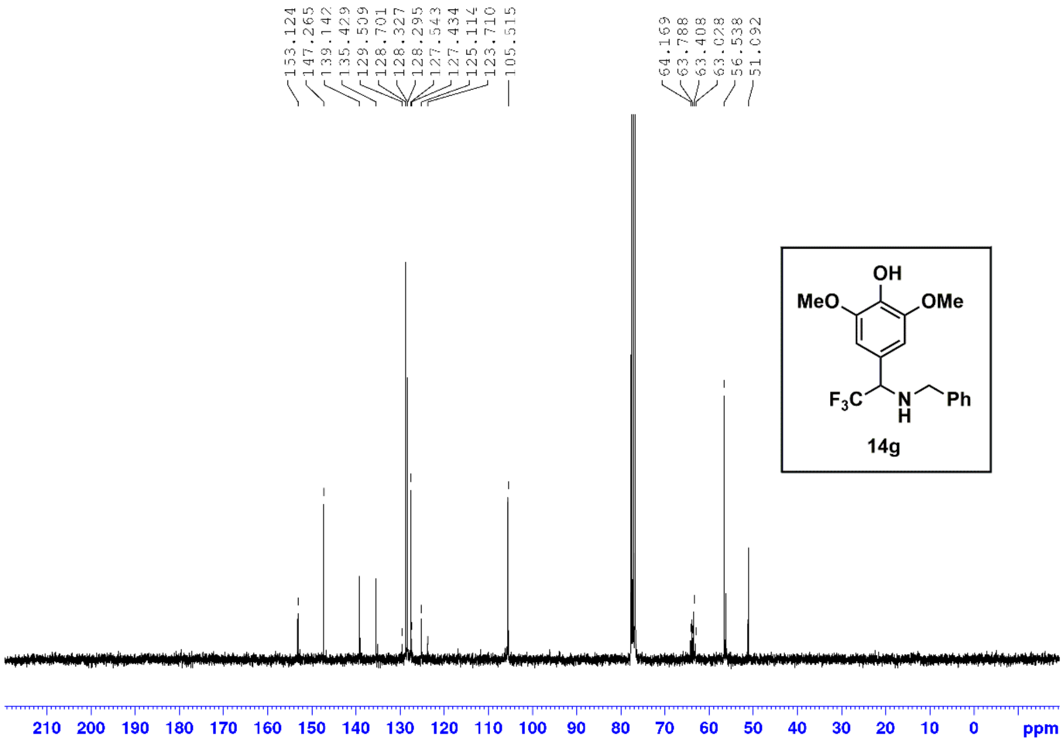
153.124
147.265
139.142
135.429
129.509
128.701
128.327
128.295
127.543
127.434
125.114
123.710
105.515
64.169
63.788
63.408
63.028
56.538
51.092
OH
MeO
OMe
F3C
N
H
Ph
14g
210 200 190 180 170 160 150 140 130 120 110 100 90 80 70 60 50 40 30 20 10 0 ppm

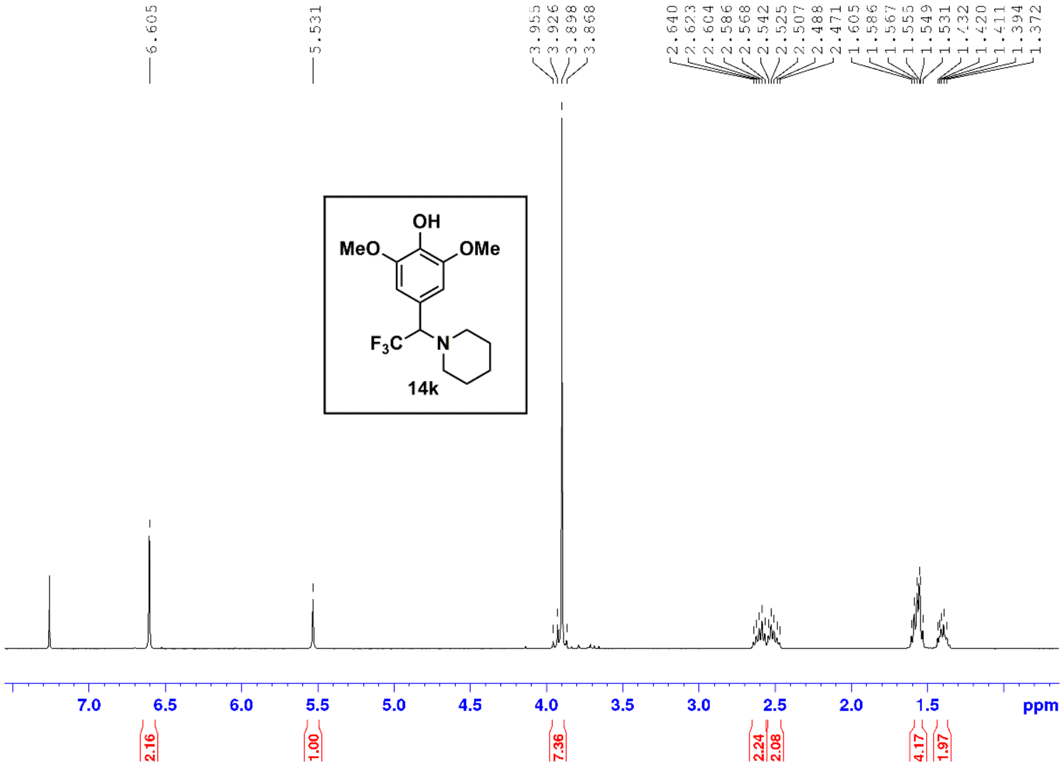
6.605
5.531
3.955
3.926
3.898
3.868
2.640
2.623
2.604
2.586
2.568
2.542
2.525
2.507
2.488
2.471
1.605
1.586
1.567
1.555
1.549
1.531
1.432
1.420
1.411
1.394
1.372
OH
MeO
OMe
F3C
N
14k
7.0 6.5 6.0 5.5 5.0 4.5 4.0 3.5 3.0 2.5 2.0 1.5 ppm
2.16
1.00
7.36
2.24
2.08
4.17
1.97

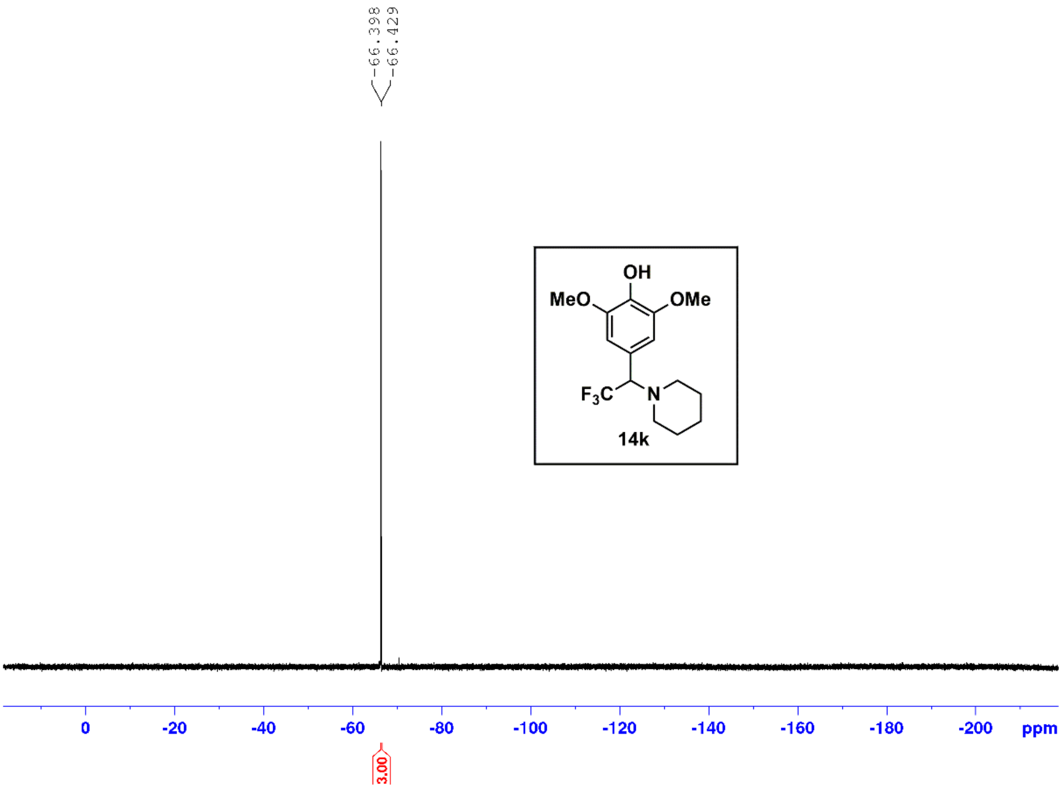
-66.398
-66.429
OH
MeO
OMe
F3C
N
14k
0
-20
-40
-60
-80
-100
-120
-140
-160
-180
-200
ppm
3.00

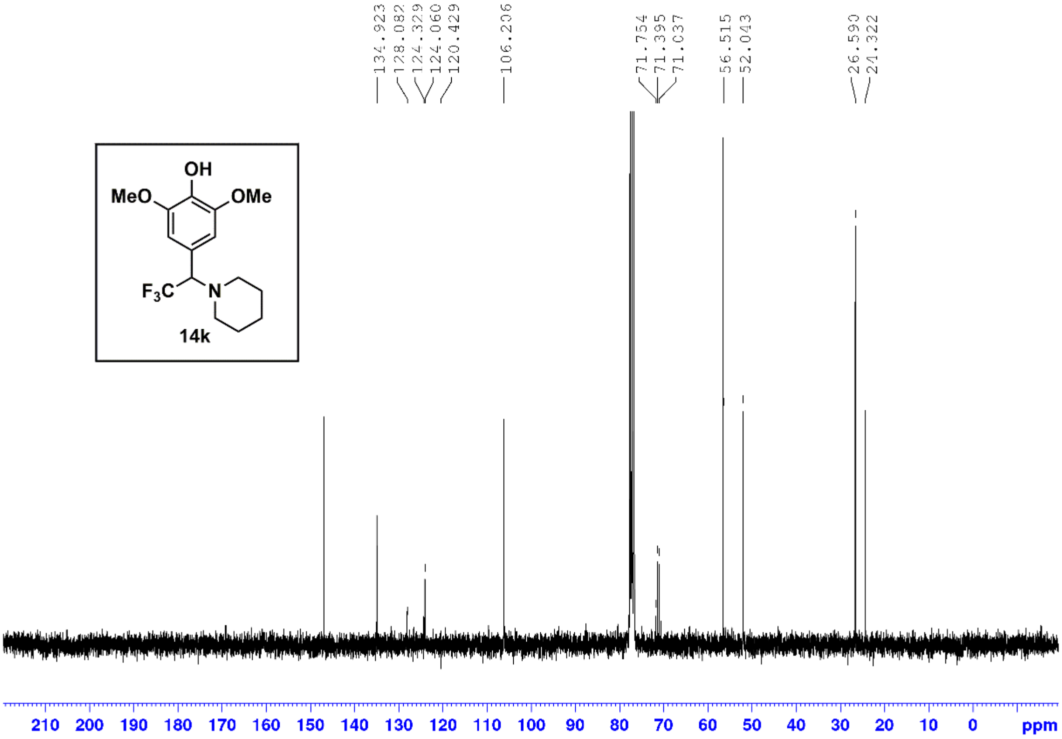
134.923
128.082
124.329
124.060
120.429
106.206
71.754
71.395
71.037
56.515
52.043
26.590
24.322
OH
MeO
OMe
F3C
N
14k
210
200
190
180
170
160
150
140
130
120
110
100
90
80
70
60
50
40
30
20
10
0
ppm

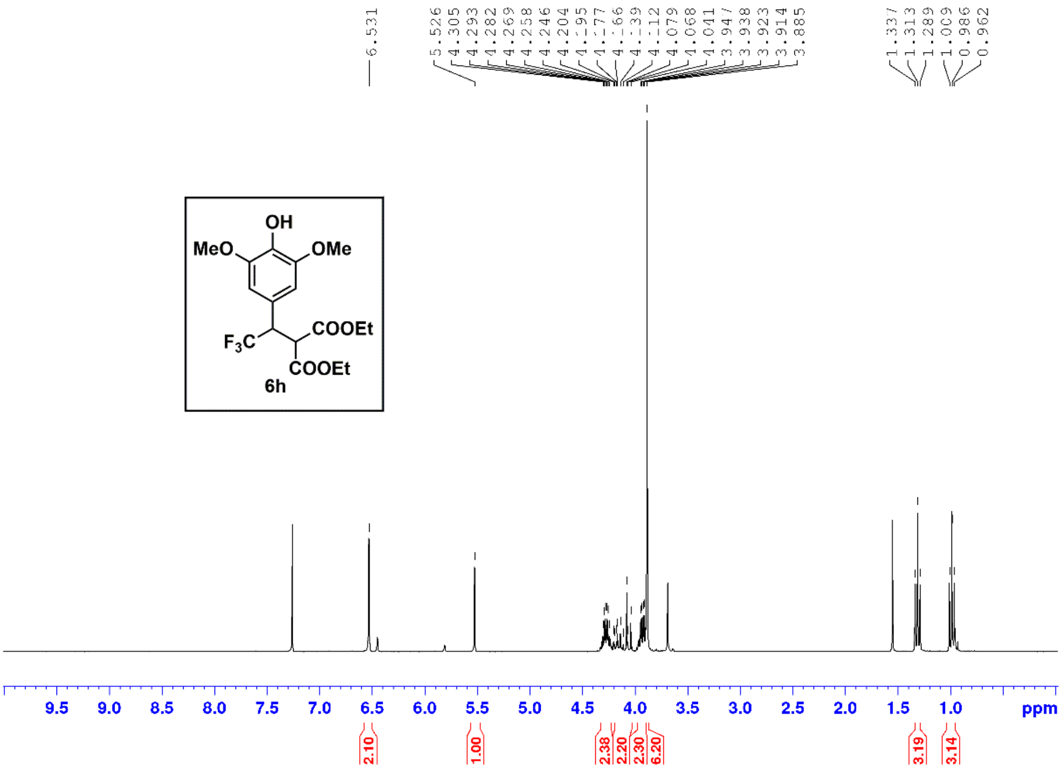
6h
ppm

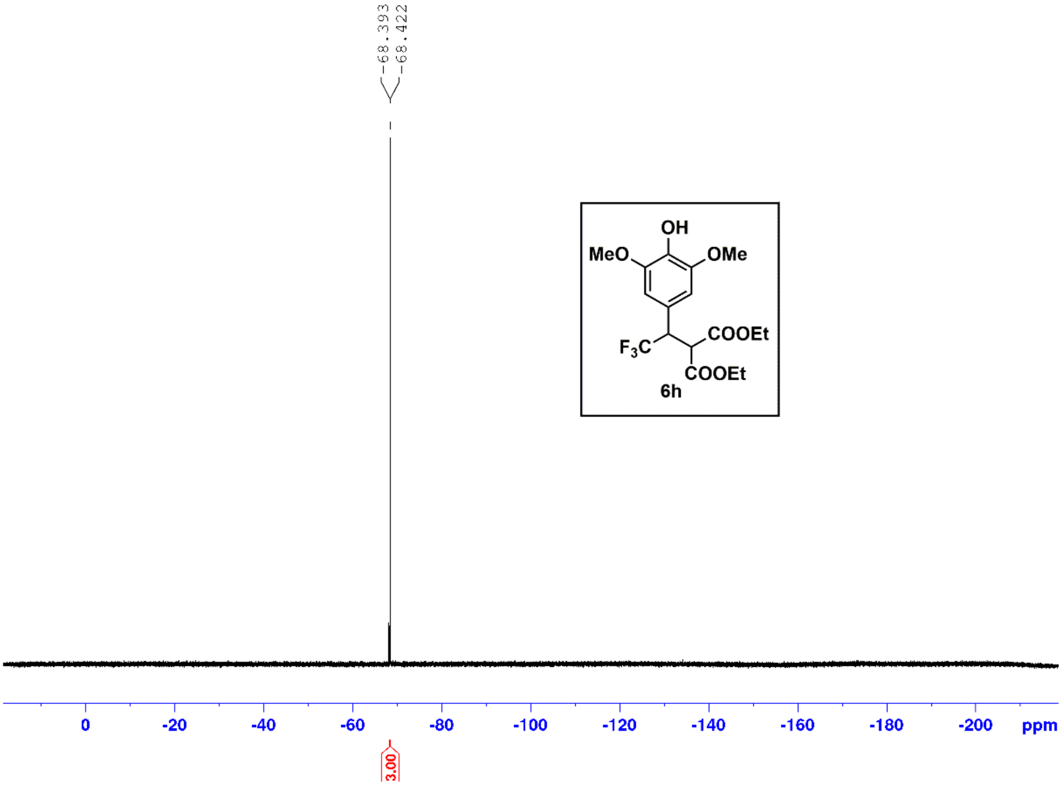
-68.393
-68.422
6h
ppm

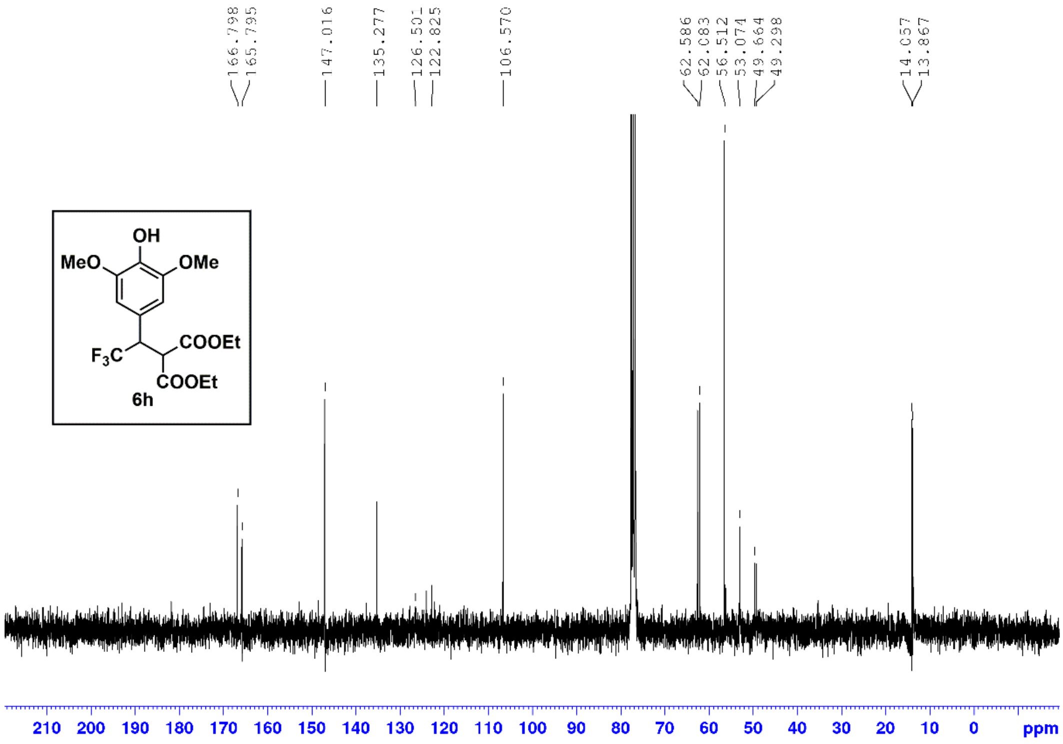
166.798
165.795
147.016
135.277
126.591
122.825
106.570
62.586
62.083
56.512
53.074
49.664
49.298
14.057
13.867
OH
MeO
OMe
F_3C
COOEt
COOEt
6h
210 200 190 180 170 160 150 140 130 120 110 100 90 80 70 60 50 40 30 20 10 0 ppm

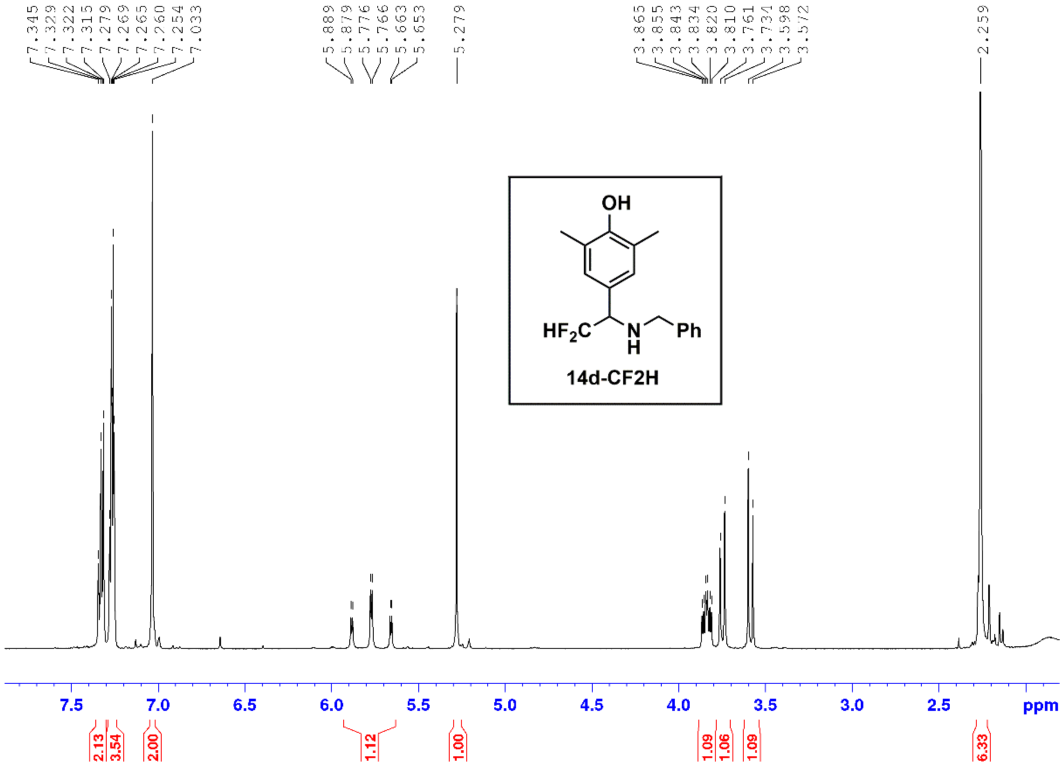
7.345
7.329
7.322
7.315
7.279
7.269
7.265
7.260
7.254
7.033
5.889
5.879
5.776
5.766
5.663
5.653
5.279
3.865
3.855
3.843
3.834
3.820
3.810
3.761
3.734
3.598
3.572
2.259
OH
HF_2C
N
H
Ph
14d-CF2H
7.5 7.0 6.5 6.0 5.5 5.0 4.5 4.0 3.5 3.0 2.5 ppm
2.13
3.54
2.00
1.12
1.00
1.09
1.06
1.09
6.33

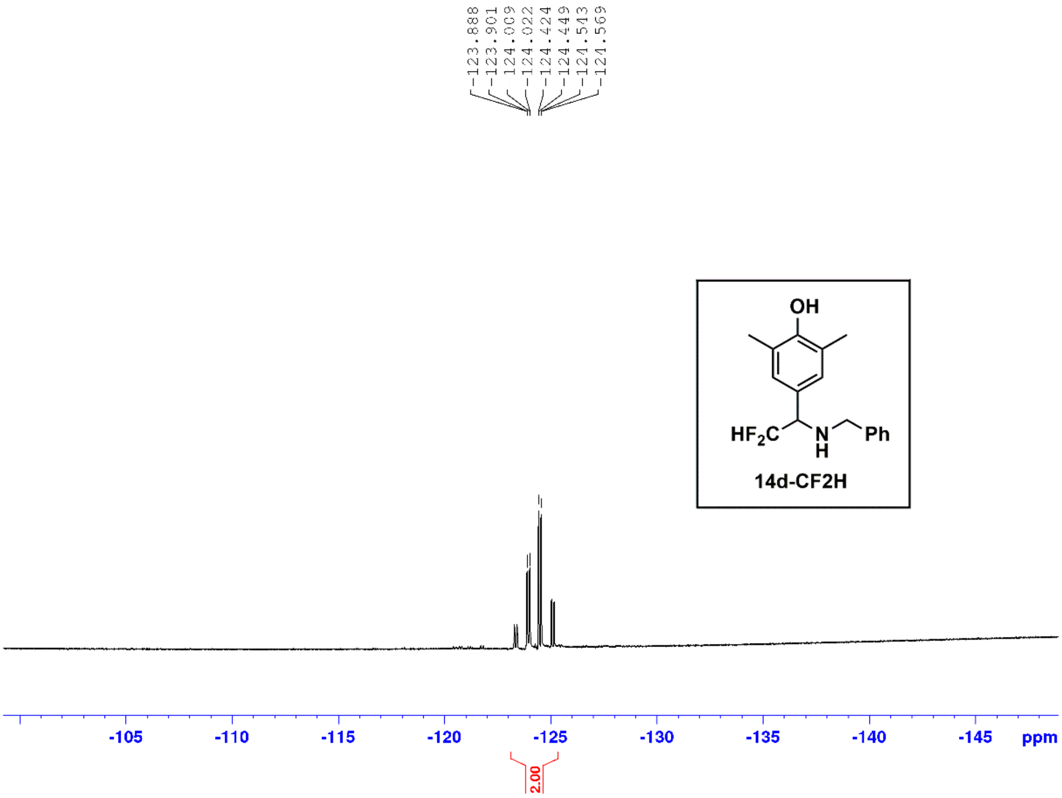
-123.888
-123.901
-124.009
-124.022
-124.424
-124.449
-124.543
-124.569
OH
HF2C
N
H
Ph
14d-CF2H
-105
-110
-115
-120
-125
-130
-135
-140
-145
ppm
2.00

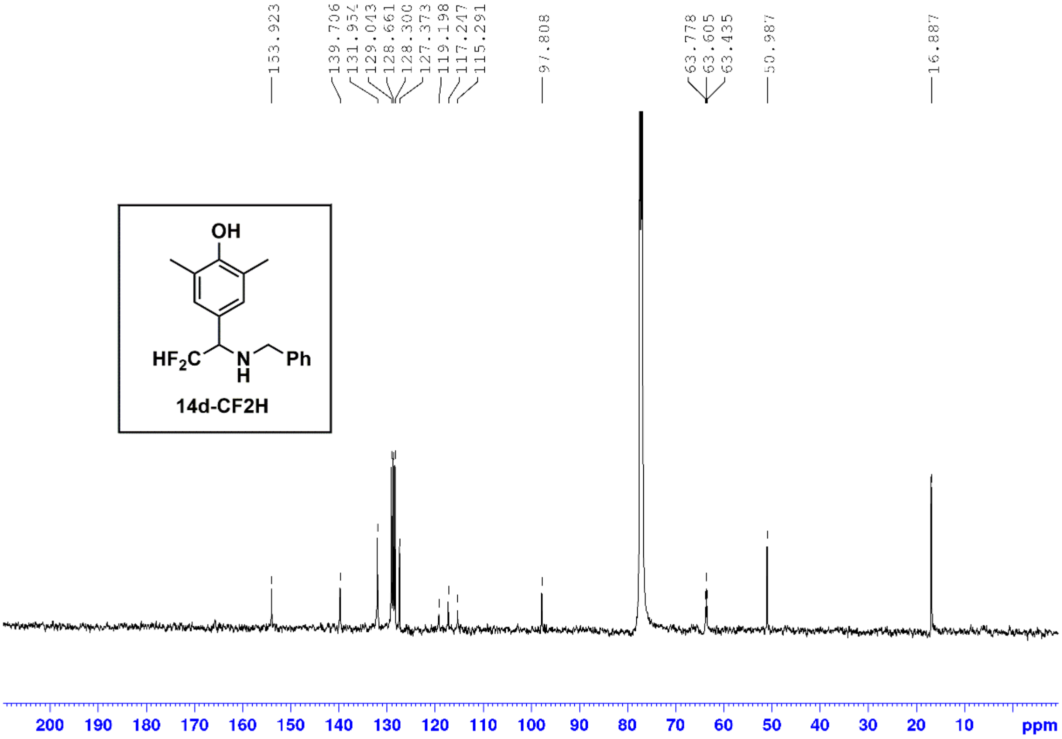
153.923
139.706
131.954
129.043
128.661
128.300
127.373
119.198
117.247
115.291
97.808
63.778
63.605
63.435
50.987
16.887
OH
HF2C
N
H
Ph
14d-CF2H
200
190
180
170
160
150
140
130
120
110
100
90
80
70
60
50
40
30
20
10
ppm

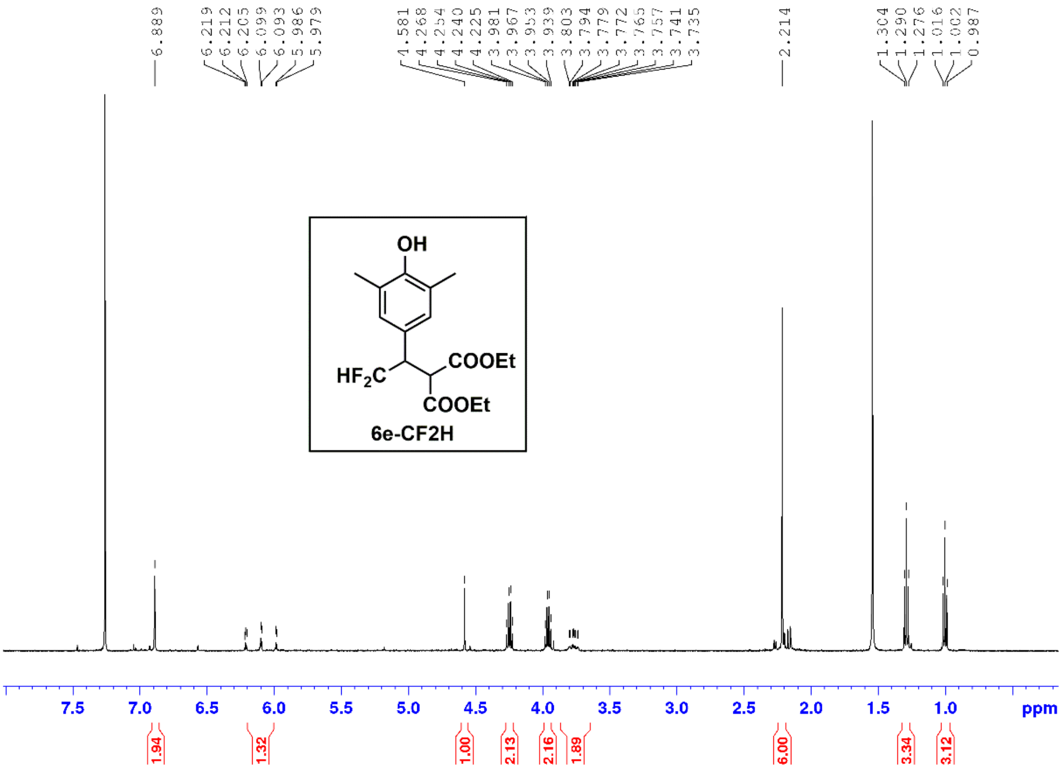
6.889
6.219
6.212
6.205
6.099
6.093
5.986
5.979
4.581
4.268
4.254
4.240
4.225
3.981
3.967
3.953
3.939
3.803
3.794
3.779
3.772
3.765
3.757
3.741
3.735
2.214
1.304
1.290
1.276
1.016
1.002
0.987
OH
HF2C
COOEt
COOEt
6e-CF2H
7.5
7.0
6.5
6.0
5.5
5.0
4.5
4.0
3.5
3.0
2.5
2.0
1.5
1.0
ppm
1.94
1.32
1.00
2.13
2.16
1.89
6.00
3.34
3.12

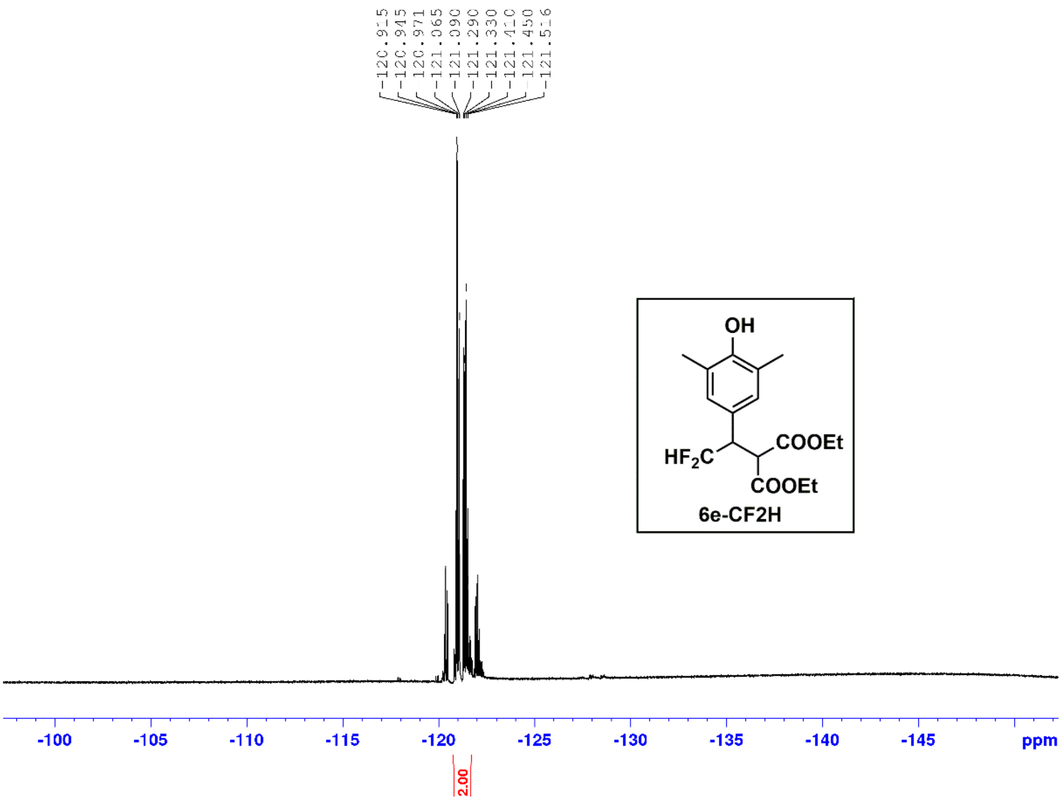
OH
HF2C
COOEt
COOEt
6e-CF2H
-100
-105
-110
-115
-120
-125
-130
-135
-140
-145
ppm
2.00

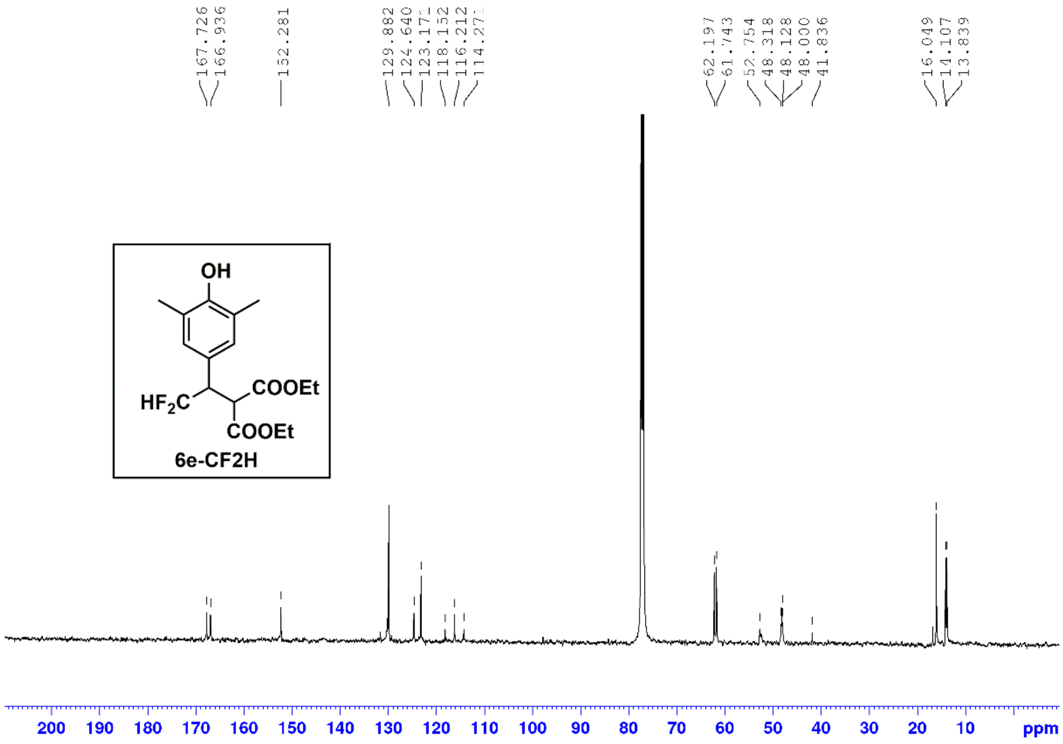
6e-CF2H
OH
COOEt
COOEt
167.726
166.936
152.281
129.882
124.640
62.197
61.743
52.754
48.318
48.128
48.000
41.836
16.049
14.107
13.839
ppm

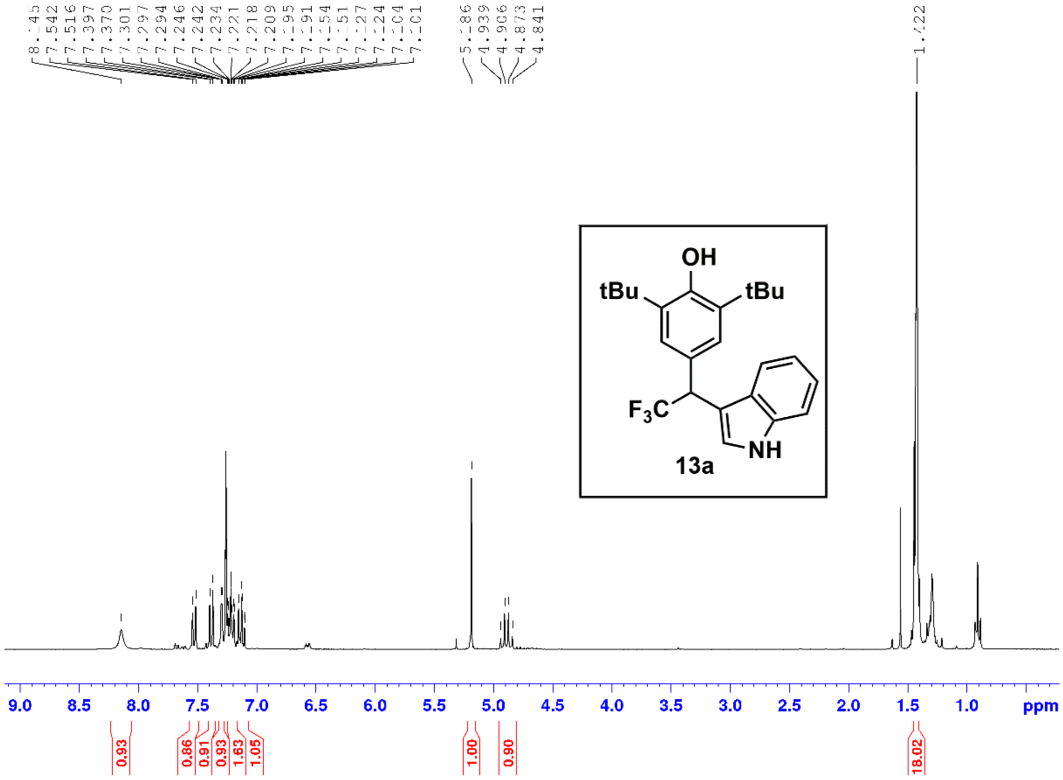
13a
OH
tBu
tBu
NH
7.542
7.516
7.397
7.370
7.301
7.297
7.294
7.246
7.242
7.234
7.221
7.218
7.209
4.939
4.906
4.873
4.841
ppm
0.93
0.86
0.91
0.93
1.63
1.05
1.00
0.90
18.02

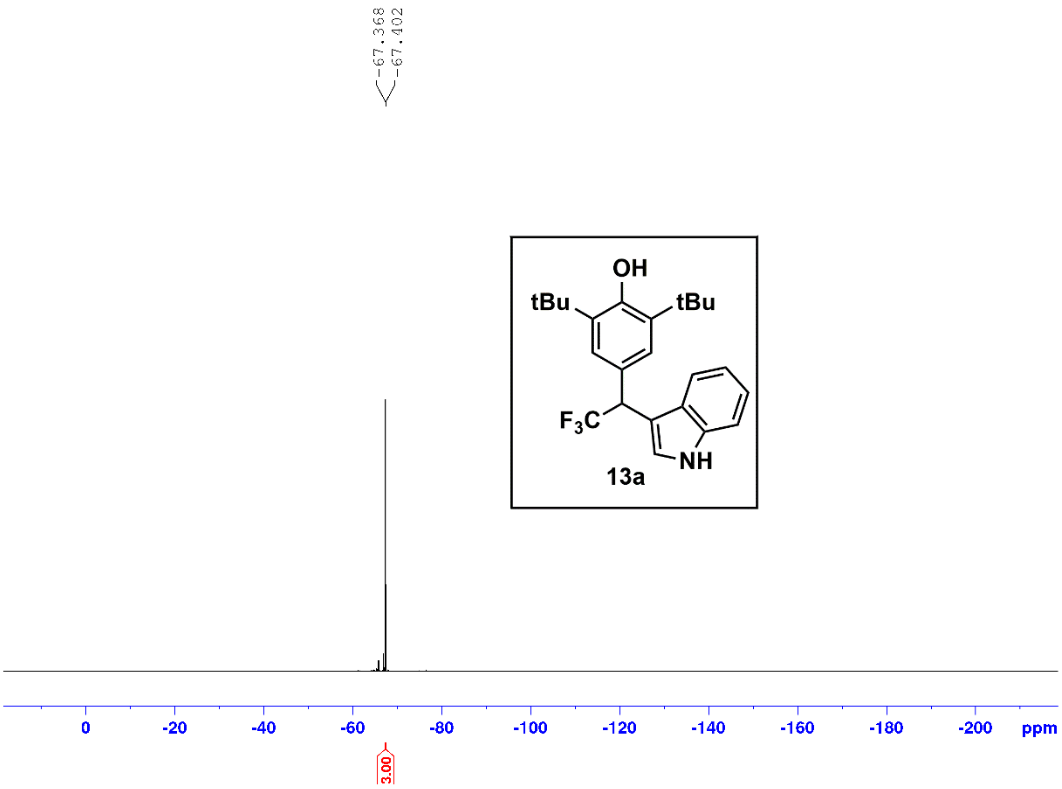
-67.368
-67.402
OH
tBu
tBu
F3C
NH
13a
0
-20
-40
-60
-80
-100
-120
-140
-160
-180
-200
ppm
3.00

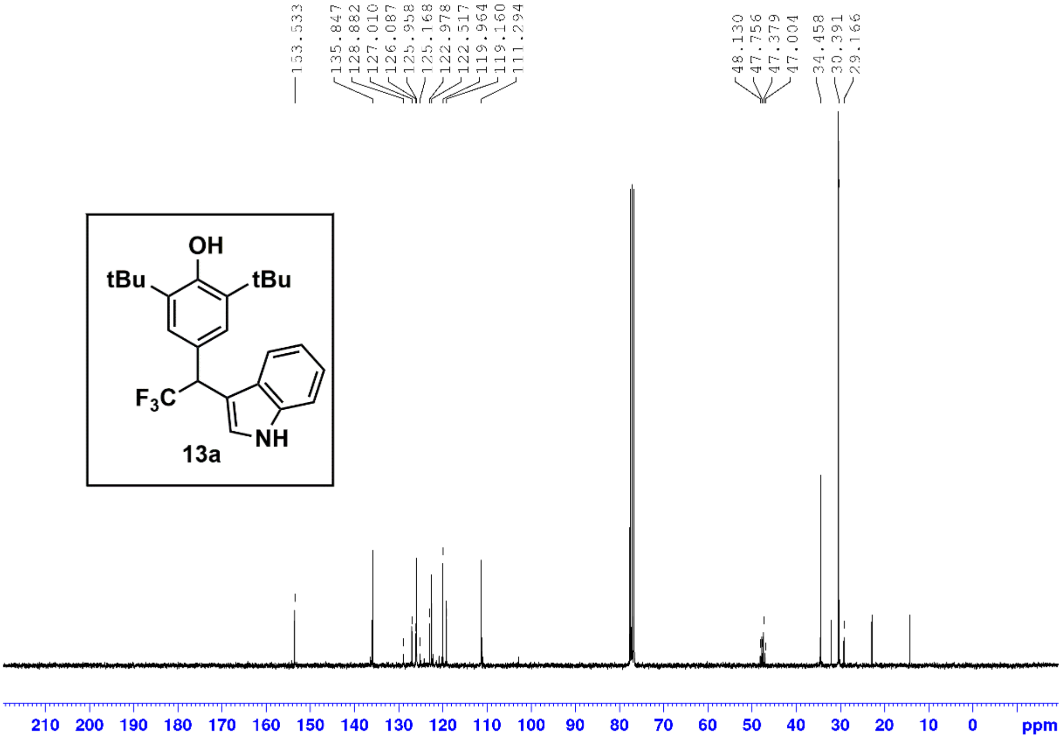
153.533
135.847
128.882
127.010
126.087
125.958
125.168
122.978
122.517
119.964
119.160
111.294
48.130
47.756
47.379
47.004
34.458
30.391
29.166
OH
tBu
tBu
F3C
NH
13a
210
200
190
180
170
160
150
140
130
120
110
100
90
80
70
60
50
40
30
20
10
0
ppm

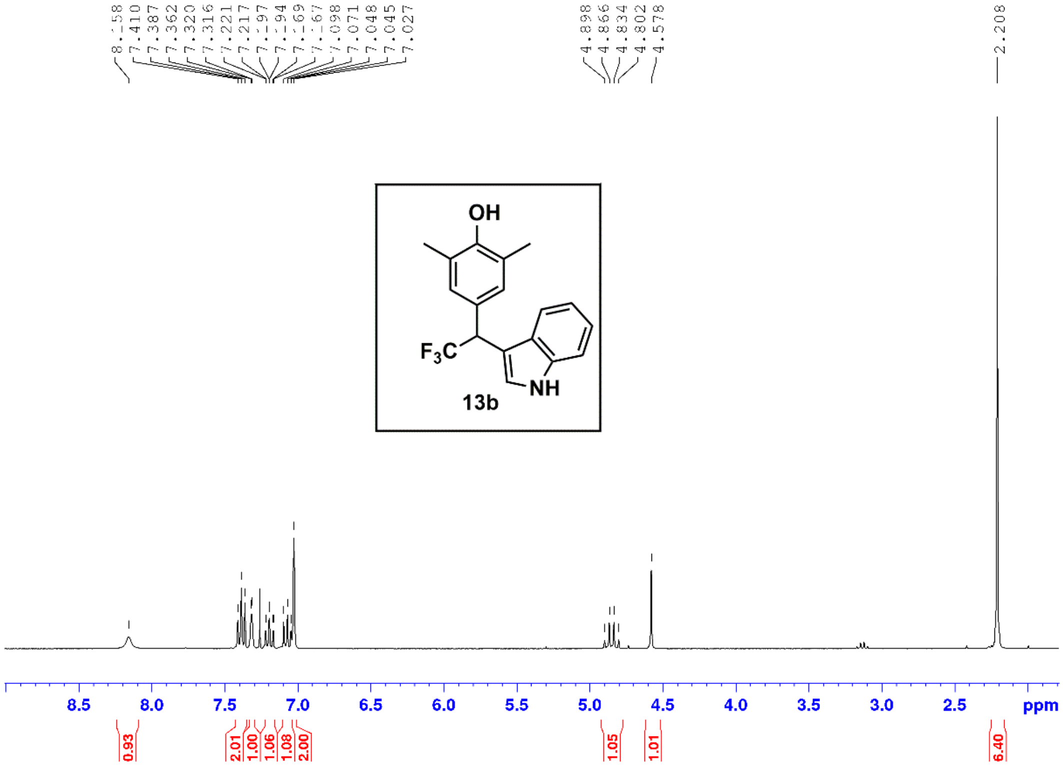
8.158
7.410
7.387
7.362
7.320
7.316
7.221
7.217
7.197
7.194
7.169
7.167
7.098
7.071
7.048
7.045
7.027
4.898
4.866
4.834
4.802
4.578
2.208
OH
F3C
NH
13b
8.5
8.0
7.5
7.0
6.5
6.0
5.5
5.0
4.5
4.0
3.5
3.0
2.5
ppm
0.93
2.01
1.00
1.06
1.08
2.00
1.05
1.01
6.40

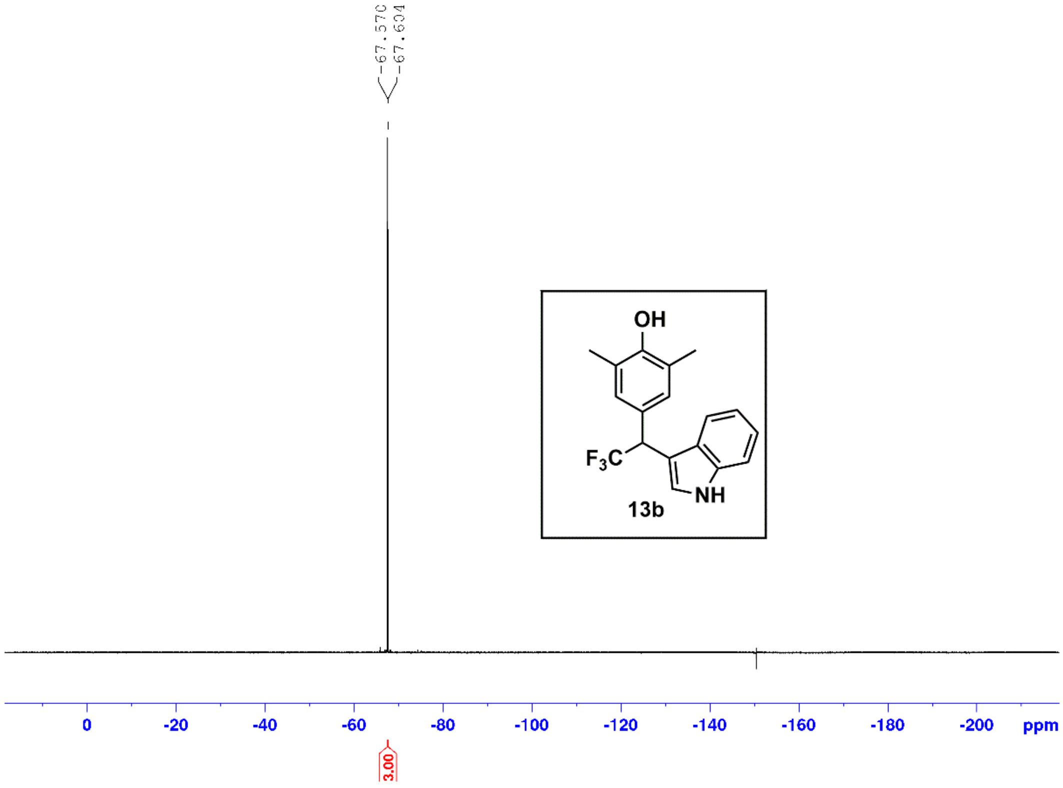
-67.570
-67.604
OH
F3C
NH
13b
0
-20
-40
-60
-80
-100
-120
-140
-160
-180
-200
ppm
3.00

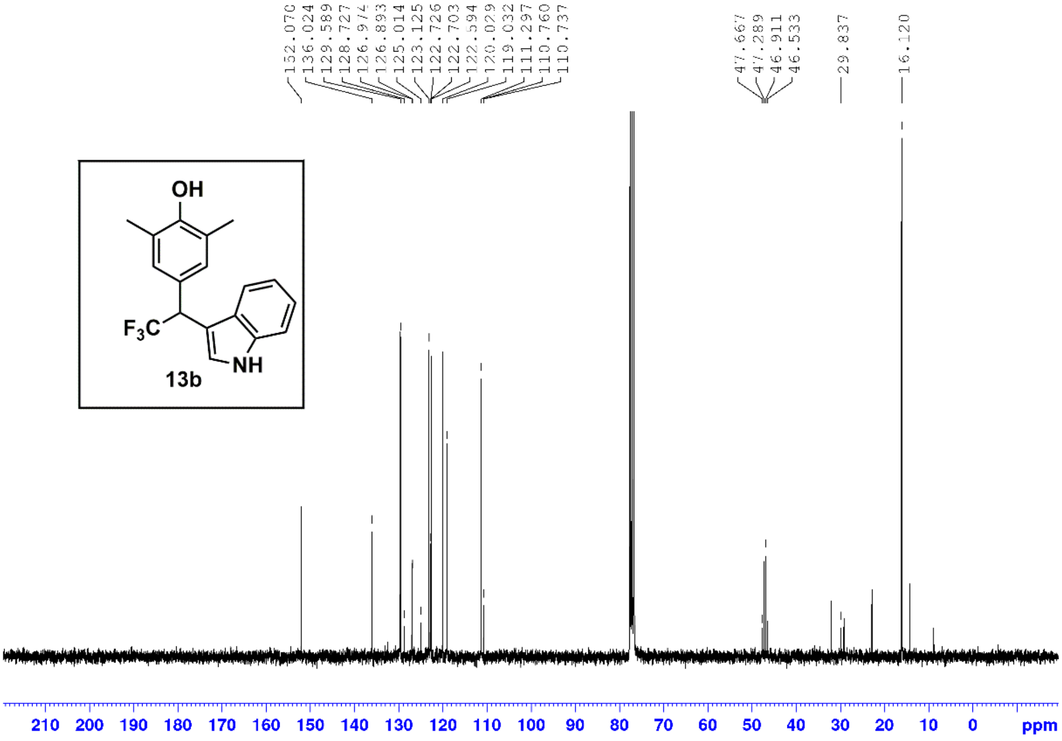
OH
F3C
NH
13b
152.070
136.024
129.589
128.727
126.974
126.893
125.014
123.125
122.726
122.703
120.029
119.032
111.297
110.760
110.737
47.667
47.289
46.911
46.533
29.837
16.120
210 200 190 180 170 160 150 140 130 120 110 100 90 80 70 60 50 40 30 20 10 0 ppm

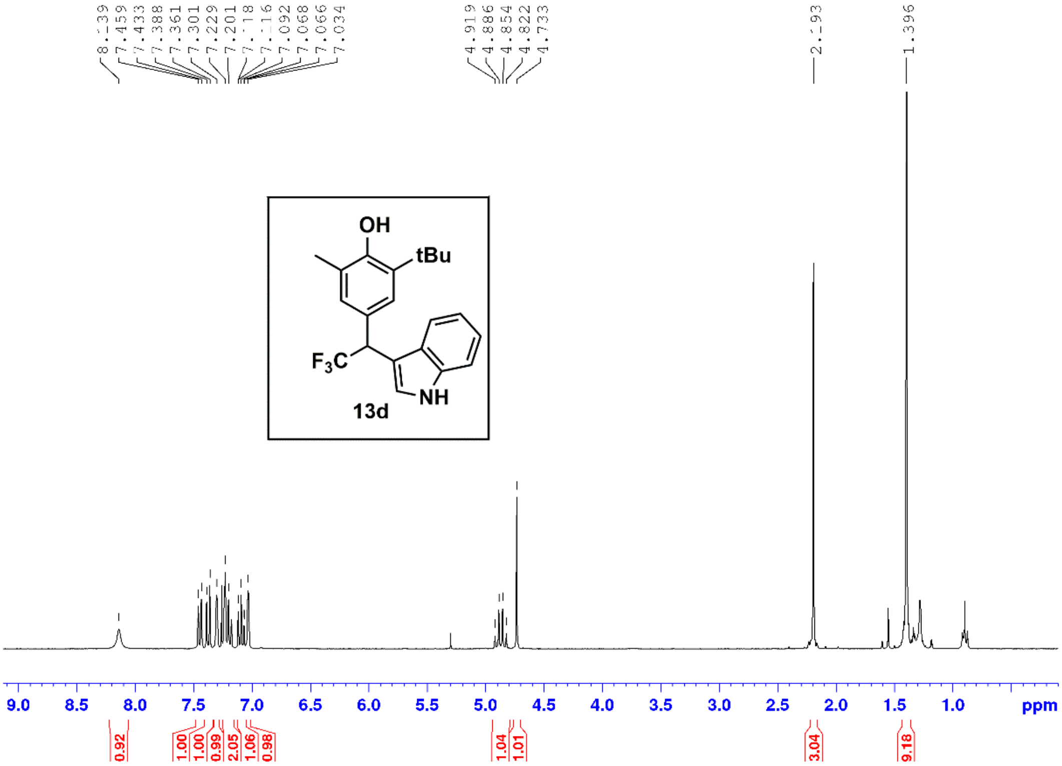
OH
tBu
F3C
NH
13d
7.459
7.433
7.388
7.361
7.301
7.229
7.201
7.092
7.068
7.066
7.034
4.919
4.886
4.854
4.822
4.733
1.396
9.0 8.5 8.0 7.5 7.0 6.5 6.0 5.5 5.0 4.5 4.0 3.5 3.0 2.5 2.0 1.5 1.0 ppm

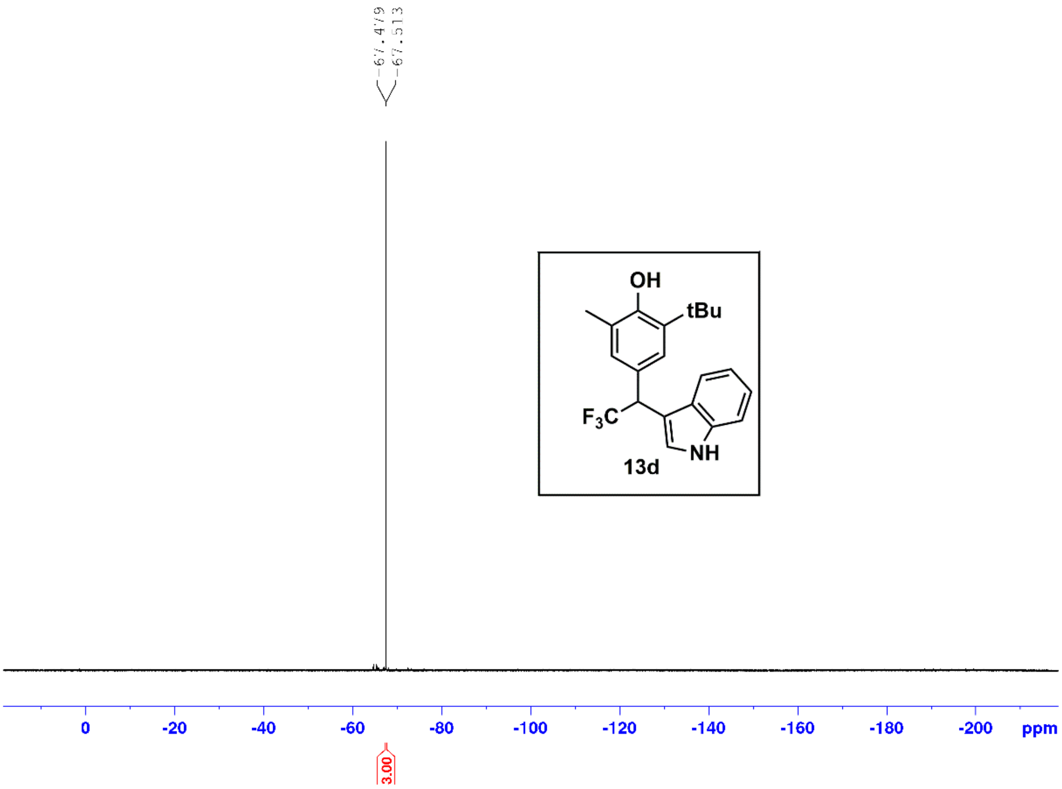
-67.479
-67.513
OH
tBu
F3C
NH
13d
0
-20
-40
-60
-80
-100
-120
-140
-160
-180
-200
ppm
3.00

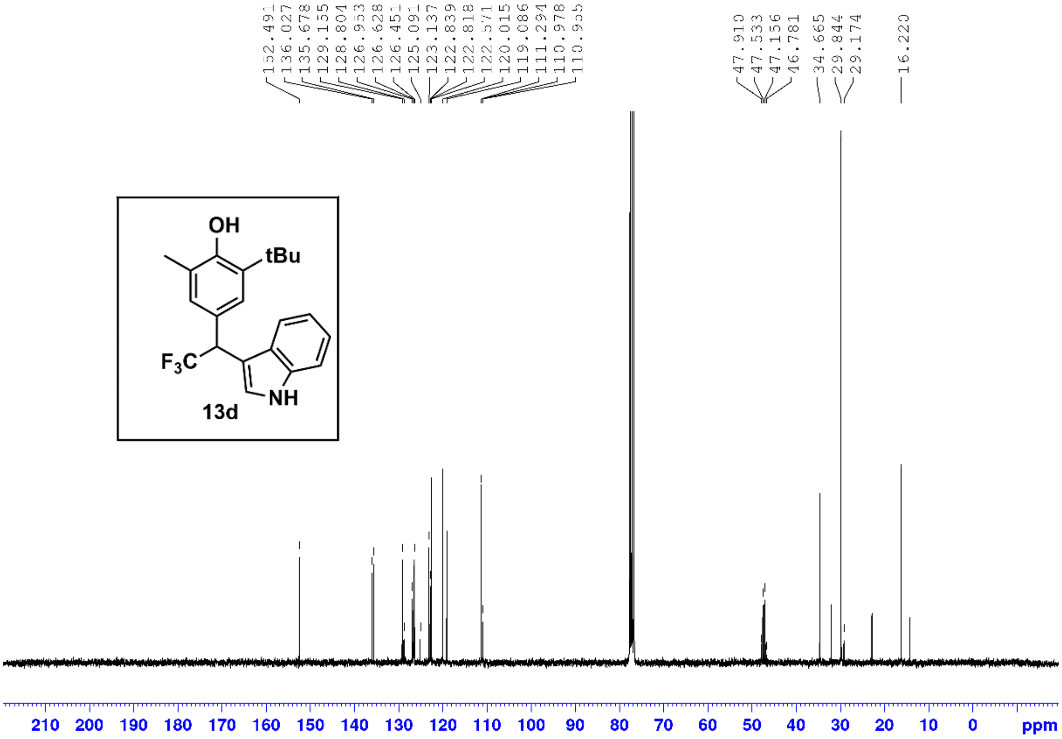
152.491
136.027
135.678
129.155
128.804
126.953
126.628
126.451
125.091
123.137
122.839
122.818
122.571
120.015
119.086
111.294
110.978
110.955
47.910
47.533
47.156
46.781
34.665
29.844
29.174
16.220
OH
tBu
F3C
NH
13d
210
200
190
180
170
160
150
140
130
120
110
100
90
80
70
60
50
40
30
20
10
0
ppm

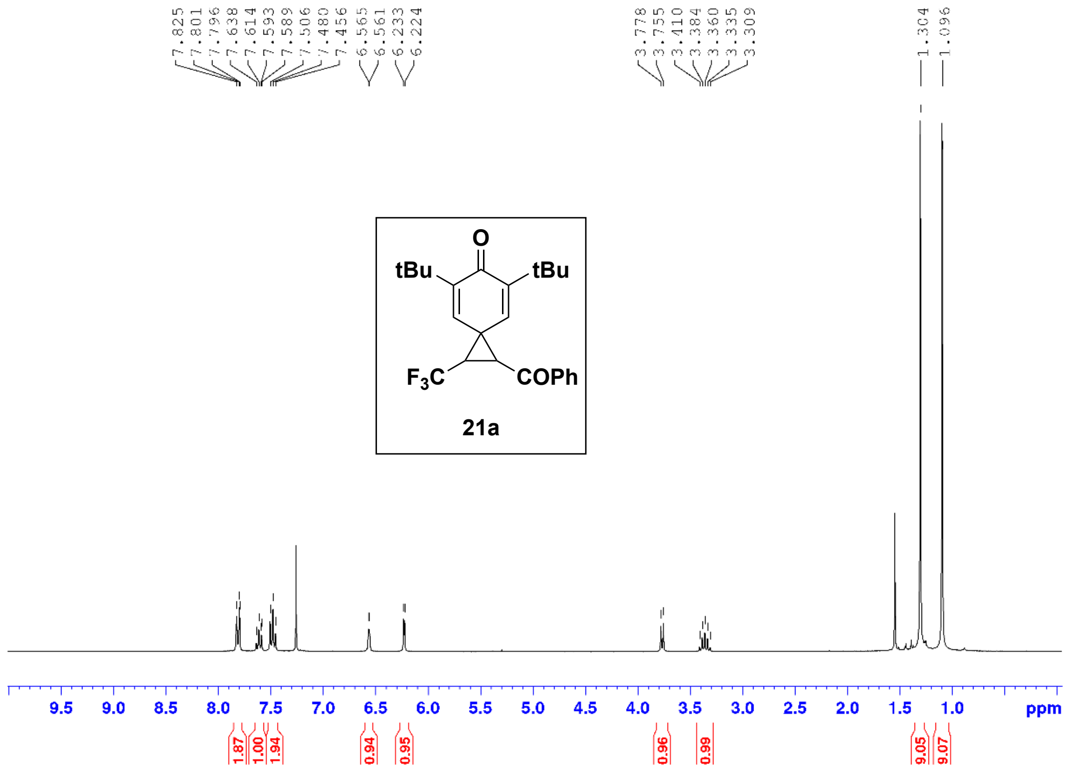
7.825
7.801
7.796
7.638
7.614
7.593
7.589
7.506
7.480
7.456
6.565
6.561
6.233
6.224
3.778
3.755
3.410
3.384
3.360
3.335
3.309
1.304
1.096
O
tBu
tBu
F3C
COPh
21a
9.5 9.0 8.5 8.0 7.5 7.0 6.5 6.0 5.5 5.0 4.5 4.0 3.5 3.0 2.5 2.0 1.5 1.0 ppm
1.87
1.00
1.94
0.94
0.95
0.96
0.99
9.05
9.07

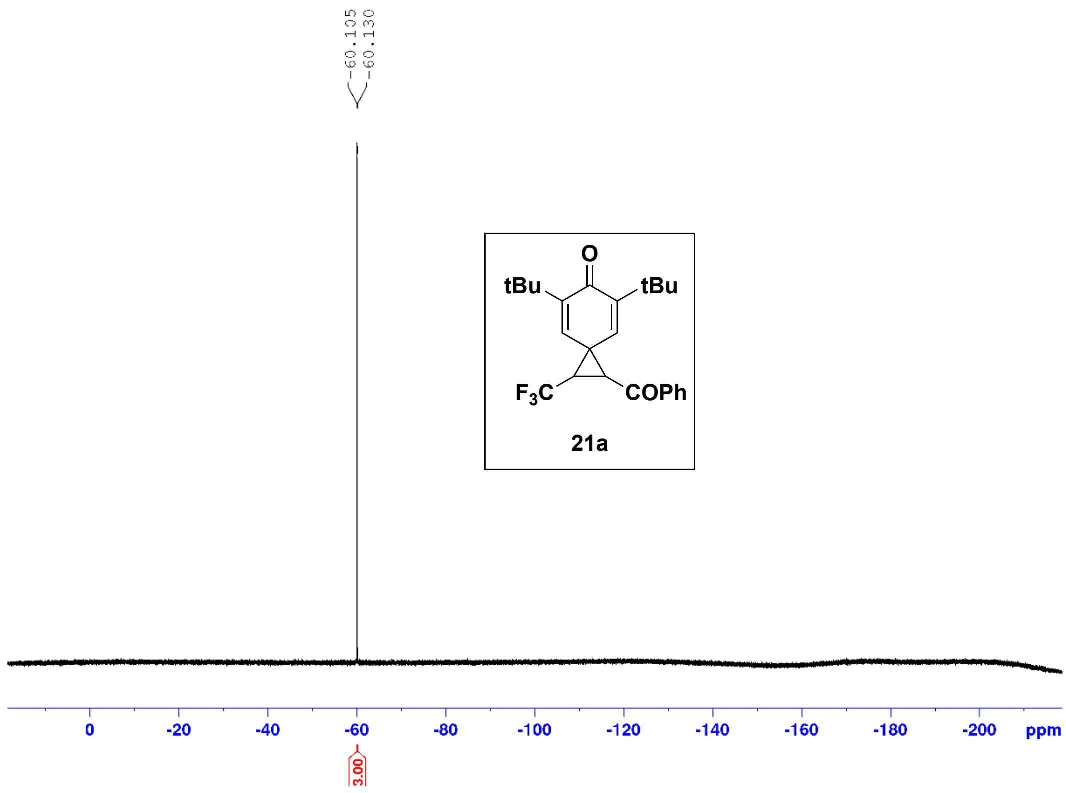
-60.105
-60.130
O
tBu
tBu
F3C
COPh
21a
0 -20 -40 -60 -80 -100 -120 -140 -160 -180 -200 ppm
3.00

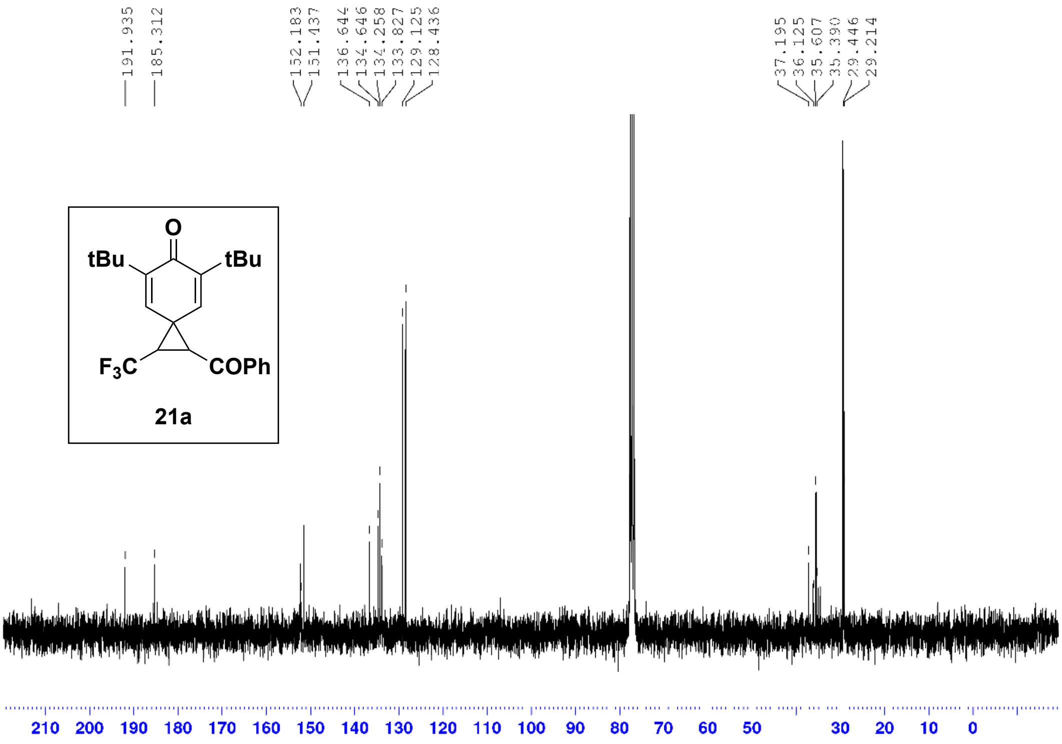
191.935
185.312
152.183
151.437
136.644
134.646
134.258
133.827
129.125
128.436
37.195
36.125
35.607
35.390
29.446
29.214
tBu
O
tBu
F3C
COPh
21a
210 200 190 180 170 160 150 140 130 120 110 100 90 80 70 60 50 30 20 10 0

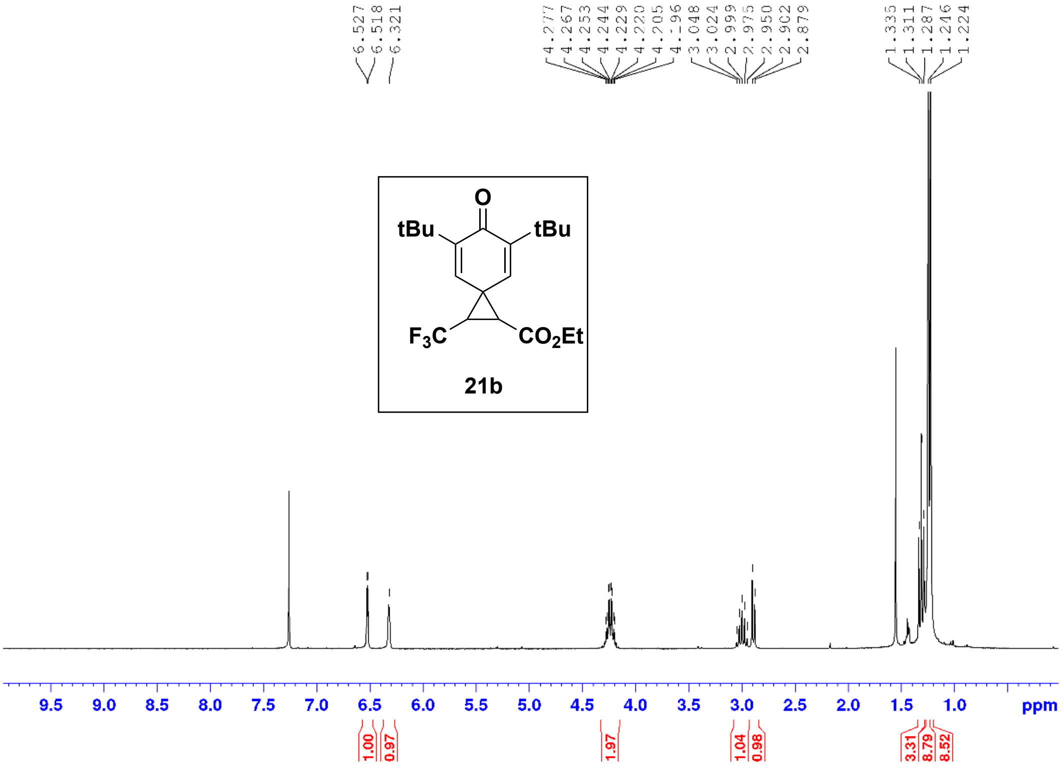
6.527
6.518
6.321
4.277
4.267
4.253
4.244
4.229
4.220
4.205
3.048
3.024
2.999
2.975
2.950
2.902
2.879
1.335
1.311
1.287
1.246
1.224
O
tBu
tBu
F3C
CO2Et
21b
9.5 9.0 8.5 8.0 7.5 7.0 6.5 6.0 5.5 5.0 4.5 4.0 3.5 3.0 2.5 2.0 1.5 1.0 ppm
1.00
0.97
1.97
1.04
0.98
3.31
8.79
8.52

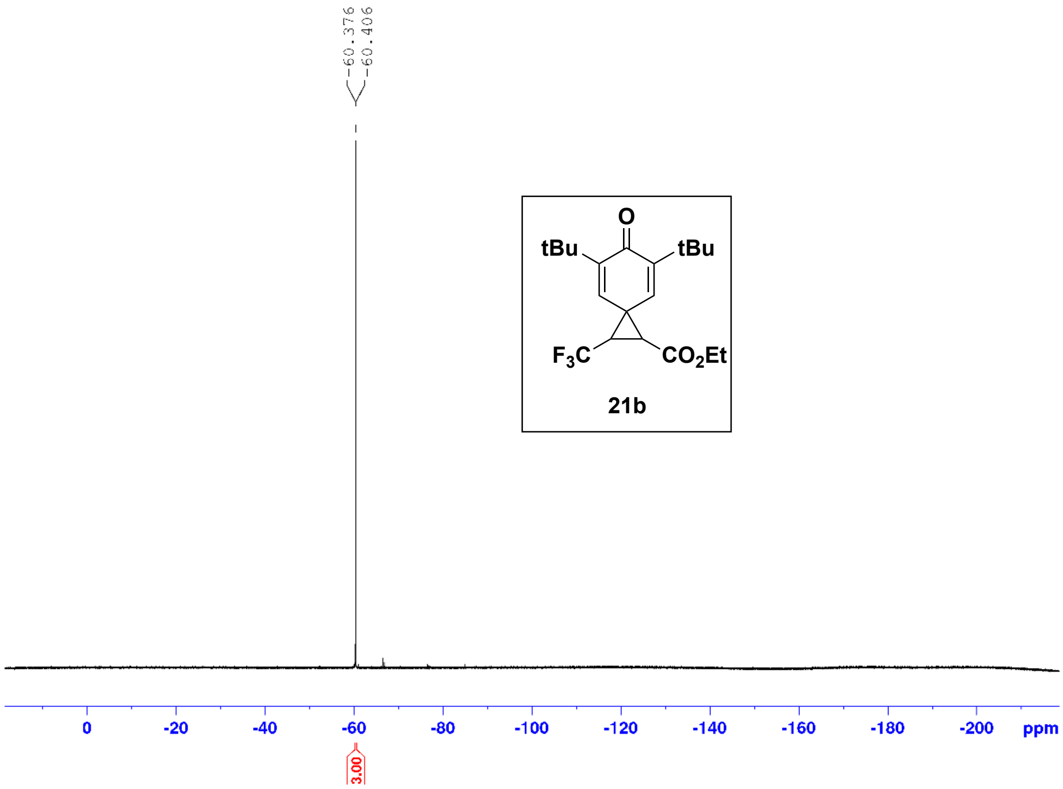
-60.376
-60.406
O
tBu
tBu
F3C
CO2Et
21b
0 -20 -40 -60 -80 -100 -120 -140 -160 -180 -200 ppm
3.00

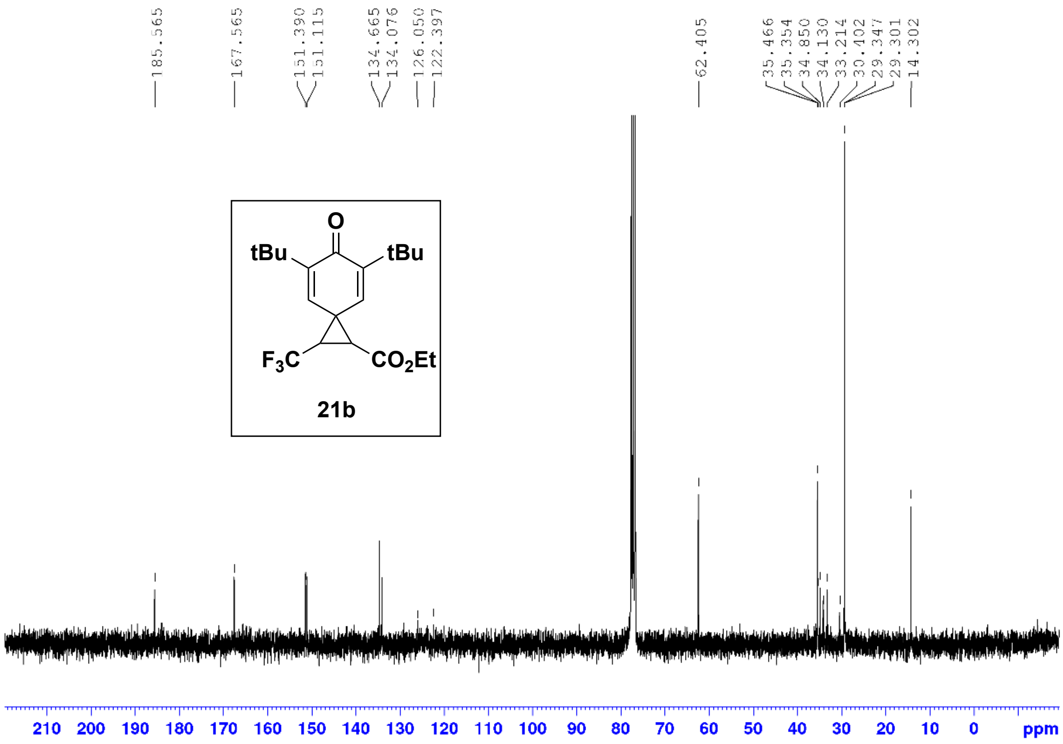
185.565
167.565
151.390
151.115
134.665
134.076
126.050
122.397
62.405
35.466
35.354
34.850
34.130
33.214
30.102
29.347
29.301
14.302
O
tBu
tBu
F3C
CO2Et
21b
210 200 190 180 170 160 150 140 130 120 110 100 90 80 70 60 50 40 30 20 10 0 ppm

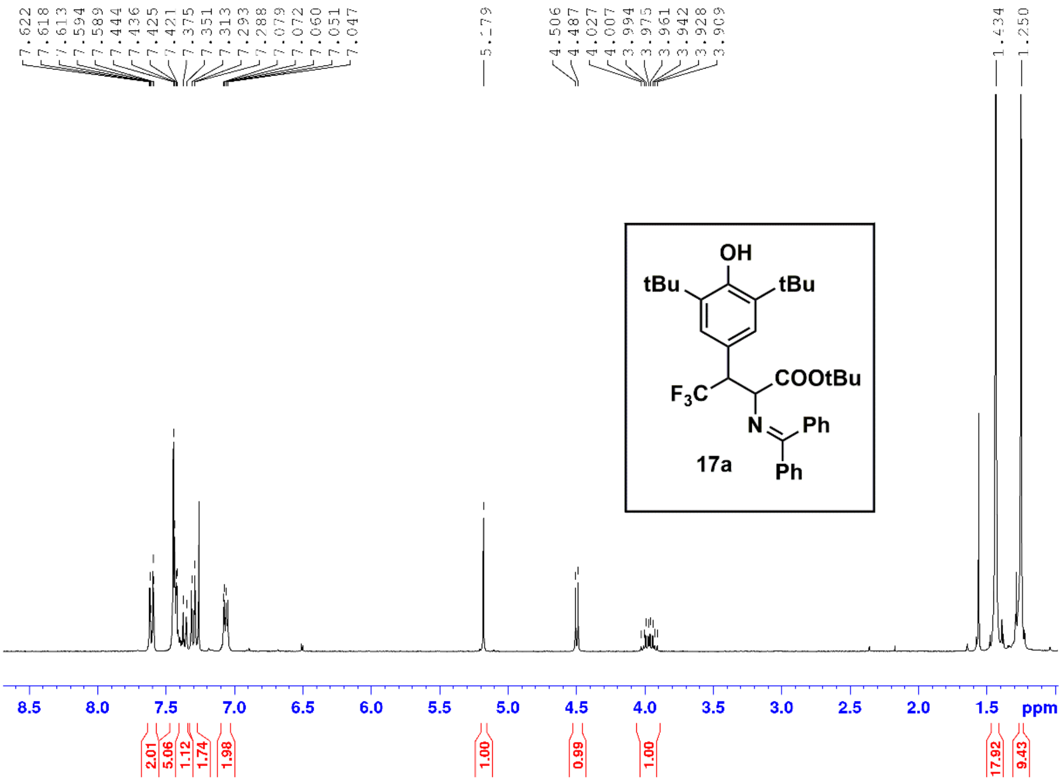
7.622
7.618
7.613
7.594
7.589
7.444
7.436
7.425
7.421
7.375
7.351
7.313
7.293
7.288
7.079
7.072
7.060
7.051
7.047
4.506
4.487
4.027
4.007
3.994
3.975
3.961
3.942
3.928
3.909
1.434
1.250
OH
tBu
tBu
COOtBu
F_3C
N
Ph
17a
Ph
8.5
8.0
7.5
7.0
6.5
6.0
5.5
5.0
4.5
4.0
3.5
3.0
2.5
2.0
1.5
ppm
2.01
5.06
1.12
1.74
1.98
1.00
0.99
1.00
17.92
9.43

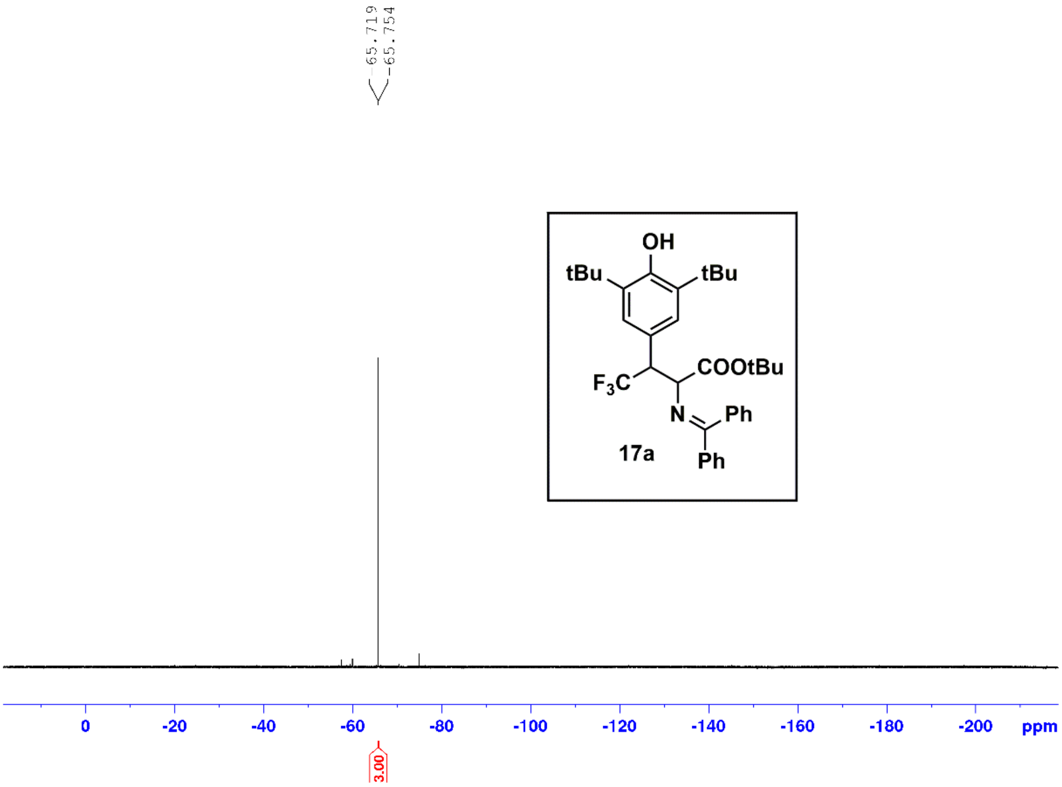
-65.719
-65.754
OH
tBu
tBu
COOtBu
F_3C
N
Ph
17a
Ph
0
-20
-40
-60
-80
-100
-120
-140
-160
-180
-200
ppm
3.00

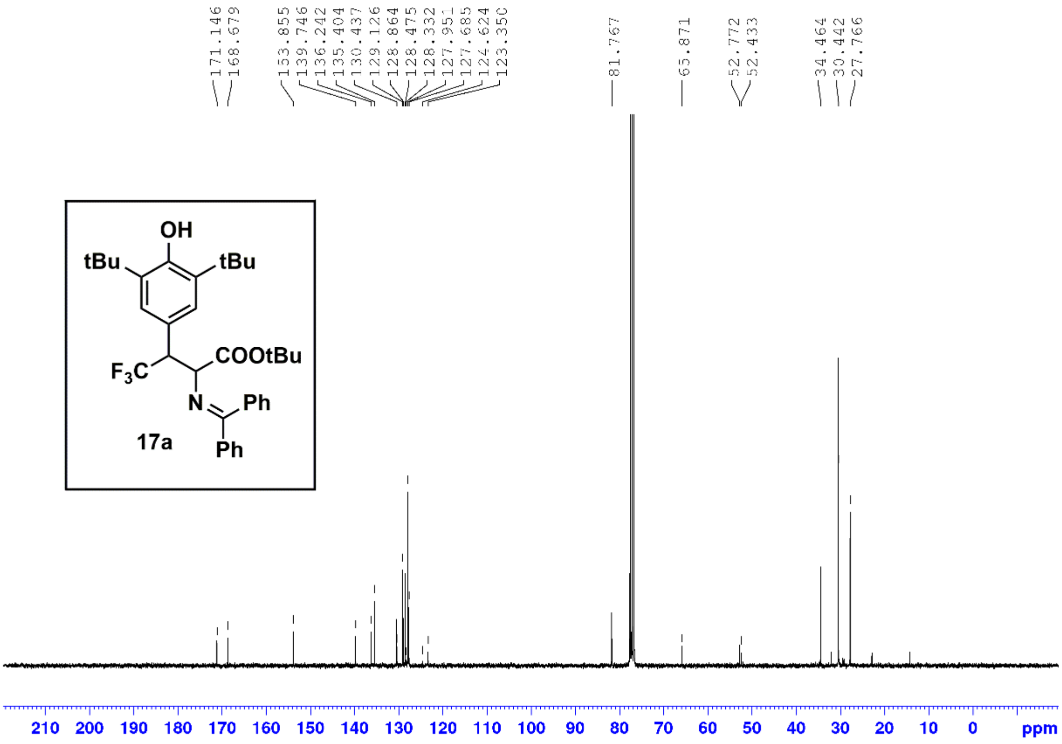
171.146
168.679
153.855
139.746
136.242
135.404
130.437
129.126
128.864
128.475
128.332
127.95
127.685
124.624
123.350
81.767
63.871
52.772
52.433
34.464
30.442
27.766
OH
tBu
tBu
COOtBu
F3C
N
Ph
Ph
17a
210 200 190 180 170 160 150 140 130 120 110 100 90 80 70 60 50 40 30 20 10 0 ppm

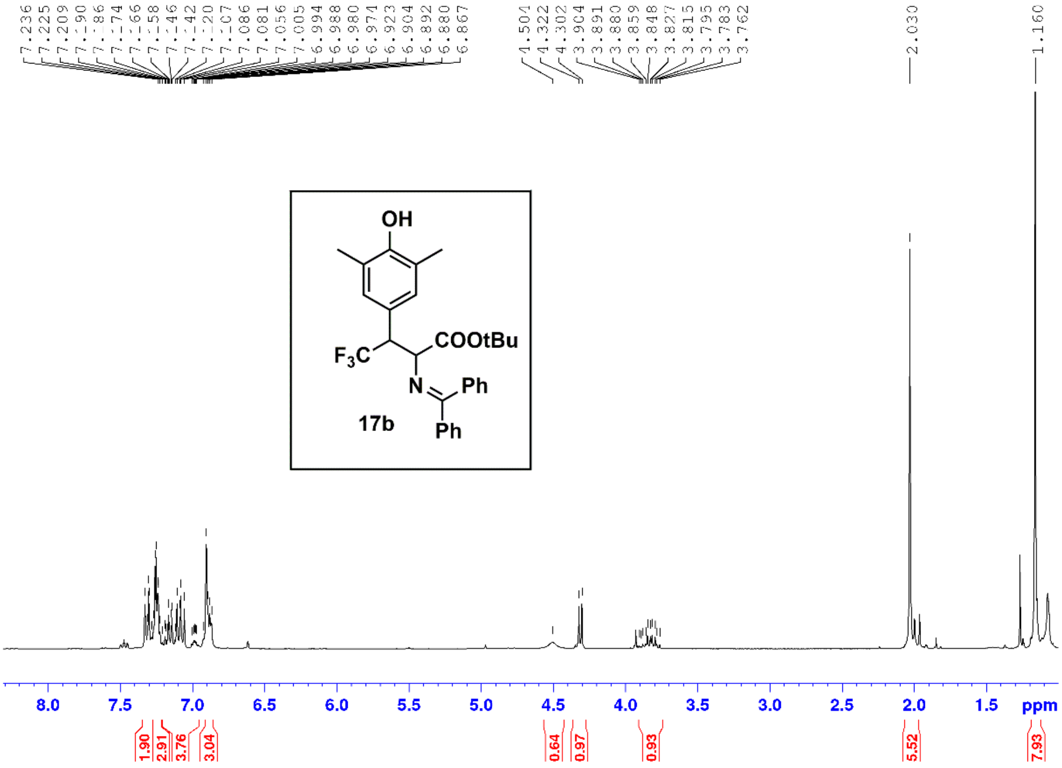
7.236
7.225
7.209
7.086
7.081
7.056
7.005
6.994
6.988
6.980
6.974
6.923
6.904
6.892
6.880
6.867
4.504
4.322
4.302
3.904
3.891
3.880
3.859
3.848
3.827
3.815
3.795
3.783
3.762
2.030
1.160
OH
COOtBu
F3C
N
Ph
Ph
17b
8.0 7.5 7.0 6.5 6.0 5.5 5.0 4.5 4.0 3.5 3.0 2.5 2.0 1.5 ppm
1.90
2.91
3.76
3.04
0.64
0.97
0.93
5.52
7.93

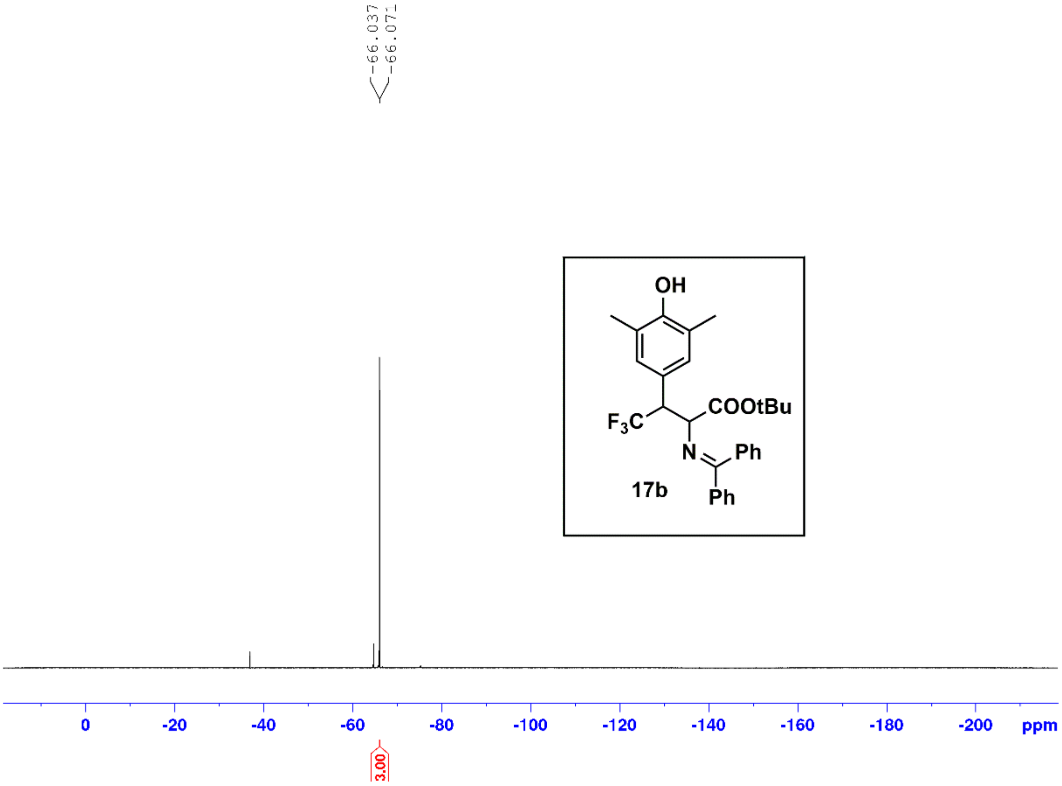
-66.037
OH
COOtBu
F3C
N
Ph
17b
Ph
0 -20 -40 -60 -80 -100 -120 -140 -160 -180 -200 ppm
3.00

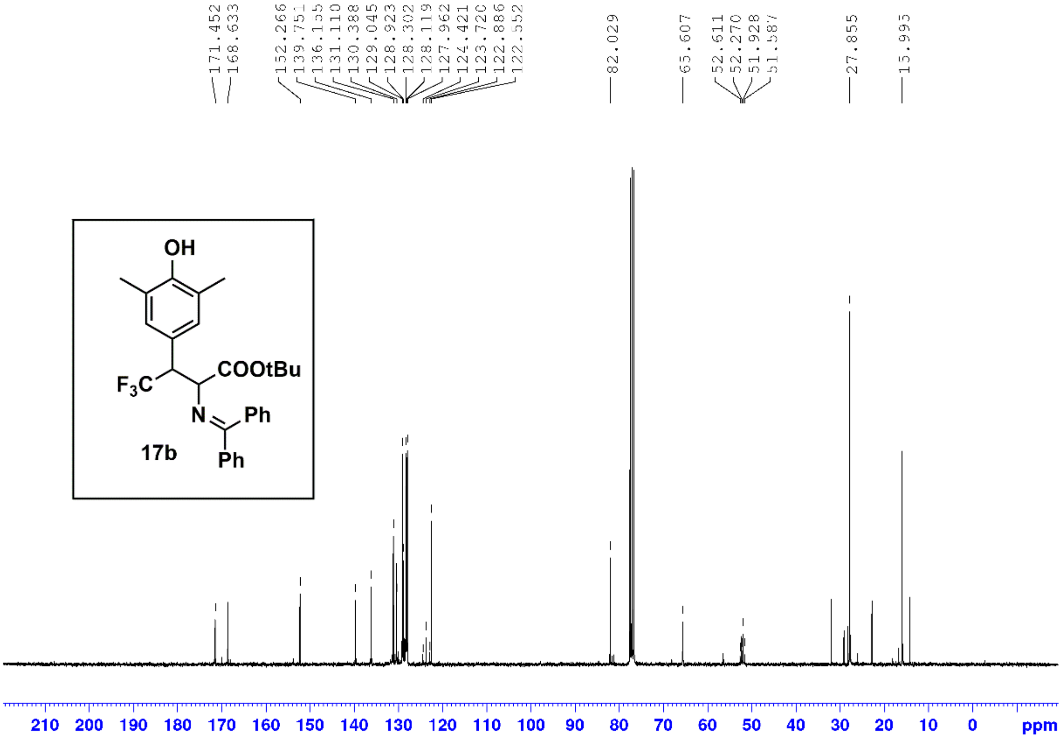
171.452
168.633
152.266
131.110
130.388
129.045
128.923
128.302
128.119
127.962
124.421
123.720
122.886
122.552
82.029
65.607
52.611
52.270
51.928
27.855
15.995
OH
COOtBu
F3C
N
Ph
17b
Ph
210 200 190 180 170 160 150 140 130 120 110 100 90 80 70 60 50 40 30 20 10 0 ppm

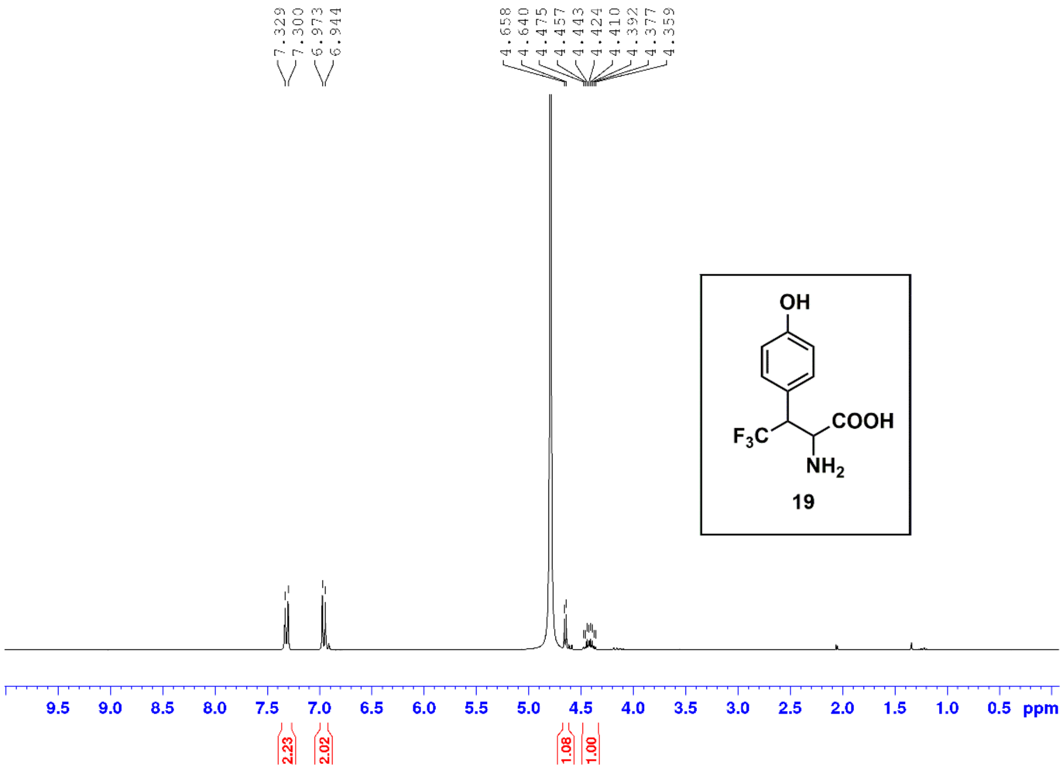
7.329
7.300
6.973
6.944
4.658
4.640
4.475
4.457
4.443
4.424
4.410
4.392
4.377
4.359
OH
F3C
COOH
NH2
19
9.5 9.0 8.5 8.0 7.5 7.0 6.5 6.0 5.5 5.0 4.5 4.0 3.5 3.0 2.5 2.0 1.5 1.0 0.5 ppm
2.23
2.02
1.08
1.00

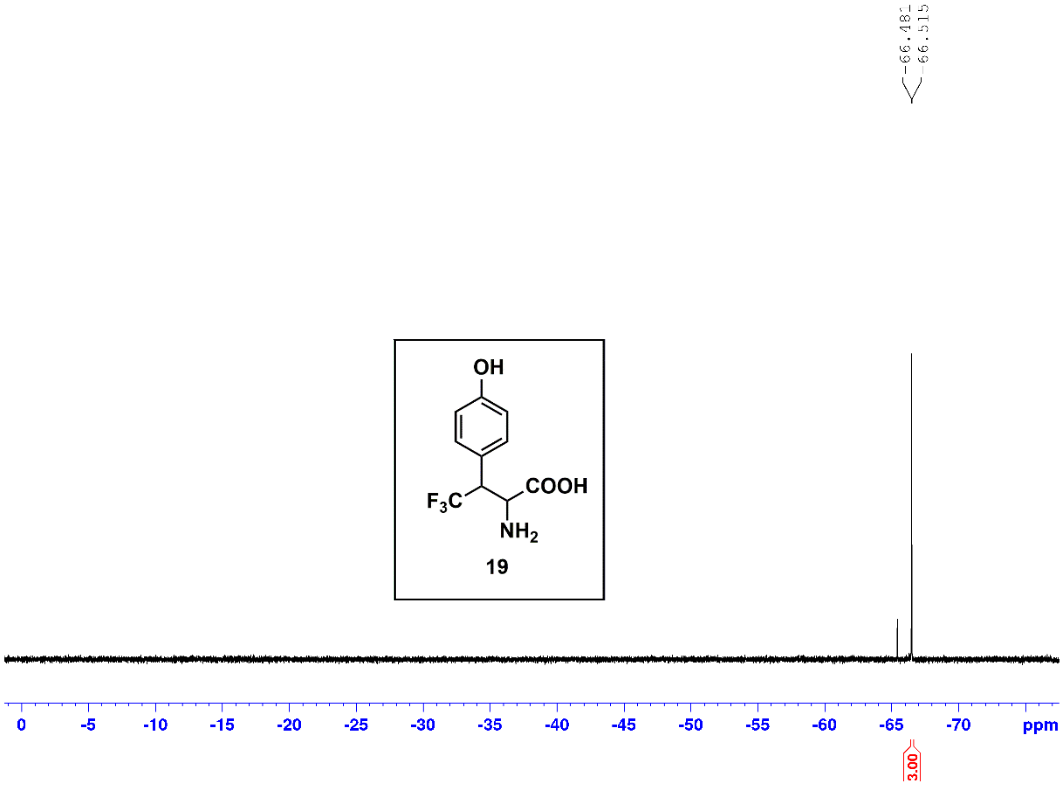
-66.515
OH
F3C
COOH
NH2
19
0 -5 -10 -15 -20 -25 -30 -35 -40 -45 -50 -55 -60 -65 -70 ppm
3.00

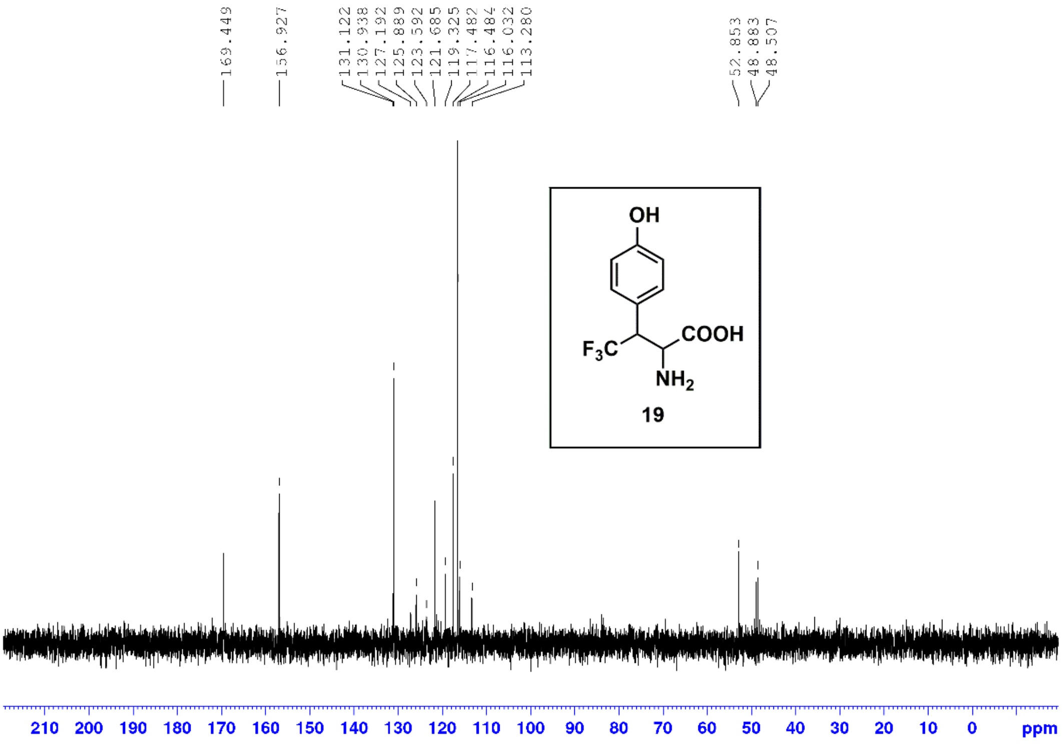
169.449
156.927
131.122
130.938
127.192
125.889
123.592
121.685
119.325
117.482
116.484
116.032
113.280
52.853
48.883
48.507
OH
COOH
F3C
NH2
19
210 200 190 180 170 160 150 140 130 120 110 100 90 80 70 60 50 40 30 20 10 0 ppm

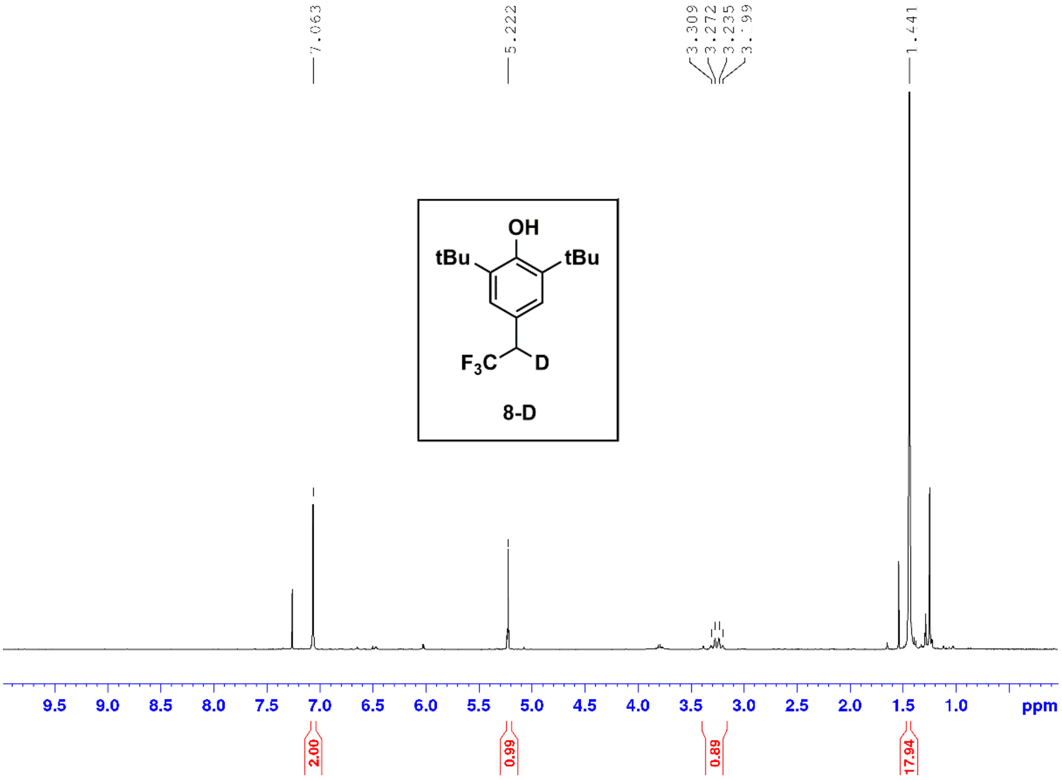
7.063
5.222
3.309
3.272
3.235
1.441
OH
tBu
tBu
F3C
D
8-D
9.5 9.0 8.5 8.0 7.5 7.0 6.5 6.0 5.5 5.0 4.5 4.0 3.5 3.0 2.5 2.0 1.5 1.0 ppm
2.00
0.99
0.89
17.94

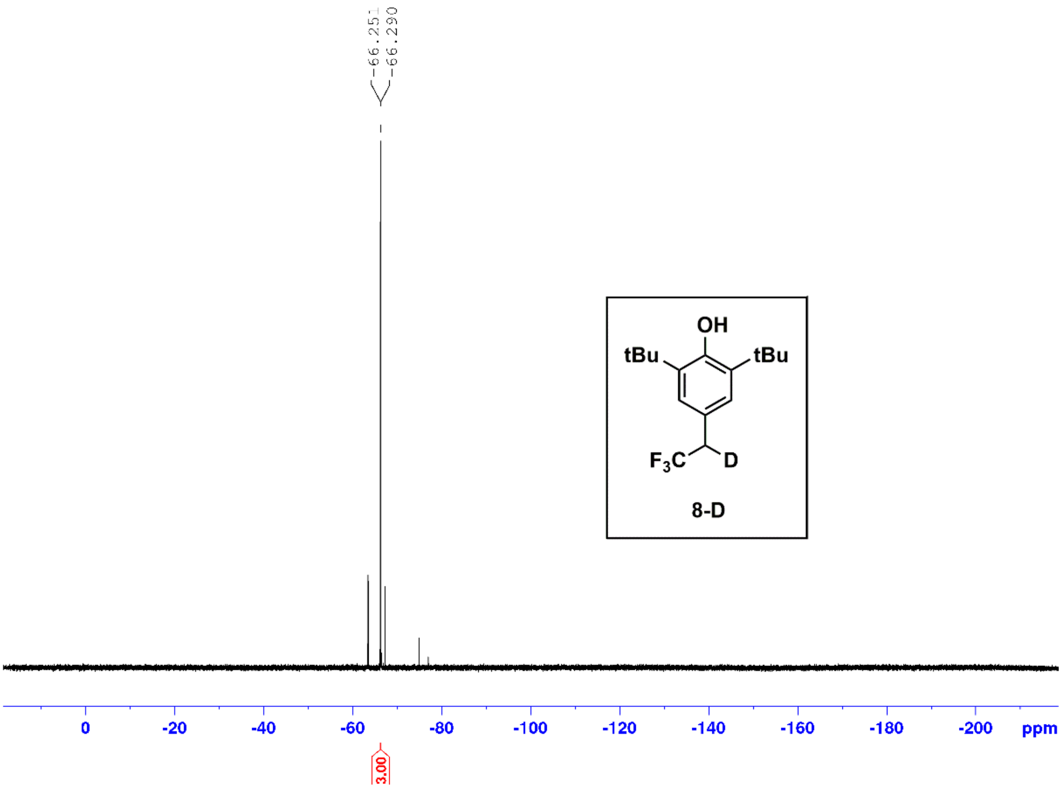
-66.251
-66.290
OH
tBu
tBu
F3C
D
8-D
0
-20
-40
-60
-80
-100
-120
-140
-160
-180
-200
ppm
3.00

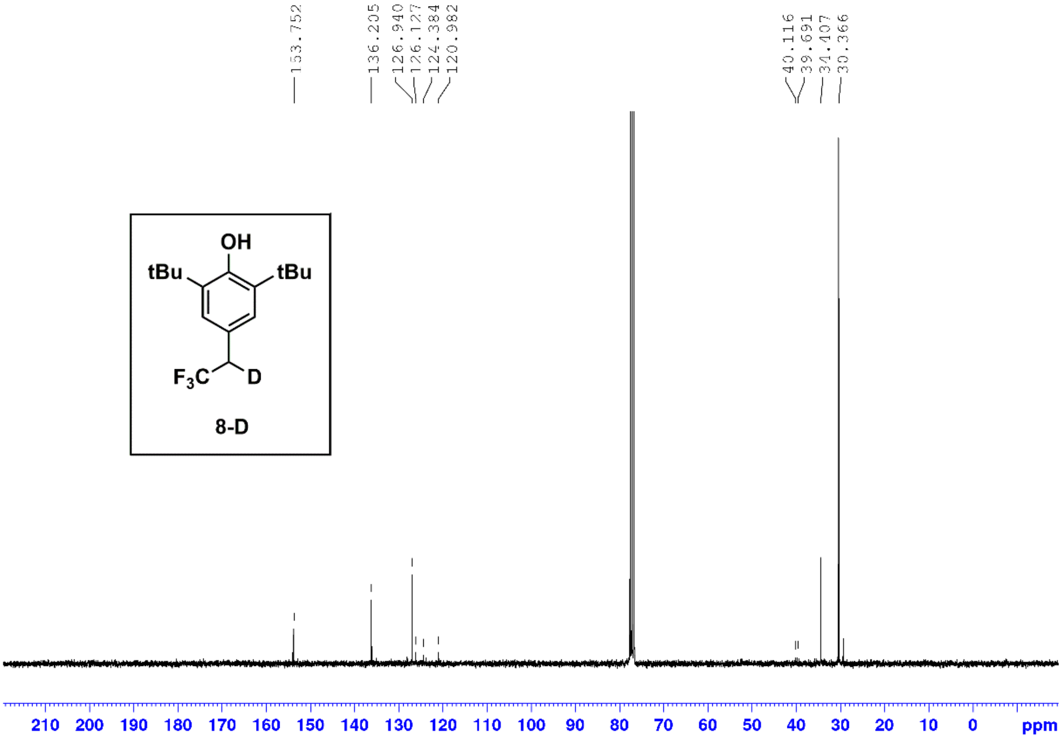
153.752
136.205
126.940
126.127
124.384
120.982
40.116
39.691
34.407
30.366
OH
tBu
tBu
F3C
D
8-D
210
200
190
180
170
160
150
140
130
120
110
100
90
80
70
60
50
40
30
20
10
0
ppm
